# Sieving for Gold—An Efficient Method for Generating *N*‑Heterocyclic Carbene Self-Assembled Monolayers on Nanostructured Gold Surfaces

**DOI:** 10.1021/jacs.5c15471

**Published:** 2025-11-18

**Authors:** Matteo Albino, Dimitar Georgiev, Ines Silva, Thomas F. F. Fernandez Debets, Mengwei Liu, Tristan N. Dell, Jonathan P. Wojciechowski, Molly M. Stevens

**Affiliations:** † Department of Materials, Department of Bioengineering, and Institute of Biomedical Engineering, 4615 Imperial College London, London SW7 2AZ, U.K.; ‡ Department of Physiology, Anatomy and Genetics, Department of Engineering Science, Kavli Institute for Nanoscience Discovery, 6396 University of Oxford, Sherrington Rd, Oxford OX1 3QU, U.K.; § Department of Computing and UKRI Centre for Doctoral Training in AI for Healthcare, Imperial College London, London SW7 2AZ, U.K.; ∥ BioInnovation Institute Foundation, Ole Maaløes Vej 3, Copenhagen N 2200, Denmark

## Abstract

*N*-Heterocyclic carbene self-assembled monolayers (NHC-SAMs) are an emerging class of ligands for metal surfaces with impressive chemical stability, proving vastly superior in specific applications compared to their thiol counterparts. Yet, unlike the latter, methods of forming such monolayers have poor functional group tolerability and require harsh or expensive reagents. Using a surface-enhanced Raman spectroscopy (SERS)-led optimization, we have developed a solution deposition methodology that relies on low-cost and easily accessible starting reagents. The addition of an external bicarbonate source greatly expanded the functional group tolerability, circumventing the need for the isolation of the benzimidazolium hydrogen carbonate. Additionally, inclusion of scavengers during the deposition, namely, molecular sieves (5 Å), improved monolayer formation and long-range ordering via the sequestration of CO_2_, a key side-product in the equilibrium between benzimidazolium salts with the hydrogen carbonate anion and their corresponding carbene. The methodology is operationally accessible, has broad functional group tolerability, and enables access to a wide substrate scope of NHCs using commonly available reagents.

## Graphical abstract

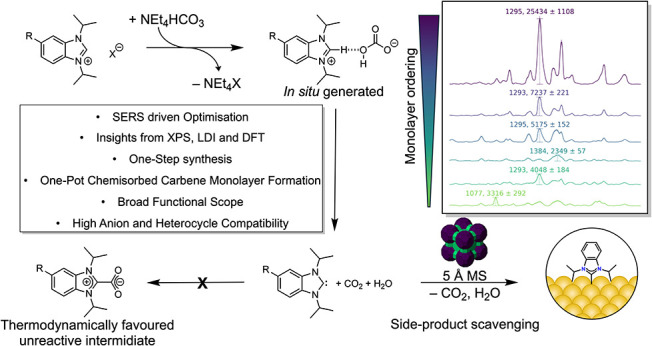

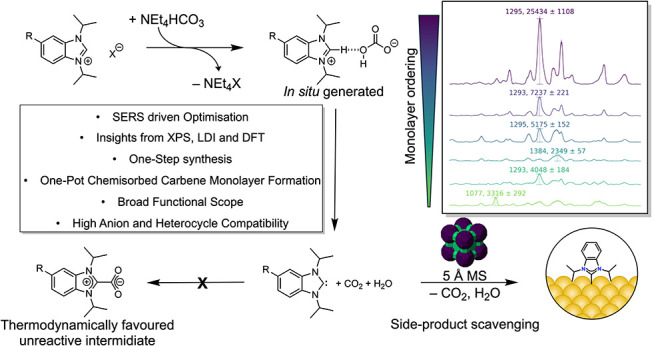

## Introduction

*N*-Heterocyclic carbene self-assembled monolayers (NHC-SAMs) have attracted significant attention due to their superior chemical and thermal stability on metal surfaces compared to thiols^1^ and their ability to functionalize materials beyond metallic surfaces.^2–4^ They demonstrate improved performance in molecular junctions,^5^ as SPR biosensors,^6^ organic field-effect transistors,^7^ on-surface photoswitches,^8^ and catalysis,^9,10^ among other applications.^1,6,11–18^ There are several methods that have been reported for the formation of NHC-SAMs. Deprotonation at the 2-position of the benzimidazolium halide salt by a strong base followed by incubation with a metal surface is typically utilized.^1^ Seminal work by Crudden and co-workers demonstrated that HCO_3_^–^ benzimidazoliums can spontaneously form SAMs on gold surfaces. This is attributed to a combination of the poor coordinating ability and weak affinity of the HCO_3_^–^ anion to gold compared to halides^6^ and the innate ability of benzimidazolium hydrogen carbonate species to be in equilibrium with the 2-carboxylate species *via* the respective carbene, water, and CO_2_ (Figure 1).^6,19^

**Figure 1:**
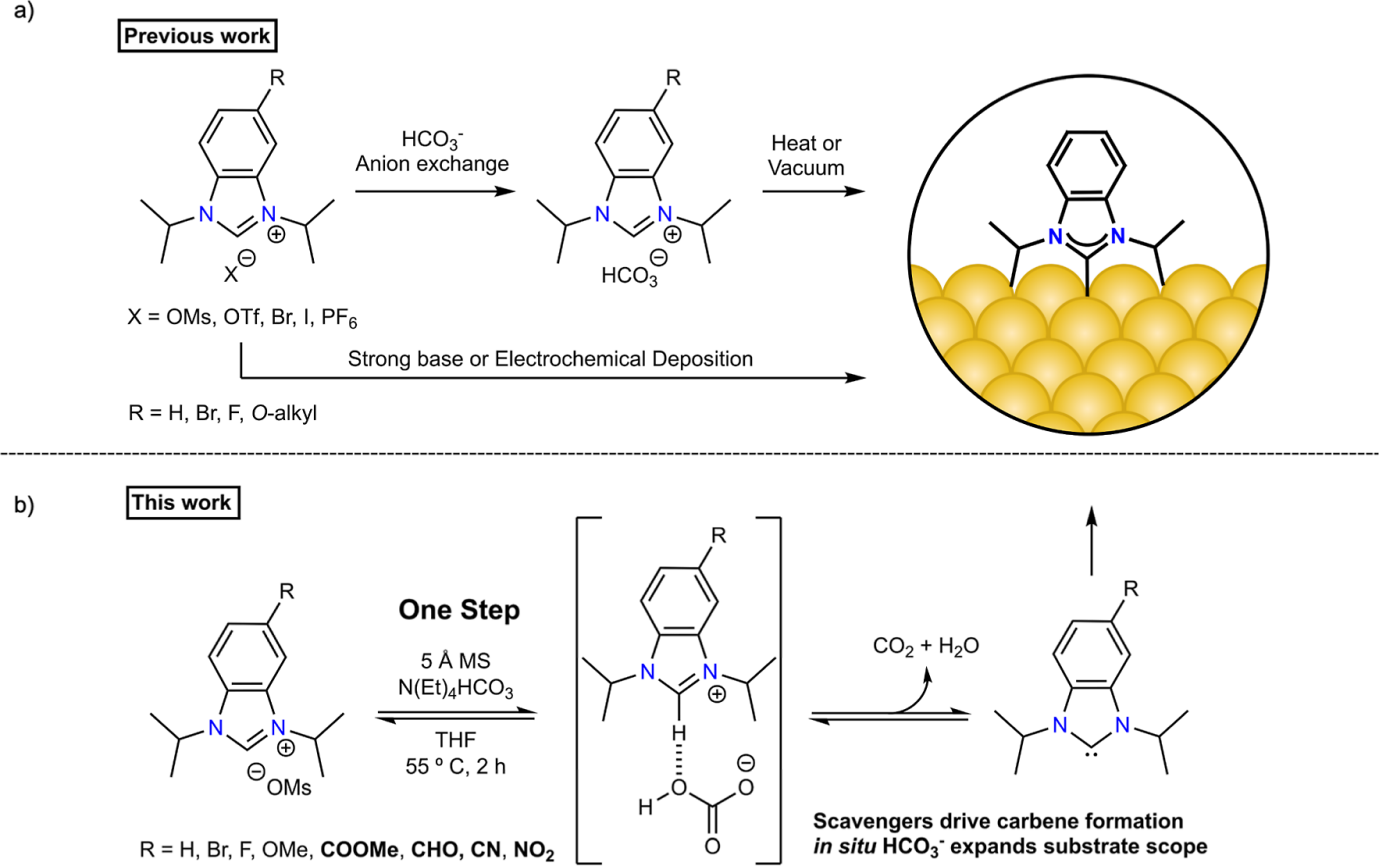
(a) Methods for the formation of chemisorbed carbene monolayers from benzimidazolium salt precursors utilized in the literature. (b) This work highlights the advantages of utilizing an external bicarbonate source to expand the functional group compatibility and scavengers to drive carbene formation and improve monolayer ordering.

Although there is some evidence that other weakly coordinating anions are capable of forming monolayers (i.e., triflate or methanesulfonate), the mechanism is poorly understood.^11,20,21^ Recently, Camden and co-workers reported that using these anions results in exclusively physiosorbed carbene monolayers, with significantly different SERS spectra compared to other methods, and no evidence of chemisorbed NHCs observed *via* laser desorption/ionization mass spectrometry (LDI-MS).^22^ In addition, Lee and co-workers had already observed a lack of long-range order in carbene monolayers formed using this method *via* polarization modulation infrared reflection absorption spectroscopy (PM-IRRAS) where the stretching frequency of alkyl chains in the monolayer was described as “liquid-like” and “conformationally floppy”.^20^ Although all these methods have been used interchangeably within the literature, Kang et al. observed a significant difference in the charge tunneling properties of carbene monolayers depending on whether the deposition was achieved *via* incubation with the PF_6_^–^ salt, by forming the carbene *in situ* with a strong base, or generated electrochemically. Hence, the authors postulated that the method of monolayer formation has drastic effects on its inherent chemical and physical properties.^23^ This was elegantly confirmed by Jenkins, Camden, and co-workers, who systematically studied the electrochemical properties, SERS, X-ray photoelectron spectroscopy (XPS), and LDI-MS spectra, showing that benzimidazolium salts of weakly coordinating anions cannot spontaneously form high-quality chemisorbed monolayers, unless heated or treated with a strong base.^22^ In addition, hydrogen carbonate benzimidazolium salts benefit from annealing at slightly elevated temperatures (55 °C).^22,24^

NHC-monolayers can be formed from the CO_2_ adduct under ultrahigh vacuum conditions,^25^ electrochemically,^26^ or by the leaching of precious metal–carbene molecular species onto a surface.^27–29^ Nevertheless, all these methods have limitations such as the utilization of strong bases,^1,25^ specialized equipment,^25,26^ or expensive reagents.^27,28^ Hence, the incubation of metal surfaces with HCO_3_^–^ benzimidazolium precursors arguably remains the most operationally efficient methodology.^6^ Carbene monolayers also allow for post deposition modifications of the monolayer, with a range of transformations now reported in the literature.^14,20,25,27,29,30^ However, due to the basicity of the HCO_3_^–^ anion, this method has poor applicability to base-unstable functional groups, such as nitriles,^25^ or protic groups in general.^25,28^

Herein, we report a methodology that circumvents the necessity for isolation of the bicarbonate salt and utilizes precursors synthesized from a single step, thus significantly improving the versatility, compatibility, and efficiency of HCO_3_^–^ benzimidazolium precursors for solution-phase deposition. We achieved this goal *via* a SERS-led optimization on nanostructured gold, demonstrating *in situ* generation of the HCO_3_^–^ salt combined with removal of CO_2_ and water to promote NHC-SAM formation, enabling a mild and efficient procedure to be achieved. We demonstrate that this optimized methodology is also applicable to *N*,*N*′-alkylimidazoles and *N*,*N*′-alkyl-1,2,3-triazoles, expanding the available scope of *N*-heterocyclic carbenes which can be used to modify gold surfaces.

## Results and Discussion

### Deposition of Hydrogen Carbonate Benzimidazoliums

We synthesized the methanesulfonate salts (**1**–**8**) from isopropyl methanesulfonate and the corresponding benzimidazole in a single step according to Scheme 1. We tested these precursors utilizing previously reported conditions for NHC-SAM formation on nanostructured gold surfaces and used SERS to confirm the formation of a monolayer. While XPS has often been considered the gold standard for characterizing NHC-SAMs, this technique has significant limitations when studying NHC-SAMs. For example, different deposition methods have been shown to yield significantly different SERS and LDI-MS spectra while presenting almost identical N 1s XPS spectra, and methods which displayed similar chemical characteristics had slightly different N 1s XPS spectra.^22^ We utilized nanopillar structured gold because of the high sensitivity when compared to other SERS-based sensors.^31^ However, under previously published conditions, we obtained weakly scattering monolayers when using **1a**, the methanesulfonate derivative,^11,20^ with no spectral features that resemble the previously reported SERS spectra for these NHC-SAMs (Figure S1).^25,32,33^ This confirms the results obtained by Kang et al. on flat Au(111),^23^ and more recently by Camden and co-workers, on both nanostructured and flat surfaces.^22^ Applying the conditions described by Glorius and co-workers which were optimized for the hydrogen carbonate derivative (55 °C in ethanol) did not improve the formation of well-defined monolayers on nanopillar gold (Figure S1).^24^ Hence, we also observe that benzimidazolium salts of weakly coordinating anions do not seem to provide a direct route for the formation of carbene monolayers.

**Scheme 1:**
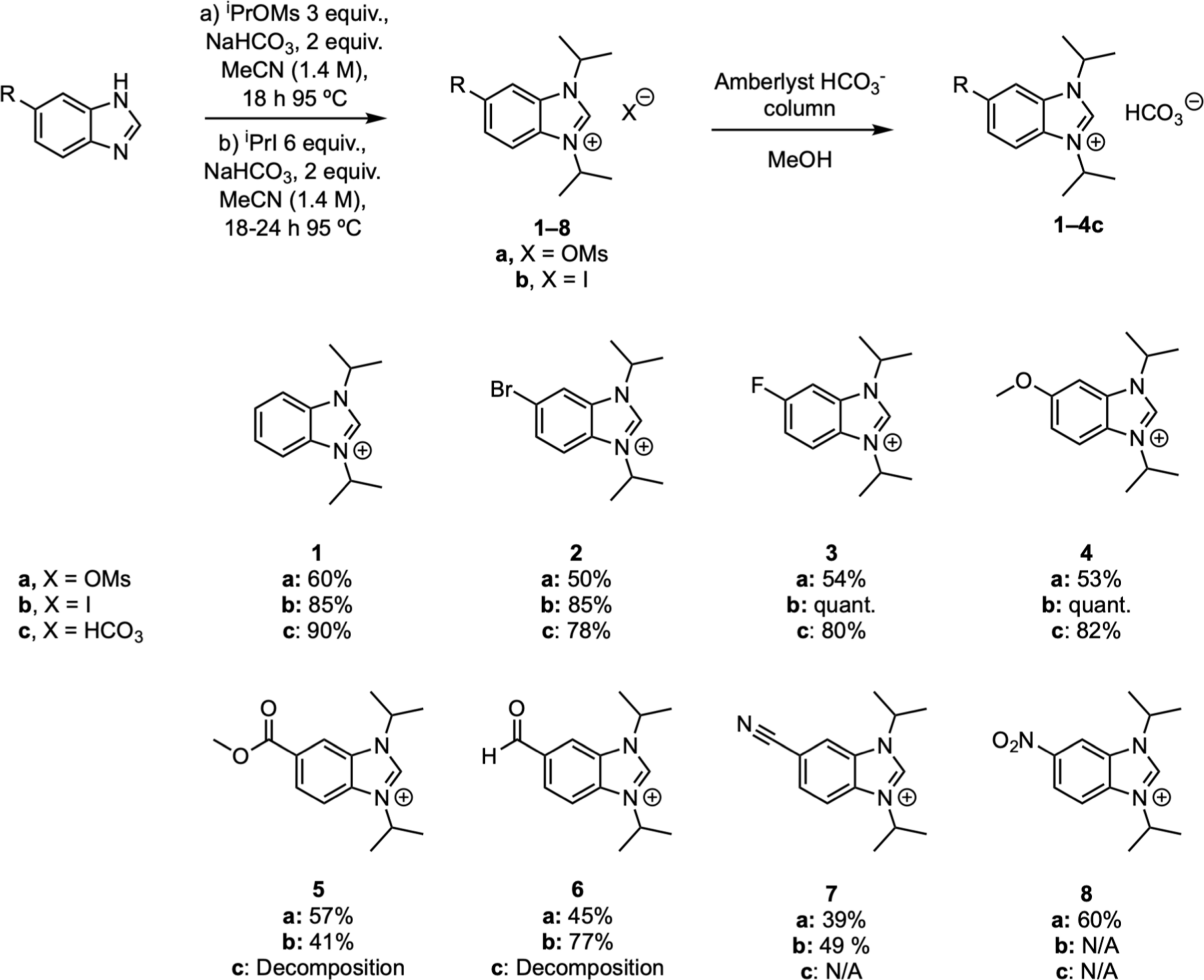
Synthesis of Benzimidazolium Precursors *via* *N*-Alkylation from the Corresponding Halide or Mesylate^a^

a Anion exchange using an Amberlyst HCO_3_^–^ resin gave the HCO_3_^–^ derivatives where possible.

We then synthesized the corresponding iodide salt and, after anion exchange, obtained the corresponding HCO_3_^–^ precursor, **1c**. Incubation of a 1 mM solution of **1c** in methanol at 20 °C for 24 h provided NHC-SAMs with spectral features in agreement with previously reported data (Figure S1).^22,25,32,33^ However, we obtained spectra with relatively broad features, indicative of a monolayer with poor organization.^6^ Additionally, the Raman shift at *ca.* 800 cm^–1^ which has been assigned to a vibrational mode with a significant contribution from the NHC–C–Au bond was low in scattering intensity, indicating a mostly physiosorbed population.^22,34^ Increasing the concentration of **1c** from 1 to 5 mM under the same conditions also gave spectra with poor Raman scattering intensities (Figure S1).

Increasing the incubation temperature from 20 to 55 °C for 2 h provided spectra with significantly sharper features in agreement with previous reports (Figure 2)^24^ and importantly an increase in the intensity of the vibrational mode at 800 cm^–1^. Longer or shorter reaction times provided very little spectral differences (Figure S2), if not for some small changes in the intensity, possibly due to an orientation change, similar to what was previously reported using sum-frequency generation (SFG) spectroscopy.^24^ Recent reports have demonstrated the dynamic nature of carbenes bound to metal surfaces, specifically Au(111). It was initially postulated that wing-tip size (*i.e.*, *via* *N*,*N*′-substitution on the benzimidazole) can play a significant role in the orientation of the NHC with respect to the metal surface.^35–37^ This was confirmed by Glorius and co-workers *via* a combination of XPS and SFG. However, the study also highlighted that temperature, concentration, and incubation time play a drastic role in the orientation of the NHC with respect to the metal surface. Increased reaction times, elevated temperatures, and higher concentrations all lead to the NHC-SAM preferring the thermodynamically favored tilted (for R = ^i^Pr) or flat (for R = Me) configurations with respect to the gold surface.^24^ Nevertheless, the small energy difference between the upright and tilted states may lead to a distribution of conformations, which has been supported experimentally by Camden and co-workers *via* SERS.^36^ Similar results were reported by Jiang and co-workers on Ag(111) surfaces, where a combination of scanning tunneling microscopy (STM) and tip-enhanced Raman spectroscopy (TERS) demonstrated a variability in configuration of the carbene as a function of temperature, with higher temperatures favoring tilted states,^38^ and Khera et al., where only flat-lying configurations were observed by low-temperature STM.^39^ Regardless of the tilt-angle, there seems to exist an agreement on the fact that the carbene binds to the adatom on the metal surface,^40^ providing explanation for their high mobility on the surface.^30^

**Figure 2:**
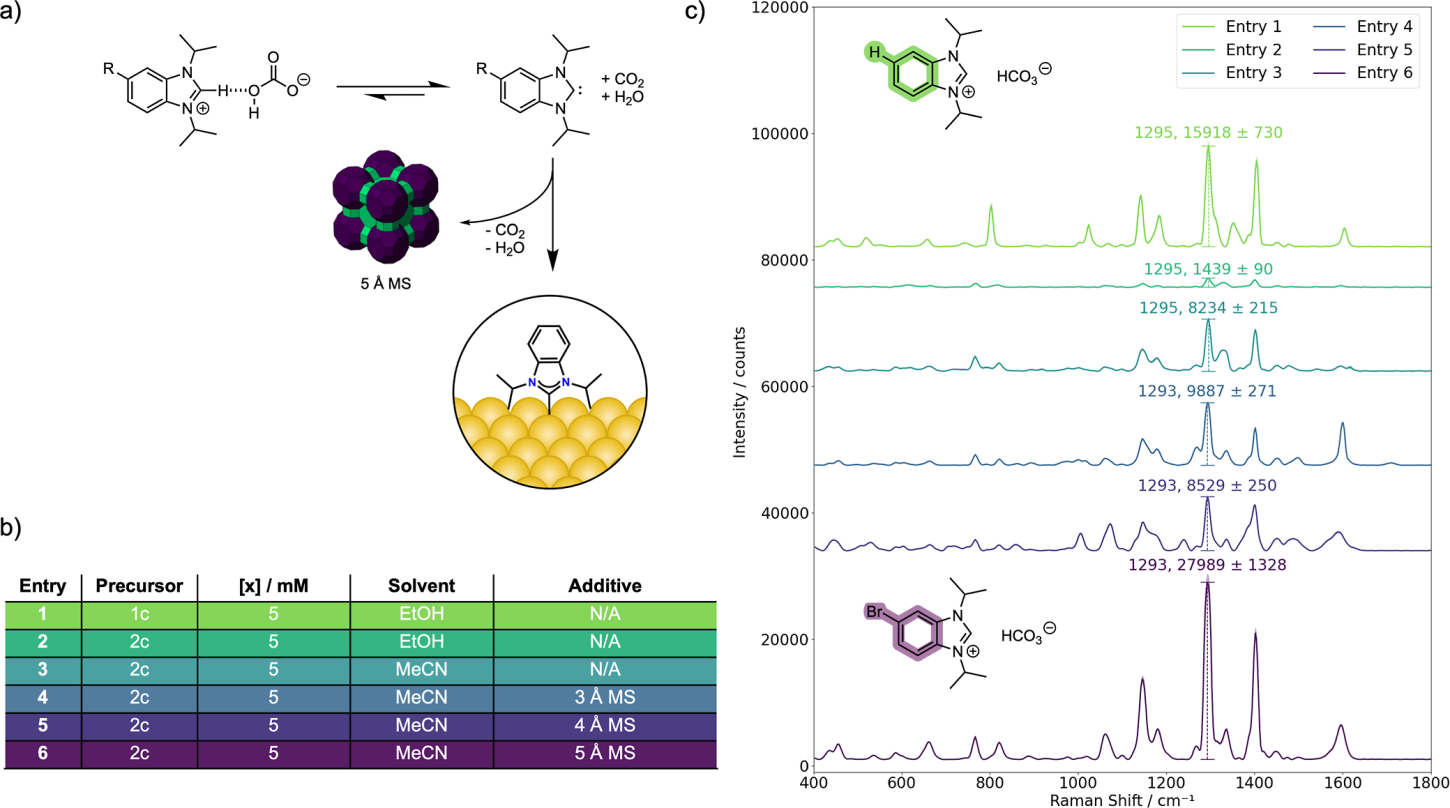
a) The deposition method, (b) table summarizing the conditions attempted, and (c) the corresponding SERS spectra obtained from each respective condition. All functionalizations were carried out at 55 °C. All spectra are the average of 15 measurements, 5 from 3 different SERS substrates, and the standard error of the mean is represented as the shaded region.

Encouraged by the results with the benzimidazolium derivative **1c**, we next tried with the bromide-functionalized precursor **2c**; however, the deposition was not as successful (Figure 2c). The monolayer was weakly scattering and showed little resemblance to literature reports on other nanostructured gold surfaces,^25^ highlighting the challenging nature of functionalizing gold nanopillars.^13^ Increasing incubation times to 24 h and decreasing the concentration had a beneficial effect and increased the scattering intensity (Figure S3), but the intensity still remained lower than **1c**.

Not satisfied by the quality of the spectra, we decided to revisit the work by Fèvre et al. where the equilibrium between the HCO_3_^–^ benzimidazolium salts and their corresponding 2-carboxy derivatives *via* the free carbene was initially reported, noticing that in polar aprotic solvents such as DMSO-*d*_6_, ^1^H NMR studies showed both species coexisting in solution.^19^ This is in contrast to the utilization of polar, protic solvents where only the imidazolium is observable.^19^ Therefore, considering the equilibrium between the two species will be fast at 55 °C due to a relatively low energy barrier,^19^ we reasoned that polar aprotic solvents might shift the equilibrium favorably toward the 2-carboxy species (and hence the carbene), therefore improving the formation of SAMs which will act as a thermodynamic or kinetic sink, driving further production of the carbene. Incubation for 2 h in acetonitrile at 55 °C for a 5 mM solution of **2c** supported this hypothesis, as it resulted in significantly improved monolayers compared to ethanol (Figure 2c), with a stark increase in the signal intensity.

Motivated by this result, we studied the equilibrium further. Upon deprotonation of **2c** by HCO_3_^–^, water and CO_2_ are generated.^19^ Formation of the monolayer already serves as a thermodynamic or kinetic sink for the production of further free carbenes; however, we reasoned that sequestration of the side products (namely, water and CO_2_) could also be beneficial to promote generation of the carbene. We therefore tested different sizes of molecular sieves (MS), as these are known to sequester small molecules such as H_2_O and CO_2_. Interestingly, the addition of 5 Å MS to the mixture in acetonitrile caused a significant increase in the SERS scattering intensity of the monolayer while maintaining high fidelity in terms of spectral features to the previously reported data.^25^ The stretching frequencies at 1293 and 1400 cm^–1^, associated with the wingtip motion and the C–Au vibration,^41^ increased by more than 3-fold in intensity following the addition of this additive. In contrast, the 3 and 4 Å molecular sieves had essentially no beneficial effect (*vide infra*). It is worth noting that when acetonitrile was used as the solvent, a peak corresponding to the nitrile C≡N symmetrical stretching frequency was observed, which persisted even after drying (Figure S5).^42–44^ This is interesting as incorporation of the solvent in NHC-SAMs has not been reported, possibly due to the weak scattering ability of ethanol, methanol, and tetrahydrofuran which are routinely used. However, Camden and co-workers have reported XPS signals corresponding to KHMDS impurities in the N 1s spectra of an NHC-SAM formed *in situ*, hence incorporation of the solvent or base in the monolayer should be assessed.^22^

Selected conditions for **2c** were also studied by XPS (Section S8 and Figure S46) as the N_(∼400 eV)_/Au ratio can be used to examine the completion of monolayer formation. All N 1s spectra for the monolayers formed under optimized conditions (Figure 2, entry 6), heated deposition in acetonitrile without 5 Å MS (Figure 2, entry 3), heated ethanolic deposition without 5 Å MS (Figure 2, entry 2), or methanolic room temperature deposition were similar in terms of chemical shifts, with the major peak at *ca.* 400 eV associated with chemisorbed carbenes.^22,45^ A second peak at *ca.* 402 eV was also occasionally observed. The latter is associated with physisorption of quaternary ammonium impurities from the anion exchange resin that can be eventually removed after thorough washings.^45^ The optimized conditions (Figure 2, entry 6) also showed a third peak centered at 397 eV, which is consistent with a nitrile group,^46^ in agreement with the observed C≡N symmetrical stretching frequency in the SERS spectra. Interestingly, the XPS peak at 397 eV and the nitrile stretching frequency are not present in the XPS and SERS spectra, respectively, for monolayers deposited in acetonitrile without the addition of 5 Å MS (Figure S5).

However, there seems to be no correlation between the SERS signal intensity and surface coverage. The deposition in acetonitrile (Figure 2, entry 3) had a slightly higher N_(∼400 eV)_/Au ratio than the optimized conditions (Figure 2, entry 6). The latter did, however, have higher surface coverage than the heated ethanolic deposition (Figure 2, entry 2) or for monolayers formed under room temperature methanolic deposition. Hence, we hypothesized that the increase in intensity originates from a more ordered monolayer, formed by a beneficial increase in free carbene concentration in solution during the deposition that can chemically bind to the surface, driven by the biasing of the equilibrium from the sieves.

To confirm this hypothesis, we examined the chips post deposition under a scanning electron microscope (SEM, Figures S31–S33). The degree of nanopillar aggregation was indistinguishable for all conditions examined, suggesting the increase in SERS intensity does not originate from a morphological change to the nanostructures but rather from chemical effects.^31^ Since physiosorbed monolayers generated from triflate salts give an XPS signal identical to those generated by deposition of the CO_2_ adduct or *in situ* generated carbene in solution formed by strong-base deprotonation, both of which provide chemisorbed monolayers,^22^ we believe that although the N_(∼400 eV)/_Au ratios are similar, this value does not differentiate between the physiosorbed and chemisorbed NHCs present on the metallic surface. Recently, NHCs have also been shown to restructure gold surfaces by extraction of adatoms.^47^ The morphological change at the atomic level could also play a role in signal enhancement. Nevertheless, both of these hypotheses point to a more ordered monolayer, with an increase in chemisorbed NHCs.

Motivated by these results, we were interested in gaining a mechanistic understanding of how the 5 Å MS influenced the SAM formation and, therefore, the general NHC-SAM mechanism using HCO_3_^–^ salts precursors. Specifically, it is known that the porosity of molecular sieves influences selectively toward sequestering of water and/or CO_2_.^48–50^ Hence, we were interested in understanding which capture was providing more beneficial for SAM formation. 5 Å MS are extremely useful CO_2_ scavengers compared to the smaller 3 Å and 4 Å derivatives, which more selectively remove smaller molecules such as water.^48,50,51^ Hence, these results seem to suggest that CO_2_ removal played a central role in the equilibrium and, therefore, monolayer formation. This was further confirmed by a drastic decrease in the scattering intensity when the functionalization was carried out under a CO_2_-saturated system. By bubbling CO_2_ through acetonitrile and then using standard Schlenk techniques to carry the functionalization under 1 atm. of CO_2_, the SERS intensity of the monolayer almost halved (Figure S4, entry 1).

As discussed above, the exclusion of water was not as beneficial as that of CO_2_. When oven-dried glassware and anhydrous acetonitrile were utilized, the intensity was similar to that observed when no sieves and reagent grade acetonitrile were used (Figure S4, entry 6). Hence, it appears that water exclusion has very little influence on monolayer formation. The energy profiles generated using DFT (Section S9) also support this observation, wherein the formation of the 2-carboxy derivative compared to the free carbene is thermodynamically favored in acetonitrile for **2c** by *ca.* 10 kcal mol^–1^. Therefore, by removing water, the equilibrium is shifted further toward this thermodynamic sink. However, selective or preferential sequestration of CO_2_ would prevent formation of the 2-carboxy species and therefore allow for the generation of the free carbene. Nevertheless, we cannot rule out a synergistic effect for the sequestration of both CO_2_ and water.

With these optimized conditions, a small deposition scope study was performed (R = H, Br, OMe, and F), and all substrates were able to form high-scattering monolayers with strong Raman scattering intensities at 800 cm^–1^ confirming their chemisorbed identity (Figure S6). One limitation of this methodology was the requirement on the synthesis of the benzimidazolium hydrogen carbonate salt, posing significant limitations in the scope itself. For example, methyl ester **5b** and aldehyde derivative **6b** showed significant decomposition after anion exchange (Figures S7 and S8). Previously, DeJesus et al. have shown that monolayers formed from the nitrile derivative **7c**, after anion exchange from **7b**, give varying intensities of the nitrile stretching frequency.^25^ We speculate that this is due to the high basicity of the anion exchange conditions, generating transient alkoxide species that can act as nucleophiles when electron-withdrawing substituents activate the imidazolium ring system, generating a plethora of ring-opened structures as reported by others.^27,52^

Suspicious of the fact that drying of the sample could be the problematic step, we used an anion exchange column on **7b** in EtOH and then used the eluent directly for functionalization with 5 Å MS. This method did result in visible monolayer formation but with a significantly lower intensity (Figure S27).

### Deposition of Methanesulfonate Benzimidazoliums

We reasoned that if CO_2_ capture can drive the formation of the monolayer, an external HCO_3_^–^ source could possibly be utilized to generate the HCO_3_^–^ derivatives *in situ* and in the presence of 5 Å MS drive the equilibrium toward generation of the carbene. To test this hypothesis, we used **1a** as a model substrate, such that the benzimidazolium source would not contain any coordinating anions, namely, halides. Initially, we added NH_4_HCO_3_ as the bicarbonate source, but the poor solubility of this additive led to no monolayer formation (Figure S9). Therefore, we trialed an organic soluble derivative, tetraethylammonium bicarbonate (TEAB), as a cost-effective candidate readily available from commercial suppliers. Upon utilization of the optimized conditions with the addition of TEAB, 5 Å MS, and the mesylate salt **1a**, we observed well-organized and high-intensity monolayers (Figure S10). The relative intensity of the NHC–Au stretching frequency at 800 cm^–1^ was unchanged compared to **1c**, indicating that a similar level of chemisorption is possible using an *in situ* hydrogen carbonate source (Figure S10).^22,34^

When applying the optimized conditions (acetonitrile, 55 °C, 5 mM benzimidazolium, 5 mM TEAB) to other derivatives, namely, the methyl ester **5a** and nitrile **7a**, the monolayers formed had unusual, broad features and lacked the characteristic sharp vibrational features of di-isopropyl functionalized NHC SAMs at *ca.* 1300 and 1400 cm^–1^ (Figure S11).^25,41^ Hence, a second round of optimization screened solvents, TEAB equivalents, benzimidazolium concentration, and temperature, which are summarized in Figure 3b. More entries are included in Section S5 and Figures S12–S14. This systematic optimization highlights a number of significant findings. First, increased temperatures did not have any beneficial effects on monolayer formation (Figures S12–S14). Interestingly, the solubility of the mesylate derivative and hydrogen carbonate source does not seem to influence the outcome of the functionalization; instead, there seems to be an extremely intricate balance between the polarity of the solvent and the monolayer functionalization, with the former playing the most drastic role out of the parameters screened. Polar solvents such as acetonitrile, dimethylformamide, and ethanol, that fully solubilized both the benzimidazolium precursor and the TEAB, yielded monolayers of extremely poor quality. Less polar solvents in which the precursors had decreased solubility yielded monolayers with reinstituted fidelity to the previously published spectra but lower intensities.^25^ Tetrahydrofuran seemed to be the apex of this balance, with less polar substitutes, like 2-methyl tetrahydrofuran (Figure S14), decreasing the quality of the monolayer. Polar solvents could yield solvated ions with lower activity, while apolar solvents might result in poor solubilization of the starting materials and therefore a low concentration of carbene in solution. Importantly, under optimized conditions, the ester stretching frequency at *ca.* 1700 cm^–1^ maintained a high relative intensity compared to the other vibrations, indicating very little hydrolysis occurred and that this moiety was retained on the surface.

**Figure 3:**
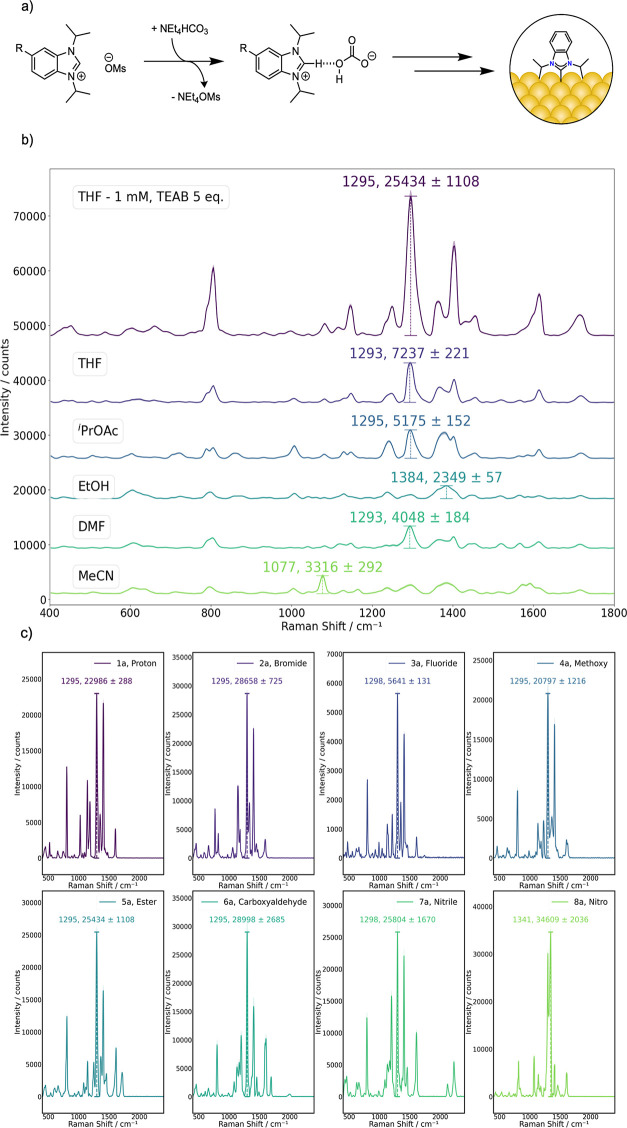
a) Deposition mechanism with mesylates; (b) SERS-led optimization using **5a**, all depositions were carried out at 55 °C, with 5 mM of benzimidazolium precursors and 1 equiv of TEAB, except for the last entry where 5 equiv was used. (c) Scope of **1–8a** using the conditions optimized for **5a**, except for **8a** where 2 equiv of TEAB was used. All spectra are the average of 15 measurements, 5 from 3 different SERS substrates, and the standard error of the mean is represented as the shaded region.

Upon utilization of these optimized conditions, a range of functionalities could be demonstrated which are not accessible *via* the synthesis of the hydrogen carbonate precursors, such as the aldehyde derivative **6a**, clearly displaying the carbonyl stretching frequency at *ca.* 1700 cm^–1^, and nitrile derivative **7a** (Figure 3c). In the SERS spectra of **7a**, there were two peaks within the C≡N symmetrical stretching frequency, an observation not previously reported.^25^ This could suggest some level of physisorption *via* the nitrile moiety, similar to what was observed when acetonitrile was previously utilized as a solvent. A peak corresponding to the physiosorbed nitrile species was, in fact, observed in the N 1s XPS spectra for this monolayer (Table S39). Nevertheless, nitrile-containing NHC-SAMs have been difficult to achieve in the literature, as methods to incorporate the HCO_3_^–^ in the benzimidazolium precursor led to significant decomposition of this functional group. DeJesus et al. elegantly circumvented the need for the bicarbonate anion *via* isolation of the 2-carboxylate species.^25^ However, this methodology required the utilization of glovebox chemistry and harsh reagents; therefore, this substrate highlights the advantages of our methodology. As reported by Camden and co-workers, the methyl ester (**5a**) and nitro derivative (**8a**) once deposited on the surface could also be hydrolyzed to reveal the carboxylic acid or reduced to the amine, respectively (Figures S19 and S20).^25,27^ These functional groups specifically are useful reactive handles for post deposition modification and bioconjugation reactions.^11,53,54^

The importance of the solvent is also supported by DFT studies (Figure 4a and Section S9). Upon increasing solvent polarity, the energies of the benzimidazolium and the bicarbonate are systematically decreased, therefore decreasing the thermodynamic driving force to form the free carbene and the 2-carboxy adduct. In contrast, the energies of the neutral and zwitterionic species change less as a function of solvent polarity. For **5**, the equilibrium in tetrahydrofuran favors the 2-carboxy adduct by 7.3 kcal mol^–1^, while the benzimidazolium is favored in both acetonitrile and ethanol by 2.7 and 3.5 kcal mol^–1^, respectively. Interestingly, even the formation of the free carbene itself is favored in tetrahydrofuran by 4.8 kcal mol^–1^ compared to the benzimidazolium hydrogen carbonate. These results also explain why CO_2_ sequestration seems to be more influential than H_2_O removal. Quenching the formation of the 2-carboxy adduct that acts as a thermodynamic sink can only be achieved by removal of the CO_2_, while removal of H_2_O only biases the equilibrium away from the benzimidazolium salt.

**Figure 4:**
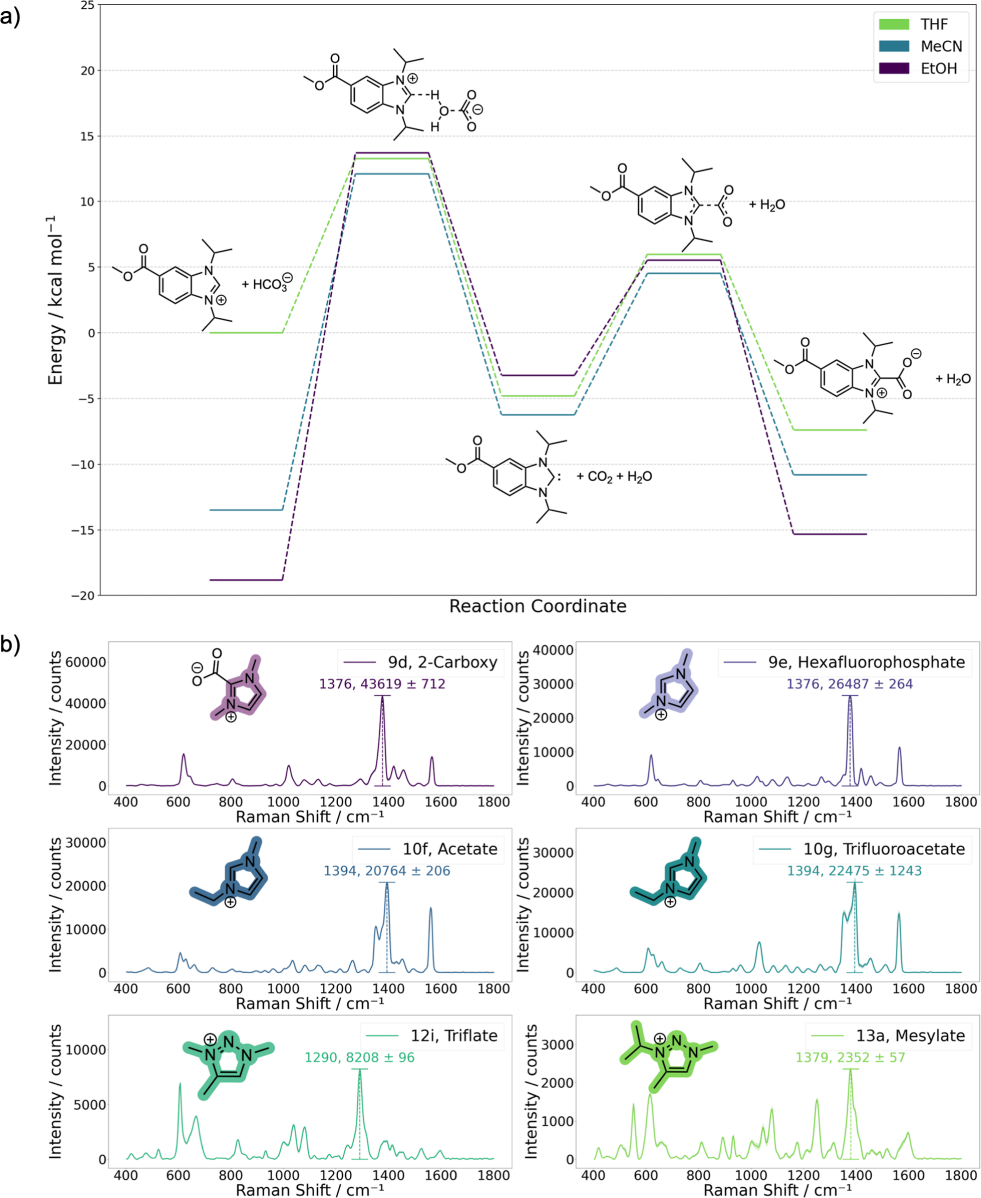
a) DFT-energy profile at the PBE0/AUG-cc-pVTZ/GD3BJ/SMD level of theory for the equilibrium between **5** and the hydrogen carbonate anion, the respective free carbene, and the 2-carboxy species at 328.15 K; (b) orthogonal scope using different heterocyclic frameworks. All spectra are the average of 15 measurements, 5 from 3 different SERS substrates, and the standard error of the mean is represented as the shaded region.

Additionally, increased solvent polarity can also diminish hydrogen bonding interactions between the acidic benzimidazolium proton and anions in solution: this reduces polarization of the said proton and prevents the bicarbonate anion and the benzimidazolium salt from reacting to form the free carbene in solution.^22,55,56^ Nevertheless, the energy profiles reveal little difference between derivatives **1**, **2**, and **5**. Hence, we speculate that the increased difficulty in forming the monolayers might rather be due to the strength of the carbene donor, which decreases systematically as the electron-withdrawing capability of the R substituent increases.^56^

Due to the heterogeneity of the reaction between the sieves, the surface, and the benzimidazolium, stirring is extremely beneficial. When the functionalization is carried out without any agitation, different levels of deposition are observed. Figure S14, entry 6, shows the SERS spectra obtained from successfully functionalized regions of the surface. Figure S15 shows the spectra obtained from the remaining regions, revealing a significant difference to the level of functionalization when the reaction is not stirred. Other reports highlight the importance of stirring in heterogeneous processes.^57^

An excess of TEAB was found to be beneficial, but this reaches saturation at 5 mol equiv to the benzimidazolium (Figures 3 and S14, entries 10, 11, and 14). Camden and co-workers showed a downfield shift in the benzimidazolium acidic 2-proton for the ^1^H NMR spectra of **1c** in DMSO-*d*_6_ when the concentration was increased, suggesting an increase in bicarbonate concentration might polarize the acidic proton *via* hydrogen bonding.^22^ This process could be beneficial due to lowering of the p*K*_a_ of said proton, allowing more facile generation of the carbene. Nonetheless, an excess of bicarbonate might cause issues due to an increase in the pH of the solution, or the insoluble salt covering the surface, preventing functionalization. It is noteworthy that under the newly developed conditions, the intensity of the peak at 806 cm^–1^ (that has a high contribution from the Au–C bond)^22,34^ grows proportionally with the other peaks, suggesting chemisorption is the predominant deposition mechanism (Figure 3b). The concentration of the starting benzimidazolium salt is also extremely important: at 1 mM, the SERS signal reaches a maximum, with the intensity dropping significantly for both more and less concentrated deposition solutions (Figure S16).

To evaluate the grafting efficiency further, LDI experiments were conducted with the optimized methodology and with variations excluding the bicarbonate source and 5 Å MS or both (Section S6). Both conditions with the bicarbonate source showed the mass of the cation bis-ligand gold species, L_2_Au^+^, indicative of a monolayer chemisorbed to gold^22,53,54^ (Figures S34 and S35). In the conditions without bicarbonate, only the ligand could be observed (Figures S36 and S37). This again confirms that the anion plays a role in the activation of the benzimidazolium to form the carbene, while the sieves function to drive the equilibrium toward the carbene but cannot do so effectively without HCO_3_^–^ anions in solution. The SERS spectra under these conditions support our claim of the synergy between the sieves and the TEAB. Without one or both of these additives, poor-quality spectra were obtained (Figure S17). Analogous results were also obtained for the other model substrate **2a**. In this case, the isotopic pattern for bromine acted as an excellent spectrometric handle, clearly showing the L_2_Au^+^ mass adduct when TEAB was added and only the mass of the free ligand when this additive was excluded (Figures S38–S41). Interestingly, under optimized conditions without molecular sieves, good-quality monolayers were still obtained for this derivative as measured by SERS but significantly weaker than with the addition of 5 Å MS (Figure S18).

The grafting procedure was studied for bromide derivative (**2**) *via* XPS, to allow for comparison to the previous conditions (Section S8 and Figure S46). Monolayers formed from the optimized methodology for **5a**, without either TEAB or 5 Å MS, and excluding both derivatives were studied. In terms of the N_(∼400 eV)_/Au ratio, the optimized conditions in tetrahydrofuran give significantly higher ratios than the alcoholic conditions and similar to the deposition in acetonitrile. This is interesting as it seems to indicate that polar aprotic solvents yield higher surface coverage. When no TEAB was introduced, the amount of chemisorbed carbene as determined by the N_(∼400 eV)_/Au ratio is significantly lower, consistent with the necessity of the hydrogen carbonate to chemically activate the carbene in solution and in agreement with the LDI experiments. These conditions also had a high amount of physiosorbed benzimidazolium as indicated by the peak at ∼401 eV.^45^ The utilization of TEAB did lead to impurities at 402 and 399 eV to be observed, consistent with quaternary ammoniums as reported by Crudden.^45^ Nevertheless, these impurities did not affect the SERS spectra. However, we still report no real correlation between the SERS intensities observed and surface coverage. The optimized conditions for **2a** result in a N_(∼400 eV)_/Au ratio essentially identical to the conditions without the sieves but give a SERS signal *ca.* 3 times higher (Figure S18). Therefore, these results again support the hypothesis that the increase in intensity relates to a monolayer with a more long-range order rather than an increase in the amount of carbene deposited on the surface, *per se*.

We also compared the optimized methodology to the electrochemical deposition,^26^ which has been shown to have significantly higher surface coverage than other reported methods.^23^ Due to the small size of the chips utilized in the SERS experiments, only one chip was successfully functionalized (Figures S23–S25). The SERS spectra for **2a** generated using the electrochemical deposition is identical to the one obtained using the optimized condition, however, is significantly higher in intensity (Figure S24). By XPS (Section S8), the N_(∼400 eV)_/Au ratio was only marginally higher (*ca.* 10%). We believe that the lack of any traces of iodide on the surface as examined by XPS is a possible explanation for the slightly higher coverage.^26^ Since the electrochemical deposition generates some of the highest gold surface coverage reported to date^23,26^ and is comparable to the methodology reported in this manuscript, we believe our methodology is a competitive alternative, offering higher throughput functionalization and not requiring specialist electrochemical equipment.

### Further Anion and Heterocyclic Framework Scope

Surprisingly, the iodide derivatives **1–7b** could also be employed to form monolayers using the optimized conditions but with an overall lower Raman scattering intensity (Figure S28). However, the methyl ester derivative **5b** did not form monolayers as determined by SERS. In terms of XPS, when **2b** was utilized, lower surface coverage was achieved as determined by the N_(∼400 eV)_/Au ratio, and a significantly larger deviation was observed (Section S8 and Figure S46). This is consistent with the iodide anion competing with the carbene for binding to the surface. In fact, the I 3d signal intensity for this derivative was by far the highest. Although iodine was also detected for surfaces functionalized with **2a**, we believe this to be due to impurities. Other reports also reveal an inverse relationship between halide binding to the surface and carbene coverage.^26^ Nevertheless, the AuL_2_^+^ species was detected by LDI-MS when **2b** was utilized as the carbene precursor, indicating chemisorbed monolayers are able to be formed directly from the iodide precursors if hydrogen carbonate is included (Figure S42).

To further support the hypothesis that iodide is disruptive to monolayer formation due to coordination, we investigated the behavior of **1a**, **b**, and **c** using ^1^H NMR in CDCl_3_ (Figure S45). Upon addition of hydrogen carbonate either as an external source using TEAB (1 equiv) or as the counterion, we would expect the C2-proton to decrease in intensity due to fast exchange with traces of HOD in the sample. If the iodide was to disrupt this process, the intensity should remain unchanged. However, for all samples that had HCO_3_^–^ present regardless of the source and initial anion, a decrease in the intensity of the C2-proton was observed. This further confirms that the iodide is not disruptive to the equilibrium and therefore to the generation of the carbene. **1b** was still able to form monolayers without the inclusion of sieves when TEAB was added, consistent with what we previously observed with **1c** (*vide supra* and Section S3 and Figure S21). Utilization of **1b** with bases other than TEAB, namely, TEA and *n*-tetrabutylammonium hydroxide with and without sieves, resulted in poor-quality monolayers with scattering that does not resemble what was previously reported^25,27^ or observed in this study, highlighting that it is not a simple deprotonation mechanism at play but the generation of the free carbene *via* its equilibrium with the hydrogen carbonate anion and the benzimidazolium precursor (Figure S21).

The ability of 5 Å MS to absorb CO_2_ decreases with increasing temperature.^51,58^ To study the influence of this factor on the optimized procedure, we carried out the functionalization of **2a** at room temperature. Well-defined monolayers were obtained as visualized by SERS but with a lower overall intensity compared to when the deposition was carried out at 55 °C (Figure S22). Since 5 Å MS will still retain the ability to sequester the gaseous side product at the latter temperature (especially when found in high concentrations such as the functionalization conditions),^51,58^ the benefit of annealing the monolayer formed^6,24^ must outweigh the decreased absorption of CO_2_.

This therefore allows significant conclusions about the mechanism of monolayer formation to be drawn:

1. Upon inclusion of bicarbonate in solution with a benzimidazolium salt, the two interact *via* hydrogen bonding to polarize the acidic proton and initiate the equilibrium between these two species, the free carbene, and the 2-carboxy species.
2. Disruption of the equilibrium in favor of the formation of the free carbene *via* CO_2_ sequestration is essential for the formation of well-organized and high-scattering monolayers, as presented in Figure 2a.
3. All coordinating and non-coordinating anions are unable to provide free carbenes in solution and therefore do not provide direct routes to NHC-SAMs, unless a bicarbonate source is added.
4. Exclusion of iodide, and possibly other halides, from the gold surface is important in the formation of well-ordered chemisorbed monolayers, due to the ability of the former to coordinate to gold and other precious metal surfaces^6,26^ but not as important as the formation of the free carbene. Complete exclusion of halide anions from the surface using alkylating agents with alternative leaving groups has a significant advantage in the formation of NHC-SAMs.

To test the versatility of our approach, a number of orthogonal substrates were tested (Figure 4b). Compound **9**, as either the PF_6_^–^ or the 2-carboxy derivatives, and compound **10**, as either the acetate or trifluoroacetate salt, demonstrated the scope of anions tolerated by this methodology. The failure of **11h**, SIMes BF_4_^–^ was attributed to the sterically bulky wingtips which might prove challenging to incorporate using the conditions developed, rather than any complications arising from the anion (Figure S26). Additionally, efforts to deposit any of the benzimidazoliums on SERS-active Ag(111) utilizing the methodology developed have failed. As discussed by Amit et al., NHC deposition on Ag is difficult due to competitive surface oxidation, which acts as a passivating layer that could hinder the process.^26^

We also tested our methodology against heterocyclic frameworks other than (benz)­imidazoliums. Recently, Crudden has shown that mesoionic carbenes derived from imidazopyridiniums or triazoliums may have interesting properties due to the superior σ-donating capabilities.^59,60^ The latter are also attractive due to the straightforward and modular synthesis employing copper-click chemistry.^60,61^ Hence, **12i** and **13a** were synthesized, and their monolayer formation was investigated using SERS.

These derivatives require longer and more forcing conditions for monolayer formation compared to their benzimidazolium counterparts.^59^ This was confirmed by weakly scattering monolayers obtained when the optimized conditions were utilized for 2 h. Extending the incubation time to 24 h, as employed by Crudden, significantly improved monolayer formation. Another advantage of the methodology developed herein is the possibility to tune the amount of bicarbonate present in the mixture. When 50 equiv of TEAB were utilized, the intensity of the monolayers after 2 h of incubation was comparable to what was obtained after 24 h with 5 equiv. Interestingly, after three additional hours, the intensity further increased for **12i** (Figures S29 and S30). The chemisorbed nature of the monolayer was confirmed for both derivatives by the presence of AuL_2_^+^ in the LDI spectra (Figures S43 and S44).

## Conclusion

We described in our study a systematic screening of factors to optimize the formation of NHC-SAMs *via* the solution deposition of benzimidazoliums salts. This work has substantially improved the mechanistic understanding behind the process, revealing a fine balance and an intricate relationship between the solvent, anion, substituents on the benzimidazolium salt, and additives that govern the efficiency of NHC-SAMs formation.

The optimized conditions are more operationally efficient, requiring minimal syntheses from commercially available, low-cost starting materials. Together with the high versatility in terms of heterocyclic substrate, wingtip, substituent, and anion, the methodology will open opportunities for new applications in the field of NHC-modified surfaces. Additionally, the kinetics of monolayer formation can be tuned by varying the amount of bicarbonate in the mixture, as made evident by the triazolium derivatives tested. This study highlights the advantages of utilizing vibrational spectroscopy-based methods, namely, SERS, for the optimization of surface-chemistry methodologies compared to other traditionally employed techniques.^22^

## Supplementary Material



## Notes

General methods, full experimental details and characterization data for all novel compounds, additional SERS spectra, and LDI-MS and XPS data (PDF)
