# Supplementary material for: Sieving for Gold—An Efficient Method for Generating N‑Heterocyclic Carbene Self-Assembled Monolayers on Nanostructured Gold Surfaces
Source: J Am Chem Soc. 2025 Nov 18;147(48):44418–29. doi: 10.1021/jacs.5c15471 (PMC12679632; doi:10.1021/jacs.5c15471)
Supplement: Supplementary file 1 [file ja5c15471_si_001.pdf]

SUPPORTING INFORMATION

Sieving for Gold – An Efficient Method for Generating *N*-Heterocyclic Carbene Self-Assembled Monolayers on Nanostructured Gold Surfaces

Matteo Albino,[1,2] Dimitar Georgiev,[1,2,3] Ines Silva,[1,2] Thomas F. F. Fernandez Debets,[2] Mengwei Liu,[2] Tristan N. Dell,[2,4] Jonathan P. Wojciechowski,[2*] and Molly M. Stevens.[1,2*]

[1] *Department of Materials, Department of Bioengineering, and Institute of Biomedical Engineering, Imperial College London, London, SW7 2AZ, United Kingdom*
[2] *Department of Physiology, Anatomy and Genetics, Department of Engineering Science, Kavli Institute for Nanoscience Discovery, University of Oxford, Sherrington Rd, Oxford, OX1 3QU United Kingdom*
[3] *Department of Computing and UKRI Centre for Doctoral Training in AI for Healthcare, Imperial College London, London SW7 2AZ, UK*
[4] *BioInnovation Institute Foundation, Ole Maaløes Vej 3, 2200 Copenhagen N, Denmark*

Number of Pages: 148; Number of Figures: 129; Number of Tables: 42.

Table of Contents

***S1 General Methods*..2**
***S2 Synthetic Procedures*..5**
***S3 Optimisation of 1 and 2*..16**
***S4 SERS spectra of Monolayers Formed from 1-4c*..20**
***S5 Deposition of Benzimidazoliums Using an External Bicarbonate Source*...23**
***S6 LDI Experiments*..42**
***S7 NMR Studies*..50**
***S8 XPS Analysis*..51**
***S9 DFT Studies*..82**
***S10 NMR Spectra*..84**
***S11 3D Printing and Stirrers*..121**
***S12 DFT Coordinates and Free energies*..123**
***S13 References*..147**

S1 General Methods

Solvents and Reagents: All reagents and solvents were purchased from chemical suppliers including Fisher Scientific, Fluorochem, Merck, Sigma-Aldrich and TCI, and were used without any additional purification. 3 Å and 4 Å molecular sieves were purchased from Sigma-Aldrich, whilst 5 Å were purchased from ThermoFischer Scientific and Fischer Scientific.

Nuclear Magnetic Resonance (NMR): NMR spectra were recorded on a 500 MHz spectrometer equipped with an Oxford Instruments Company magnet, a Bruker Avance III HD console and a room temperature broadband probe, and 600 MHz and 750 MHz spectrometers equipped with an Oxford Instruments Company magnet, a Bruker Avance III HD console and a 5 mm TCI CryoProbe. Chemical shifts in ppm were calibrated from proton chemical shifts of the solvents. ^{1}H spectra were recorded at 500, 600 or 750 MHz, ^{13}C spectra recorded at 126 or 151 MHZ, ^{19}F recorded at 565 MHz. Coupling constants are reported in Hz. All spectra were processed using MestReNova. All spectra were recorded at room temperature.

Mass Spectrometry: Electrospray ionisation (ESI) was performed on a Thermo Exactive High-Resolution Orbitrap FTMS (University of Oxford). Flow injection analysis was performed on an ACQUITY I-Class PLUS UPLC System (Waters, Milford, MA, USA) coupled to an ACQUITY RDa mass spectrometer (Waters, Milford, MA, USA) equipped with an ESI probe, in positive ion mode (University of Oxford) and a Waters LCT Premier TOF mass spectrometer operating in positive electrospray ionisation (ESI) with full MS scan (Imperial College London). Mass to charge ratios (m/z) are reported in Daltons with percentage abundance in parentheses along with the corresponding fragment ion, where known. Where complex isotope patterns were observed, the most abundant ion is reported. High resolution mass spectra (HRMS) are reported with less than 5 ppm error.

Infrared Spectroscopy: Solid state or neat spectra were recorded on a Bruker Alpha II spectrometer. Absorption maxima (v_{max}) are reported in wavenumbers (cm^{-1}) to the nearest whole number. Background was measured with 4 scans and a resolution of 2 cm^{-1}. 64 scans with a resolution of 0.5 cm^{-1} were used for each measurement between 400 and 4000 cm^{-1}.

X-ray Photoelectron Spectroscopy (XPS): For XPS analysis of gold film samples, a Thermo Scientific K-Alpha XPS equipped with a monochromatic Al Kα X-ray source (photon energy = 1486.6 eV) was used. Three different locations on the substrate were probed to acquire high-resolution spectra using a 50 eV pass energy and 0.1 eV step size with ultra-high vacuum conditions. 3 points per chips were acquired. The data was then analysed using KherveFitting. Spectra were processed using a Shirley for *C* 1s and *Au* 4f, or linear for *N* 1s background subtraction, and fitted using a Voigt function.[1]

Laser Desorption/Ionisation – Mass Spectroscopy (LDI–MS): Experiments were conducted using a Shimadzu MALDI-8020 spectrometer equipped with a 200 Hz solid-state laser, 355 nm (Nd:YAG). Gold substrates were immobilized on a custom-made aluminium plate using double-sided tape. All mass spectra were

obtained in positive ion mode with Linear-TOF. The maximum laser power was expressed as a percentage relative to the highest achievable power. Typically, 200 profiles composed of 10 laser shots per spectrum were accumulated with a laser power set between 60 and 100%. The instrument was calibrated using Au clusters according to a method developed by Havel.[2]

Surface Enhanced Raman Spectroscopy (SERS)–substrates: all SERS substrates were purchased from SILMECO ApS and utilised for SERS, LDI–MS and XPS experiments.

SERS-measurements: All spectra were acquired using a custom Raman microspectroscopy platform designed for high-throughput analysis known as 'B-Raman'.[3,4] Continuous-wave excitation at 785 nm was supplied by a single-mode diode laser (BRM-785-0.55-100-0.22-SMA, B&W Tek) and attenuated to 5 mW at the sample plane, as verified with a commercial power meter (PM121D, Thorlabs). The excitation line was spectrally cleaned with a 3 nm FWHM clean-up filter (785 nm MaxLine®, LL01-785-12.5, Semrock). Back-scattered photons were collected in an epi-illumination (backscattering) geometry through a 10×, 0.25 NA objective (N PLAN 10×/0.25, Leica). The excitation light was rejected by an ultrasteep long-pass dichroic (RazorEdge® 785 nm, LP02-785RU-25, Semrock) followed by a 785 nm notch filter (33 nm FWHM, NF785-33, Thorlabs). The spectral data was captured with a commercial high-performance Raman spectrometer (EAGLE Raman-S - Ibsen), which features a deeply cooled Andor's iVac 316 camera. The raw spectra were acquired over the spectral wavenumber range of 142.0 to 3684.8 cm^{-1}. For each condition, unless stated in the figure caption, 5 measurements on 3 separate SERS substrates were recorded with 0.1 s integration time and 3 accumulations.

Data pre-processing: Raw measurements were pre-processed to account for spectral artefacts, such as cosmic spikes, baseline variations and experimental noise. The pre-processing involved:

(i) spectral cropping to the range 400–1800 cm^{-1} or 400–3000 cm^{-1};
(ii) cosmic spikes removal using the modified z-score algorithm presented by Whitaker *et al.*[5]
(iii) denoising using a second-order Whittaker smoother with λ=1,[6]
(iv) baseline correction using a second-order Improved Asymmetrically Reweighted Penalized Least Squares (IARPLS) algorithm with a smoothness factor λ = 10.[7] All preprocessing steps were implemented in Python using a combination of custom in-house scripts and open-source packages, including RamanSPy[8] and pybaselines[9]

Once the data was processed, the 15 measurements were averaged and plotted using custom in-house python code. The standard error of the mean was calculated and added to the plot as the ± range of the mean.

3D–Printing: All 3D printing was done on a Bambu Lab X1-carbon printer, using either RS PRO – POPLYPROPYLENE 1.75 mm filament or Bambu PA6-CF filament. See section S11 for details.

Plasma Cleaner: All SERS-substrates and spinners were cleaned using oxygen plasma before functionalisation using a Leybold CTR 100N electronic Diener machine.

The chamber was first pumped down to 0.2 mbar and then backfilled with medical grade 95% O_2, 5% CO_2 gas mixture up to 0.3 mbar. The plasma was then activated at 400 watts for 30 seconds. Before inserting the spinners and the chips the plasma cleaner chamber was cleaned for 5 minutes using the same procedure.

Scanning Electron Microscopy (SEM): Nanopillar chips were transferred to conductive tape on the top surface of a metal stub. Scanning electron imaging was conducted on a Zeiss Sigma 300 FEG-SEM microscope with 2 kV accelerating voltage, *circa* 10 mm working distance and 30 µM aperture.

Electrochemical deposition: 2a was deposited using an adapted literature procedure from Amit *et al.*[10] Electrochemical experiment was performed with an electrochemical work station (PalmSens 4. The electrochemistry experiment was performed by using a Platinum Wire Auxiliary Electrode (7.5 cm), with gold-plated connector, mounted in CTFE cylinder, as a counter electrode, an Ag/AgCl (3 M NaCl) reference electrode, and the SERS chips as working electrode. Briefly, a 5 mM solution of **2a** salt in MeCN along with 0.1 M of a supporting electrolyte ($TBABF_4$) and 50 mM milliQ water at room temperature was added to the cell. A voltage of −1 V was applied for five minutes. After this step, the SERS chips were rinsed excess water (x3) and MeOH (3) and dried under a stream of dinitrogen.

General procedure 1 for the deposition using hydrogen carbonates salts:
The corresponding benzimidazolium hydrogen carbonate was dissolved in the desired solvent (MeOH, EtOH or acetonitrile) to yield a solution of the specified concentration (1, 5 or 10 mM) in a 28 mL glass vial. Sieves were activated by heating to 200 ºC under vacuum for 20 minutes and added after cooling down to room temperature (if called for by the procedure), followed by the cleaned SERS-substrates immobilised into the spinners, *vedi supra*, and the vial sealed. The mixture was stirred at 120 rpm at the specified temperature for the specified time. The substrates were then rinsed with excess methanol 3 times and dried under a stream of dinitrogen.
NOTE: the sieves are cream-colored in appearance, if over-heated they turn blue and the functionalisation fails.

General procedure 2 for the deposition using all other anions ($OMs^–$, $OTf^–$, $I^–$, $PF_6^–$, 2-carboxy, acetate, trifluoroacetate): the corresponding benzimidazolium or triazolium salt and tetraethylammonium bicarbonate (5 eq. for benzimidazoliums, 2 eq. for **8a**, or 50 eq. for triazoliums) were added to a 28 mL glass vial. The solids were then suspended in THF to yield a 1 mM solution of benzimidazolium or triazolium. 5 Å molecular sieves were activated by heating to 200 ºC under vacuum for 20 minutes, cooled to room temperature, added, immediately followed by the cleaned SERS-substrates immobilised into the spinners, and the vial sealed. The mixture was stirred at 120 rpm at 55 ºC for 2 hours (or 5 hours for triazoliums). The substrates were then rinsed with excess water (3 times) and methanol (3 times), and dried under a stream of dinitrogen. NOTE: the sieves are cream-colored in appearance, if over-heated they turn blue and the functionalisation fails.

Computational details: all calculations were carried out using Gaussian 16,[11] with PBE0 hybrid functional,[12] AUG-cc-pVTZ basis set, and GD3BJ empirical dispersion factor,[13] and the SMD solvent model in the relevant solvents,[14] at 328.15 K. For transition states, the Berny algorithm was utilised.[15] All structures were checked for

lack of imaginary frequency, except for transition states were a single imaginary frequency was found relevant to the bond breaking and forming process.

S2 Synthetic Procedures

SAFETY NOTE: For general procedure A and B we noticed a significant pressure build up during the synthesis of all derivatives, regardless of base, solvent and alkylating agent. We believe the production of CO_2 and propene from the competing elimination reaction could be responsible for this observation. Nevertheless, under reflux conditions we saw a significant decrease in the reaction rate. We therefore encourage caution and the use of blast shields for reactions under pressure before repeating the procedure henceforth presented.

General procedure A, Synthesis of Benzimidazolium methanesulfonates:
The corresponding benzimidazole (*ca*. 3 mmol, 1.00 eq.), $NaHCO_3$ (3.00 eq.) was added to a microwave vial, charged with a stirrer bar. The solids were suspended in acetonitrile in order to make a 1.4 M solution. Isopropyl methanesulfonate (3.00 eq.) was then added and the vial sealed. The mixture was then heated at 95 ºC for 18-24 hours, after which all volatiles were removed *in vacuo*, yielding a paste. The residue was redissolved in 10 mL 9:1 DCM/MeOH (v/v), filtered through a short pad of celite, and the latter washed with further 9:1 DCM/MeOH (3 x 5 mL). The volatiles were once again removed *in vacuo* yielding an oily residue that was triturated and sonicated with ethyl acetate until a solid began to precipitate. The supernatant was then carefully removed and the solid washed with further ethyl acetate twice, and then dried under vacuum to yield the corresponding benzimidazolium methanesulfonate.

General procedure B, Synthesis of Benzimidazolium iodides:
The corresponding benzimidazole (*ca*. 3 mmol, 1.00 eq.), $NaHCO_3$ (3.00 eq.) was added to a microwave vial, charged with a stirrer bar. The solids were suspended in acetonitrile in order to make a 1.4 M solution. 2-iodopropane (6.00 eq.) was then added and the vial sealed. The mixture was then heated at 95 ºC for 18-24 hours. After this period, the volatiles were removed *in vacuo* and the residue redissolved in 10 mL 9:1 DCM/MeOH (v/v), filtered through a short pad of celite, and the latter washed with further 9:1 DCM/MeOH (3 x 5 mL). The volume was then reduced to *ca.* 10% its original volume and diethyl ether 30 mL was added, causing precipitation of the product that was collected by centrifugation. If precipitation was not immediate, the solution was triturated and sonicated in a glass vial. Drying under vacuum overnight yielded the desired benzimidazolium iodide.

General procedure C, Synthesis of Benzimidazolium hydrogen carbonates:
Procedure adapted from Gutheil *et al.*[16] Briefly, Amberlyst-hydroxide resin was charged in a normal chromatographic glass column, and NH_4HCO_3 (100 g L^{-1}) flown through the resin without the aid of pressure, until the pH reached 8. MeOH was then used to rinse the column once, before the corresponding benzimidazolium (*ca.* 0.6 mmol) was dissolved in minimal amount of MeOH before gently loading it onto the column. The eluent was checked for the presence of compound by spotting TLC-plates and visualising them under 254 nm light. The fractions containing the compound were pooled together and dried under a stream of compressed air overnight. These

derivatives are known to be hygroscopic and were not dried under vacuum to avoid decomposition.[17,18]
It is important to note that the pH has been observed to initially drop from 14 to 10-9 as measured by pH paper, and then 8. **Significant degradation was noticed for all derivatives when the pH was not stabilised at 8.**

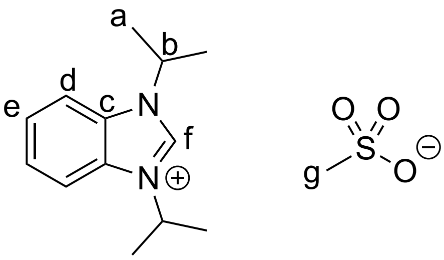

1a, prepared according to general procedure A, as an off–pink crystalline solid, 60%; ^{1}H NMR (500 MHz, Chloroform-*d*) δ 10.63 (s, 1H, H_f), 7.76 (dt, *J* = 6.7, 3.4 Hz, 2H, H_e or H_d), 7.67 – 7.55 (m, 2H, H_e or H_d), 5.12 (hept, *J* = 6.8 Hz, 2H, H_b), 2.80 (s, 3H, H_g), 1.81 (d, *J* = 6.9 Hz, 12H, H_a). ^{13}C NMR (126 MHz, Chloroform-*d*) δ 141.2 (C_f), 131.0 (C_c), 126.8 (C_d or C_e), 114.0 (C_d or C_e), 52.5 (C_b), 39.7 (C_g), 22.0 (C_a). FTIR (ATR) / cm^{-1}: 3532, 3452, 3155, 3107, 3087, 3057, 3031, 2978, 2934, 1556, 1488, 1469, 1439, 1423, 1372, 1340, 1318, 1252, 1185, 1162, 1143, 1112, 1079, 1034, 1018, 984, 847, 778, 757, 620. HRMS (ESI+) m/z: Calculated mass for $C_{13}H_{19}N_2^+$: 203.1543; measured: 203.1539. Characterisation data is consistent with the literature.[19]

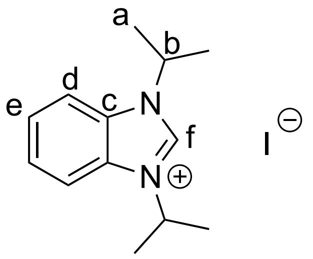

1b, prepared according to general procedure B, as a crystalline white solid, 85%. ^{1}H NMR (500 MHz, Chloroform-*d*) δ 10.94 (s, 1H, H_f), 7.81 (dd, *J* = 6.2, 3.1 Hz, 2H, H_e or H_d), 7.65 (dd, *J* = 6.2, 3.0 Hz, 1H, H_e or H_d), 5.22 (hept, *J* = 6.3 Hz, 2H, H_b), 1.88 (d, *J* = 6.8 Hz, 12H, H_a). ^{13}C NMR (126 MHz, Chloroform-*d*) δ 139.8 (C_f), 131.0 (C_c), 127.2 (C_d or C_e), 114.1 (C_d or C_e), 52.6 (C_b), 22.4 (C_a). FTIR (ATR) / cm^{-1}: 3483, 3421, 3164, 3021, 2984, 2871, 1605, 1555, 1482, 1424, 1379, 1368, 1329, 1311, 1247, 1204, 1140, 1129, 1112, 1080, 1017, 982, 824, 785, 763. HRMS (ESI+) m/z: Calculated mass for $C_{13}H_{19}N_2^+$: 203.1543; measured: 203.1538. Characterisation data is consistent with the literature.[19]

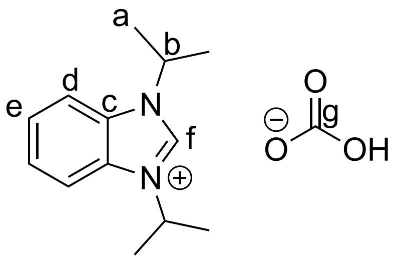

1c, prepared according to general procedure C, as an off–white solid, 90%. ^{1}H NMR (500 MHz, Chloroform-*d*) δ 7.77 – 7.67 (m, 2H, H_e or H_d), 7.62 – 7.52 (m, 2H, H_e or H_d), 5.11 (hept, *J* = 6.9 Hz, 2H, H_b), 1.79 (d, *J* = 6.9 Hz, 12H, H_a). ^{13}C NMR (126 MHz, Chloroform-*d*) δ 160.6 (C_g), 131.1 (C_c), 126.5 (C_d or C_e), 114.0 (C_d or C_e), 52.4 (C_b), 22.0 (C_a). Proton and carbon f are not observed due to fast exchange in consistent with what reported in the literature.[16] FTIR (ATR) / cm^{-1}: 3326, 3155, 3109, 3057, 3031, 2978, 2934, 2763, 1733, 1615, 1555, 1484, 1468, 1436, 1371, 1341, 1319, 1248, 1176, 1162, 1142, 1112, 1033, 1018, 984, 951, 834, 757, 685, 621, 602. HRMS (ESI+) m/z: Calculated mass for $C_{13}H_{19}N_2^+$: 203.1543; measured: 203.1539.

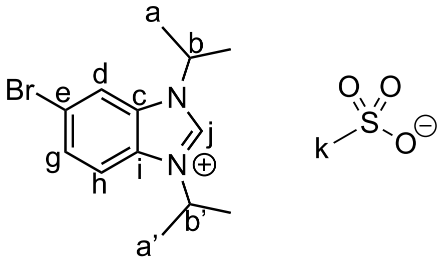

2a, prepared according to general procedure A, as a yellow-to-grey solid, 50%. ^{1}H NMR (500 MHz, Chloroform-*d*) δ 10.72 (s, 1H, H_j), 7.91 (d, *J* = 1.6 Hz, 1H, H_d), 7.72 (dd, *J* = 8.9, 1.7 Hz, 1H, H_g), 7.67 (d, *J* = 8.8 Hz, 1H, H_h), 5.17 – 5.10 (m, 1H, H_b or $H_{b'}$), 5.10 – 5.02 (m, 1H, H_b or $H_{b'}$), 2.79 (s, 3H, H_k), 1.80 (apparent dd, *J* = 6.8, 2.8 Hz, 12H, H_a or $H_{a'}$). ^{13}C NMR (126 MHz, Chloroform-*d*) δ 142.3 (C_j), 132.1 (C_i), 130.3 (C_g), 130.0 (C_c), 120.4 (C_e), 117.0 (C_d), 115.3 (C_h), 53.0 (C_b or $C_{b'}$), 52.9 (C_b or $C_{b'}$), 39.7 (C_k), 22.0 (C_a or $C_{a'}$), 22.0 (C_a or $C_{a'}$). FTIR (ATR) / cm^{-1}: 3494, 3444, 3118, 3047, 2984, 2934, 1646, 1611, 1600, 1552, 1470, 1455, 1433, 1398, 1379, 1324, 1283, 1246, 1214, 1180, 1145, 1115, 1056, 1038, 983, 946, 918, 892, 820, 808, 764, 638. HRMS (ESI+) m/z: Calculated mass for $C_{13}H_{18}BrN_2^+$: 281.0648; measured: 281.0638.

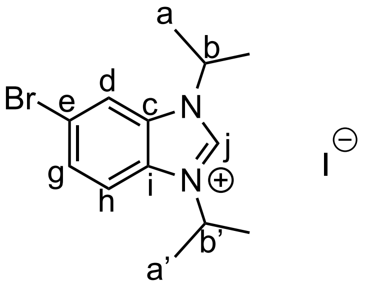

2b, prepared according to general procedure B, as a yellow solid, 85%. ^{1}H NMR (500 MHz, Chloroform-*d*) δ 10.84 (s, 1H H_j), 7.95 (d, *J* = 1.7 Hz, 1H, H_d), 7.79 (d, *J* = 8.9 Hz, 1H, H_h), 7.74 (dd, *J* = 8.9, 1.7 Hz, 1H, H_g), 5.24 (h, *J* = 6.8 Hz, 1H, H_b or

$H_{b'}$), 5.15 (h, *J* = 6.8 Hz, 1H, H_b or $H_{b'}$), 1.85 (apparent dd, *J* = 6.7, 2.5 Hz, 12H, H_a or $H_{a'}$). ^{13}C NMR (126 MHz, Chloroform-*d*) δ 140.3 (C_j), 131.8 (C_i), 130.6 (C_g), 129.9 (C_c), 120.7 (C_e), 116.9 (C_d), 115.6 (C_h), 52.9 (C_b or $C_{b'}$), 52.7 (C_b or $C_{b'}$), 22.3 (C_a or $C_{a'}$), 22.3 (C_a or $C_{a'}$). FTIR (ATR) / cm^{-1}: 3440, 3173, 3024, 3002, 2975, 2929, 2873, 1989, 1915, 1774, 1612, 1599, 1552, 1475, 1422, 1396, 1368, 1317, 1272, 1247, 1236, 1201, 1175, 1146, 1114, 1081, 1049, 982, 882, 845, 826, 813, 789, 763, 741, 641, 621, 603. HRMS (ESI+) m/z: Calculated mass for $C_{13}H_{18}BrN_2^+$: 281.0648; measured: 281.0639.

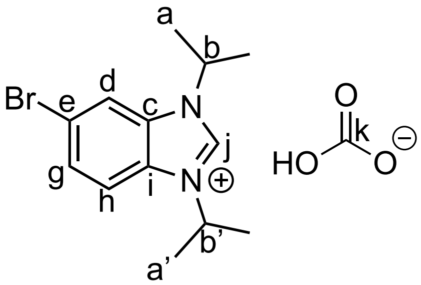

2c, prepared according to general procedure C, as a yellow solid, 78%. ^{1}H NMR (600 MHz, DMSO-d_6) δ 10.12 (s, 1H, H_j), 8.51 (s, 1H, H_d), 8.13 (d, *J* = 8.9 Hz, 1H, H_g), 7.85 (d, *J* = 8.9 Hz, 1H, H_h), 5.20 – 4.91 (m, 2H), 1.89 – 1.33 (m, 12H). ^{13}C NMR (151 MHz, DMSO-d_6) δ 155.8 (C_k), 140.6 (C_j), 131.9 (C_i), 129.9 (C_c), 129.4 (C_g), 119.0 (C_e), 116.8 (C_d), 115.9 (C_h), 51.1 (C_b or $C_{b'}$), 50.9 (C_b or $C_{b'}$), 21.6 (C_a or $C_{a'}$), 21.5 (C_a or $C_{a'}$). FTIR (ATR) / cm^{-1}: 3329, 3032, 2969, 1981, 1735, 1669, 1631, 1595, 1552, 1508, 1459, 1376, 1321, 1286, 1249, 1229, 1174, 1111, 1044, 980, 834, 741, 686, 641. HRMS (ESI+) m/z: Calculated mass for $C_{13}H_{18}BrN_2^+$: 281.0648; measured: 281.0637.

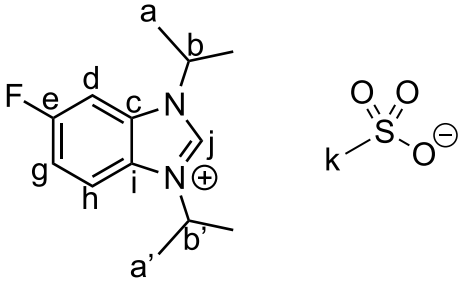

3a, prepared according to general procedure A, as a light brown solid, 54%.^{1}H NMR (500 MHz, Chloroform-*d*) δ 10.70 (s, 1H, H_j), 7.76 (dd, *J* = 9.1, 4.2 Hz, 1H, H_h), 7.46 (dd, *J* = 7.9, 2.4 Hz, 1H, H_d), 7.38 (td, *J* = 8.9, 2.4 Hz, 1H, H_g), 5.09 (apparent dh, *J* = 27.2, 6.8 Hz, 2H, H_b and $H_{b'}$), 2.80 (s, 3H, H_k), 1.93 – 1.71 (m, 12H, H_a and $H_{a'}$). ^{19}F NMR (565 MHz, Chloroform-*d*) δ -110.83 (td, *J* = 8.3, 4.2 Hz$^)$. ^{13}C NMR (126 MHz, Chloroform-*d*) δ 161.1 (d, *J* = 248.9 Hz, C_e), 142.6 (Cj), 131.6 (d, *J* = 12.5 Hz, C_c), 127.5 (C_i), 115.9 (d, *J* = 26.0 Hz, C_g), 115.4 (d, *J* = 10.0 Hz, C_h), 101.0 (d, *J* = 28.1 Hz, C_d), 53.0 (C_b or $C_{b'}$), 52.9 (C_b or $C_{b'}$), 39.8 (C_k), 22.0 (C_a or $C_{a'}$), 21.8 (C_a or $C_{a'}$). FTIR (ATR) / cm^{-1}: 3032, 2976, 1625, 1609, 1552, 1493, 1466, 1436, 1410, 1378, 1312, 1293, 1179, 1137, 1101, 1064, 1037, 990, 919, 857, 828, 767, 691, 640. HRMS (ESI+) m/z: Calculated mass for $C_{13}H_{18}FN_2^+$: 221.1449; measured: 221.1442.

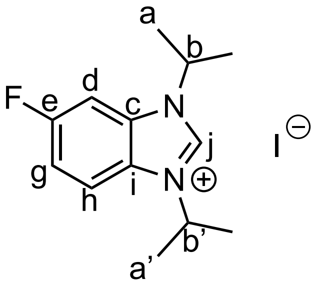

3b, prepared according to general procedure B, as a brown solid, quant. ^{1}H NMR (500 MHz, Chloroform-*d*) δ 10.93 (s, 1H, H_j), 7.84 (dd, *J* = 9.2, 4.2 Hz, 1H, H_h), 7.50 (dd, *J* = 7.8, 2.3 Hz, 1H, H_d), 7.41 (td, *J* = 8.9, 2.3 Hz, 1H, H_g), 5.23 (apparent h, *J* = 6.8 Hz, 1H, H_b or $H_{b'}$), 5.15 (apparent h, *J* = 6.8 Hz, 1H, H_b or $H_{b'}$), 1.87 (apparent dd, *J* = 6.8, 2.7 Hz, 12H, H_a and $H_{a'}$). ^{19}F NMR (565 MHz, Chloroform-*d*) δ -110.20 (t, *J* = 8.7 Hz). ^{13}C NMR (126 MHz, Chloroform-*d*) δ 161.3 (d, *J* = 249.6 Hz, C_e), 140.9 (C_j), 131.52 (d, *J* = 12.5 Hz, C_c), 127.5 (C_i), 116.3 (d, *J* = 26.0 Hz, C_g), 115.7 (d, *J* = 10.0 Hz, C_h), 101.0 (d, *J* = 28.2 Hz, C_d), 53.1 (C_b or $C_{b'}$), 53.0 (C_b or $C_{b'}$), 22.4 (C_a or $C_{a'}$), 22.2 (C_a or $C_{a'}$). 3443, 3120, 3048, 3014, 2994, 2978, 2945, 2928, 2880, 2776, 2175, 2044, 1906, 1786, 1759, 1625, 1611, 1557, 1491, 1465, 1428, 1406, 1391, 1374, 1358, 1338, 1313, 1289, 1244, 1223, 1200, 1161, 1146, 1136, 1123, 1103, 1063, 988, 941, 919, 880, 858, 812, 691, 633. HRMS (ESI+) m/z: Calculated mass for $C_{13}H_{18}FN_2^+$: 221.1449; measured: 221.1442.

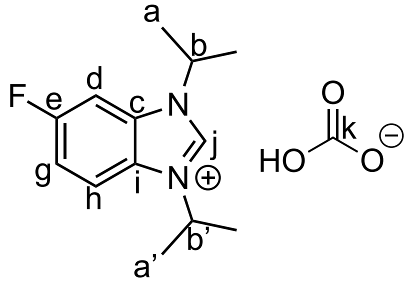

3c, prepared according to general procedure C, as a brown solid, 80%. ^{1}H NMR (600 MHz, DMSO-d_6) δ 10.23 (d, *J* = 9.9 Hz, 1H, H_j), 8.21 (dd, *J* = 9.2, 4.3 Hz, 1H, H_h), 8.17 (d, *J* = 8.9 Hz, 1H, H_d), 7.59 (t, *J* = 9.4 Hz, 1H, H_g), 5.06 (apparent dhept, *J* = 27.2, 7.0 Hz, 2H, H_b or $H_{b'}$), 1.63 (apparent dd, *J* = 6.8, 3.4 Hz, 12H, H_a or $H_{a'}$). ^{19}F NMR (565 MHz, DMSO-*d*6) δ -109.95 – -115.89 (m). ^{13}C NMR (151 MHz, DMSO-d_6) δ 160.4 (d, *J* = 242.6 Hz, C_e), 155.7 (C_k), 141.2 (C_j), 131.3 (d, *J* = 13.5 Hz, C_c), 127.4 (C_i), 115.8 (d, *J* = 10.2 Hz, C_h), 115.0 (d, *J* = 26.0 Hz, C_g), 101.0 (d, *J* = 29.0 Hz, C_d), 51.0 (C_b and C_b'), 21.5 (C_a or $C_{a'}$), 21.4 (C_a or $C_{a'}$). FTIR (ATR) / cm^{-1}: 3457, 3314, 3104, 3027, 2976, 2918, 2772, 1694, 1613, 1550, 1495, 1467, 1438, 1368, 1335, 1290, 1242, 1228, 1202, 1162, 1141, 1127, 1110, 1100, 1075, 1047, 993, 952, 915, 856, 832, 813, 788, 688, 657. HRMS (ESI+) m/z: Calculated mass for $C_{13}H_{18}FN_2^+$: 221.1449; measured: 221.1438.

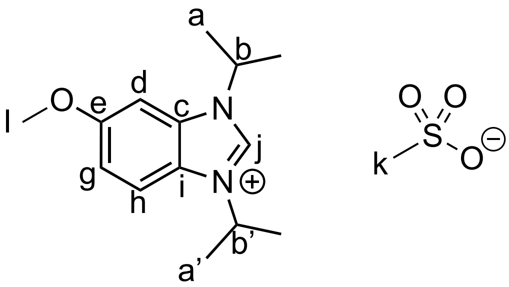

4a, obtained according to general procedure A, as dark oil, 53%. ^{1}H NMR (500 MHz, Chloroform-*d*) δ 10.39 (s, 1H, H_j), 7.62 (d, *J* = 9.2 Hz, 1H, H_h), 7.17 (dd, *J* = 9.1, 2.3 Hz, 1H, H_g), 7.10 (d, *J* = 2.3 Hz, 1H, H_d), 5.11 – 5.02 (m, 1H, H_b or $H_{b'}$), 5.02 – 4.95 (m, 1H, H_b or $H_{b'}$), 3.90 (s, 3H, H_l), 2.76 (s, 3H, H_k), 1.75 (apparent dd, *J* = 6.9, 5.8 Hz, 12H, H_a and $H_{a'}$). ^{13}C NMR (126 MHz, Chloroform-*d*) δ 159.1 (C_e), 139.8 (C_j), 132.2 (C_c), 125.0 (C_i), 116.7(C_g), 114.5 (C_h), 96.1 (C_d), 56.3(C_l), 52.3 (C_b or $C_{b'}$), 51.8 (C_b or $C_{b'}$), 39.6 (C_k), 21.9 (C_a or $C_{a'}$), 21.8 (C_a or $C_{a'}$). FTIR (ATR) / cm^{-1}: 3433, 3116, 3036, 2981, 2937, 2842, 1625, 1553, 1502, 1463, 1435, 1375, 1297, 1241, 1180, 1140, 1111, 1079, 1067, 1037, 986, 912, 856, 823, 765, 697, 644. HRMS (ESI+) m/z: Calculated mass for $C_{14}H_{21}N_2O^+$: 233.1648; measured: 233.1641.

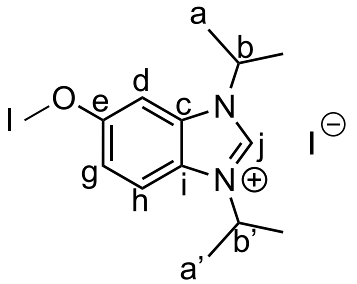

4b, obtained according to general procedure B, as a brown solid, quant. ^{1}H NMR (500 MHz, Chloroform-*d*) δ 10.58 (s, 1H, H_j), 7.68 (d, *J* = 9.1 Hz, 1H, H_h), 7.24 – 7.19 (m, 2H, H_g and H_d), 5.16 (apparent heptd, *J* = 6.8, 1.9 Hz, 2H, H_b and $H_{b'}$), 3.95 (s, 3H, H_l), 1.83 (apparent t, *J* = 6.4 Hz, 12H, H_a and $H_{a'}$). ^{13}C NMR (126 MHz, Chloroform-*d*) δ 159.4 (C_e), 138.5 (C_j), 132.2 (C_c), 125.0 (C_i), 117.3 (C_g), 114.7 (C_h), 96.3 (C_d), 56.7 (C_l), 52.5 (C_b or $C_{b'}$), 52.1 (C_b or $C_{b'}$), 22.4 (C_a or $C_{a'}$), 22.4 (C_a or $C_{a'}$). FTIR (ATR) / cm^{-1}: 3878, 3851, 3799, 3743, 3437, 3111, 3023, 3005, 2970, 2832, 1625, 1548, 1501, 1464, 1430, 1395, 1375, 1333, 1297, 1250, 1238, 1209, 1167, 1132, 1114, 1081, 1016, 986, 914, 858, 820, 742, 697, 638. HRMS (ESI+) m/z: Calculated mass for $C_{14}H_{21}N_2O^+$: 233.1648; measured: 233.1641.

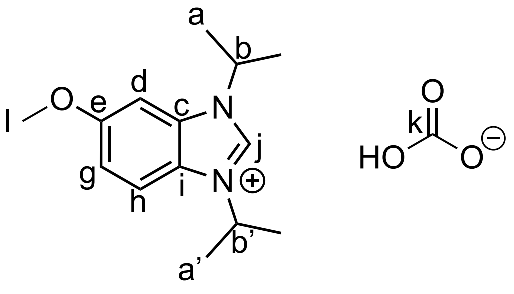

4c, obtained according to general procedure C, as brown solid, . ^{1}H NMR (500 MHz, Methanol-*d*$_4$) δ 7.90 (d, *J* = 9.2 Hz, 1H, H_h), 7.46 (d, *J* = 2.3 Hz, 1H, H_d), 7.31 (dd,

J = 9.2, 2.3 Hz, 1H, H_g), 5.02 (apparent dhept, J = 13.5, 6.7 Hz, 2H, H_b and $H_{b'}$), 3.96 (s, 3H, H_l), 1.72 (d, J = 6.7 Hz, 12H, H_a and $H_{a'}$). ^{13}C NMR (126 MHz, Methanol-d_4) δ 161.5 (C_k), 161.1 (C_e), 133.9 (C_c), 126.7 (C_i), 118.5 (C_g), 115.7 (C_h), 96.8 (C_d), 56.8 (C_l), 52.8 (C_b or $C_{b'}$), 52.3 (C_b or $C_{b'}$), 22.2 (C_a or $C_{a'}$), 22.1 (C_a or $C_{a'}$). Note: H_j and C_j not observed due to fast exchange in protic solvents. FTIR (ATR) / cm^{-1}: 3113, 2980, 2937, 2765, 2658, 1619, 1553, 1501, 1455, 1435, 1367, 1334, 1300, 1243, 1217, 1163, 1141, 1131, 1111, 1098, 1068, 1042, 1024, 983, 914, 858, 834, 814, 684, 642. HRMS (ESI+) m/z: Calculated mass for $C_{14}H_{21}N_2O^+$: 233.1648; measured: 233.1642.

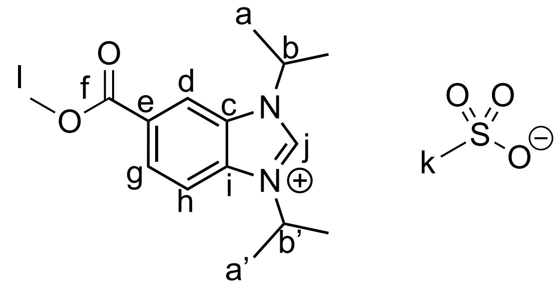

5a, obtained according to general procedure A, as dark oil that solidified upon standing at 4 ºC, 57%. ^{1}H NMR (500 MHz, Chloroform-d) δ 10.62 (s, 1H, H_j), 8.40 (8.40 (d, J = 1.5 Hz, 1H, H_d), 8.24 (dd, J = 8.7, 1.5 Hz, 1H, H_g), 7.86 (d, J = 8.8 Hz, 1H, H_h), 5.20 – 5.05 (m, J = 7.0 Hz, 2H, H_b and $H_{b'}$), 3.94 (s, 3H, H_l), 2.72 (s, 3H, H_k), 1.89 – 1.54 (m, 12H, H_b and $H_{b'}$). ^{13}C NMR (126 MHz, Chloroform-d) δ 165.2 (C_f), 143.1 (C_j), 133.7 (C_c), 130.8 (C_i), 128.9 (C_e), 127.8 (C_g), 115.6 (C_d), 114.0 (C_h), 53.0 (C_b and $C_{b'}$ or C_l), 52.80 (C_b and $C_{b'}$ or C_l), 39.62 (C_k), 21.9 (C_a or $C_{a'}$), 21.8 (C_a or $C_{a'}$). 3577, 3401, 3120, 3038, 2990, 2956, 1712, 1622, 1605, 1546, 1496, 1435, 1396, 1375, 1360, 1329, 1297, 1265, 1248, 1206, 1174, 1149, 1118, 1101, 1037, 992, 967, 903, 853, 843, 795, 767, 752, 646, 630, 604. HRMS (ESI+) m/z: calculated mass for $C_{15}H_{21}N_2O_2^+$: 261.1598; measured: 261.1590.

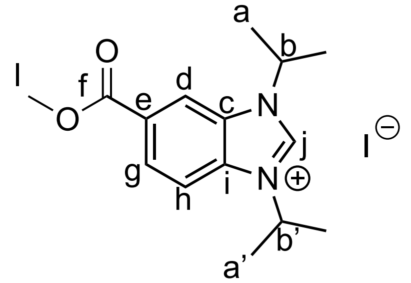

5b, obtained according to general procedure B, as a brown solid, 41%. ^{1}H NMR (500 MHz, Chloroform-d) δ 11.19 (s, 1H, H_j), 8.47 (d, J = 1.6 Hz, 1H, H_d), 8.32 (dd, J = 8.8, 1.5 Hz, 1H, H_g), 7.86 (d, J = 8.8 Hz, 1H, H_h), 5.26 (hept, J = 6.8 Hz, 2H, H_b and $H_{b'}$), 4.02 (s, 3H, H_l), 2.06 – 1.75 (m, 12H, H_a and $H_{a'}$). ^{13}C NMR (126 MHz, Chloroform-d) δ 165.3 (C_f), 141.9 (C_j), 133.7 (C_c), 130.9 (C_i), 129.3 (C_e), 128.1 (C_g), 116.0 (C_d), 114.1 (C_h), 53.2 (C_b, $C_{b'}$ or C_l), 53.2 (C_b, $C_{b'}$ or C_l), 53.2 (C_b, $C_{b'}$ or C_l), 22.4 (C_a or $C_{a'}$), 22.3 (C_a or $C_{a'}$). FTIR (ATR) / cm^{-1}: 3532, 3417, 3109, 2976, 2950, 2763, 1819, 1723, 1623, 1556, 1463, 1432, 1414, 1400, 1388, 1361, 1345, 1318, 1293, 1269, 1243, 1222, 1183, 1147, 1135, 1115, 1085, 992, 961, 889, 844, 794, 764, 743,

628, 603. HRMS (ESI+) m/z: calculated mass for $C_{15}H_{21}N_2O_2^+$: 261.1598; measured: 261.1590. Characterisation data is in agreement with the literature.[18]

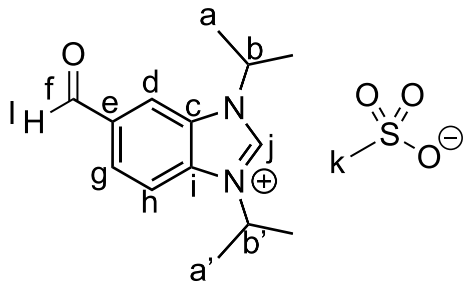

6a, obtained according to general procedure A, as colourless solid, 45%. ^{1}H NMR (500 MHz, Chloroform-*d*) δ 10.94 (s, 1H, H_j), 10.19 (s, 1H, H_l), 8.33 (s, 1H, H_d), 8.15 (d, J = 8.6 Hz, 1H, H_g), 7.95 (d, J = 8.4 Hz, 1H, H_h), 5.32 – 5.07 (m, 2H, H_b and $H_{b'}$), 2.81 (s, 3H, H_k), 1.95 – 1.69 (m, 12H, H_a and $H_{a'}$). ^{13}C NMR (126 MHz, Chloroform-*d*) δ 189.9 (C_f), 144.6 (C_j), 134.7 (C_e or C_c), 134.6 (C_e or C_c), 131.5 (C_i), 127.8 (C_g), 115.7 (C_d), 114.9 (C_h), 53.4 (C_b or $C_{b'}$), 53.4 (C_b or $C_{b'}$), 39.8 (C_k), 22.1 (C_a or $C_{a'}$), 22.0 (C_a or $C_{a'}$). FTIR (ATR) / cm^{-1}: 3852, 3743, 3628, 3432, 3116, 3036, 2982, 2937, 2778, 1685, 1620, 1598, 1555, 1466, 1435, 1396, 1376, 1338, 1315, 1185, 1141, 1104, 1039, 987, 940, 906, 841, 790, 769, 739, 637. HRMS (ESI+) m/z: calculated mass for $C_{15}H_{21}N_2O_2^+$: 231.1492; measured: 231.1486.

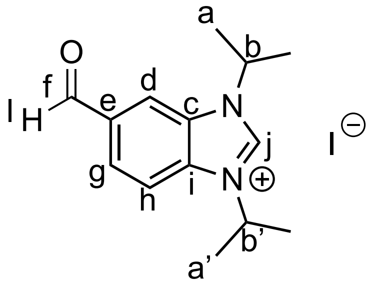

6b, obtained according to general procedure B, as colourless solid, 77%. ^{1}H NMR (750 MHz, Chloroform-*d*) δ 10.92 (s, 1H, H_j), 10.17 (s, 1H, H_l), 8.41 (s, 1H, H_d), 8.13 (d, J = 8.6 Hz, 1H, H_g), 7.97 (d, J = 8.6 Hz, 1H, H_h), 5.29 – 5.22 (m, 1H, H_b or $H_{b'}$), 5.24 – 5.17 (m, 1H, H_b or $H_{b'}$), 1.85 (apparent dd, J = 13.3, 6.8 Hz, 12H, H_a and $H_{a'}$). ^{13}C NMR (126 MHz, Chloroform-*d*) δ 190.2 (C_f), 142.2 (C_j), 135.0 (C_e), 134.5 (C_c), 131.4 (C_i), 127.9 (C_g), 116.4 (C_d), 115.2 (C_h), 53.4 (C_b or $C_{b'}$), 53.3 (C_b or $C_{b'}$), 22.5 (C_a or $C_{a'}$), 22.4 (C_a or $C_{a'}$). FTIR (ATR) / cm^{-1}: 3480, 3419, 3209, 3109, 2979, 2774, 1693, 1622, 1604, 1553, 1435, 1397, 1373, 1340, 1315, 1255, 1227, 1197, 1173, 1146, 1099, 908, 836, 790, 739, 636. HRMS (ESI+) m/z: calculated mass for $C_{15}H_{21}N_2O_2^+$: 231.1492; measured: 231.1486.

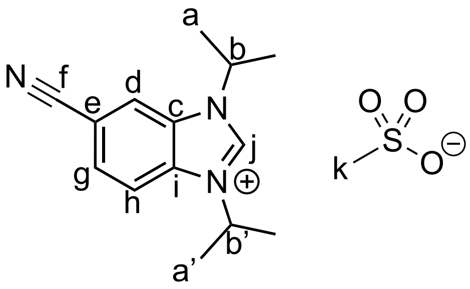

7a, obtained according to general procedure A, as a light brown solid, 39%. ^{1}H NMR (500 MHz, DMSO-d_6) δ 9.98 (s, 1H, H_j), 8.89 (dd, J = 1.5, 0.7 Hz, 1H, H_d), 8.37 (dd, J = 8.8, 0.7 Hz, 1H, H_h), 8.13 (dd, J = 8.7, 1.4 Hz, 1H, H_g), 5.22 – 4.95 (m, 2H, H_b and $H_{b'}$), 2.29 (s, 3H, H_k), 1.65 (apparent dd, J = 6.7, 5.6 Hz, 12H, H_a and $H_{a'}$). ^{13}C NMR (126 MHz, DMSO-d_6) δ 142.2 (C_j), 133.3 (C_c), 130.5 (C_i), 129.5 (C_g), 119.7 (C_d), 118.1 (C_e or C_f), 115.6 (C_h), 108.8 (C_e or C_f), 51.5 (C_b or $C_{b'}$), 51.4 (C_b or $C_{b'}$), 39.8 (C_k), 21.5 (C_a and $C_{a'}$). Note: full assignment was not possible for this structure based on 2D–NMR experiments. FTIR (ATR) / cm^{-1}: 3106, 3037, 2981, 2229, 1678, 1626, 1549, 1495, 1437, 1413, 1393, 1376, 1355, 1316, 1260, 1215, 1182, 1160, 1133, 1102, 1069, 1037, 987, 927, 907, 832, 765, 649, 625, 605. HRMS (ESI+) m/z: calculated mass for $C_{14}H_{18}N_3^+$: 228.1495; measured: 228.1488.

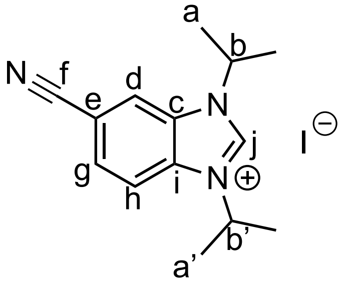

7b, obtained according to general procedure B, as yellow solid, 49%. ^{1}H NMR (500 MHz, DMSO-d_6) δ 10.02 (s, 1H, C_j), 8.89 (d, J = 1.4 Hz, 1H, C_d), 8.38 (d, J = 8.7 Hz, 1H, C_h), 8.12 (dd, J = 8.7, 1.1 Hz, 1H, C_g), 5.21 – 5.01 (m, 2H, H_b and $H_{b'}$), 1.81 – 1.30 (m, 12H, H_a and $H_{a'}$). ^{13}C NMR (126 MHz, DMSO-d_6) δ 142.6 (C_j), 133.7 (C_c), 131.0 (C_i), 130.0 (C_g), 120.2 (C_d), 118.6 (C_e or C_f), 116.1 (C_h), 109.3 (C_e or C_f), 52.1 (C_b or $C_{b'}$), 51.9 (C_b or $C_{b'}$), 22.0 (C_a and $C_{a'}$). FTIR (ATR) / cm^{-1}: 3434, 3112, 2981, 2941, 2878, 2841, 2771, 2230, 1992, 1980, 1962, 1948, 1932, 1918, 1906, 1894, 1874, 1847, 1822, 1685, 1652, 1621, 1598, 1553, 1490, 1462, 1435, 1410, 1396, 1374, 1355, 1337, 1314, 1253, 1186, 1161, 1140, 1100, 1063, 1039, 985, 940, 906, 832, 790, 769, 739, 678, 646, 622, 604. HRMS (ESI+) m/z: calculated mass for $C_{14}H_{18}N_3^+$: 228.1495; measured: 228.1484. Characterisation data is in agreement with the literature.[18]

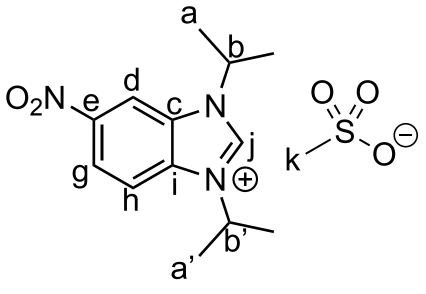

8a, obtained according to general procedure A, as a yellow solid, 60%. ^{1}H NMR (500 MHz, Chloroform-*d*) δ 11.11 (s, 1H, H_j), 8.72 (s, 1H, H_d), 8.54 (d, *J* = 9.2 Hz, 1H, H_g), 7.96 (d, *J* = 9.1 Hz, 1H, H_h), 5.89 – 5.04 (m, 2H, H_b and $H_{b'}$), 2.82 (s, 3H, H_k), 1.90 (dd, *J* = 11.7, 6.8 Hz, 12H, H_a and $H_{a'}$). ^{13}C NMR (126 MHz, Chloroform-*d*) δ 146.2 (C_c), 145.9 (C_j), 134.6 (C_e), 130.8 (C_i), 122.1 (C_g), 115.1 (C_h), 110.7 (C_d), 53.8 (C_b or $C_{b'}$), 53.7 (C_b or $C_{b'}$), 39.8 (C_k), 22.1 (C_a or $C_{a'}$), 22.0 (C_a or $C_{a'}$). FTIR (ATR) / cm^{-1}: 3110, 3034, 2993, 2980, 2931, 2840, 1626, 1613, 1553, 1528, 1437, 1392, 1374, 1347, 1335, 1306, 1286, 1241, 1224, 1211, 1193, 1144, 1131, 1115, 1087, 1071, 1040, 987, 923, 904, 861, 835, 803, 791, 767, 739, 639. HRMS (ESI+) m/z: calculated mass for $C_{13}H_{18}N_3O_2^+$: 248.1394; measured: 248.1387.

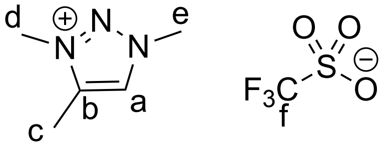

12i, prepared according to an adapted literature procedure.[20] Briefly, 1,4-dimethyl-1*H*-1,2,3-triazole (270 mg, 2.78 mmol) was dissolved in dichloromethane (1.64 mL) and methyl triflate (0.35 ml, 3.06 mmol, 1.10 eq.) was added dropwise to maintain the mixture close to room temperature. After overnight stirring the solvent was removed *in vacuo* to yield a red oil that was triturated in diethyl ether until all starting material was removed, 489 mg, 67%. ^{1}H NMR (500 MHz, Chloroform-*d*) δ 8.51 (s, 1H, H_a), 4.30 (s, 3H, H_e), 4.19 (s, 3H, H_d), 2.56 (s, 3H, H_c). ^{19}F NMR (565 MHz, Chloroform-*d*) δ -78.65, -79.00. Note: We suspect the second peak to arise from some leftover triflic acid. This observation did not noticeably influence the deposition. ^{13}C NMR (126 MHz, Chloroform-*d*) δ 141.0 (C_b), 130.3 (C_a), 120.6 (d, *J* = 319.9 Hz, C_f, two peaks not observed due to *J* value), 40.2 (C_e), 37.4 (C_d), 9.1 (C_c). FTIR (ATR) / cm^{-1}: 3514, 3483, 3125, 1635, 1591, 1453, 1432, 1411, 1387, 1361, 1252, 1223, 1151, 1074, 1027, 840, 801, 757, 634, 600. HRMS (ESI+) m/z: calculated for $C_5H_{10}N_3^+$: 112.0869; measured: 112.0868.

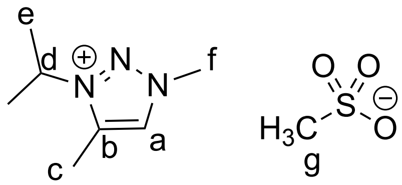

13a: Into a microwave vial, 1,4-dimethyl-1*H*-1,2,3-triazole (300 mg, 3.09 mmol) was dissolved in acetonitrile (1.83 mL) and isopropyl methanesulfonate (0.556 ml, 4.63 mmol, 1.50 eq.) added. The vial was sealed and heated at 95 ºC overnight. The mixture was then cooled down, the volatiles evaporated *in vacuo* and the mixture triturated in ethyl acetate to yield a clear oil, 277 mg, 38%. ^{1}H NMR (500 MHz, Chloroform-*d*) δ 9.03 (s, 1H, H_a), 4.85 (hept, *J* = 6.6 Hz, 1H, H_d), 4.39 (s, 3H, H_f), 2.82 (s, 3H, H_g), 2.59 (s, 3H, H_c), 1.64 (d, *J* = 6.7 Hz, 6H, H_e). Note: the integral of proton g is significantly above 3, most likely due to the leftover methanesulfonic acid (as also evidence by a broad peak at 10.20 ppm). This observation did not noticeably influence the deposition. ^{13}C NMR (126 MHz, Chloroform-*d*) δ 139.4 (C_b), 131.3 (C_a), 54.8 (C_d),

40.5 (C_f), 39.5 (C_g), 22.1 (C_e), 9.1 (C_c). FTIR (ATR) / cm^{-1}: 3499, 3447, 3107, 2991, 2938, 1651, 1586, 1446, 1417, 1376, 1353, 1329, 1236, 1212, 1172, 1150, 1133, 1105, 1070, 1037, 889, 788, 767, 652, 609. HRMS (ESI+) m/z: calculated for $C_7H_{14}N_3^+$: 140.1182; measured: 140.1180.

S3 Optimisation of **1** and **2**

Functionalisation was carried out using the general procedure 1 specified in section S1.

Table S1. Conditions for entries in **Figure S1**.

Entry	Precursor	Concentration / mM	Solvent	Temperature / ºC	Time / h
1	**1a**	1	MeOH	RT	24
2	**1a**	5	EtOH	55	2
3	**1a**	1	EtOH	RT	24
4	**1c**	5	MeOH	RT	24
5	**1c**	1	MeOH	RT	24
6	**1c**	5	EtOH	55	2

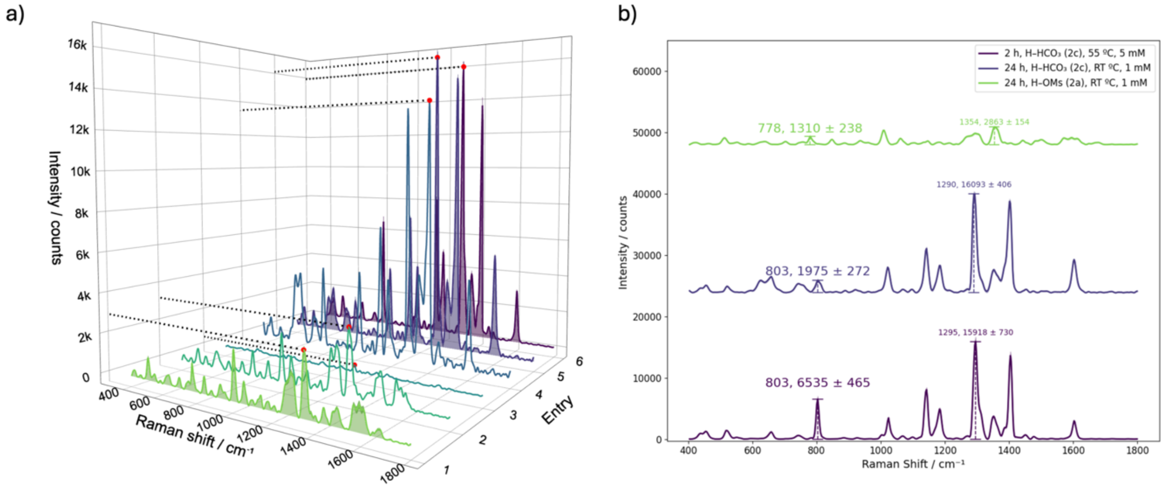

Figure S1. a) Initial screening of conditions for the alcoholic deposition of **1**. b) Stack of Selected entries from a) (from top to bottom: 1, 5 and 6).

Table S2. Deposition time for each entry in **Figure S2**.

Entry	Time / h
1	0.25
2	2
3	6
4	24
5	48
6	168

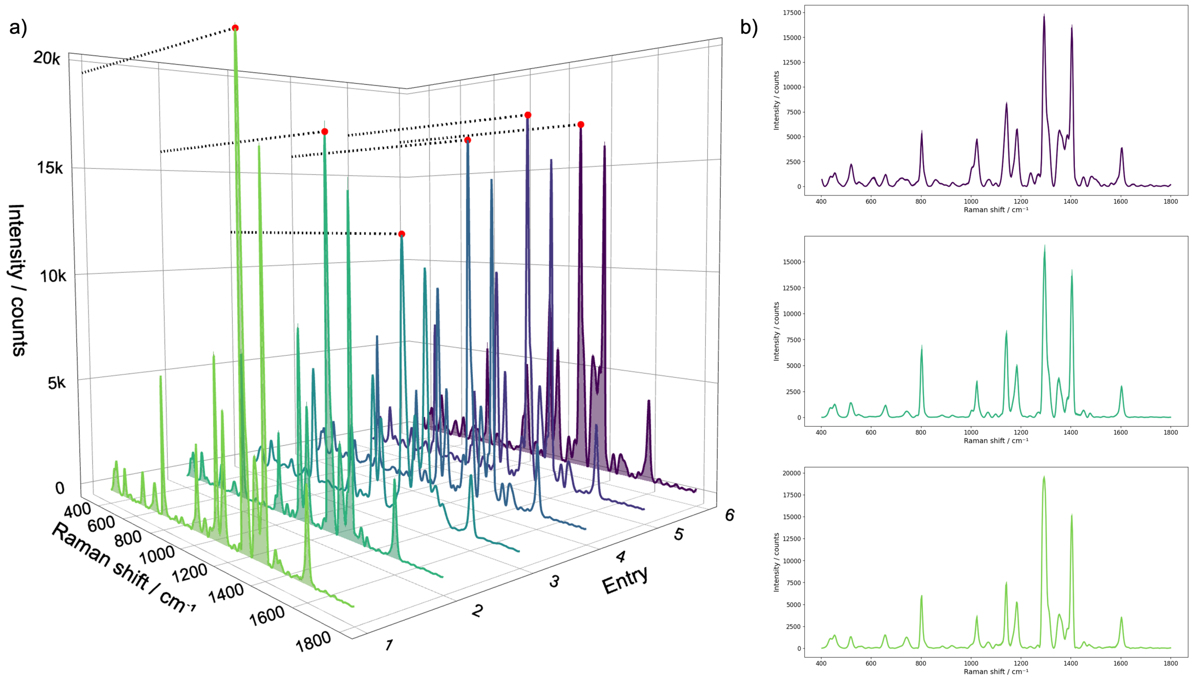

Figure S2. a) SERS spectra of monolayers formed from **1c** as a function of time; experimental conditions from general procedure 1, *vedi supra*. Briefly: 5 mM **1c**, EtOH, 55 ºC. b) Stack of selected entries from a) (from top to bottom: 6, 2 and 1).

Table S3. Deposition conditions for each entry in **Figure S3**.

Entry	Time / h	[2c] / mM	Solvent	Additives
1	**2**	5	EtOH	/
2	**24**	5	EtOH	/
3	**2**	1	EtOH	/
4	**2**	10	EtOH	/
5	**2**	5	MeCN	/
6	**2**	5	EtOH	5 Å MS
7	**2**	5	MeCN	5 Å MS

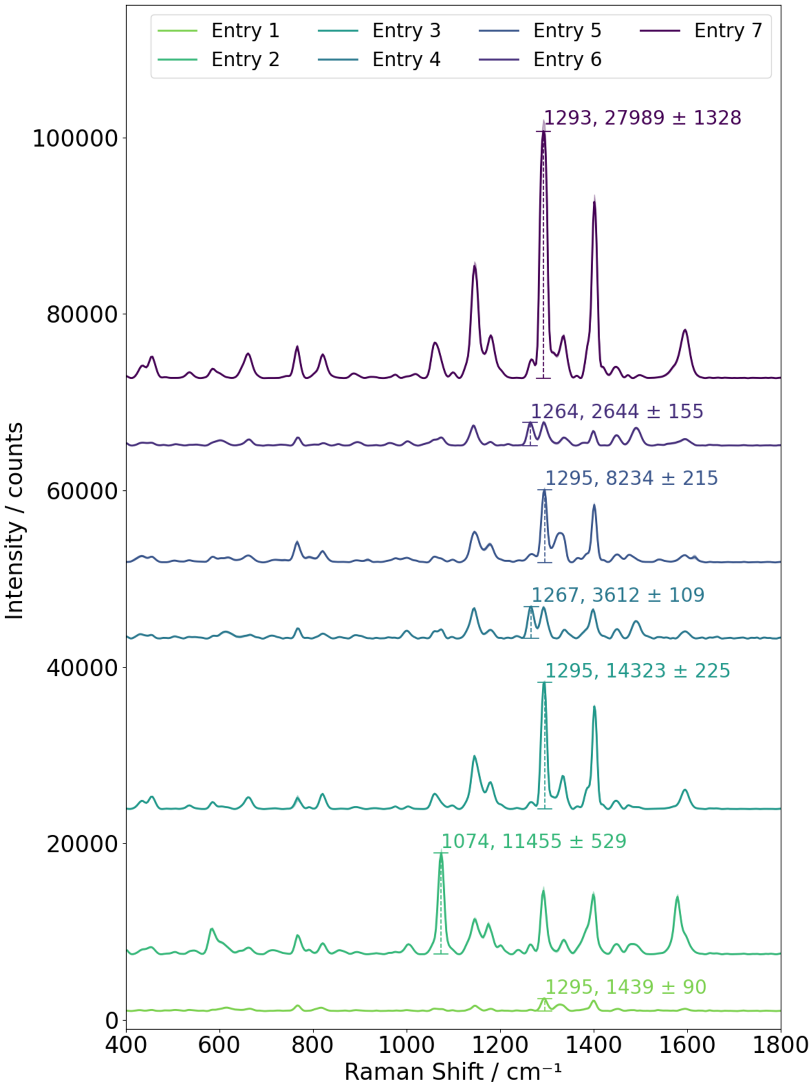

Figure S3. SERS spectra of monolayers formed from **2c** as a function of solvent, concentration and addition of 5 Å MS.

Table S4. Deposition conditions for each entry in **Figure S4**.

Entry	Conditions
1	CO_2 atmosphere, no 5 Å MS
2	No MS, benchtop MeCN
3	3 Å MS
4	4 Å MS
5	Inactivated 5 Å MS
6	Dry MeCN, dry argon atmosphere, oven dried glassware, no MS
7	Optimised conditions

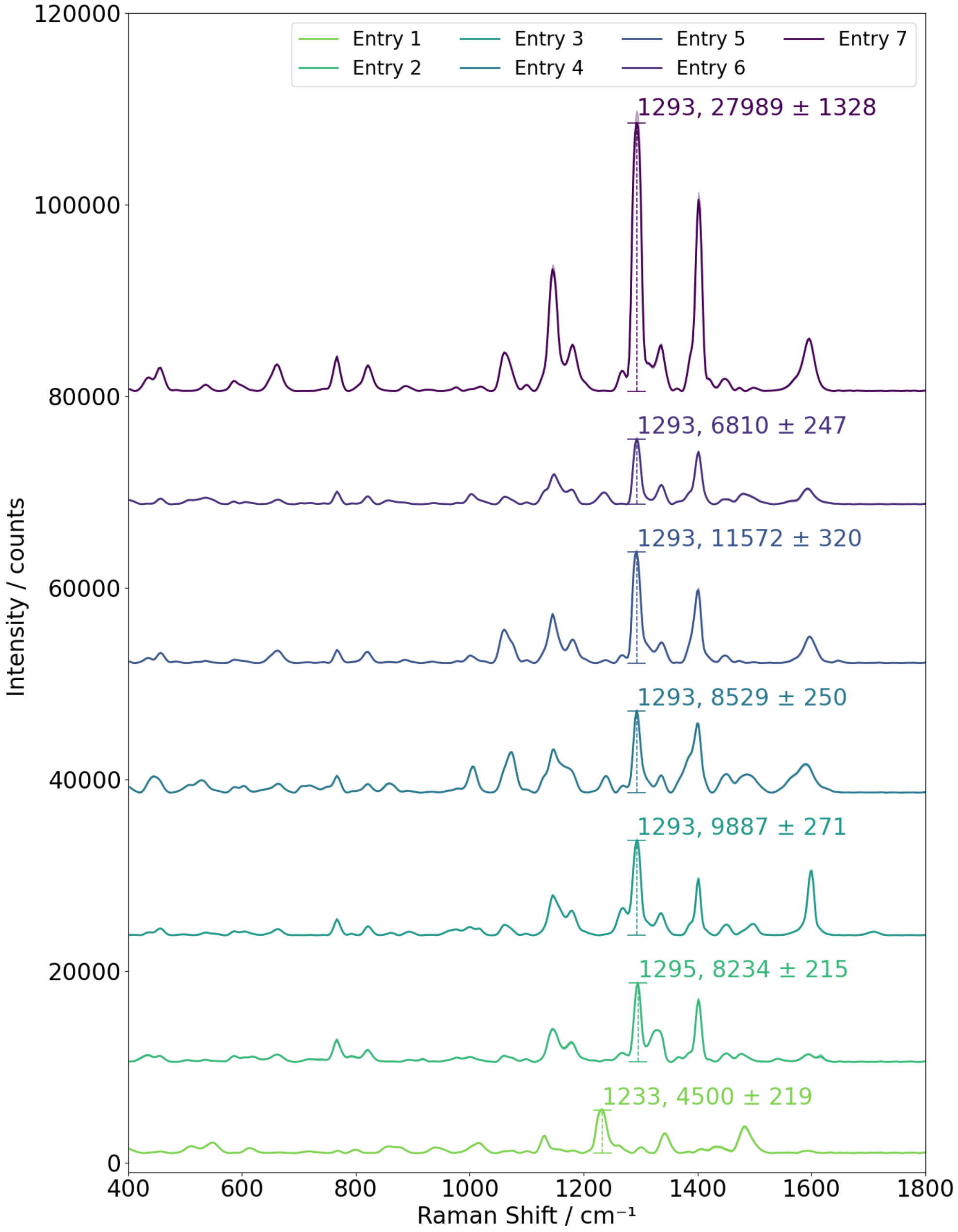

Figure S4. Mechanistic SERS experiments: SERS spectra of monolayers formed from **2c** under modified optimised conditions (5 mM **2c**, benchtop MeCN, 55 ºC, 2 h).

S4 SERS spectra of Monolayers Formed from **1-4c**

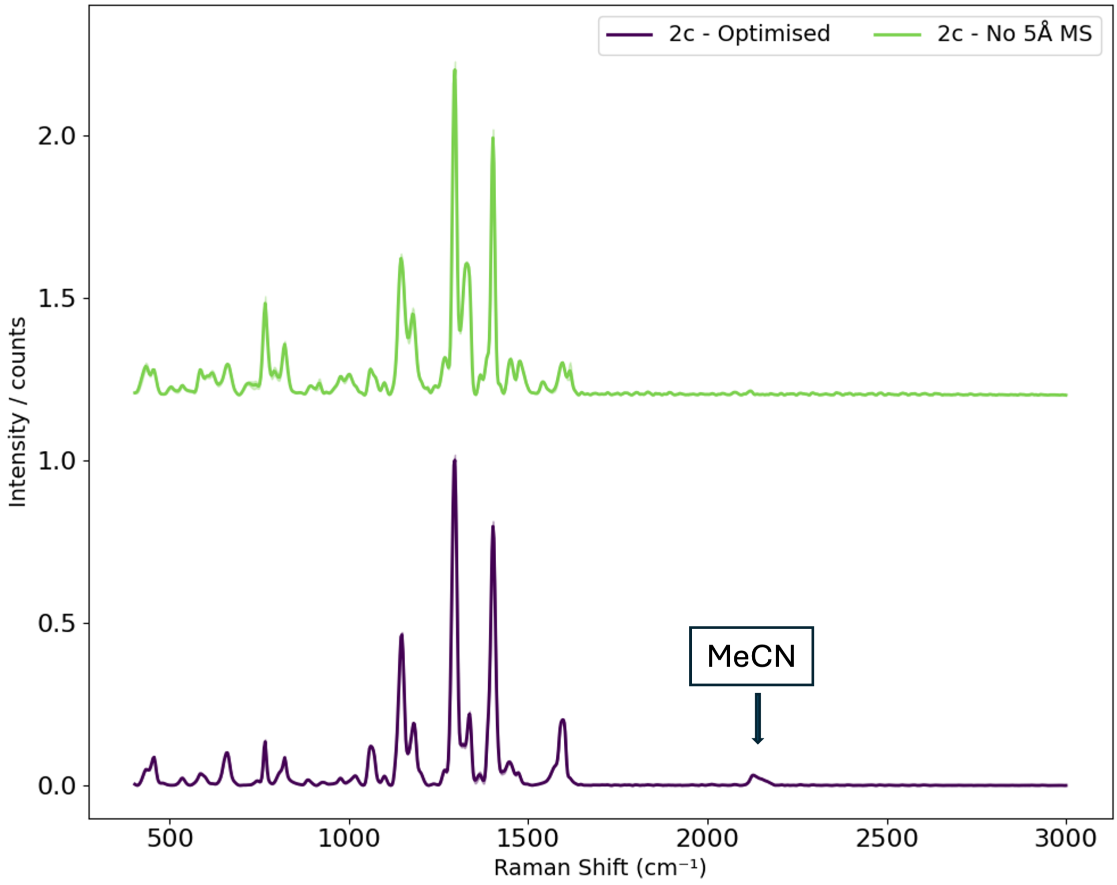

Figure S5. SERS spectrum of monolayers obtained using 5mM **2c** in MeCN, 55 ºC, 2 h with 5 Å MS (bottom) showing the MeCN peak in the nitrile region and without (top) showing no nitrile peak.

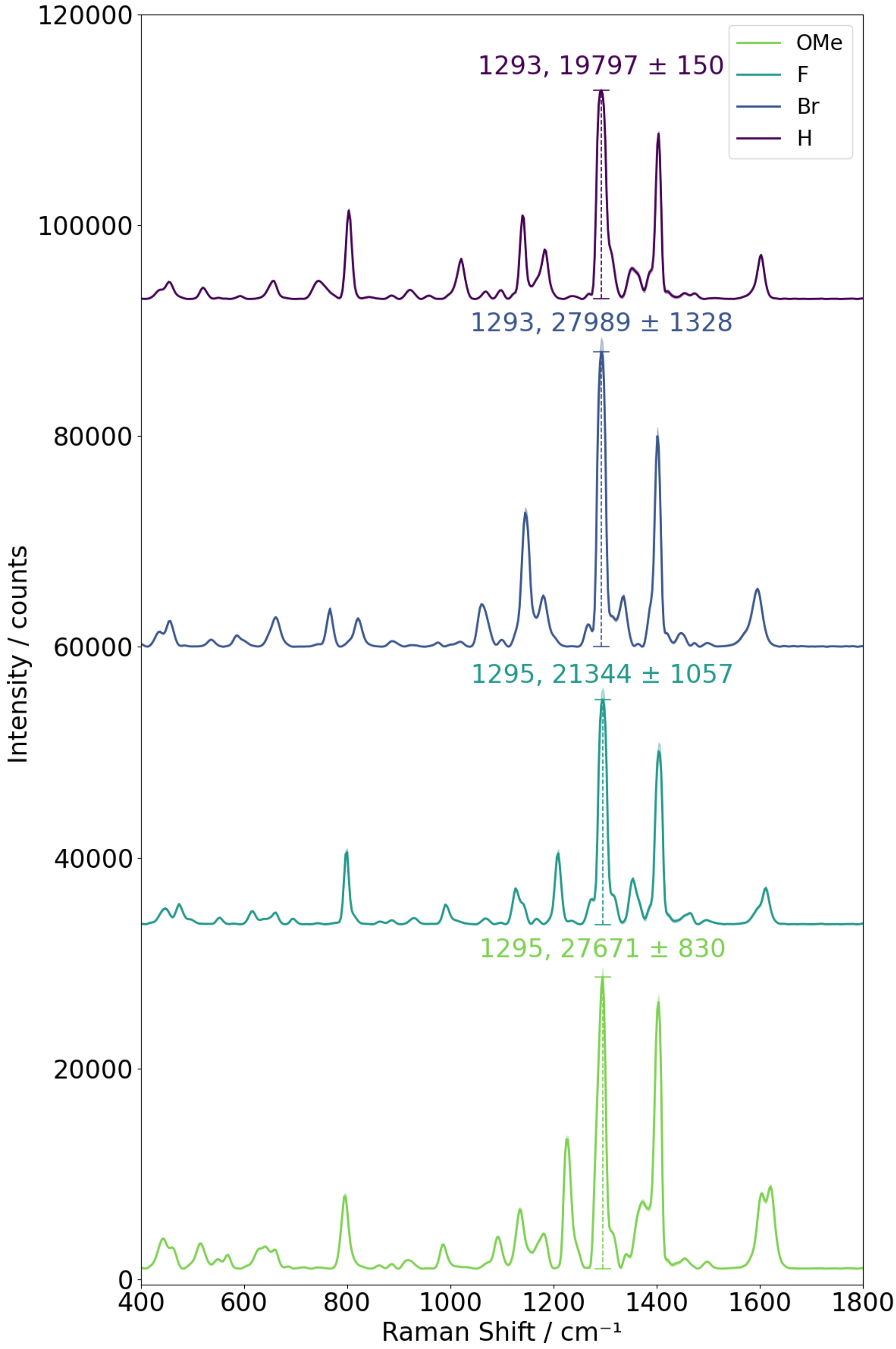

Figure S6. SERS spectra obtained using 5 mM **1-4c** in MeCN, 55 ºC, 2 h with 5 Å MS.

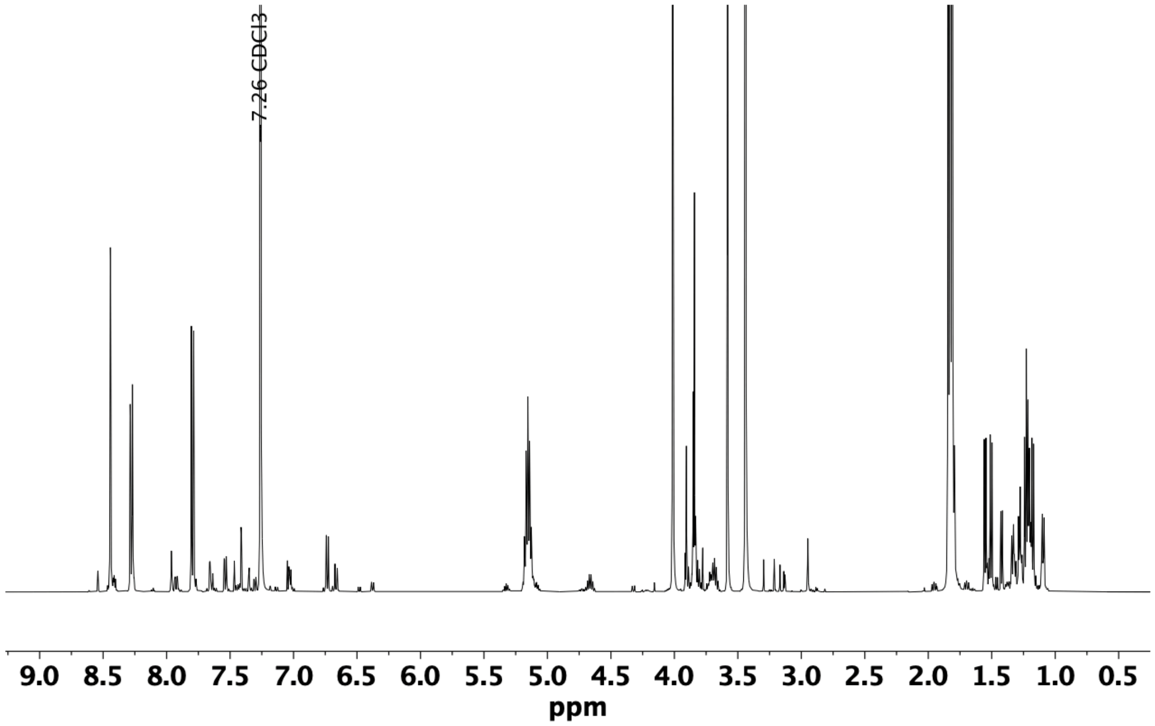

Figure S7. ^{1}H NMR spectrum of **5b** after anion exchange column in $CDCl_3$ showing significant evidence of decomposition.

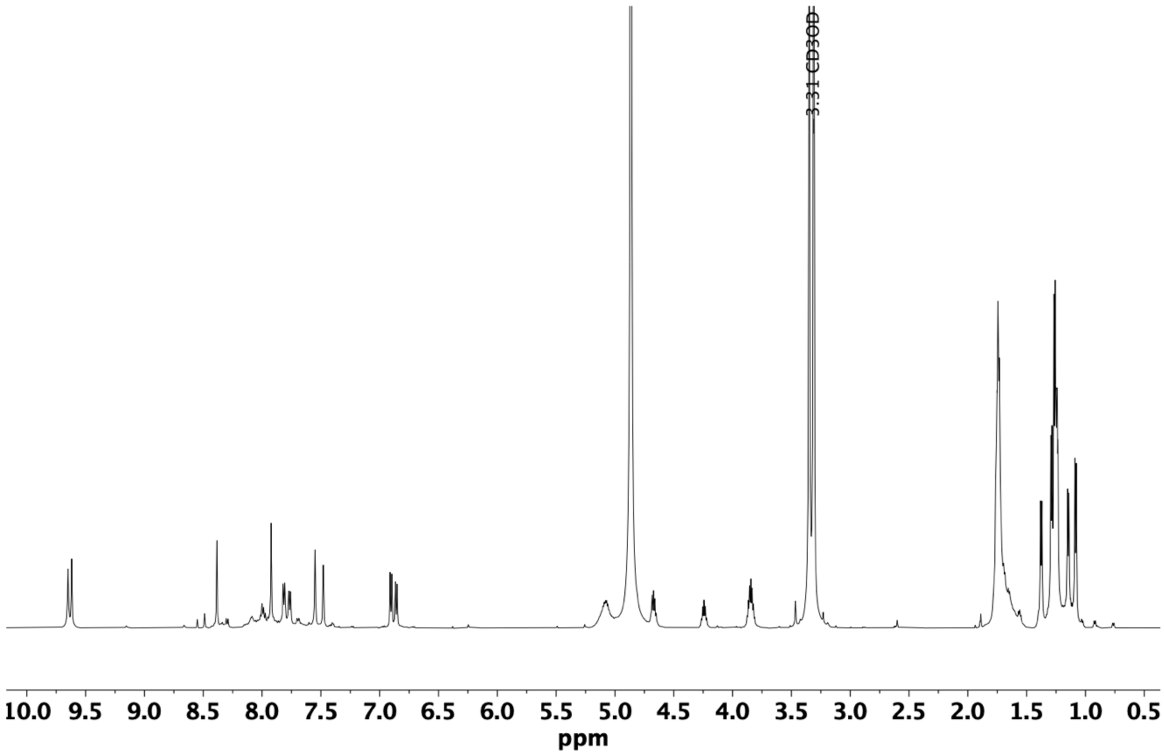

Figure S8. ^{1}H NMR spectrum of **6b** after anion exchange column in MeOD showing significant evidence of decomposition.

S5 Deposition of Benzimidazoliums Using an External Bicarbonate Source

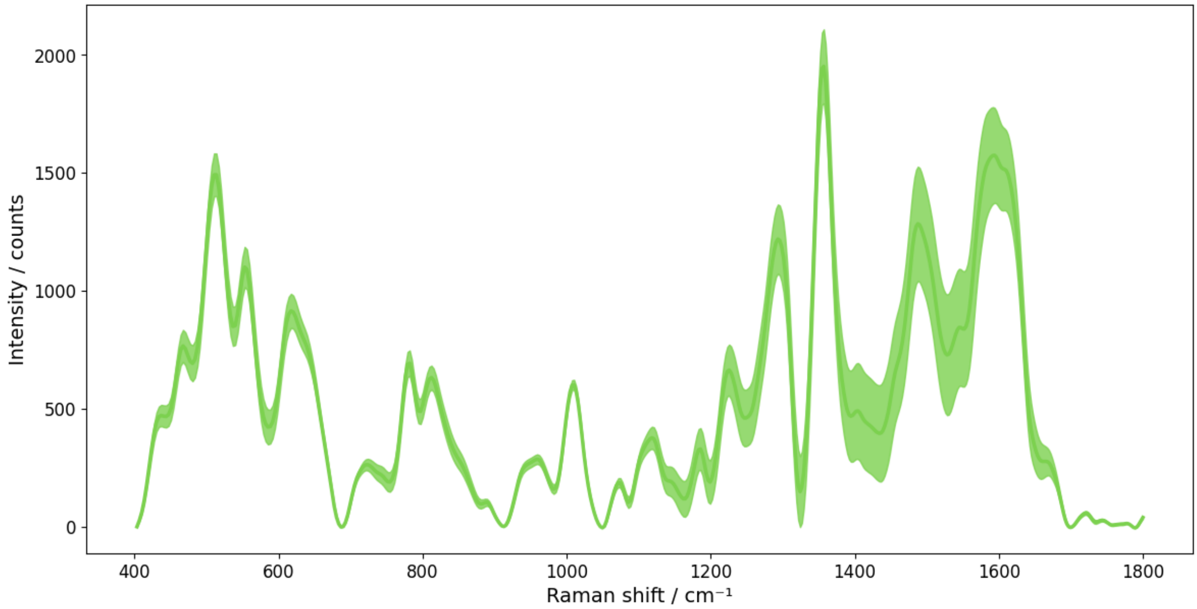

Figure S9. SERS spectrum of monolayers formed using optimised procedure developed for hydrogen carbonates derivatives but using **1a** and NH_4HCO_3 instead of **1c**.

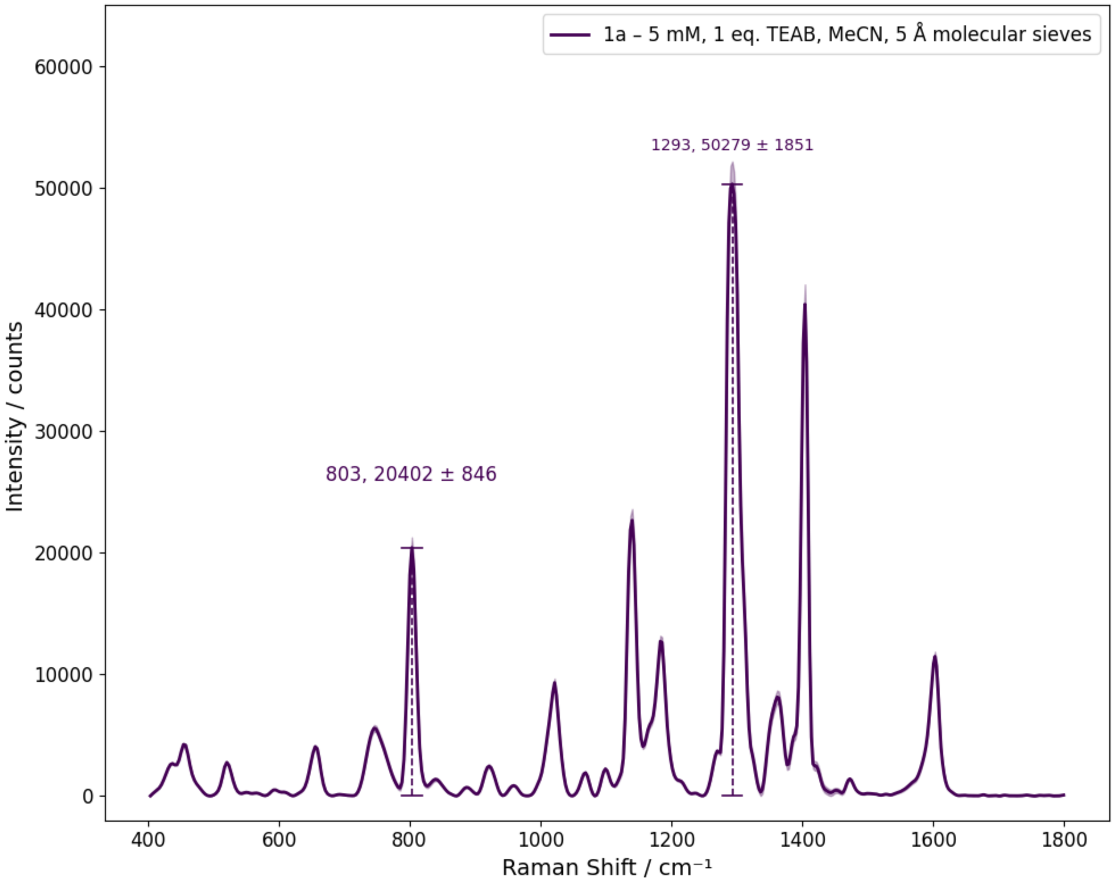

Figure S10. SERS spectrum of monolayers formed using optimised procedure developed for hydrogen carbonates derivatives but using **1a** and TEAB instead of **1c**.

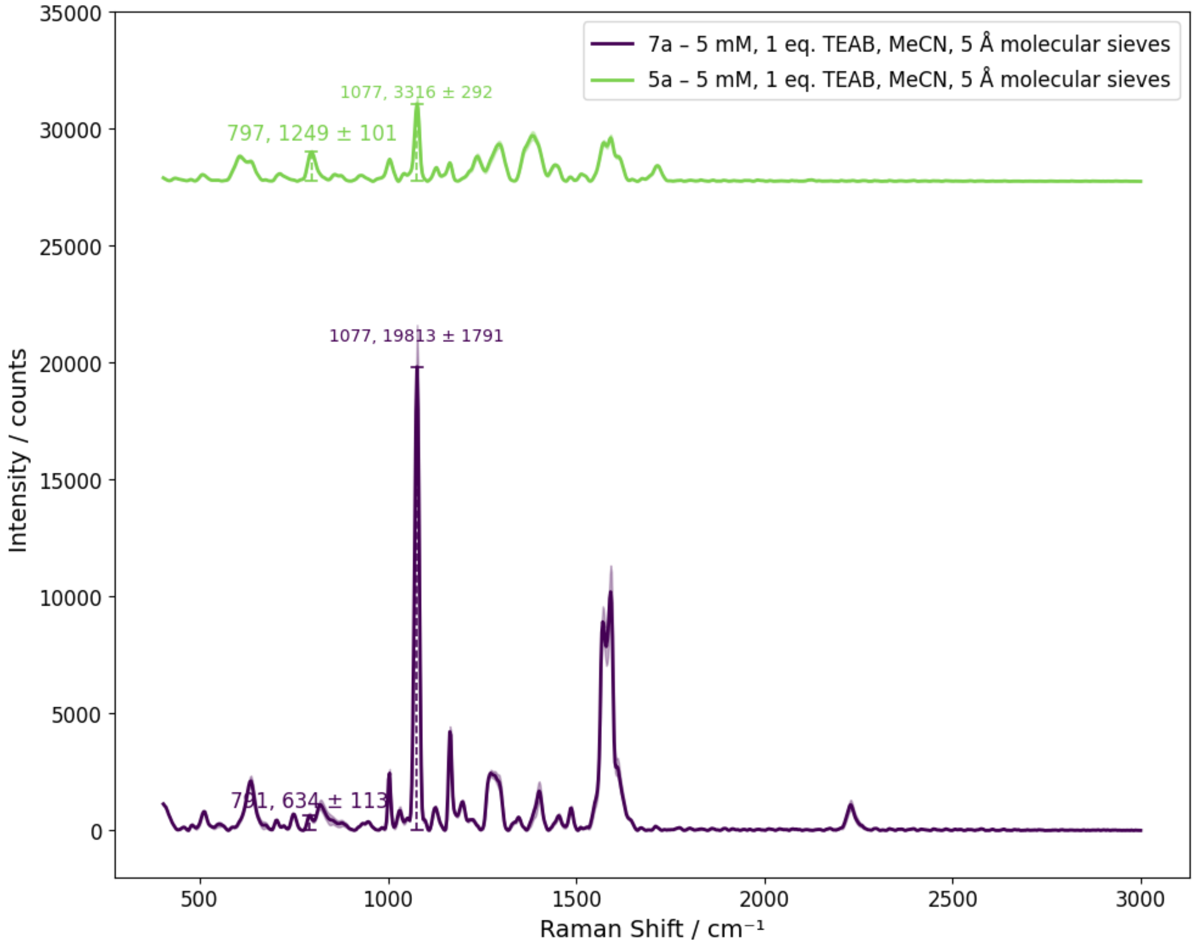

Figure S11. SERS spectra of monolayers formed using optimised procedure developed for hydrogen carbonates derivatives but using **5a** and **7a**, and TEAB.

Table S5. Optimisation entries for **5a** using TEAB in MeCN.

Entry	Concentration / mM	Equivalences of TEAB	Temperature / ºC
1	1	1	55
2	1	10	55
3	5	1	55
4	5	10	55
5	10	1	55
6	10	10	55
7	5	1	70
8	5	10	70

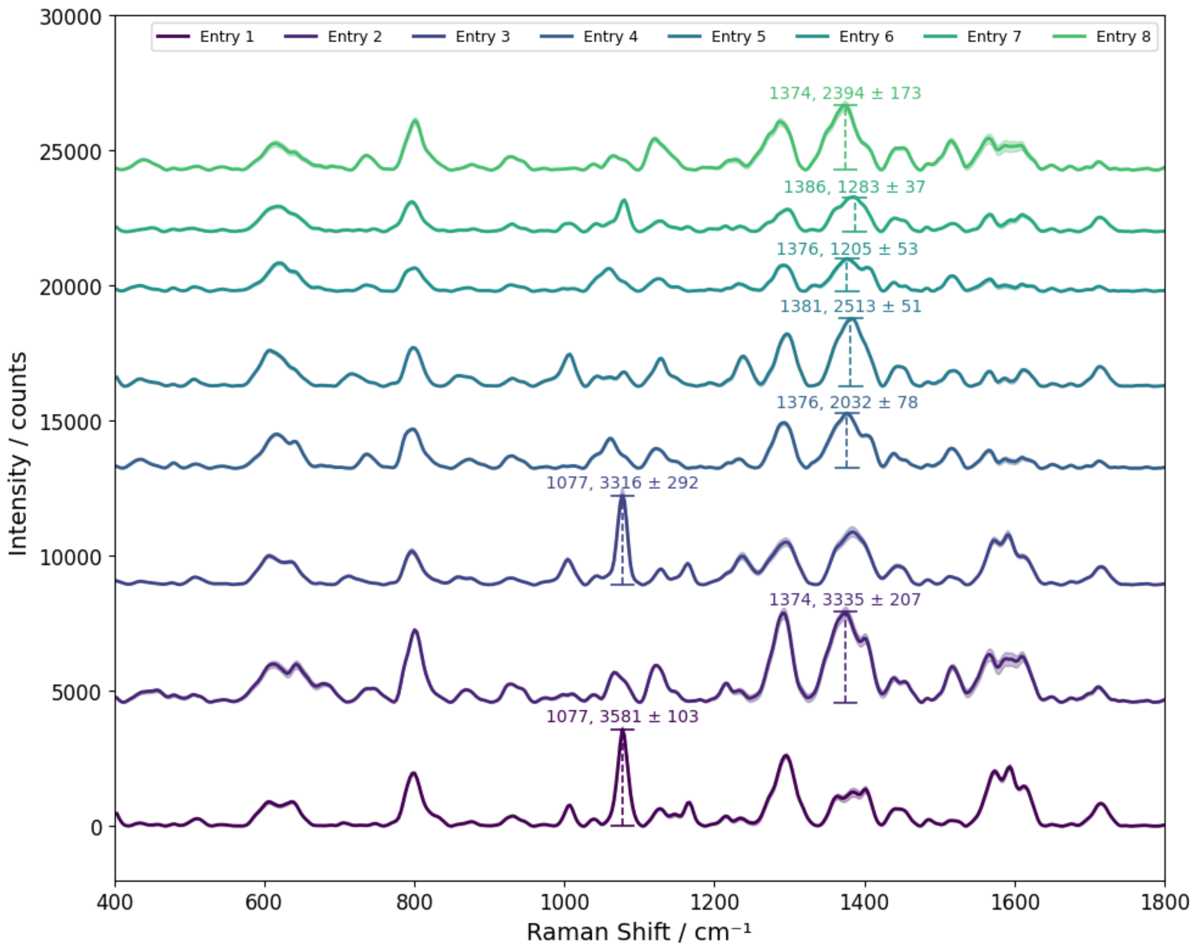

Figure S12. SERS spectra obtained from entries in **Table S3**.

Table S6. Optimisation entries for **5a** using TEAB in ^{i}PrOAc.

Entry	Concentration / mM	Equivalences of TEAB	Temperature / ºC
1	5	1	55
2	5	10	55
3	5	1	70

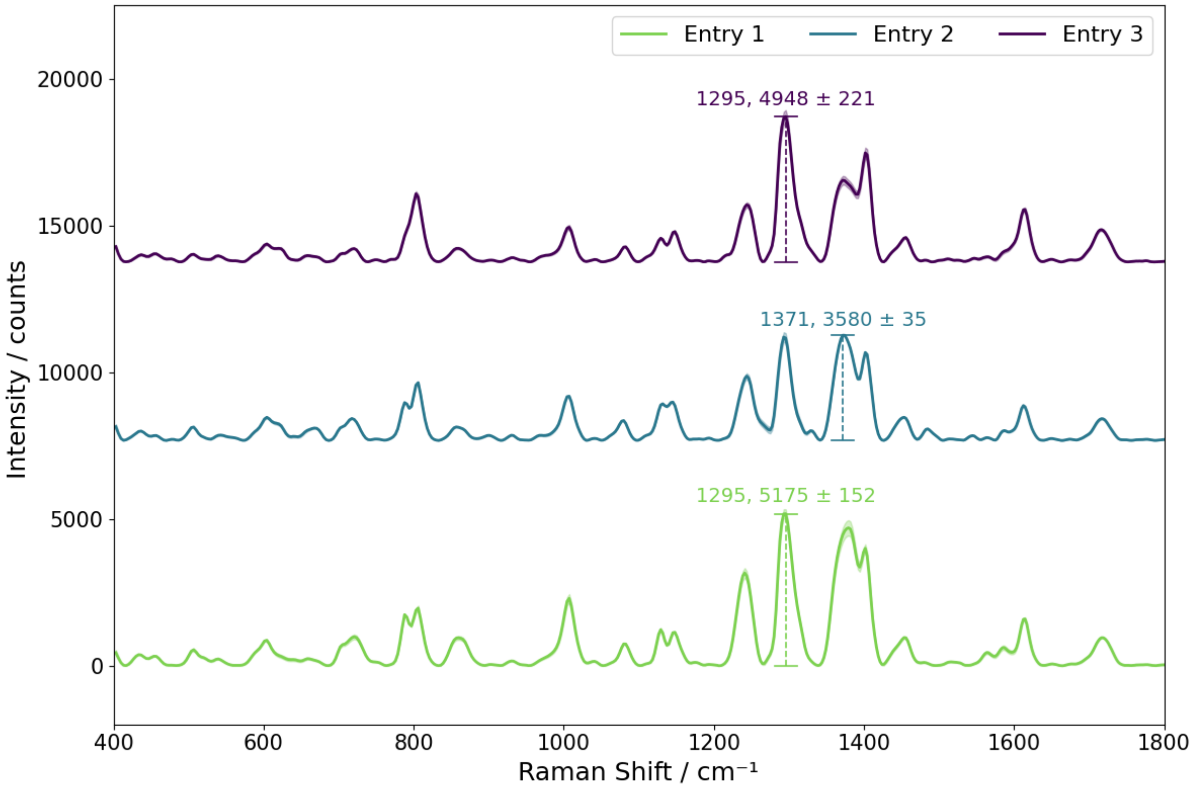

Figure S13. SERS spectra obtained from entries in **Table S4**.

Table S7. Optimisation entries for **5a** under different conditions. * = no stirring applied, N=1, n=5.

Entry	Solvent	Temperature / °C	[TEAB] / mM	[5a] / mM
1	MeCN	55	5	5
2	MeCN	70	5	5
3	DMF	55	5	5
4	EtOH	55	5	5
5	^{i}PrOAc	55	5	5
6	THF*	55	5	5
7	THF	55	5	5
8	THF	55	1	1
9	2-MeTHF	55	10	1
10	THF	55	10	1
11	THF	55	50	1
12	THF	55	5	10
13	THF	55	5	0.1
14	THF	55	5	1

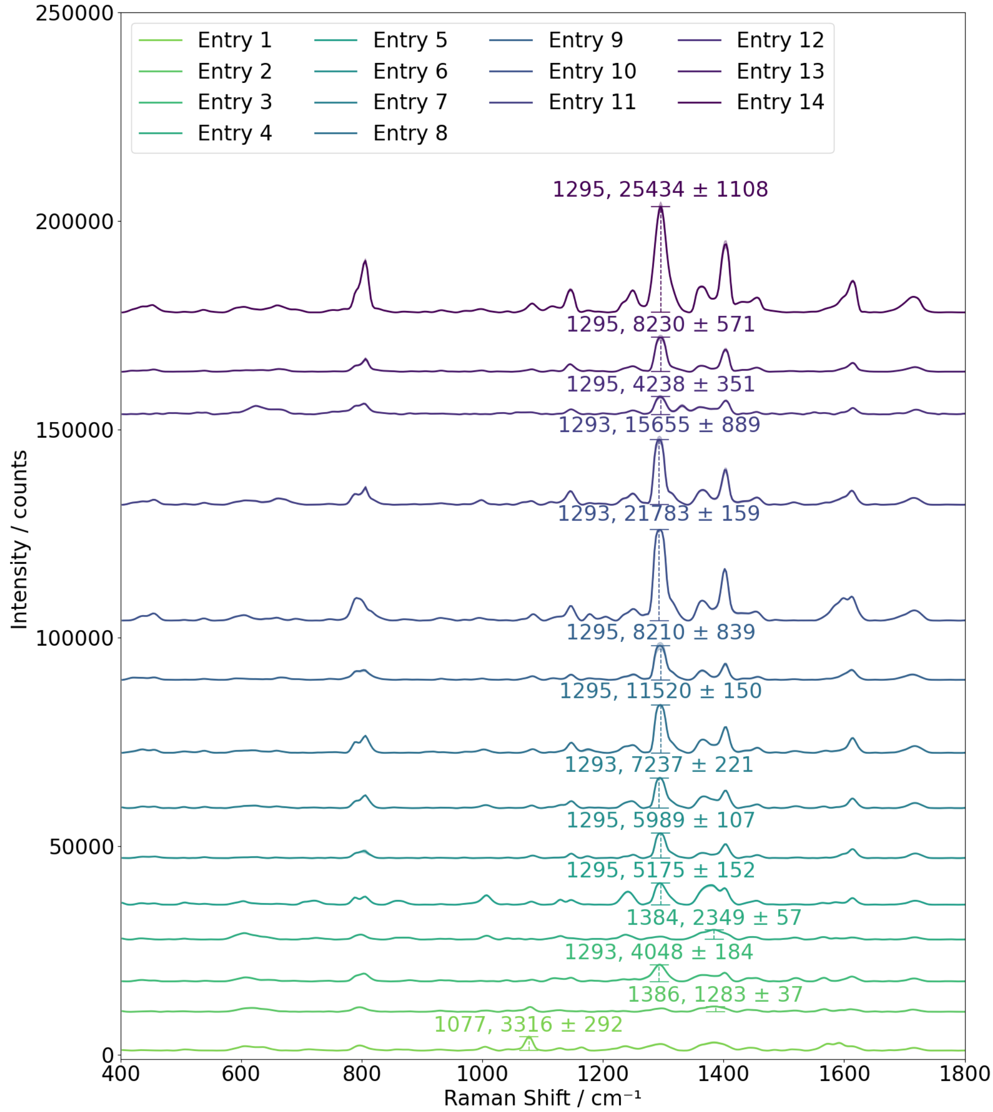

Figure S14. SERS spectra obtained from entries in **Table S5**. For entry 6, N=1, n=5.

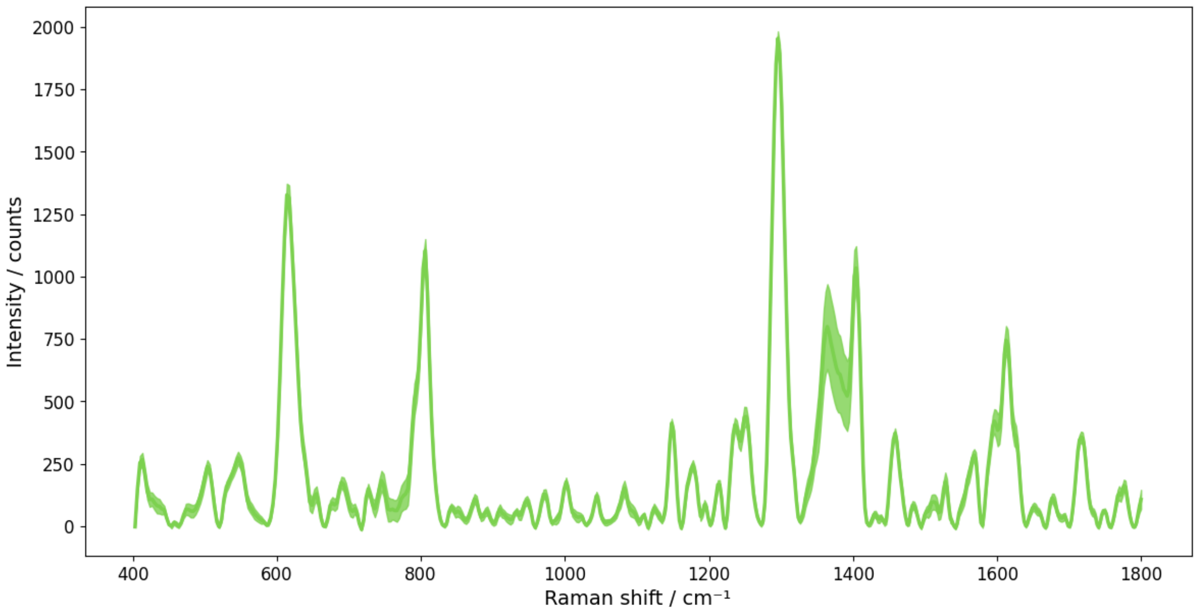

Figure S15. Remaining SERS spectrum of two substrates from **Figure 4a**, entry 6, showing lower functionalisation due to the lack of stirring. N=2, n=5.

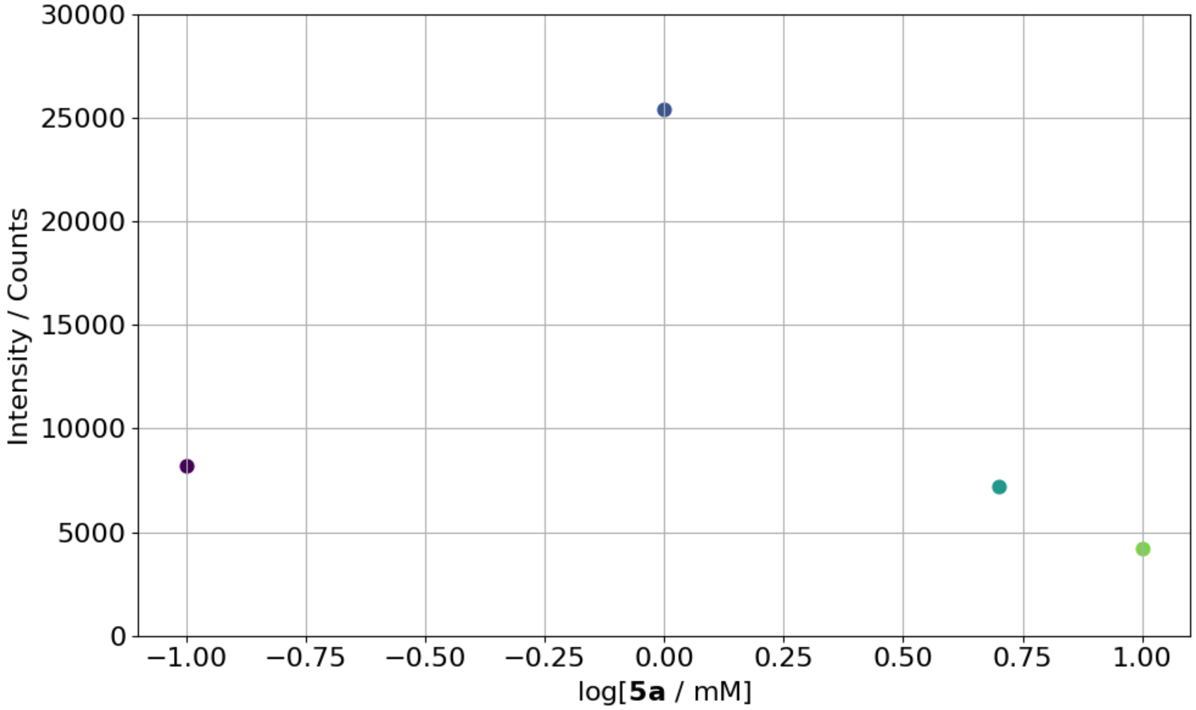

Figure S16. SERS intensity versus concentration of **5a** with a constant concentration of 5 mM for TEAB in THF with 5 Å MS and stirring (entry 7, 12, 13 and 14 from table S7).

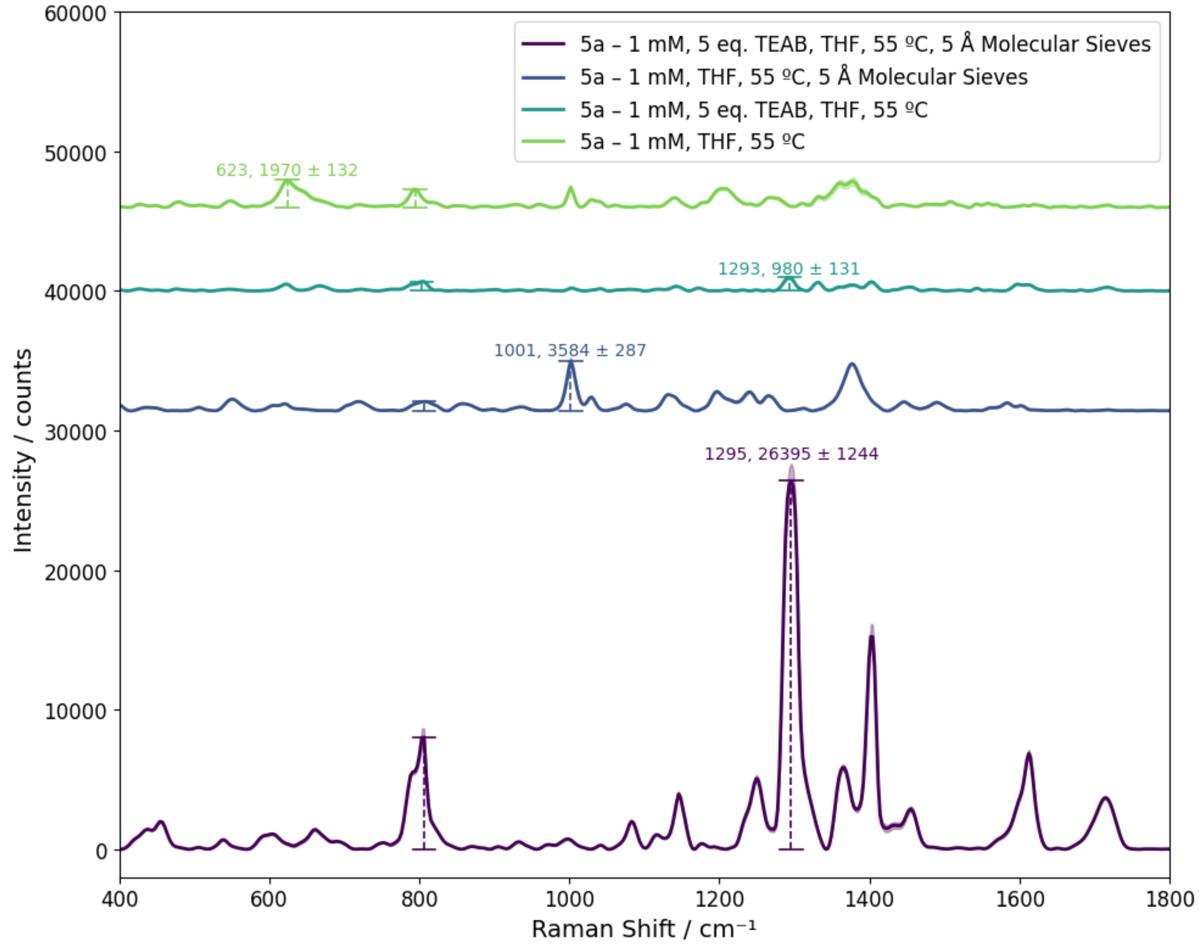

Figure S17. Control experiments utilising **5a** to show the synergism between the TEAB and the 5 Å MS.

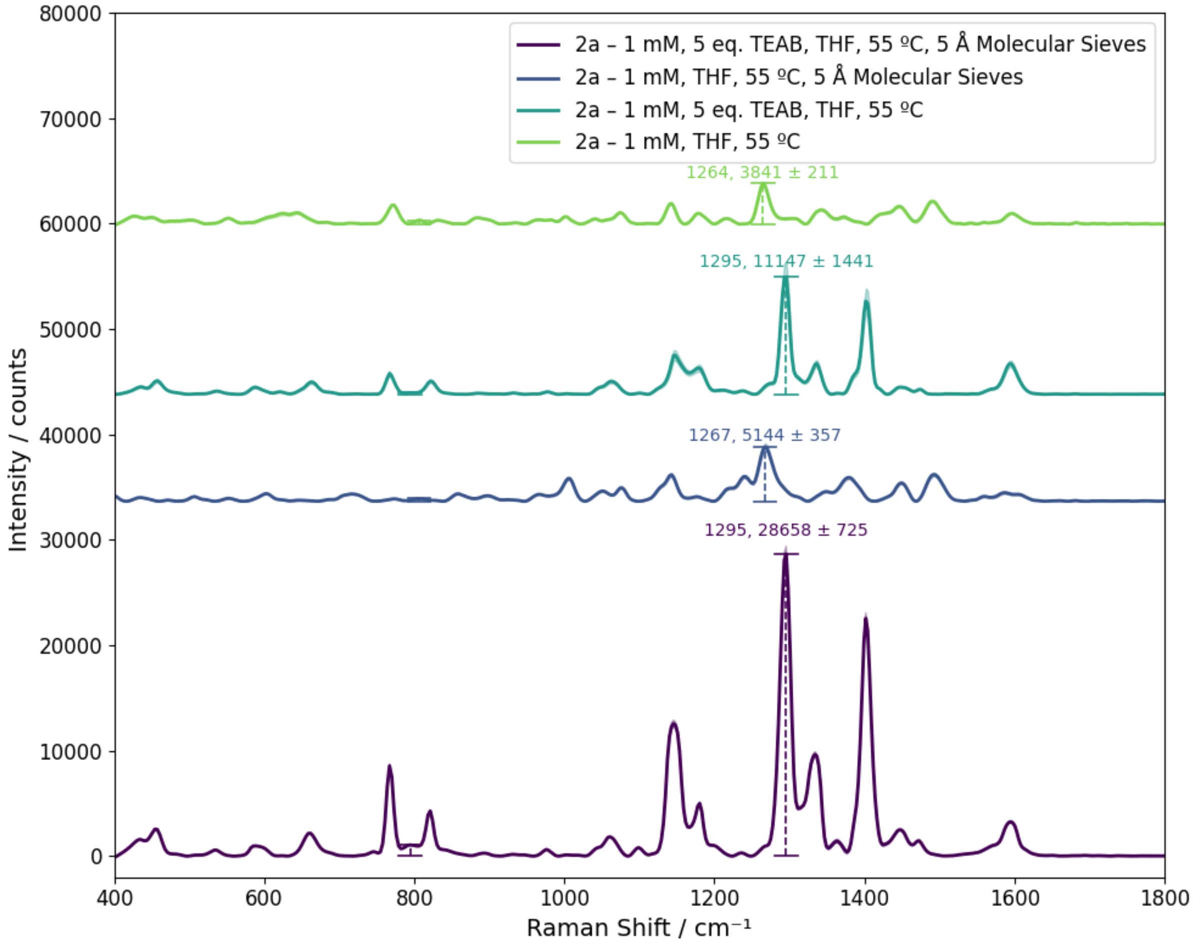

Figure S18. Control experiments utilising **2a** to show the synergism between the TEAB and the 5 Å MS.

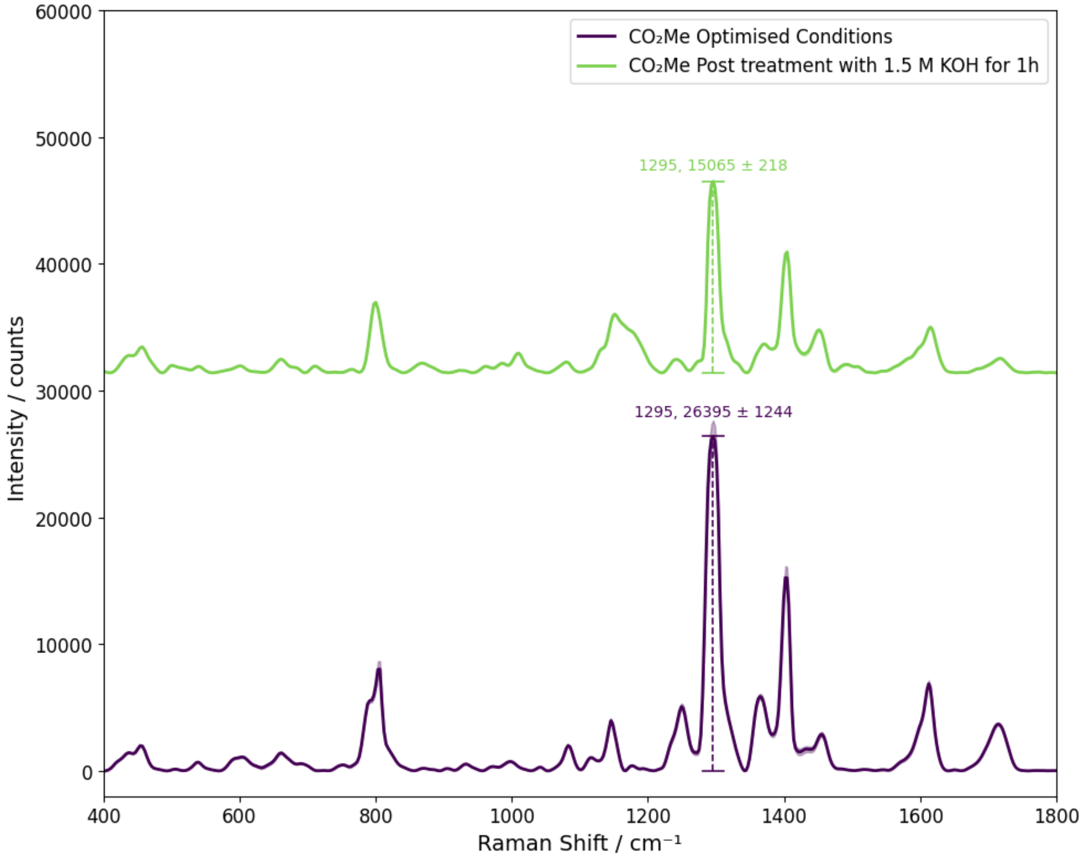

Figure S19. SERS spectra of **5a** before and after treatment with 1.5 M KOH for **1**.

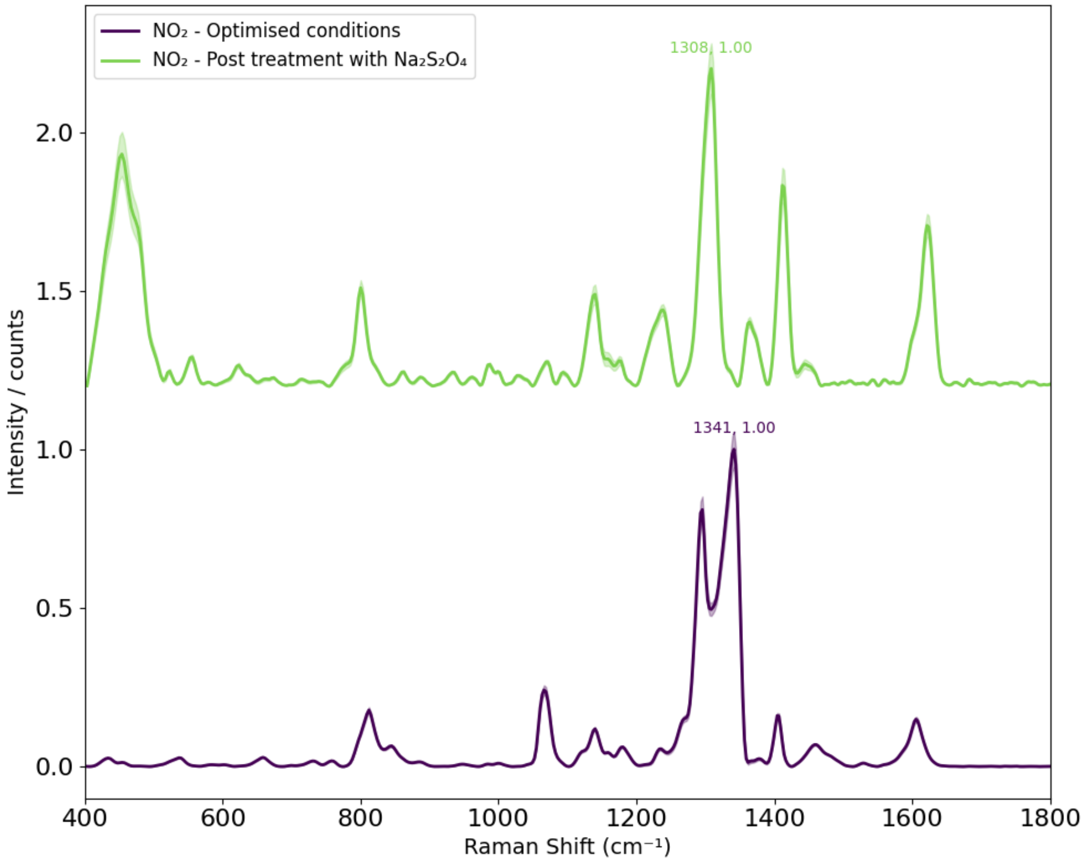

Figure S20. SERS spectra of **8a** before and after treatment with $Na_2S_2O_4$.

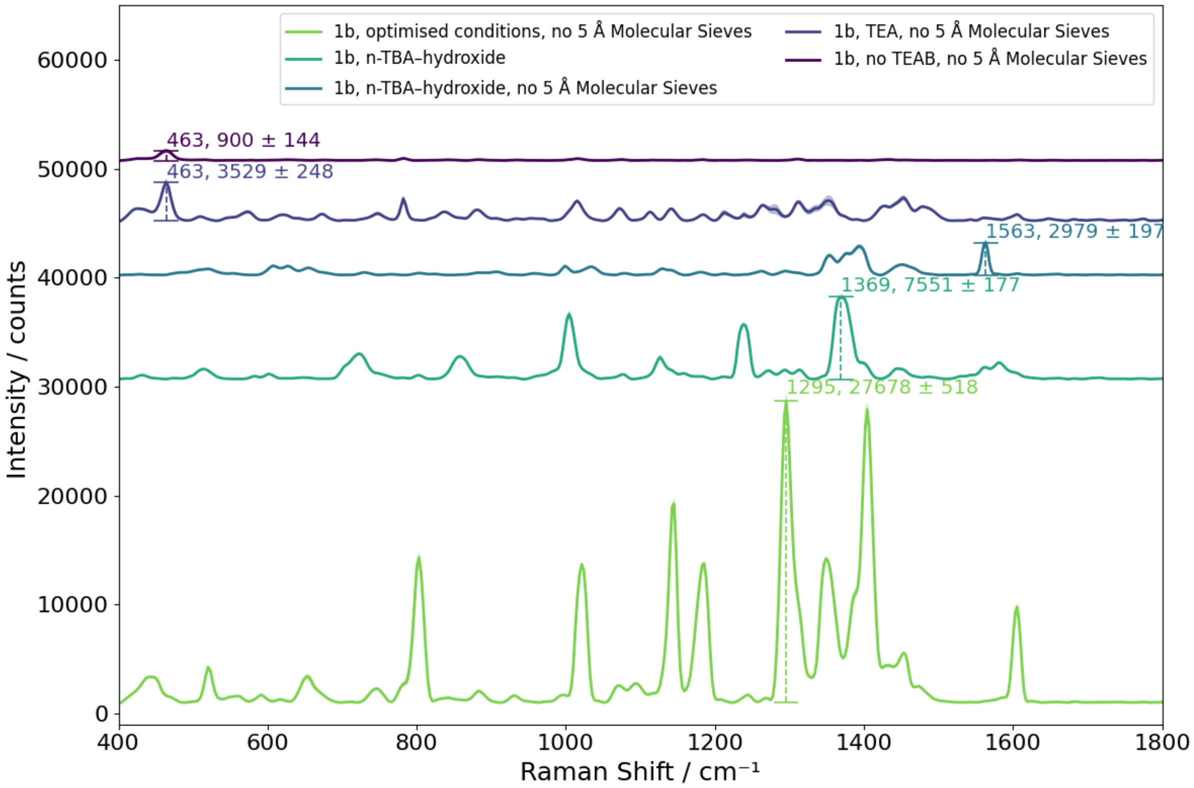

Figure S21. SERS spectra of **1b** under different variations of the optimised condition.

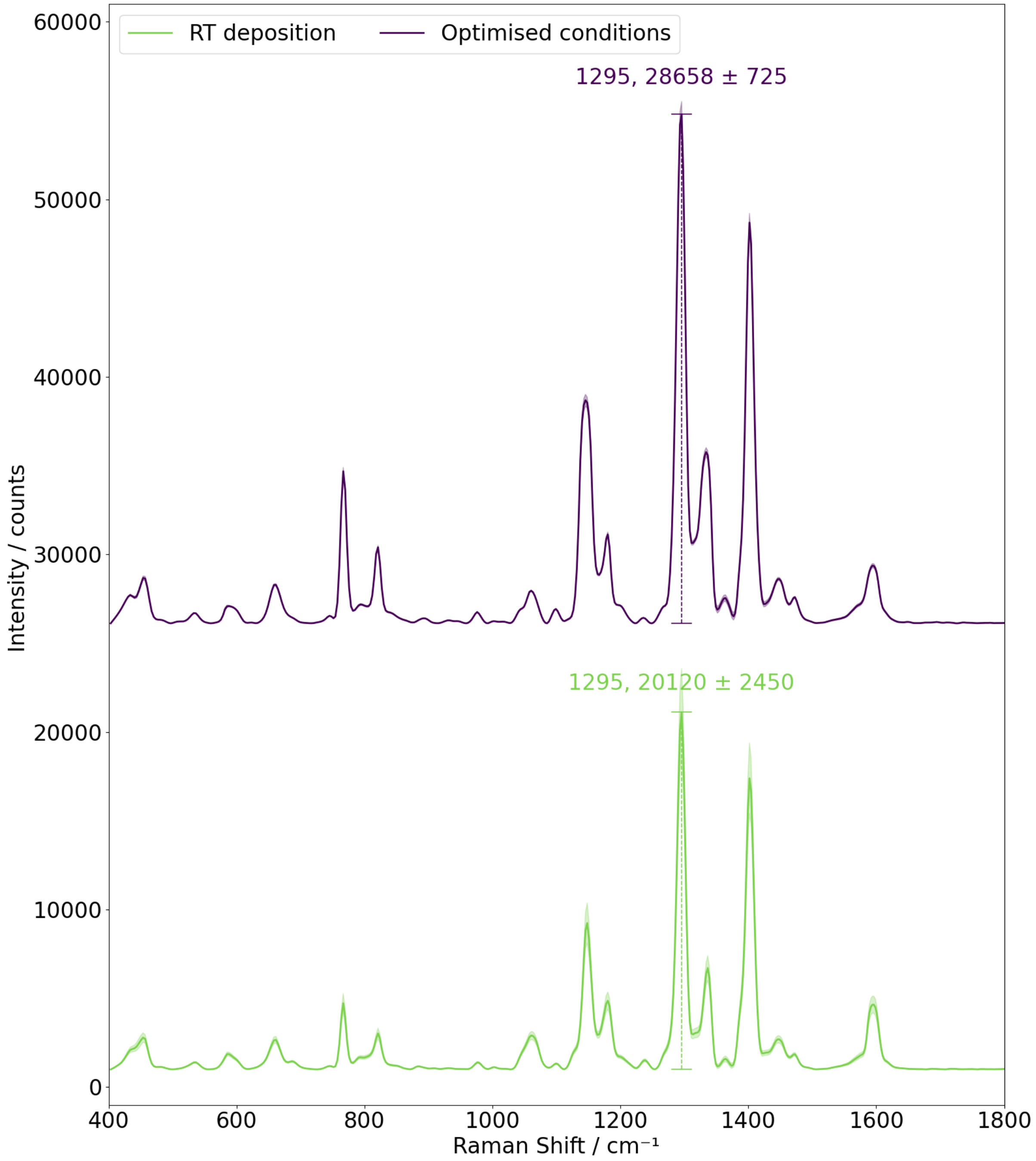

Figure S22. SERS spectra of **2a** deposited using optimised conditions and the same conditions at room temperature.

For comparison, functionalisation of **2a** was attempted using electrochemical deposition developed by Amit *et al.*[10] Unfortunately, only 1 out of 4 chips was successfully functionalised.

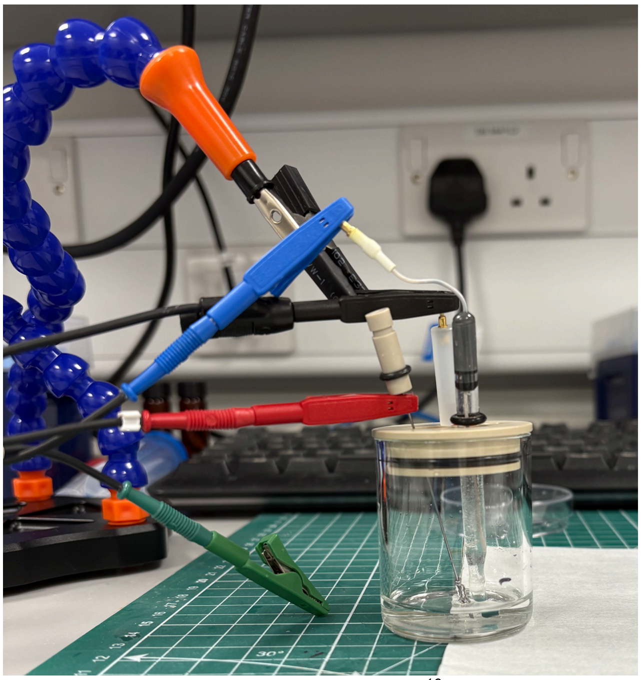

Figure S23. Electrochemical set up used for the deposition of **2a**.[10] The red, blue and black crocodile clips were attached to the working, reference and counter electrode respectively. See general methods above for details.

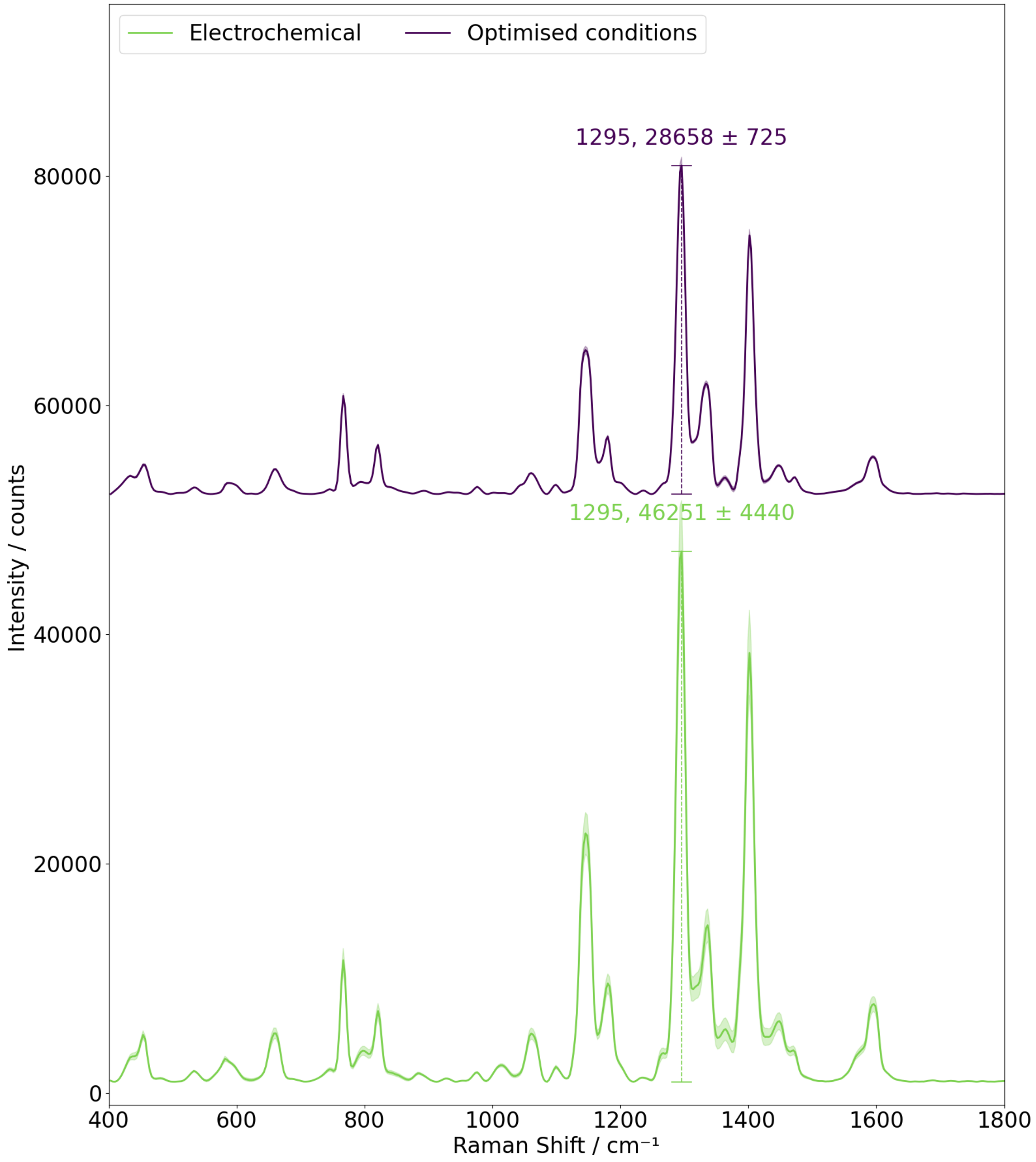

Figure S24. SERS spectra of **2a** deposited using optimised conditions or electrochemical deposition.[10] For the electrochemical deposition, N=1, n=5.

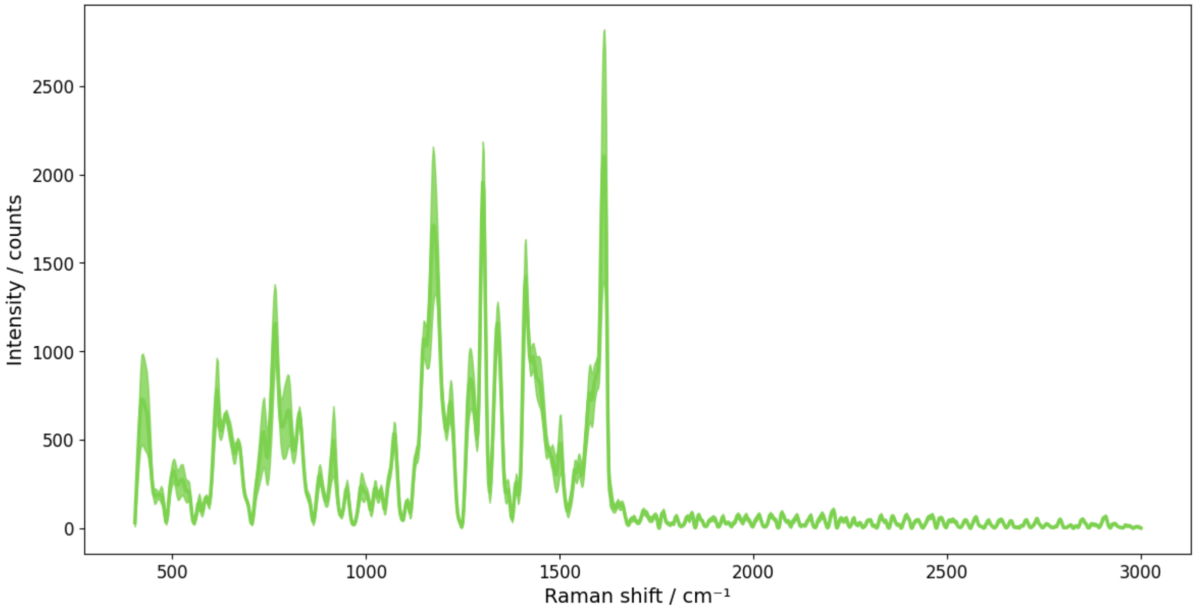

Figure S25. SERS spectra of the failed electrochemical deposition of **2a**.[10]

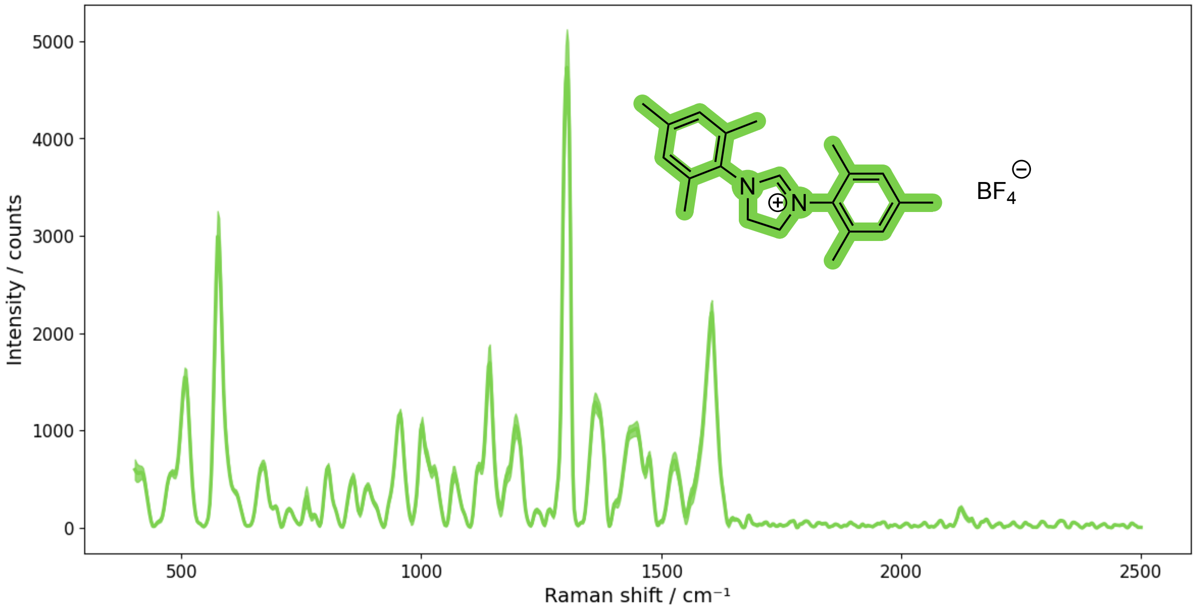

Figure S26. SERS spectrum of **11h** under optimised conditions.

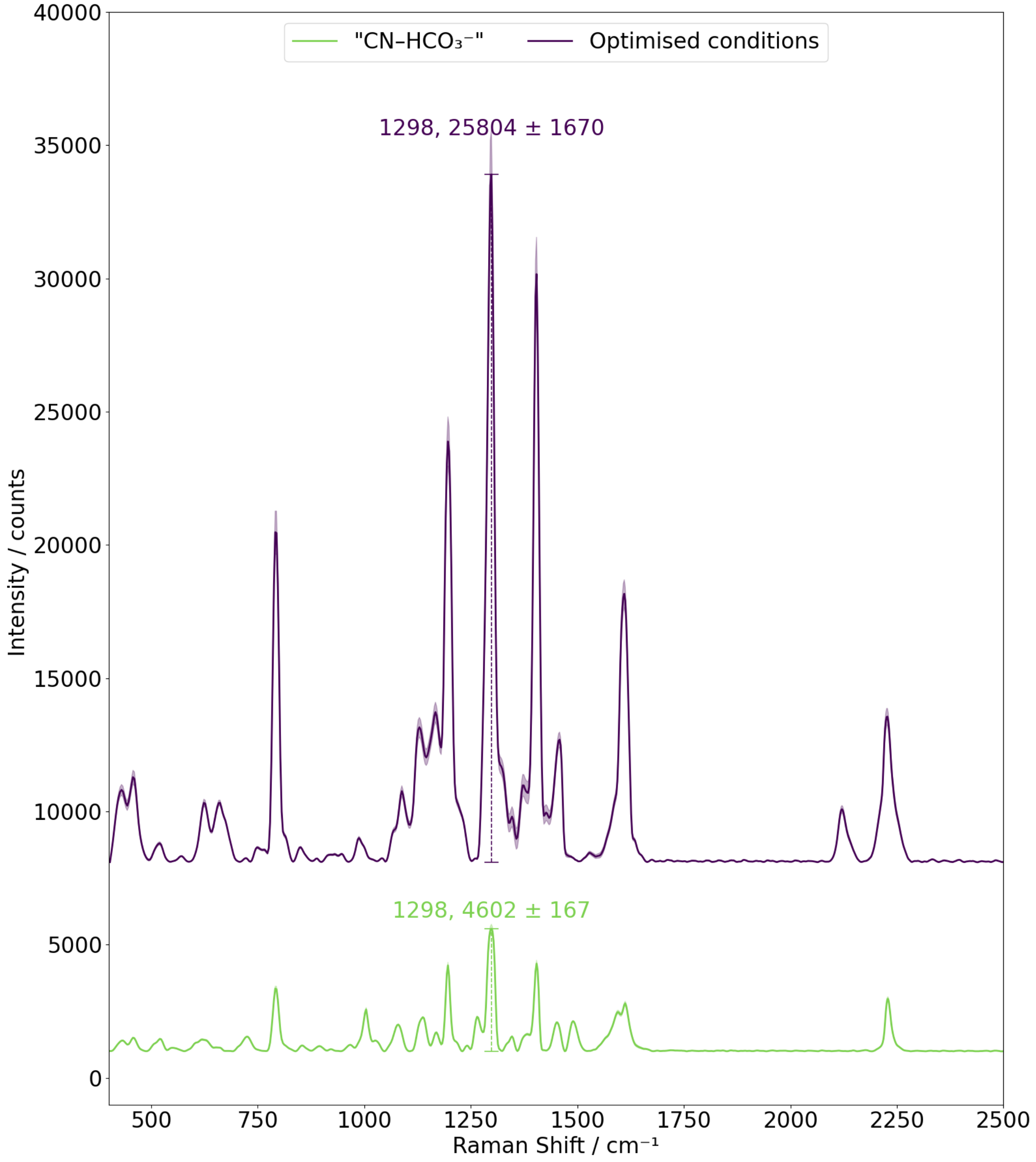

Figure S27. Comparison of SERS spectra obtained from the deposition of **7** under optimised conditions using **7a**, and by using directly the eluent from an anion exchange column for **7c** in EtOH. Both functionalisations were carried out at 55 ºC using 5 Å MS.

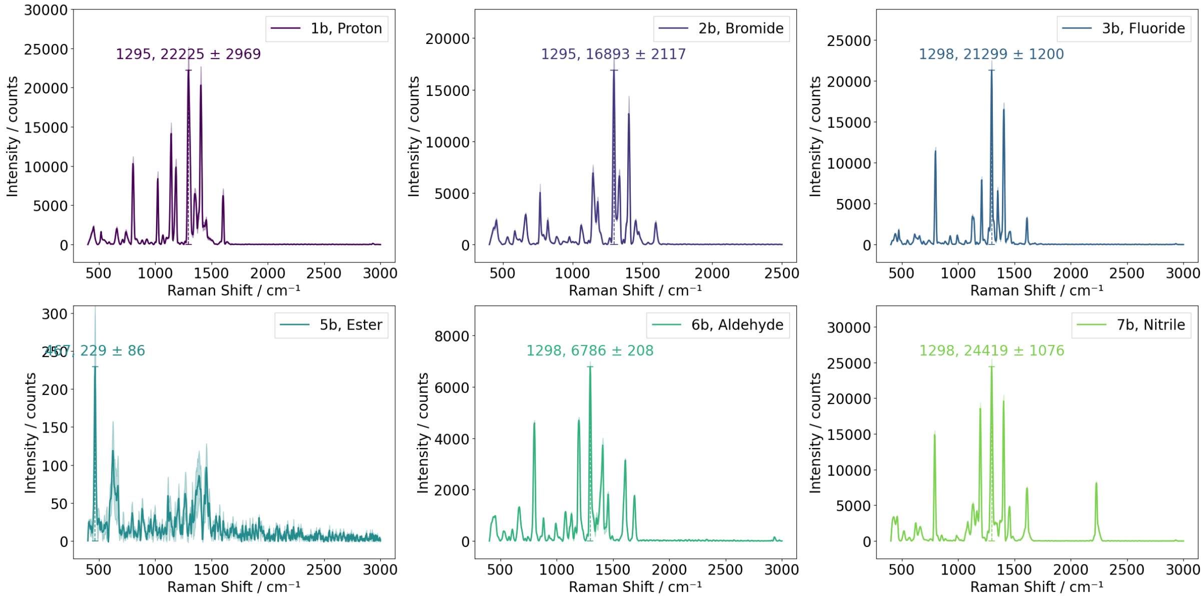

Figure S28. SERS spectra of monolayers formed from benzimidazolium iodides.

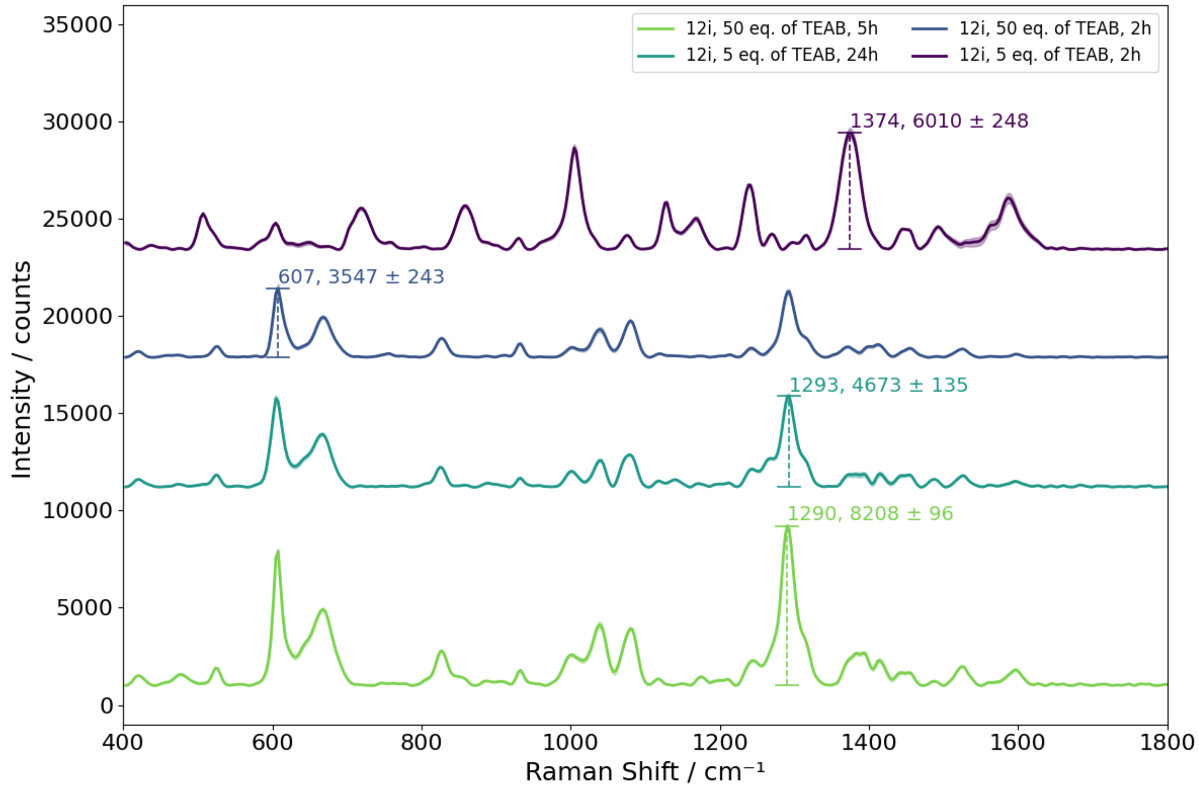

Figure S29. Optimisation of MIC-substrate **12i**.

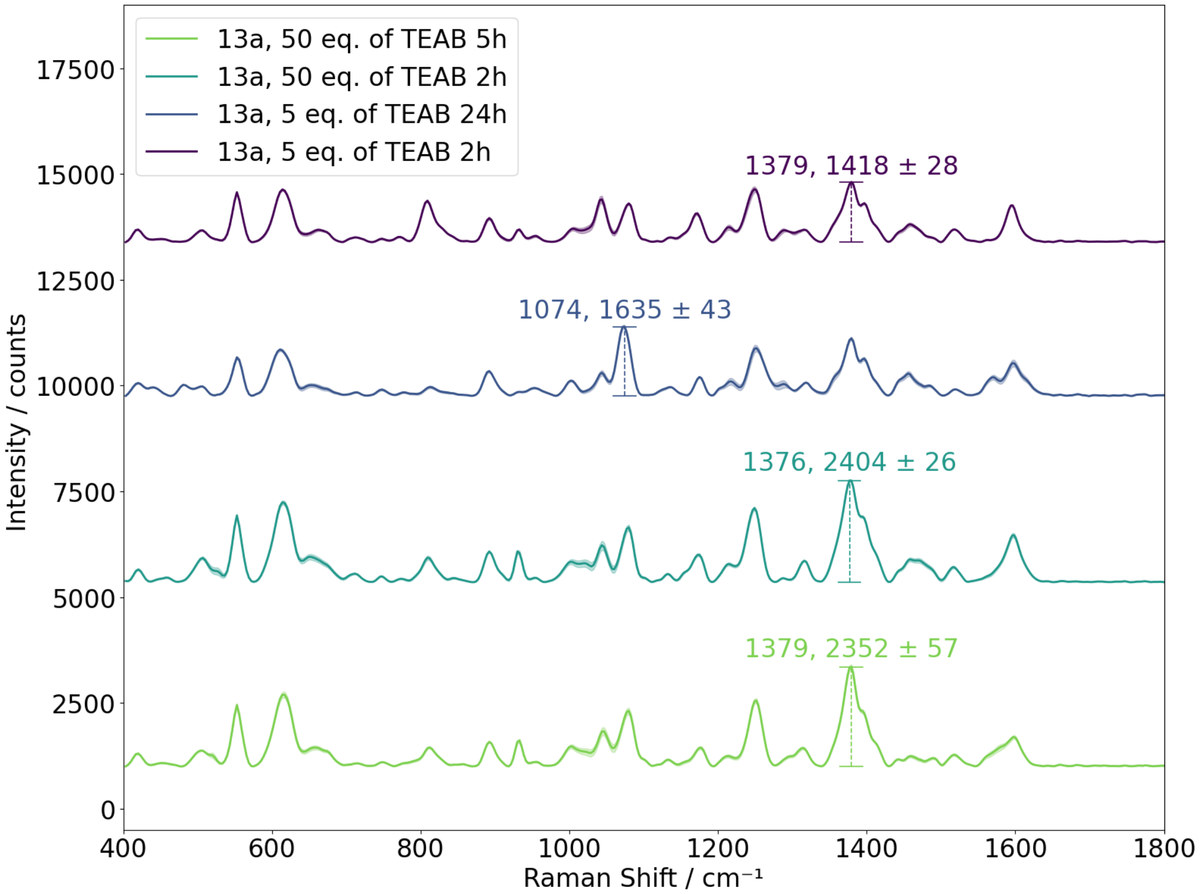

Figure S30. Optimisation of MIC-substrate **13a**.

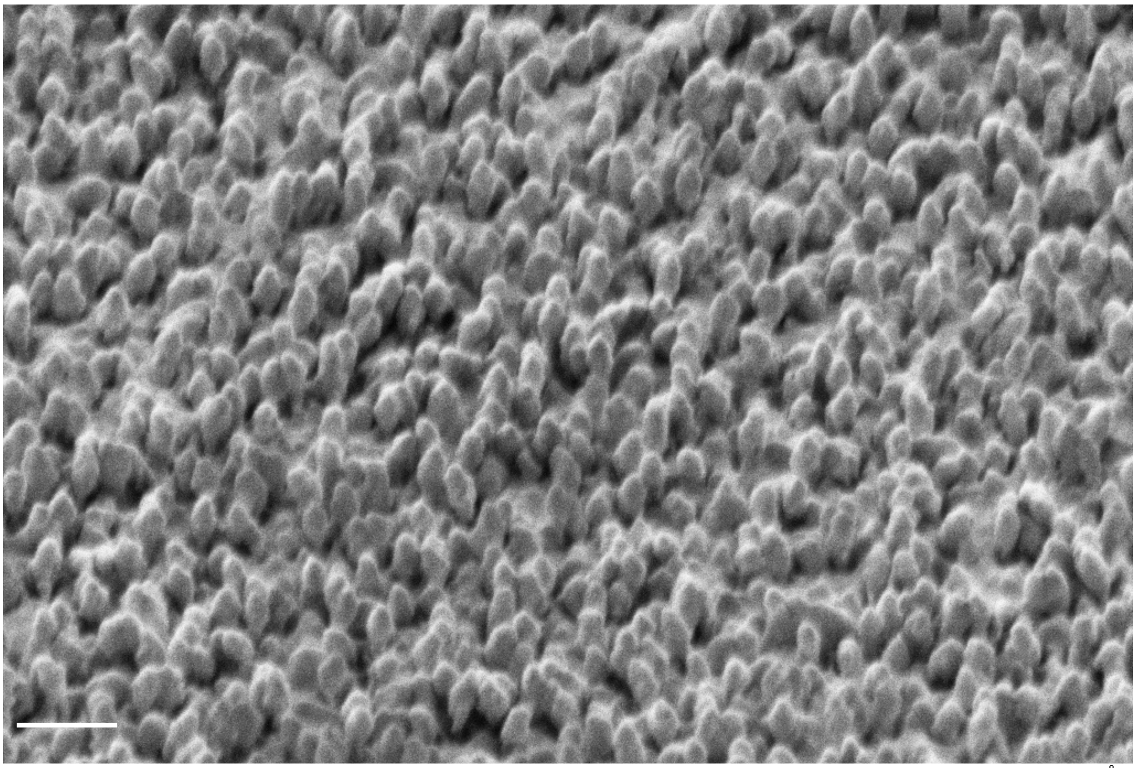

Figure S31. SEM image of the SERS chips after deposition of **2a** using optimised conditions minus the 5 Å MS. Scale bar = 400 nm.

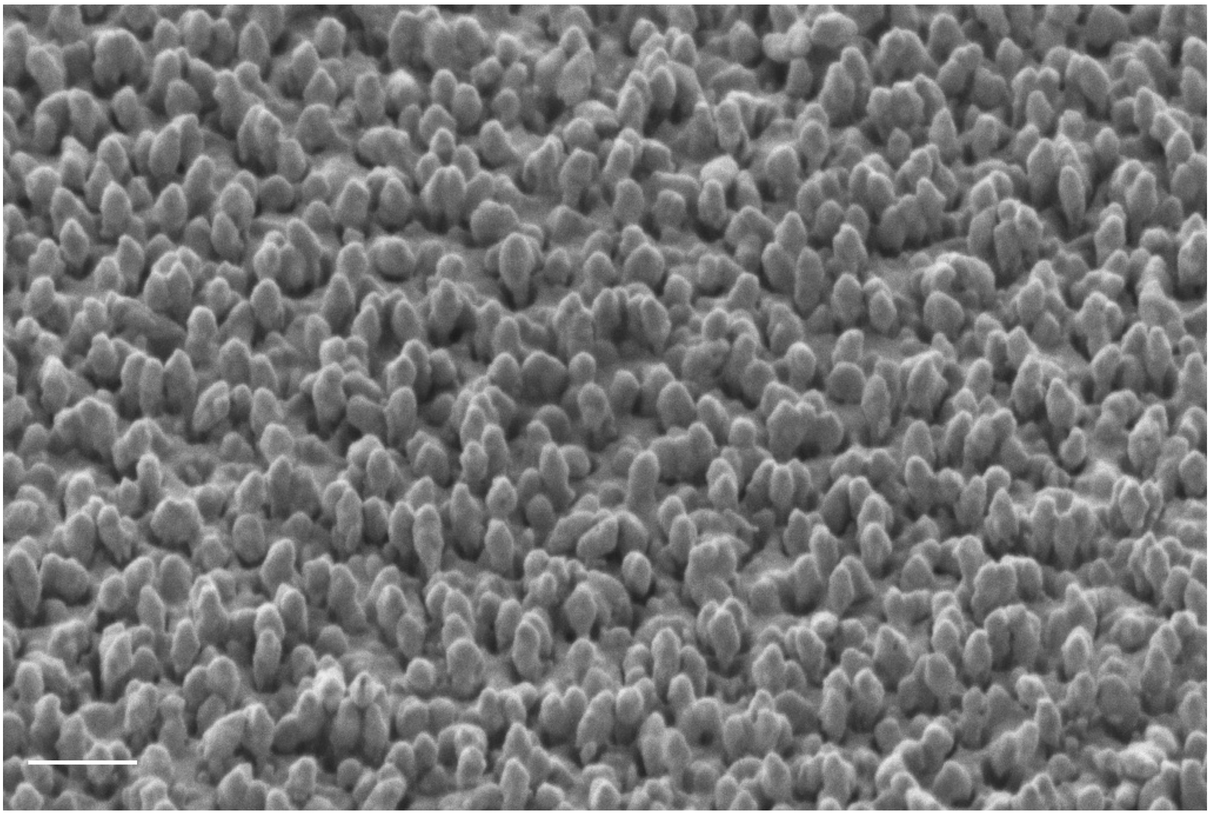

Figure S32. SEM image of the SERS chips after deposition of **2a** using optimised conditions. Scale bar = 400 nm.

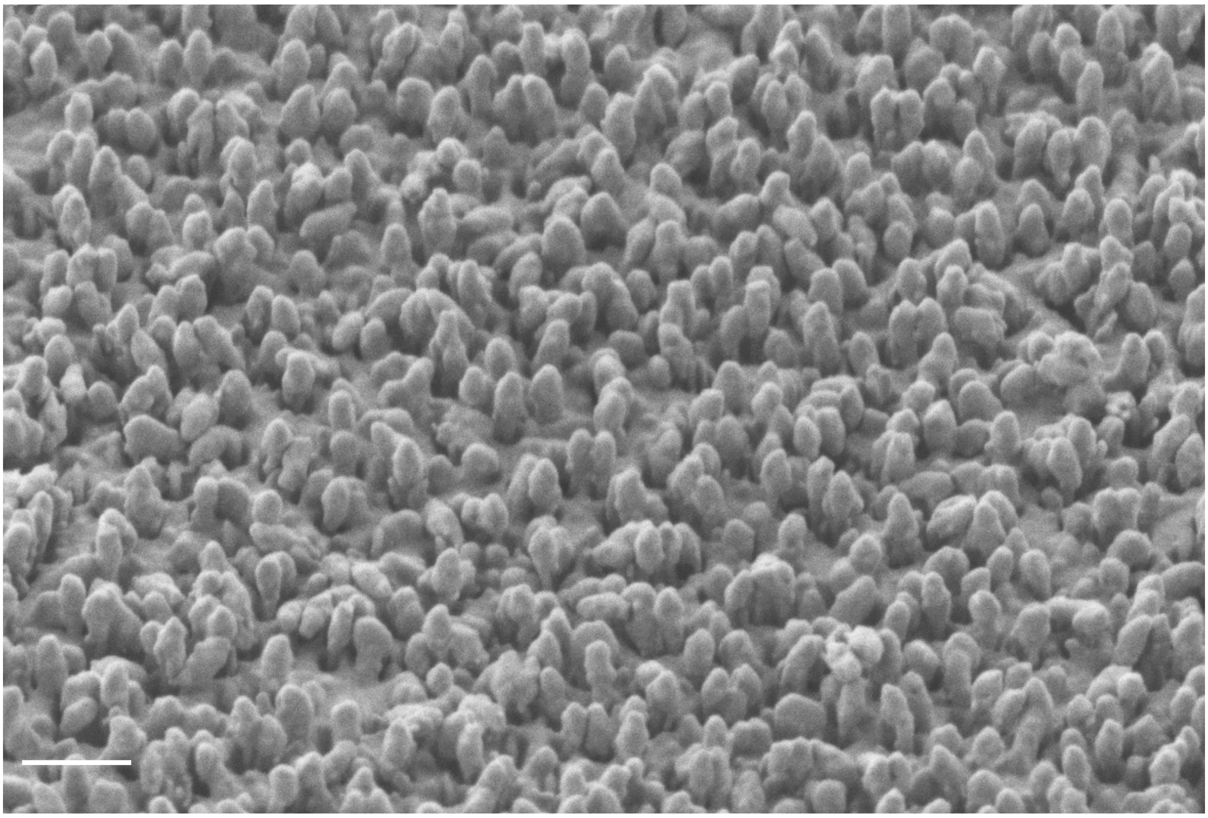

Figure S33. SEM image of the SERS chips after deposition of **2a** using electrochemical deposition. Scale bar = 400 nm.

Note: the optimised conditions were also trialled on silver surfaces but with no success. Increasing temperature, substrate, TEAB equivalences or incubation time had no beneficial effect.

S6 LDI Experiments

Table S8. Species of interest in the LDI spectrum of gold-substrates functionalised with **5a**.

Species	Mass Distribution / m/z (Abundance / %)
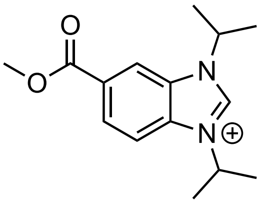	261.1598 (100.0), 262.1632 (16.2), 263.1665 (1.2)
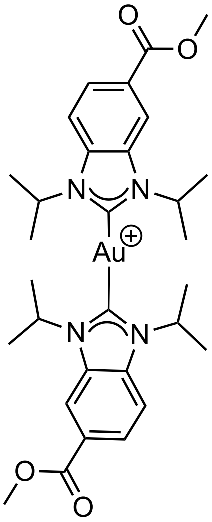	717.2710 (100.0), 718.2744 (32.4), 719.2777 (5.1), 718.2681 (1.5)

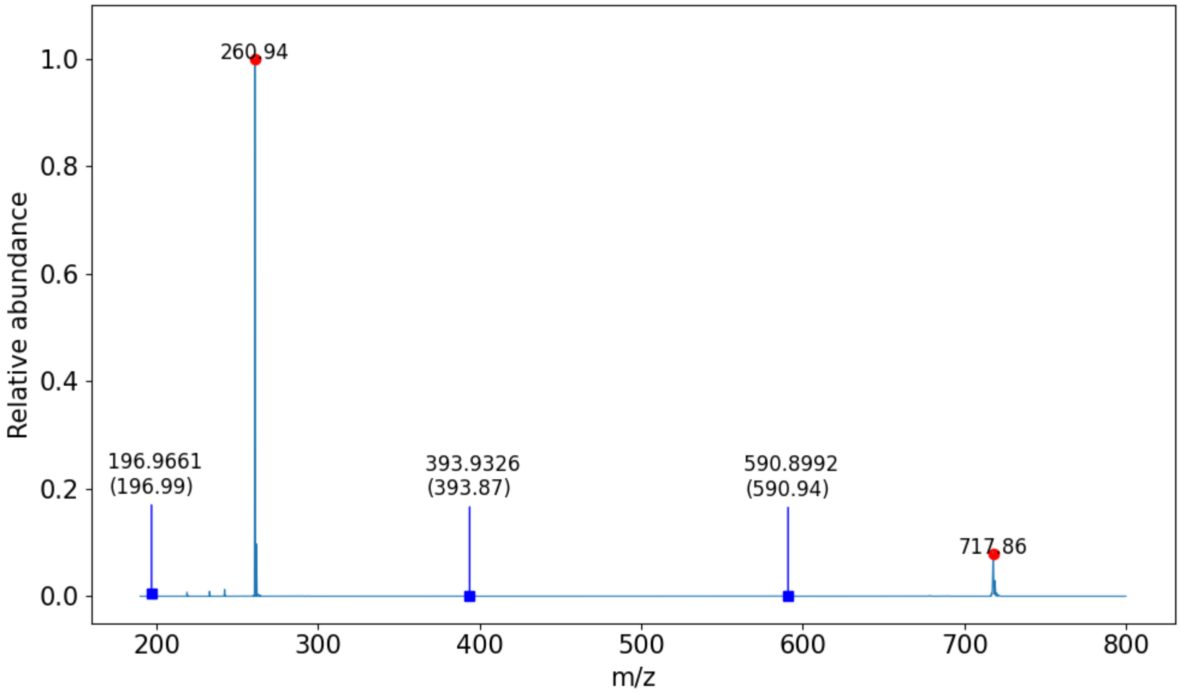

Figure S34. LDI spectrum of a gold-substrates functionalised with **5a** using optimised conditions, entry 12, **Figure 4a**.

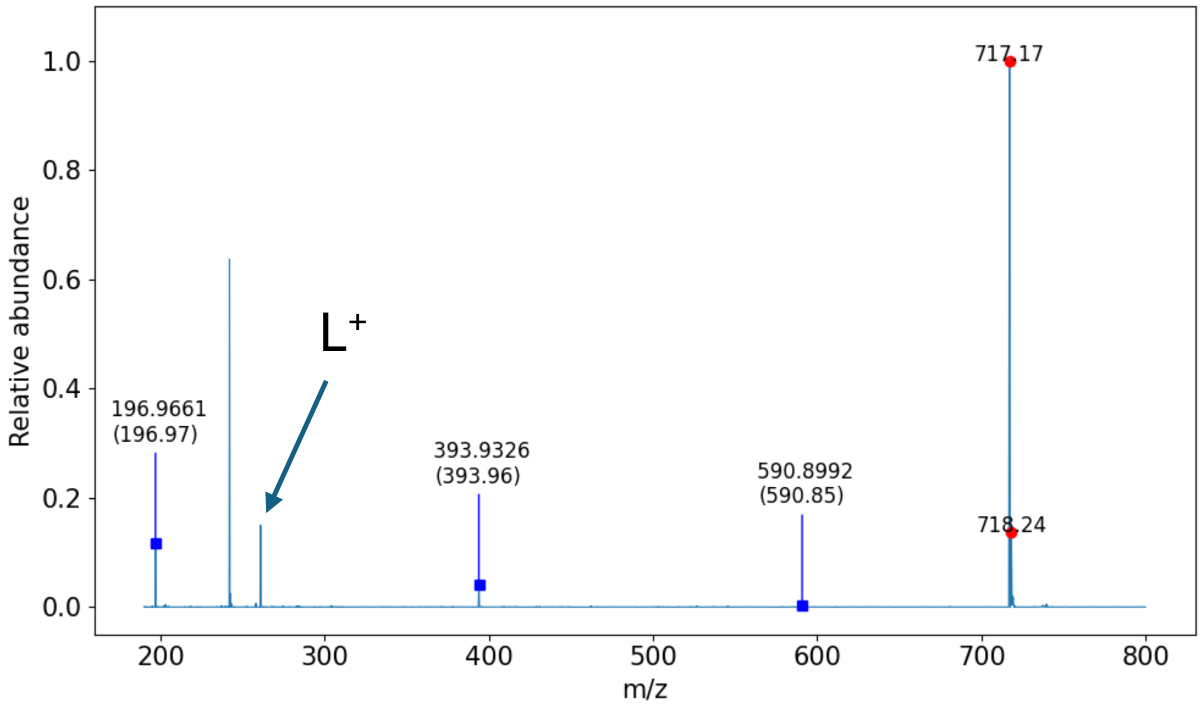

Figure S35. LDI spectrum of a gold-substrates functionalised with **5a** using optimised conditions, entry 12, **Figure 4a**, minus the 5 Å Molecular Sieves.

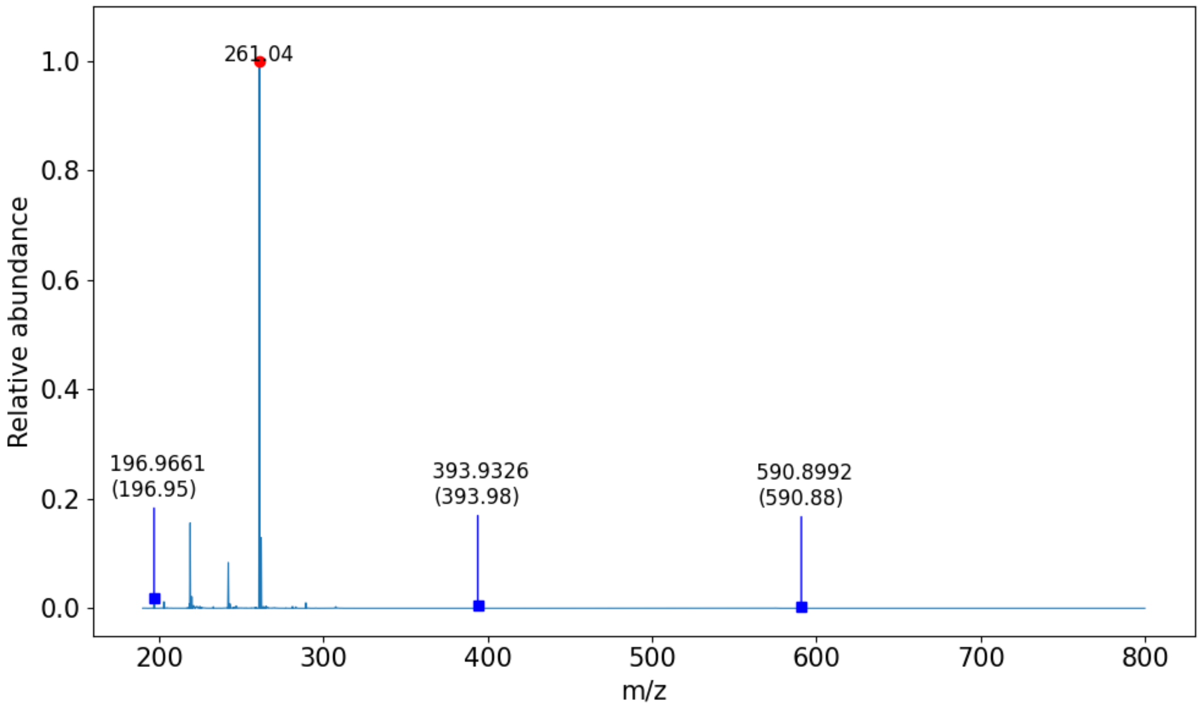

Figure S36. LDI spectrum of a gold-substrates functionalised with **5a** using optimised conditions, entry 12, **Figure 4a**, minus the TEAB.

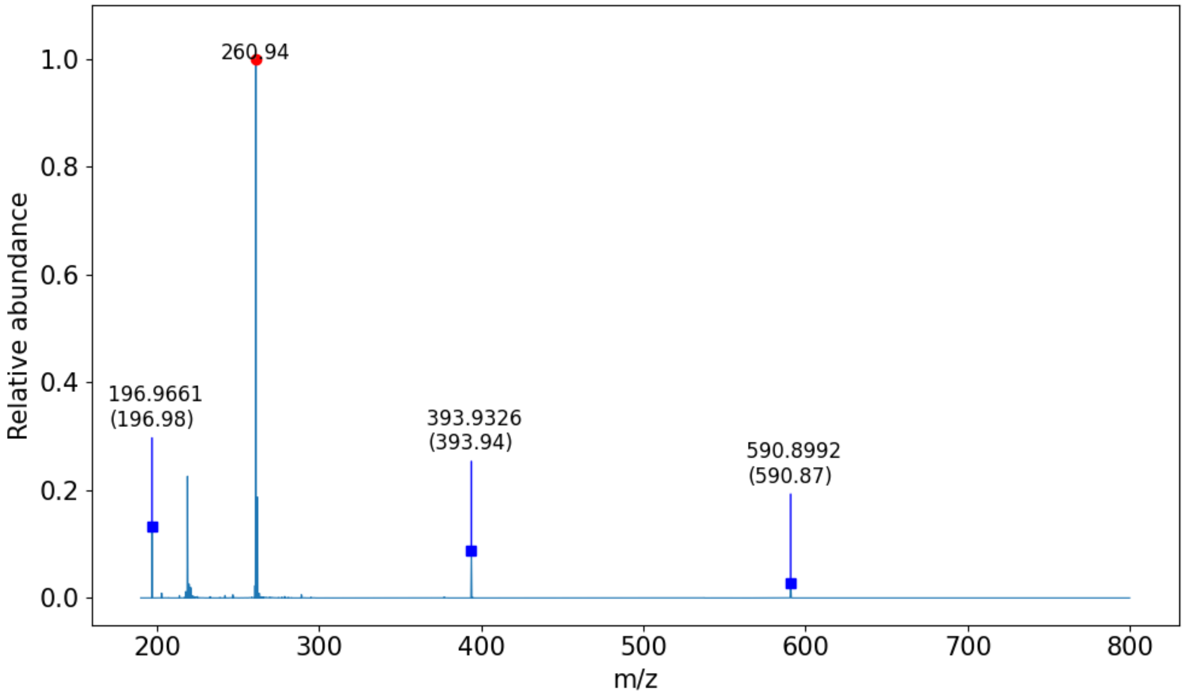

Figure S37. LDI spectrum of a gold-substrates functionalised with **5a** using optimised conditions, entry 12, **Figure 4a**, minus the TEAB and the 5 Å Molecular Sieves.

Table S9. Species of interest in the LDI spectrum of gold-substrates functionalised with **2**.

Species	Mass Distribution / m/z (Abundance / %)
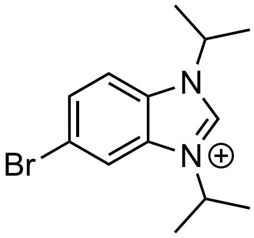	281.0648 (100.0), 283.0628 (97.3), 282.0682 (14.1), 284.0661 (13.7)
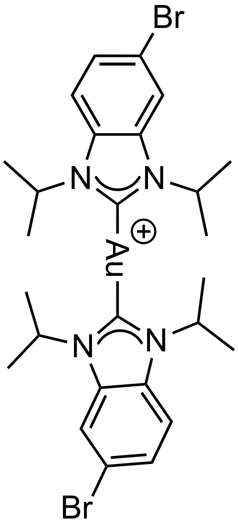	759.0790 (100.0), 757.0811 (51.4), 761.0770 (48.6), 760.0824 (28.1), 758.0844 (14.5), 762.0804 (13.7), 761.0858 (3.8), 759.0878 (2.0%), 763.0837 (1.8), 760.0761 (1.5)

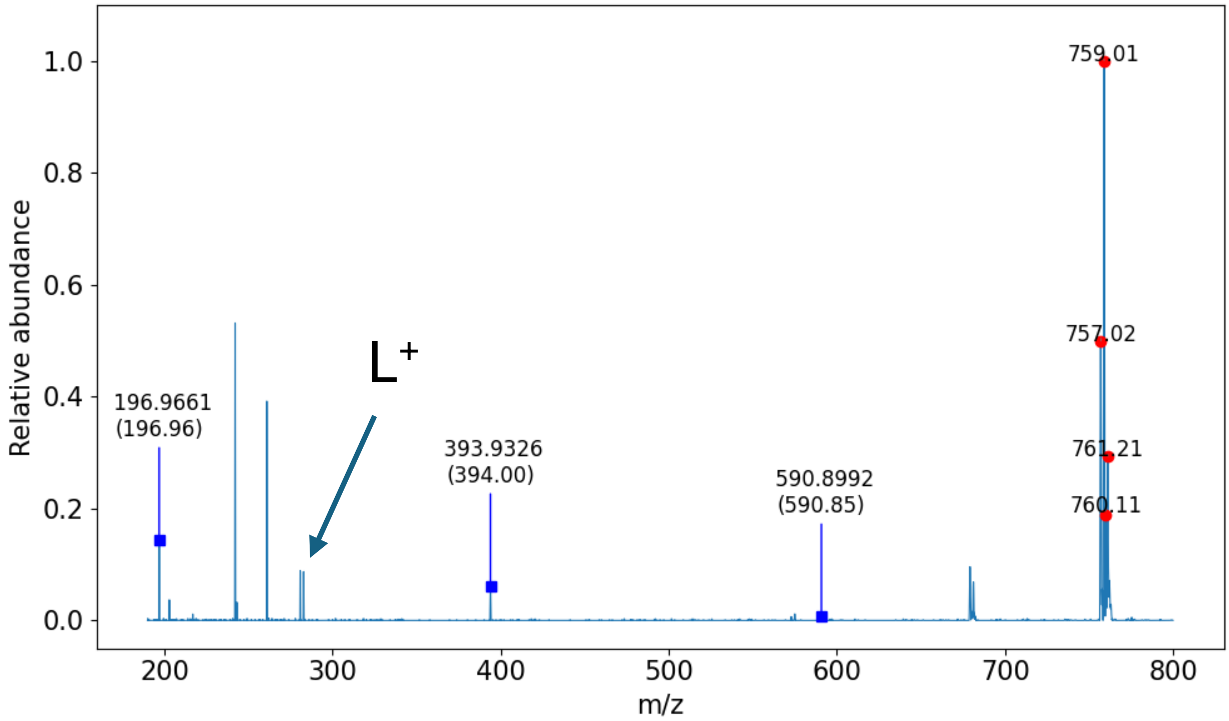

Figure S38. LDI spectrum of a gold-substrates functionalised with **2a** using optimised conditions.

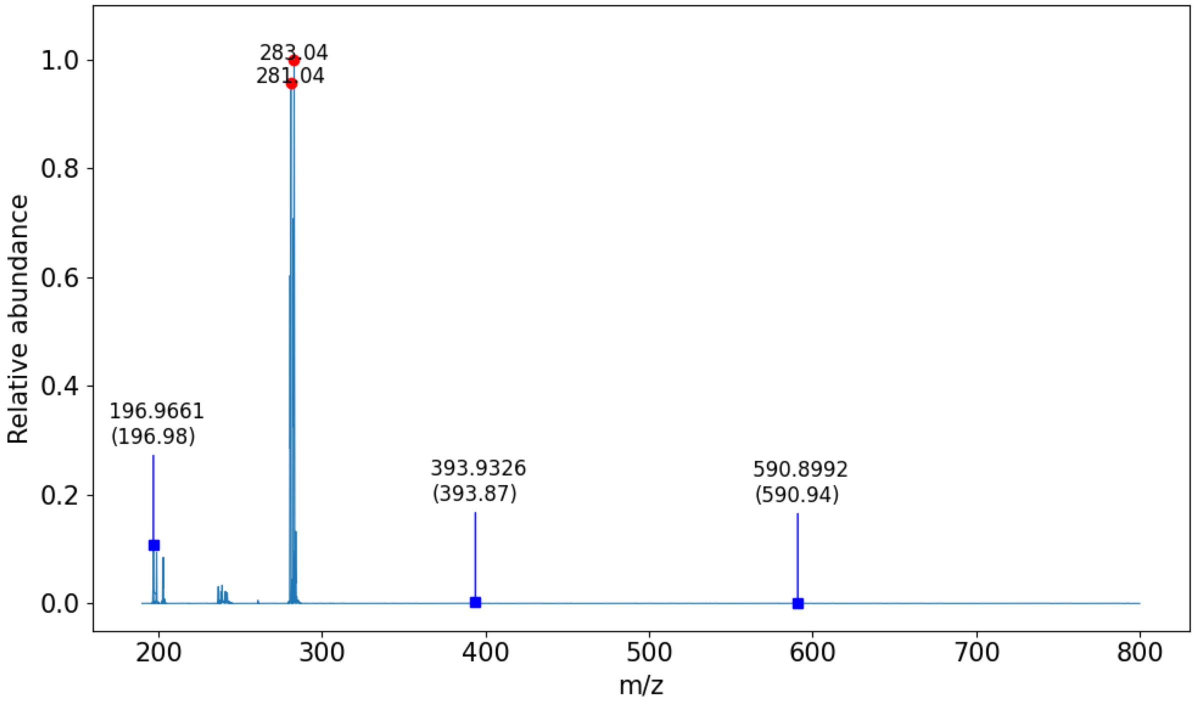

Figure S39. LDI spectrum of a gold-substrates functionalised with **2a** using optimised conditions without TEAB.

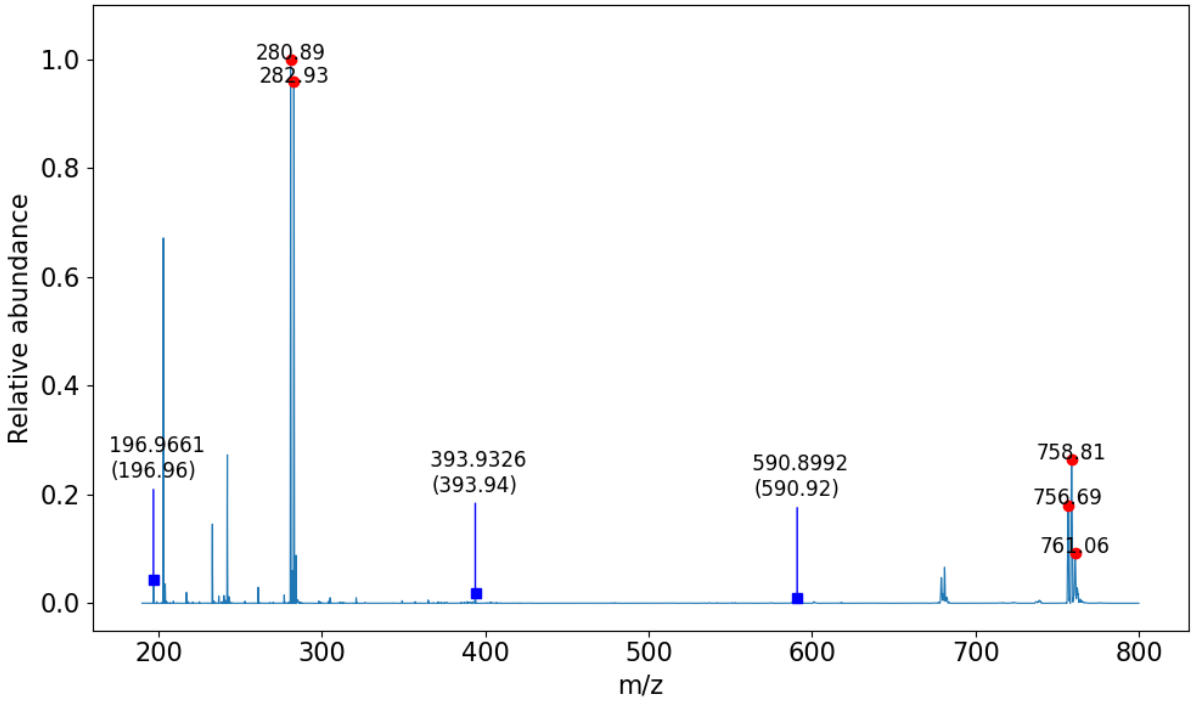

Figure S40. LDI spectrum of a gold-substrates functionalised with **2a** using optimised conditions without 5 Å MS.

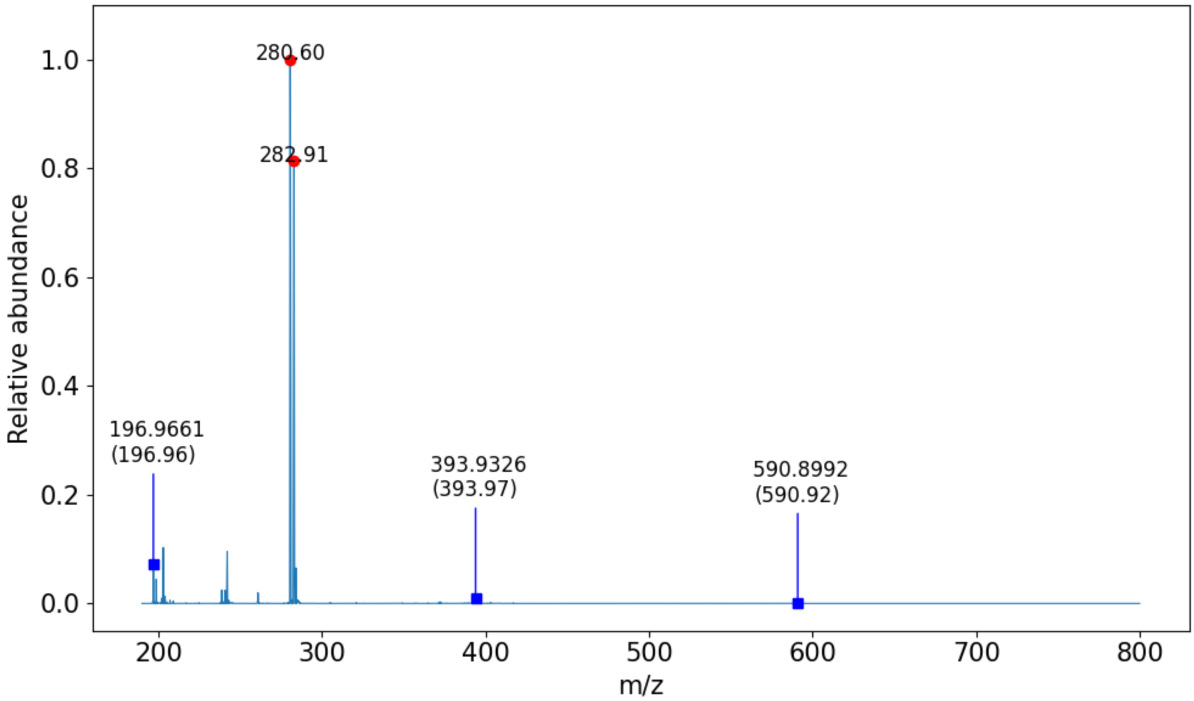

Figure S41. LDI spectrum of a gold-substrates functionalised with **2a** using optimised conditions without 5 Å MS and TEAB.

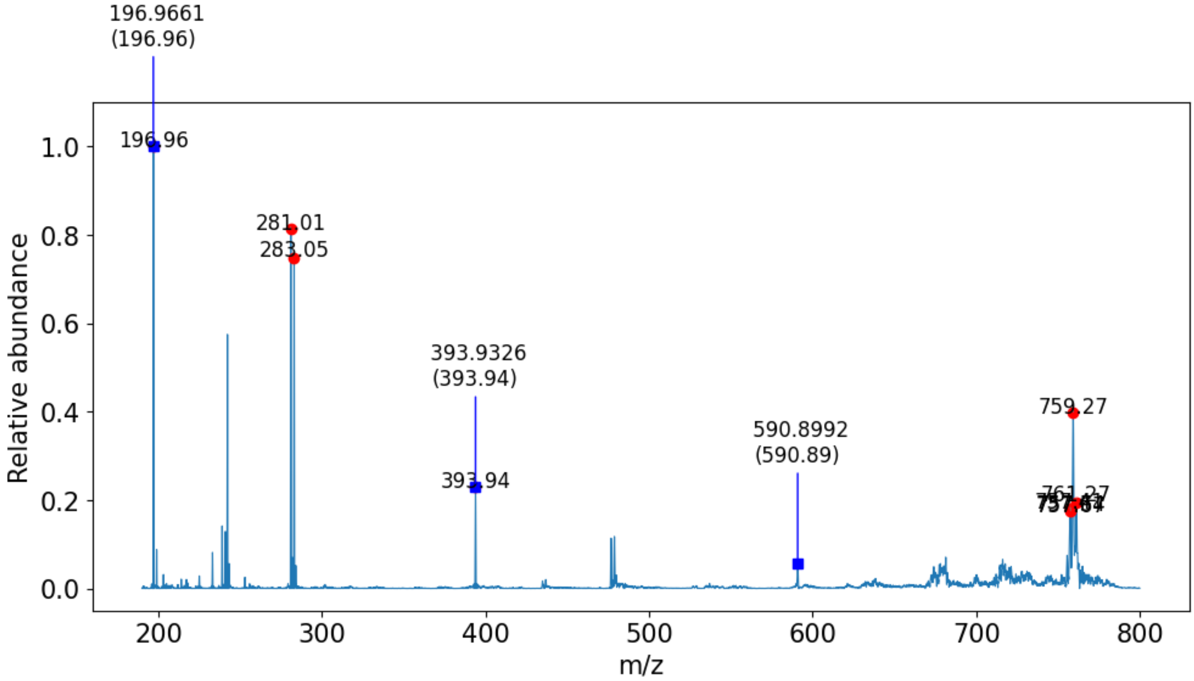

Figure S42. LDI spectrum of a gold-substrates functionalised with **2b** using optimised conditions.

Table S10. Species of interest in the LDI spectrum of gold-substrates functionalised with **12i**.

Species	Mass Distribution / m/z (Abundance / %)
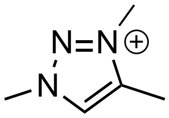	112.0870 (100.0), 113.0903 (5.4), 113.0840 (1.1%)
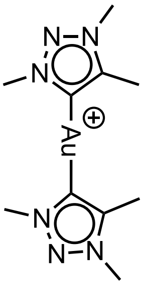	419.1254 (100.0), 420.1287 (10.8), 420.1224 (2.2)

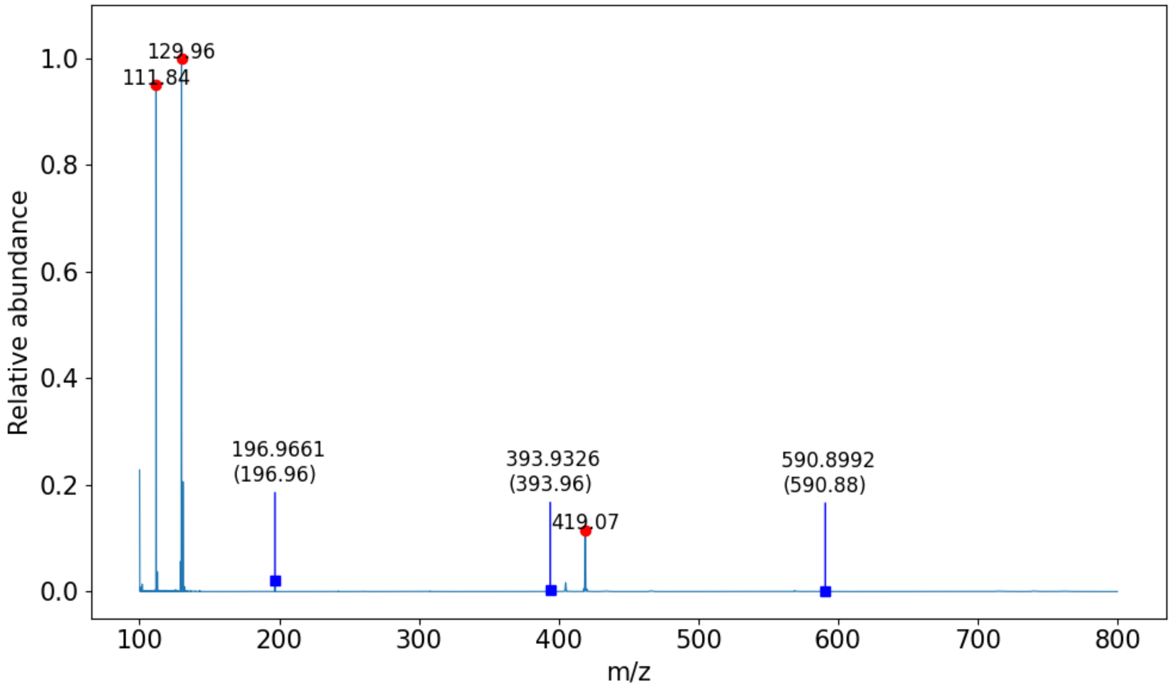

Figure S43. LDI spectrum of a gold-substrates functionalised with **12i** using optimised conditions for MIC after 2 h.

Table S11 Species of interest in the LDI spectrum of gold-substrates functionalised with **13a**.

Species	Mass Distribution / m/z (Abundance / %)
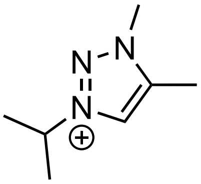	140.1183 (100.0), 141.1216 (7.6), 141.1153 (1.1)
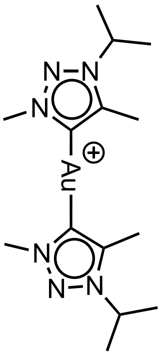	475.1880 (100.0), 476.1913 (15.1), 476.1850 (2.2), 477.1947 (1.1)

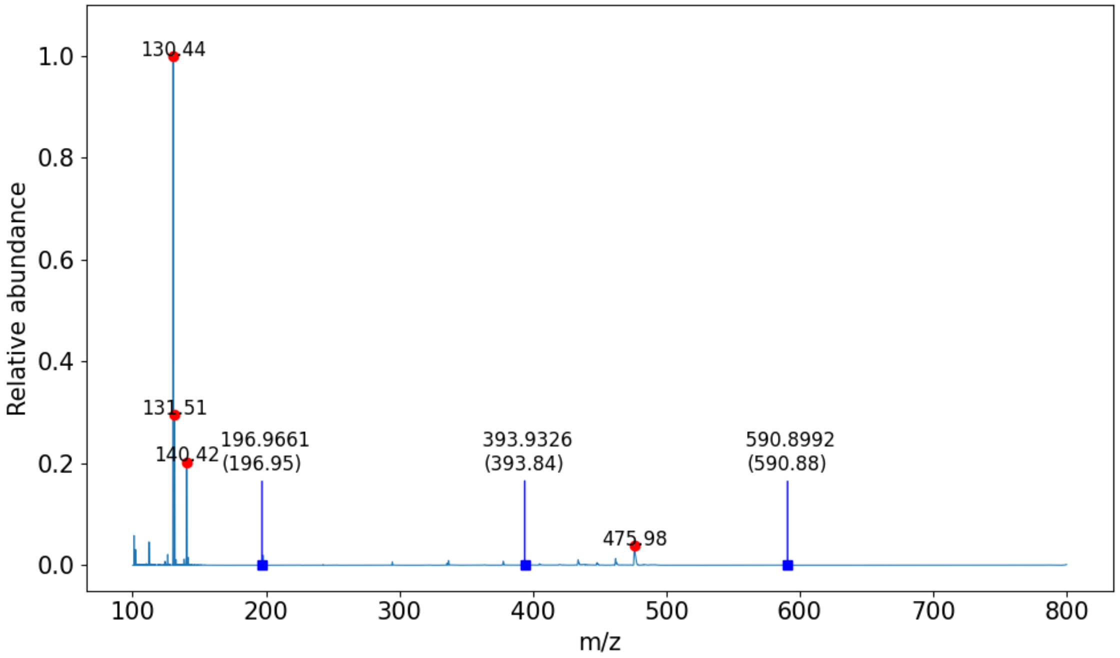

Figure S44. LDI spectrum of a gold-substrates functionalised with **13a** using optimised conditions for MIC after 2h.

S7 NMR Studies

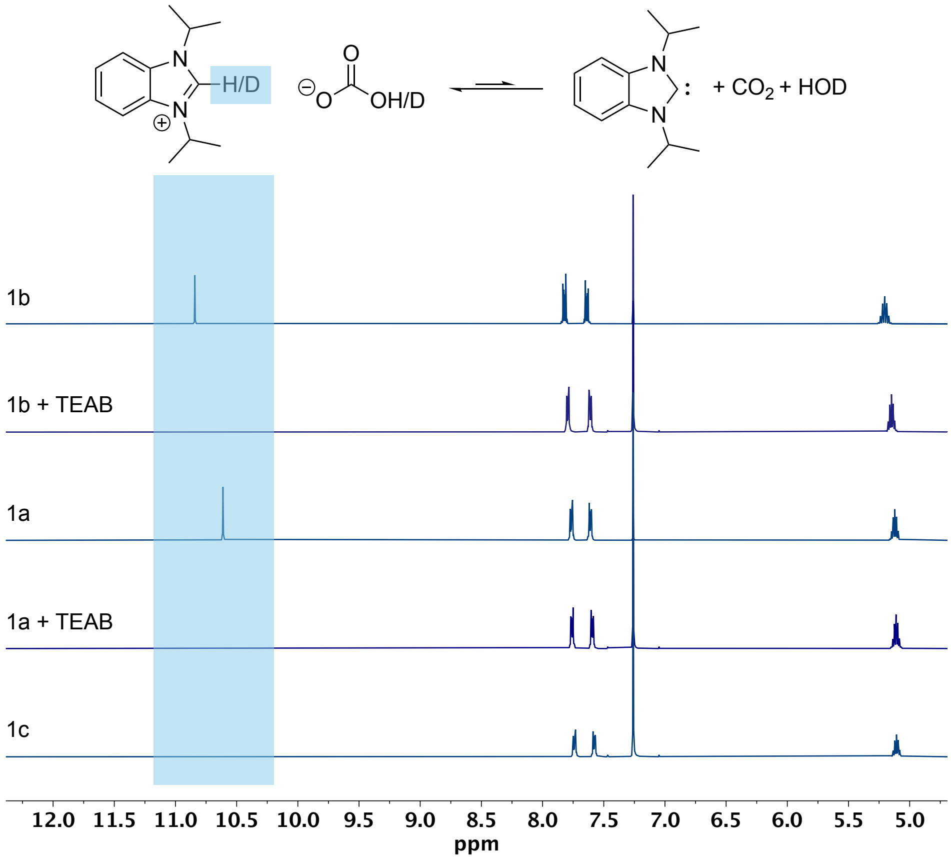

Figure S45. Zoomed ^{1}H NMR stack spectra showing the influence of bicarbonate from either **1c** or from an external source (TEAB) on the acidic proton of **1** in $CDCl_3$.

S8 XPS Analysis

Note: Area is given is CPS•eV. The area values given for the *N* 1s spectra are only for the chemisorbed carbene peak at *ca.* 400 eV, and are the ones utilised to calculate the $N_{(\sim 400\ eV)}/Au$ ratio.

Table S12. Au 4f and N 1s XPS spectra of monolayers of **2c** formed under optimised conditions (5 mM, MeCN, 5 Å MS, 2 h, 55 ºC).

<table>
<tr><td></td><td></td><td></td><td colspan="2">Atom</td></tr>
<tr><td></td><td></td><td></td><td>Au 4f</td><td>N 1s</td></tr>
<tr><td rowspan="6">Point</td><td rowspan="2">1</td><td>Fit</td><td>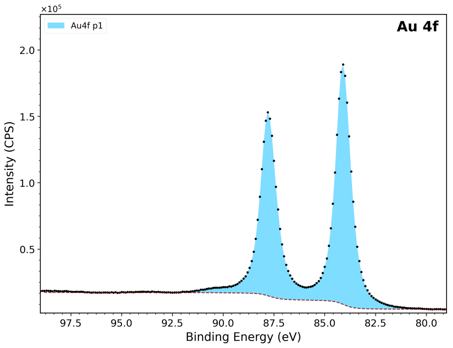
</td><td>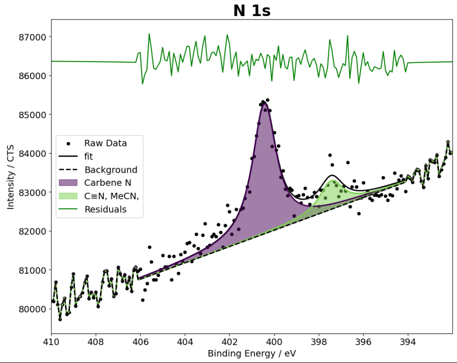
</td></tr>
<tr><td>Area</td><td>365404</td><td>6572</td></tr>
<tr><td rowspan="2">2</td><td>Fit</td><td>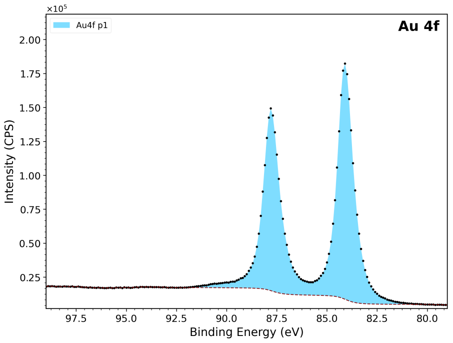
</td><td>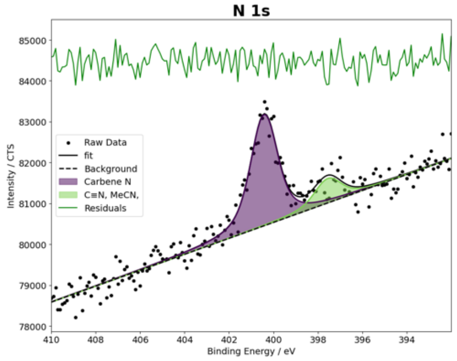
</td></tr>
<tr><td>Area</td><td>363367</td><td>5166</td></tr>
<tr><td rowspan="2">3</td><td>Fit</td><td>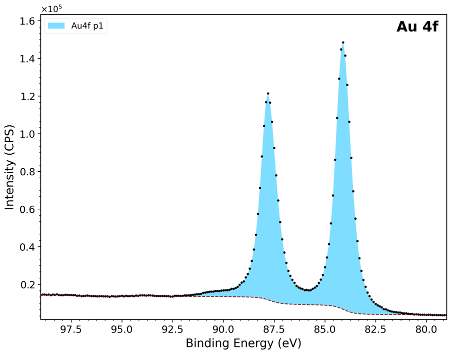
</td><td>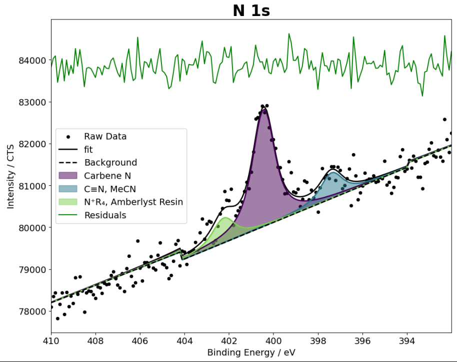
</td></tr>
<tr><td>Area</td><td>292514</td><td>4844</td></tr>
</table>

Table S13. *C* 1s and *I* 3d XPS spectra of monolayers of **2c** formed under optimised conditions (5 mM, MeCN, 5 Å MS, 2 h, 55 ºC).

<table>
<tr><td></td><td></td><td colspan="2">Atom</td></tr>
<tr><td></td><td></td><td>C 1s</td><td>I 3d</td></tr>
<tr><td rowspan="3">Point</td><td>1</td><td>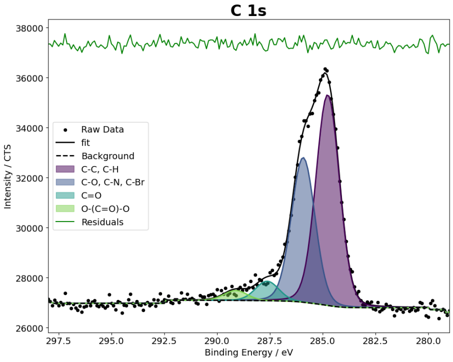
</td><td>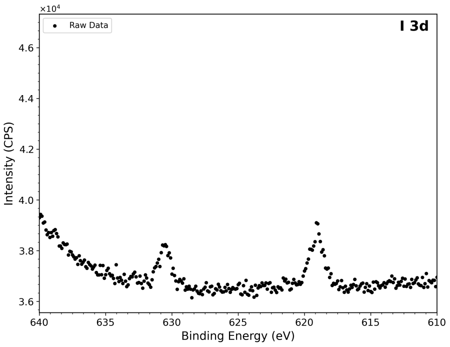
</td></tr>
<tr><td>2</td><td>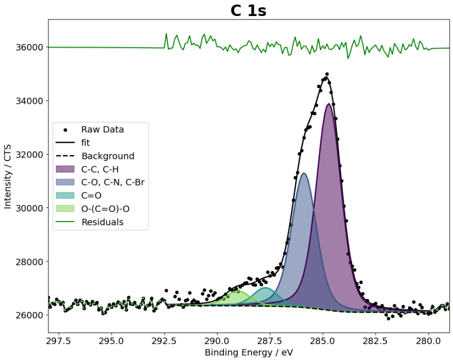
</td><td>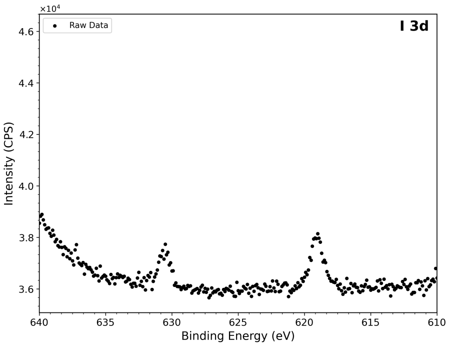
</td></tr>
<tr><td>3</td><td>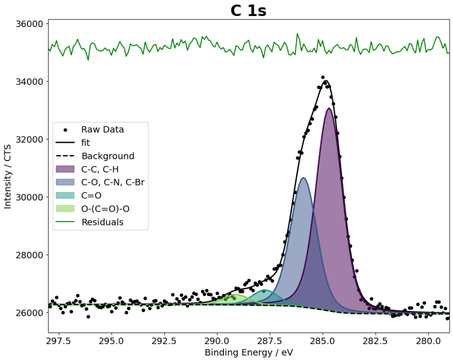
</td><td>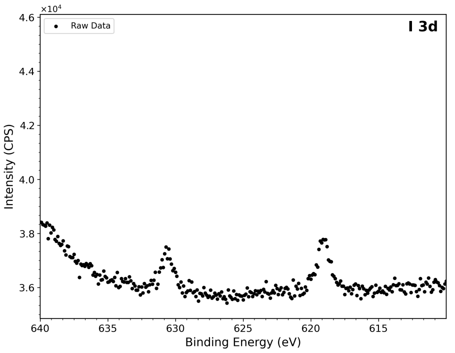
</td></tr>
</table>

Table S14. *Au* 4f and *N* 1s XPS spectra of monolayers of **2c** formed under optimised conditions developed by Glorius (5 mM, EtOH, 2 h, 55 ºC).[16]

<table>
<tr><td colspan="3"></td><td colspan="2">Atom</td></tr>
<tr><td colspan="3"></td><td>Au 4f</td><td>N 1s</td></tr>
<tr><td rowspan="6">Point</td><td rowspan="2">1</td><td>Fit</td><td>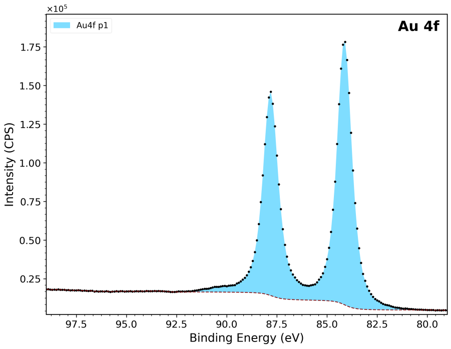
</td><td>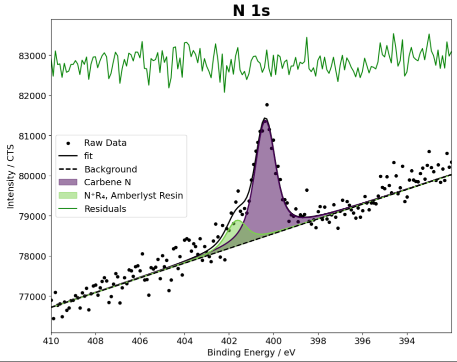
</td></tr>
<tr><td>Area</td><td>348712</td><td>4264</td></tr>
<tr><td rowspan="2">2</td><td>Fit</td><td>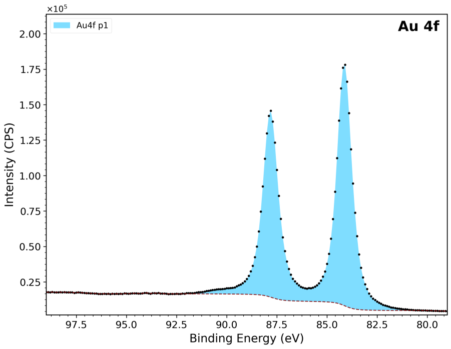
</td><td>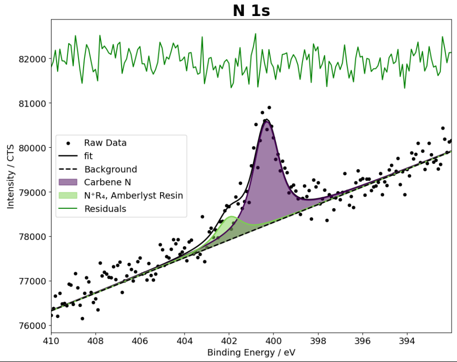
</td></tr>
<tr><td>Area</td><td>346171</td><td>4428</td></tr>
<tr><td rowspan="2">3</td><td>Fit</td><td>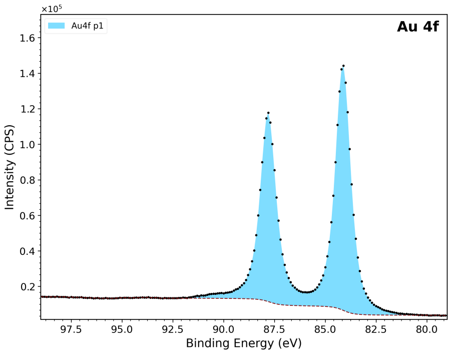
</td><td>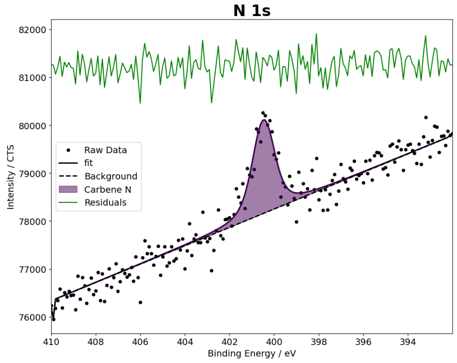
</td></tr>
<tr><td>Area</td><td>281756</td><td>2991</td></tr>
</table>

Table S15. *C* 1s and *I* 3d XPS spectra of monolayers of **2c** formed under optimised conditions developed by Glorius (5 mM, EtOH, 2 h, 55 ºC).[16]

		Atom	
		C 1s	*I* 3d
Point	1	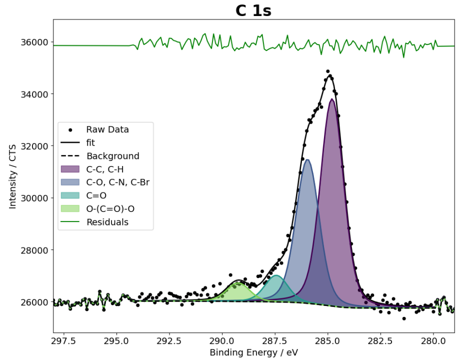	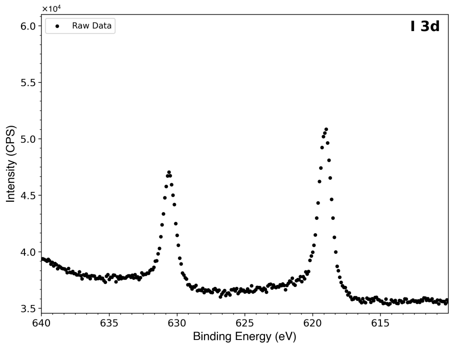
	2	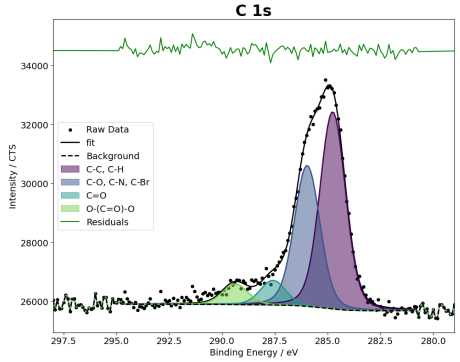	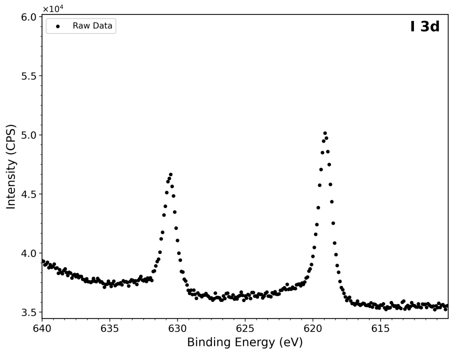
	3	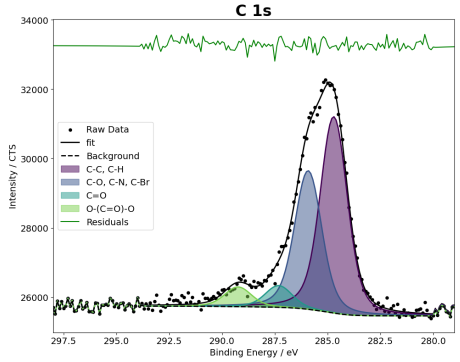	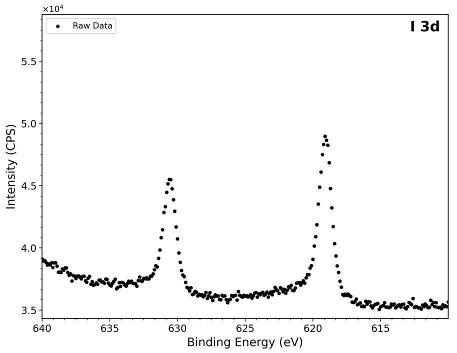

Table S16. *Au* 4f and *N* 1s XPS spectra of monolayers of **2c** formed under optimised conditions minus the 5 Å MS (5 mM, MeCN, 2 h, 55 ºC).

<table>
<tr><td colspan="3"></td><td colspan="2">Atom</td></tr>
<tr><td colspan="3"></td><td>Au 4f</td><td>N 1s</td></tr>
<tr><td rowspan="6">Point</td><td rowspan="2">1</td><td>Fit</td><td>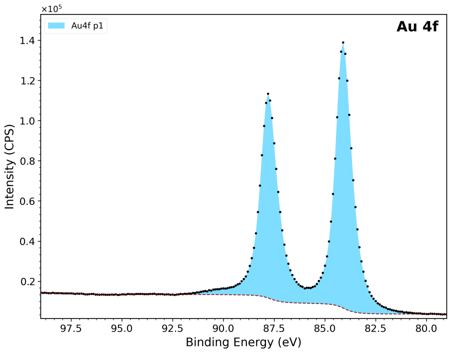
</td><td>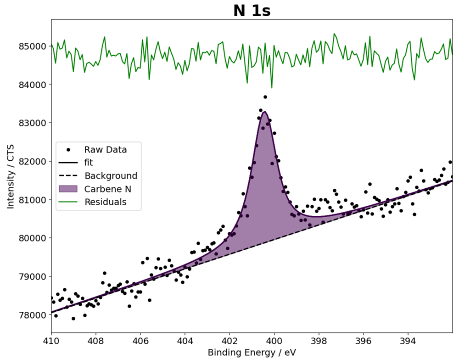
</td></tr>
<tr><td>Area</td><td>283177</td><td>7000</td></tr>
<tr><td rowspan="2">2</td><td>Fit</td><td>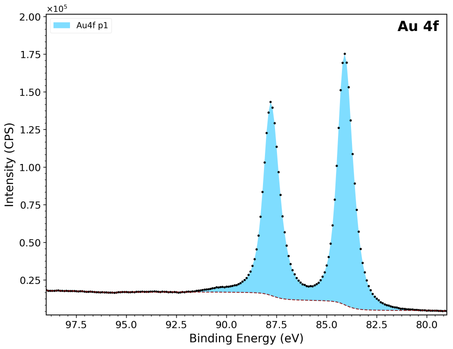
</td><td>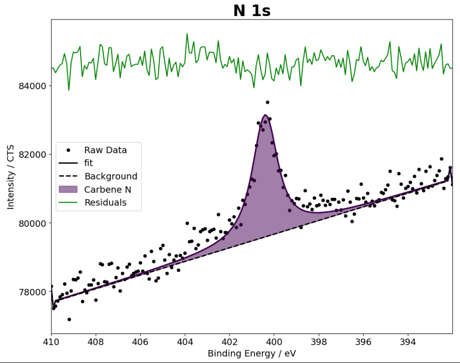
</td></tr>
<tr><td>Area</td><td>352874</td><td>7286</td></tr>
<tr><td rowspan="2">3</td><td>Fit</td><td>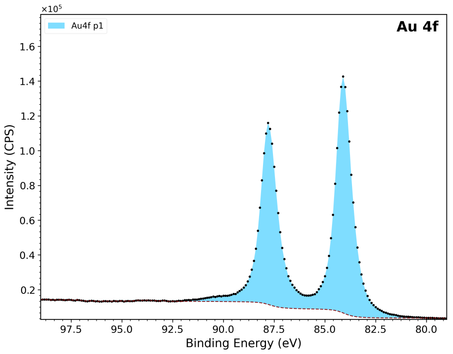
</td><td>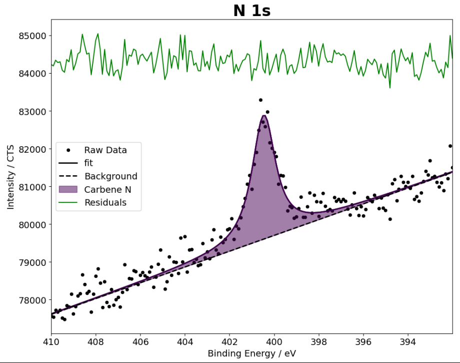
</td></tr>
<tr><td>Area</td><td>285144</td><td>6430</td></tr>
</table>

Table S17 C 1s and *I* 3d XPS spectra of monolayers of **2c** formed under optimised conditions minus the 5 Å MS (5 mM, MeCN, 2 h, 55 ºC).

<table>
<tr><td></td><td></td><td colspan="2">Atom</td></tr>
<tr><td></td><td></td><td>C 1s</td><td>I 3d</td></tr>
<tr><td rowspan="3">Point</td><td>1</td><td>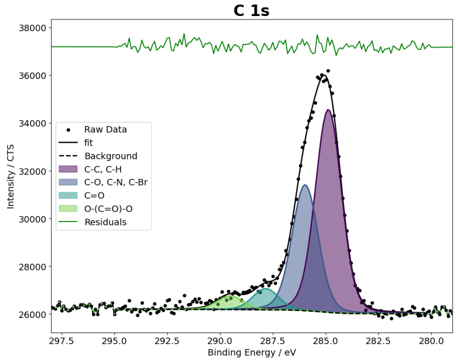
</td><td>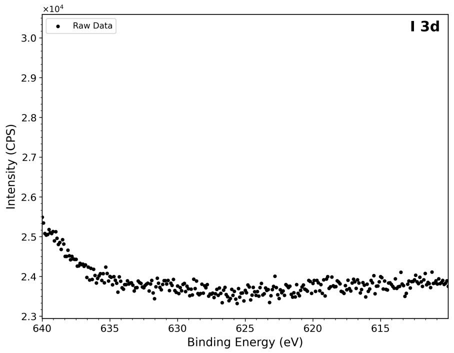
</td></tr>
<tr><td>2</td><td>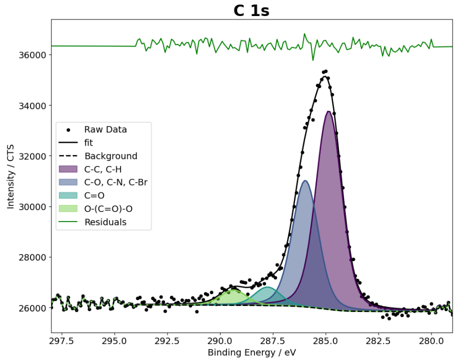
</td><td>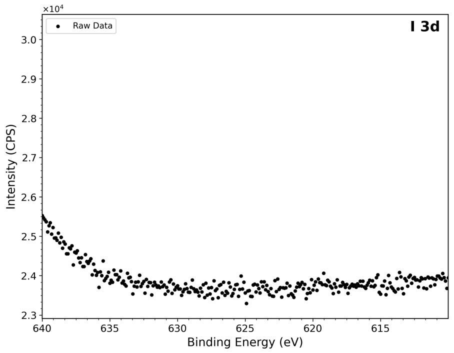
</td></tr>
<tr><td>3</td><td>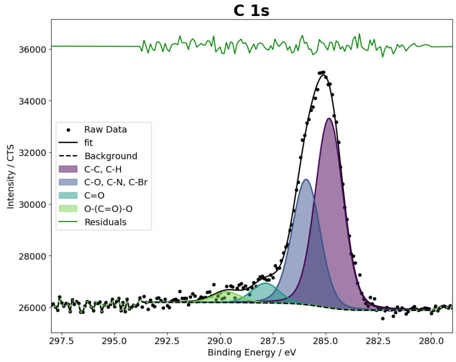
</td><td>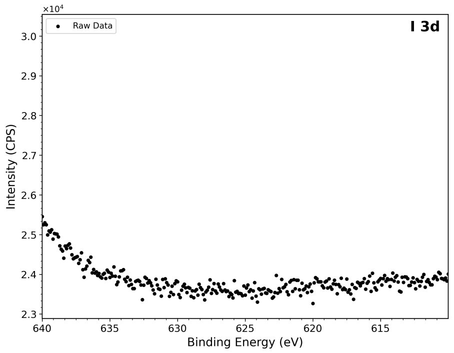
</td></tr>
</table>

Table S18. *Au* 4f and *N* 1s XPS spectra of monolayers of **2c** formed under methanolic room temperature deposition (1 mM, MeOH, 24 h, RT).

<table>
<tr><td colspan="3"></td><td colspan="2">Atom</td></tr>
<tr><td colspan="3"></td><td>Au 4f</td><td>N 1s</td></tr>
<tr><td rowspan="6">Point</td><td rowspan="2">1</td><td>Fit</td><td>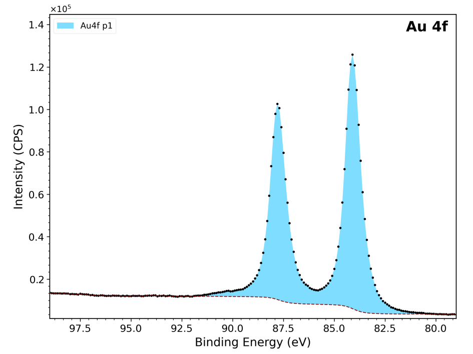
</td><td>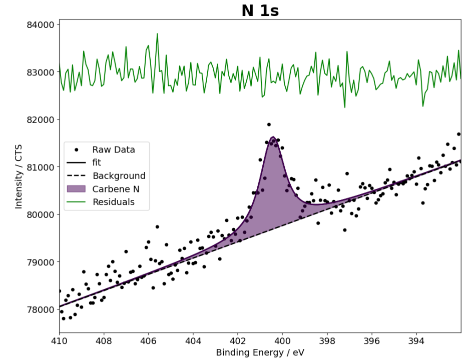
</td></tr>
<tr><td>Area</td><td>249799</td><td>3931</td></tr>
<tr><td rowspan="2">2</td><td>Fit</td><td>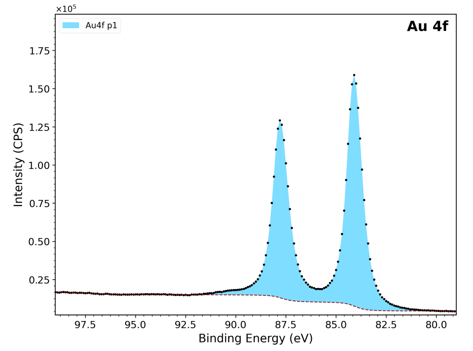
</td><td>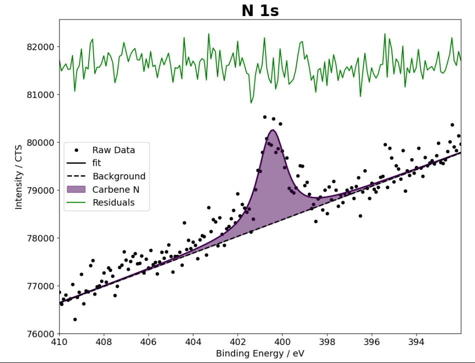
</td></tr>
<tr><td>Area</td><td>314659</td><td>4194</td></tr>
<tr><td rowspan="2">3</td><td>Fit</td><td>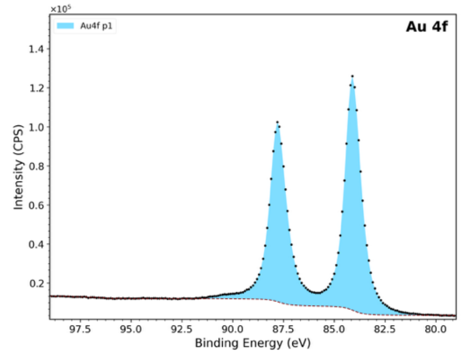
</td><td>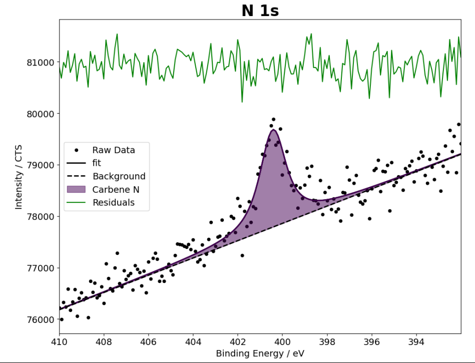
</td></tr>
<tr><td>Area</td><td>251156</td><td>4003</td></tr>
</table>

Table S19. *C* 1s and *I* 3d XPS spectra of monolayers of **2c** formed methanolic room temperature deposition (1 mM, MeOH, 24 h, RT).

		Atom	
		C 1s	*I* 3d
Point	1	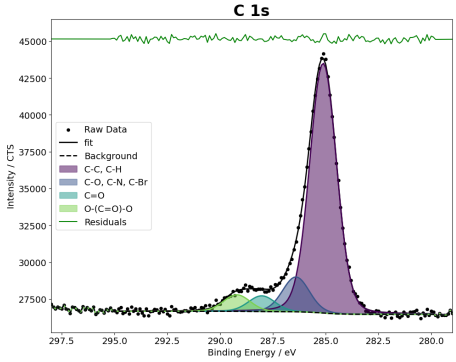	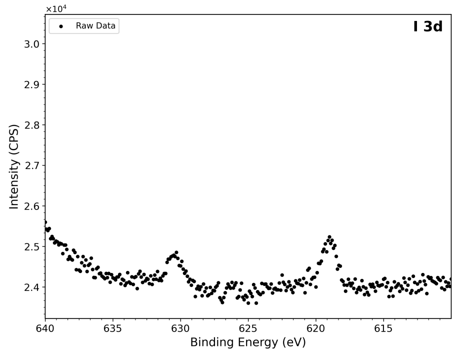
	2	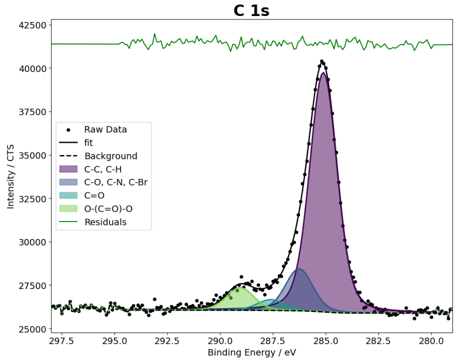	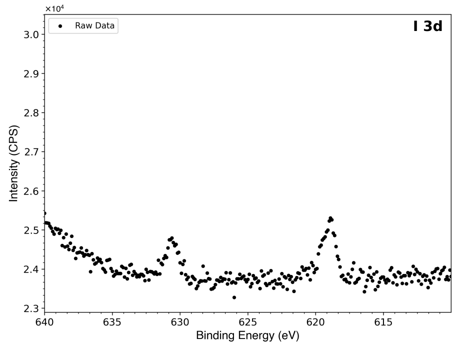
	3	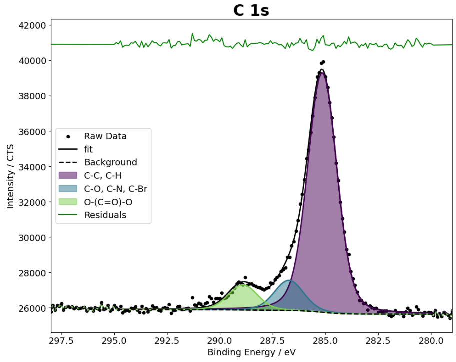	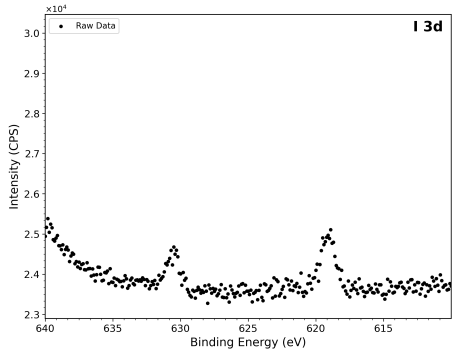

Table S20. *Au* 4f and *N* 1s XPS spectra of monolayers of **2a** formed under optimised conditions, minus the TEAB and 5 Å MS (1 mM, THF, 2 h, 55 ºC).

<table>
<tr><td></td><td></td><td></td><td colspan="2">Atom</td></tr>
<tr><td></td><td></td><td></td><td>Au 4f</td><td>N 1s</td></tr>
<tr><td rowspan="6">Point</td><td rowspan="2">1</td><td>Fit</td><td>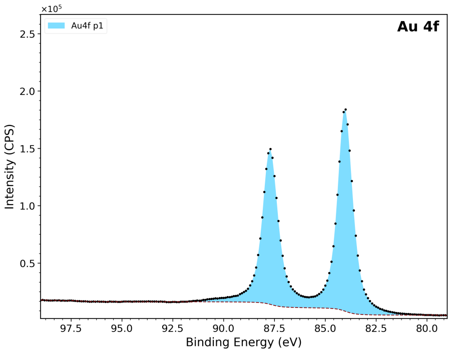
</td><td>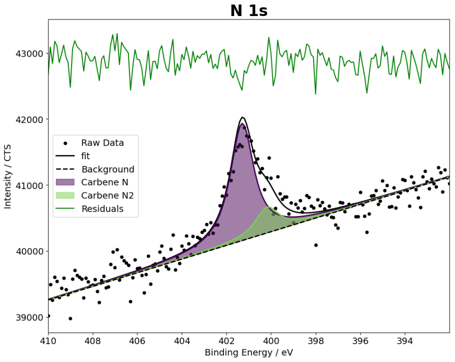
</td></tr>
<tr><td>Area</td><td>347280</td><td>809</td></tr>
<tr><td rowspan="2">2</td><td>Fit</td><td>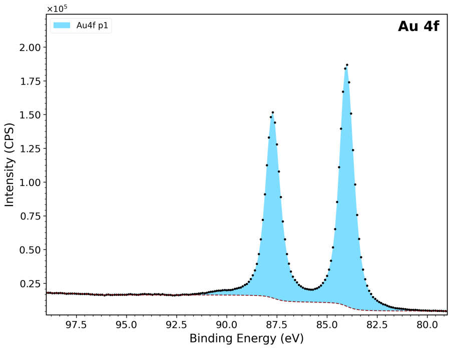
</td><td>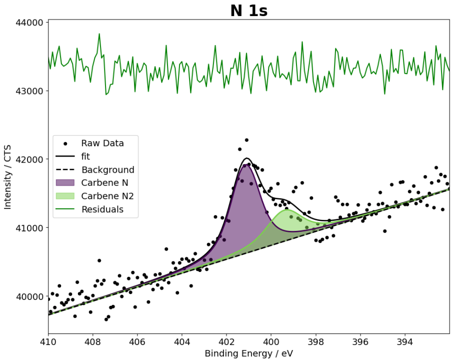
</td></tr>
<tr><td>Area</td><td>353850</td><td>1228</td></tr>
<tr><td rowspan="2">3</td><td>Fit</td><td>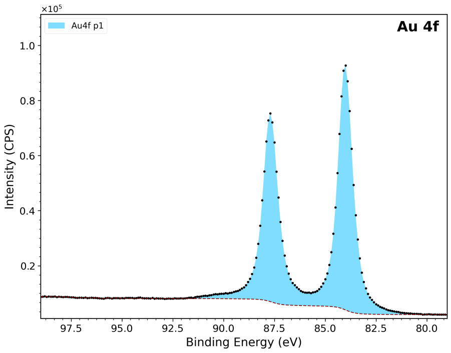
</td><td>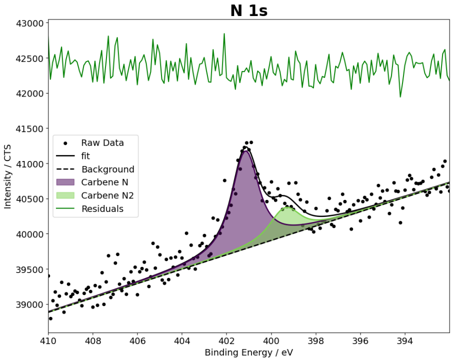
</td></tr>
<tr><td>Area</td><td>175051</td><td>925</td></tr>
</table>

Table S21. *C* 1s and *I* 3d XPS spectra of monolayers of **2a** formed under optimised conditions, minus the TEAB and 5 Å MS (1 mM, THF, 2 h, 55 ºC).

		Atom	
		C 1s	*I* 3d
Point	1	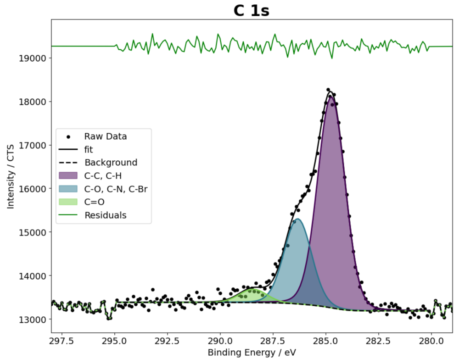	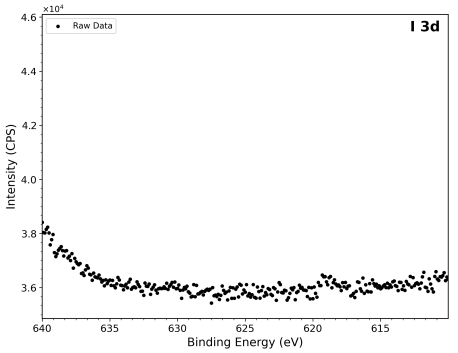
	2	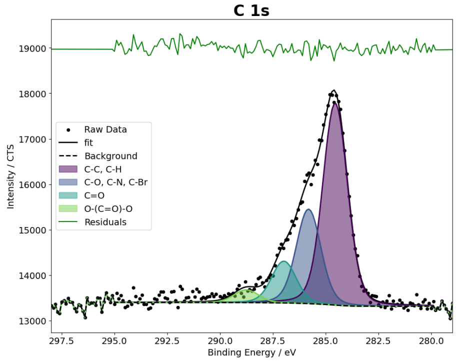	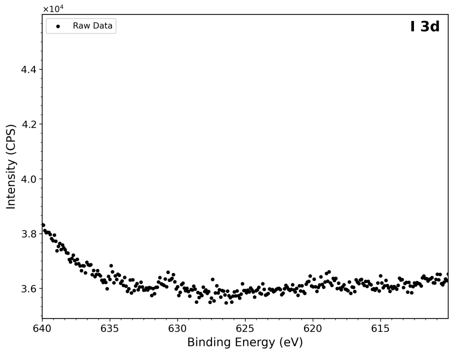
	3	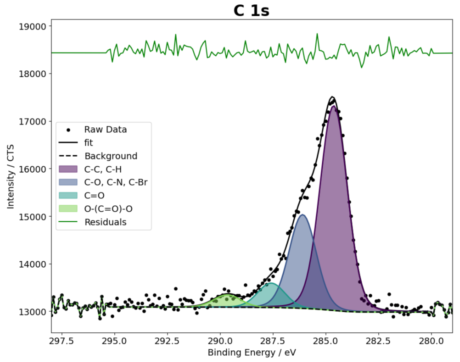	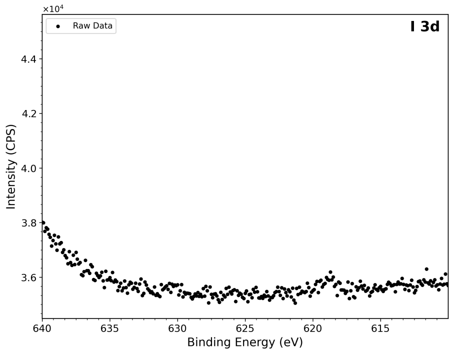

Table S22. *Au* 4f and *N* 1s XPS spectra of monolayers of **2a** formed under optimised conditions, minus the TEAB (1 mM, THF, 5 Å MS, 2 h, 55 ºC).

			Atom	
			Au 4f	*N* 1s
Point	1	Fit	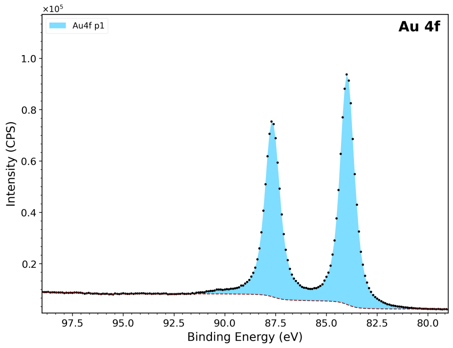	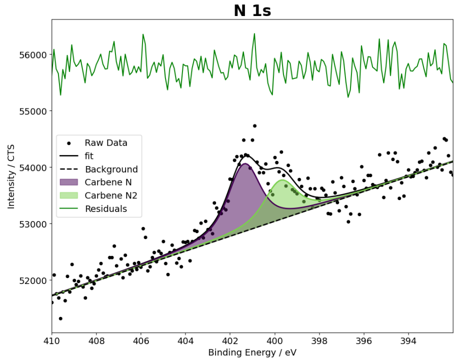
		Area	176080	1695
	2	Fit	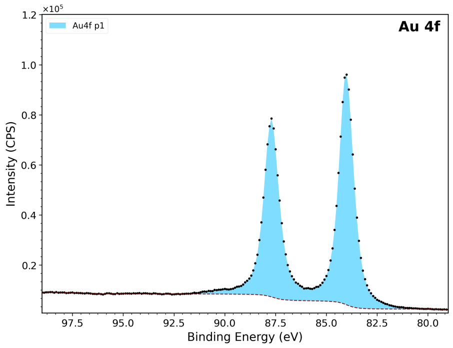	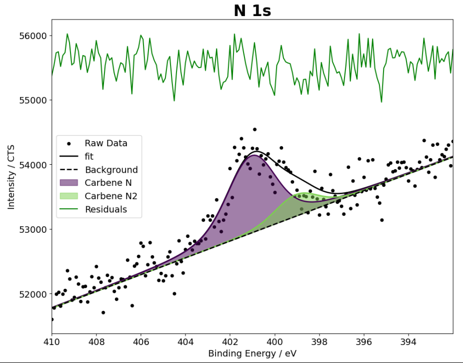
		Area	181779	864
	3	Fit	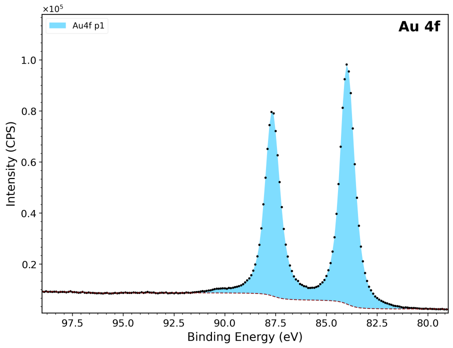	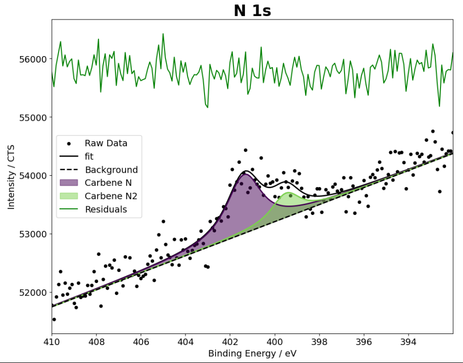
		Area	186242	952

Table S23. *C* 1s and *I* 3d XPS spectra of monolayers of **2a** formed under optimised conditions, minus the TEAB (1 mM, THF, 5 Å MS, 2 h, 55 ºC).

		Atom	
		C 1s	*I* 3d
Point	1	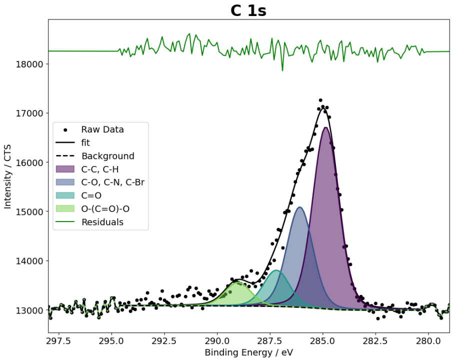	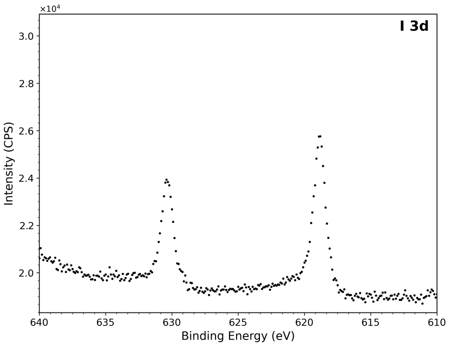
	2	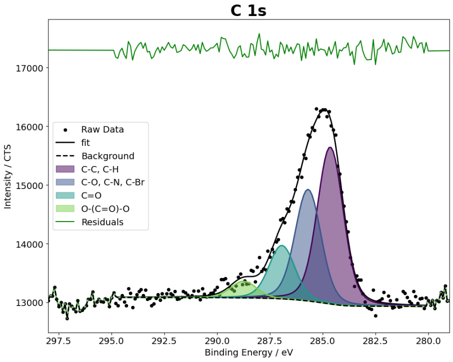	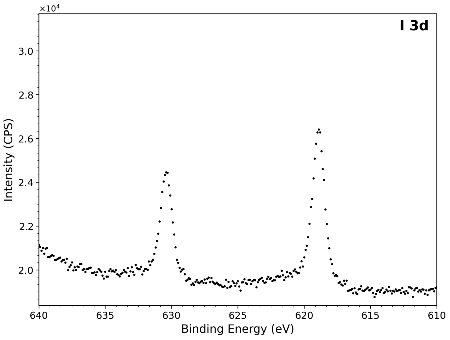
	3	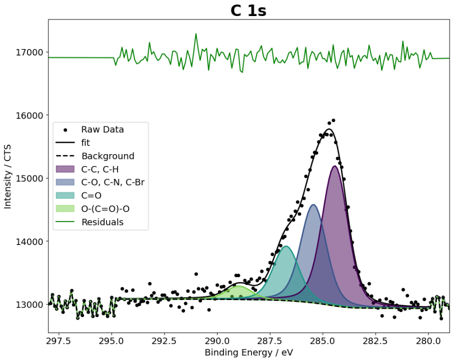	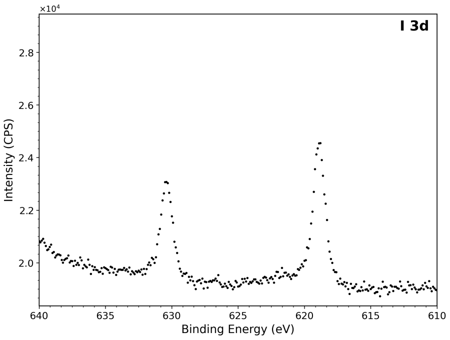

Table S24. *Au* 4f and *N* 1s XPS spectra of monolayers of **2a** formed under optimised conditions, minus the 5 Å MS (1 mM, 5 mM TEAB, THF, 2 h, 55 ºC).

			Atom	
			Au 4f	*N* 1s
Point	1	Fit	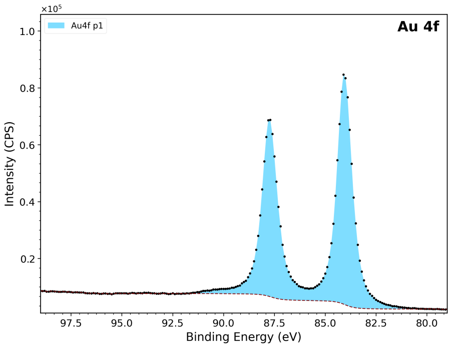	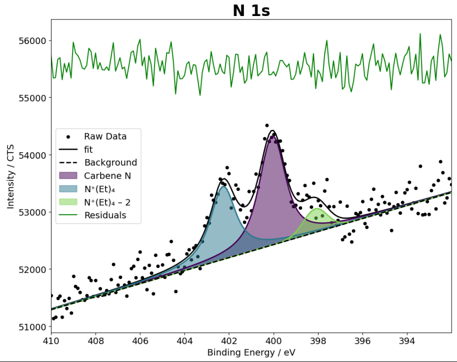
		Area	160754	2562
	2	Fit	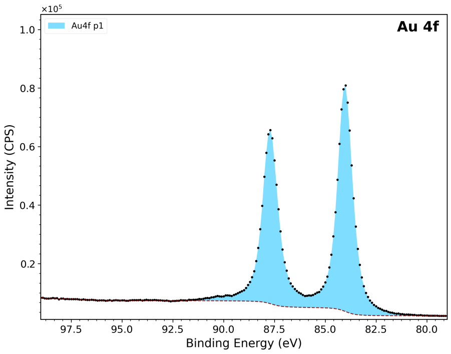	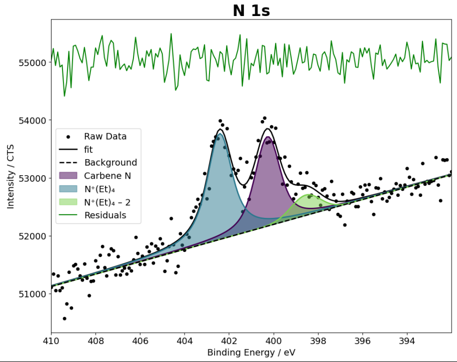
		Area	153337	2770
	3	Fit	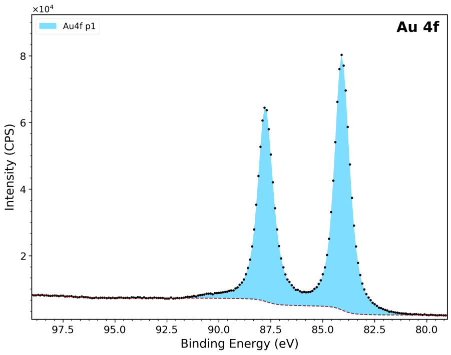	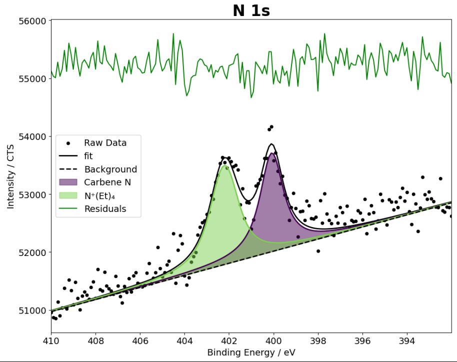
		Area	152123	3348

Table S25. *C* 1s and *I* 3d XPS spectra of monolayers of **2a** formed under optimised conditions, minus the 5 Å MS (1 mM, 5 mM TEAB, THF, 2 h, 55 ºC).

<table>
<tr><td></td><td></td><td colspan="2">Atom</td></tr>
<tr><td></td><td></td><td>C 1s</td><td>I 3d</td></tr>
<tr><td rowspan="3">Point</td><td>1</td><td>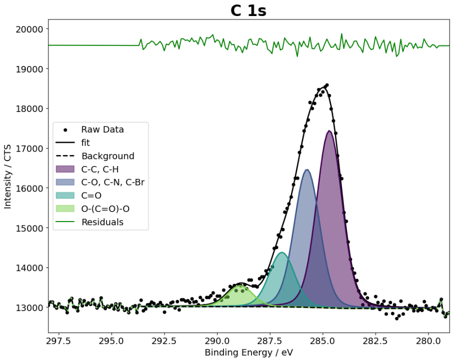</td><td>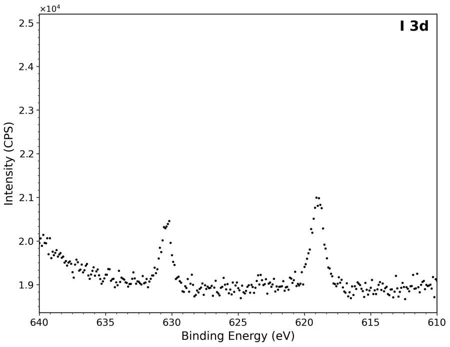</td></tr>
<tr><td>2</td><td>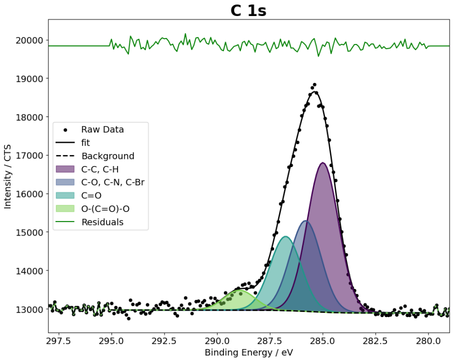</td><td>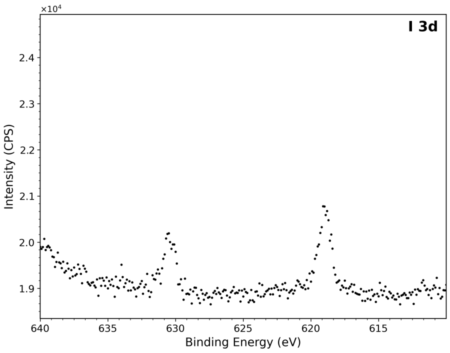</td></tr>
<tr><td>3</td><td>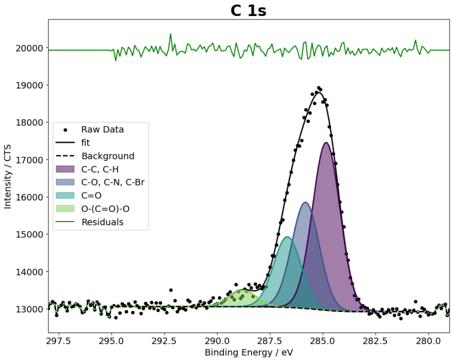</td><td>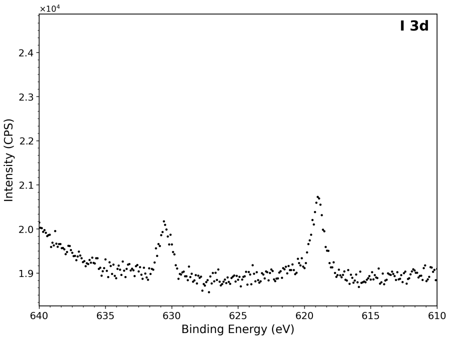</td></tr>
</table>

Table S26. *Au* 4f and *N* 1s XPS spectra of monolayers of **2a** formed under optimised conditions (1 mM, 5 mM TEAB, 5 Å MS, THF, 2 h, 55 ºC).

			Atom	
			Au 4f	*N* 1s
Point	1	Fit	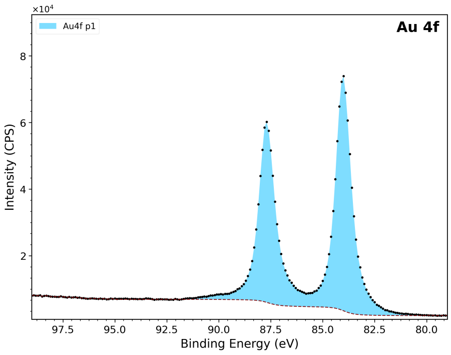	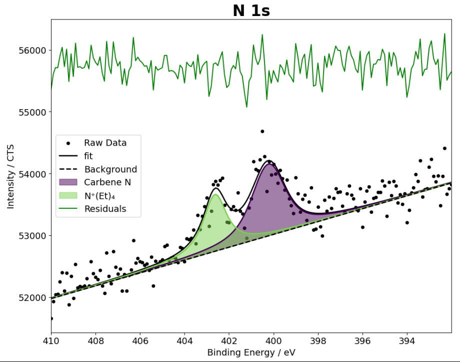
		Area	142743	2905
	2	Fit	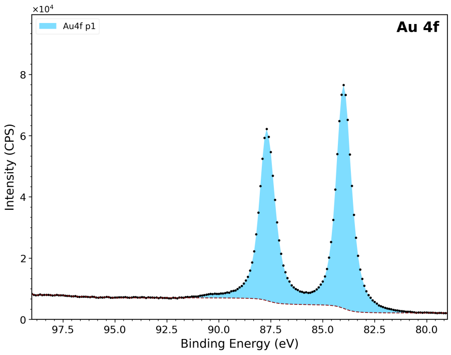	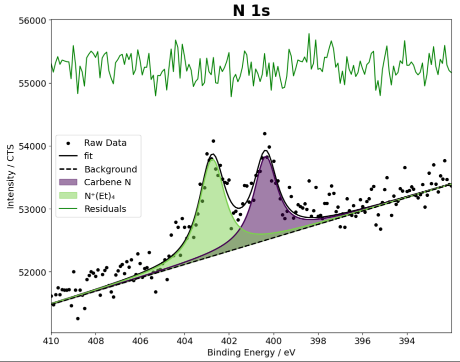
		Area	145291	2612
	3	Fit	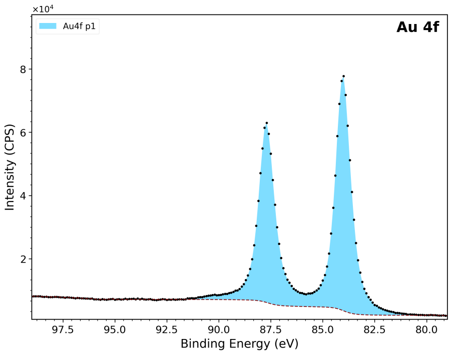	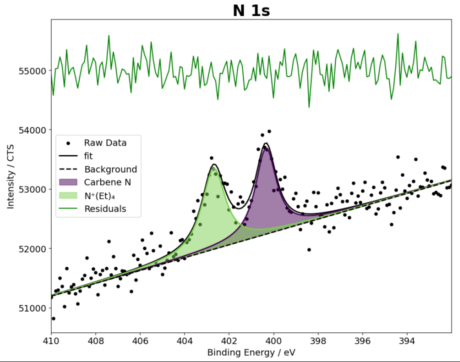
		Area	147355	2494

Table S27. *C* 1s and *I* 3d XPS spectra of monolayers of **2a** formed under optimised conditions (1 mM, 5 mM TEAB, 5 Å MS, THF, 2 h, 55 ºC).

<table>
<tr><td></td><td></td><td colspan="2">Atom</td></tr>
<tr><td></td><td></td><td>C 1s</td><td>I 3d</td></tr>
<tr><td rowspan="3">Point</td><td>1</td><td>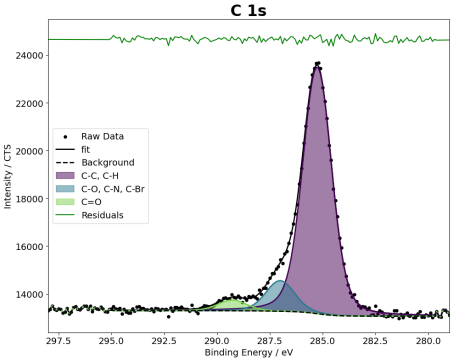
</td><td>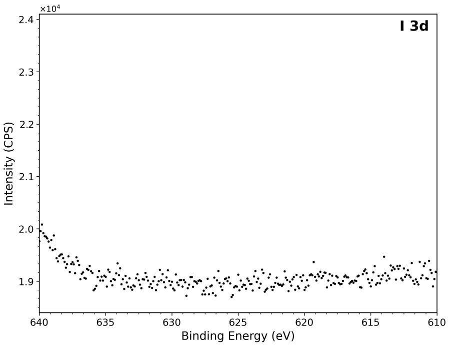
</td></tr>
<tr><td>2</td><td>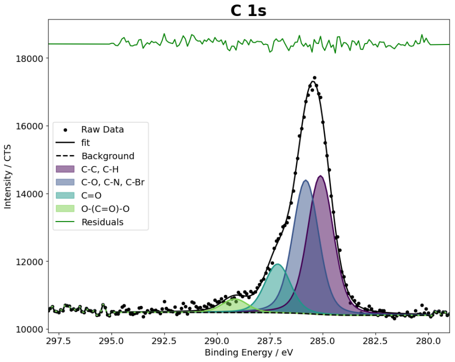
</td><td>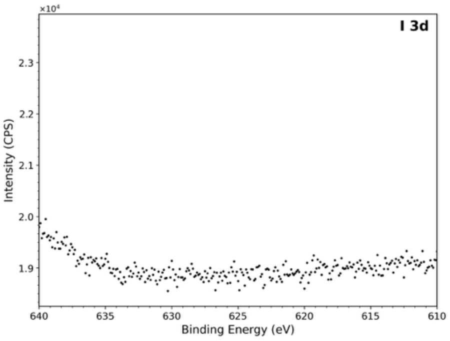
</td></tr>
<tr><td>3</td><td>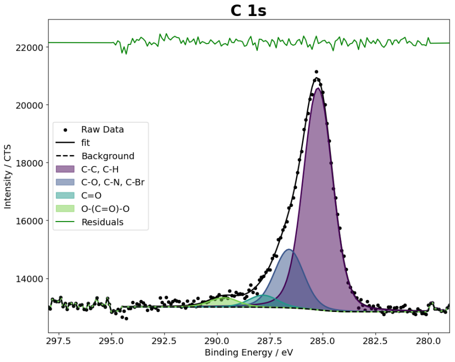
</td><td>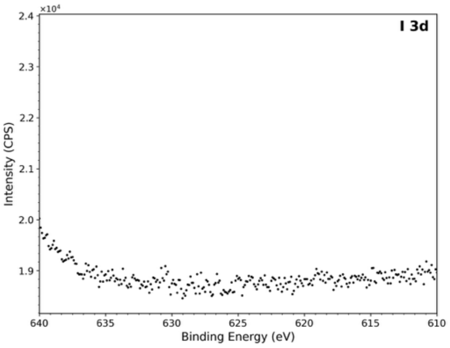
</td></tr>
</table>

Table S28. *Au* 4f and *N* 1s XPS spectra of monolayers of **2b** formed under optimised conditions (1 mM, 5 mM TEAB, 5 Å MS, THF, 2 h, 55 ºC).

			Atom	
			Au 4f	*N* 1s
Point	1	Fit	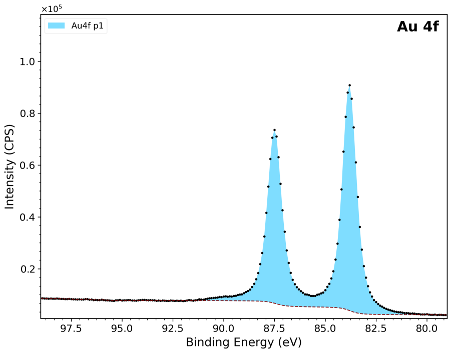	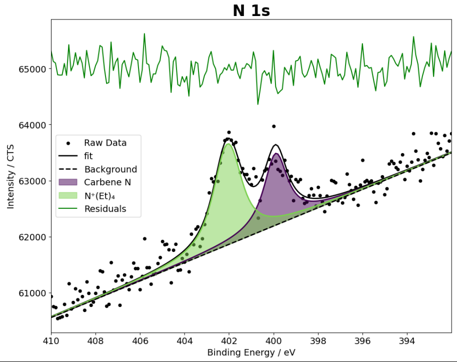
		Area	166627	2517
	2	Fit	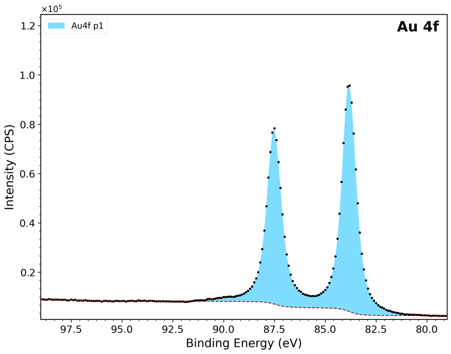	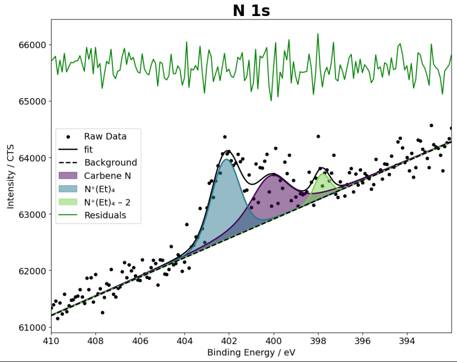
		Area	177952	2361
	3	Fit	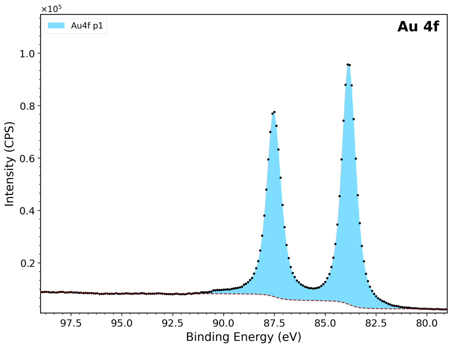	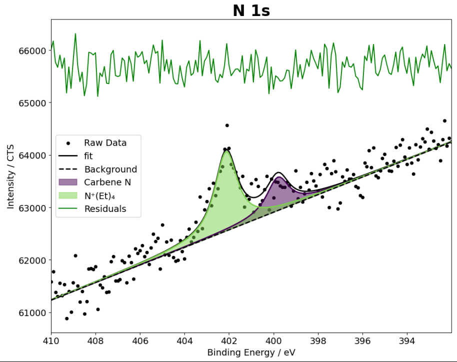
		Area	177242	1188

Table S29. *C* 1s and *I* 3d XPS spectra of monolayers of **2b** formed under optimised conditions (1 mM, 5 mM TEAB, 5 Å MS, THF, 2 h, 55 ºC).

		Atom	
		C 1s	*I* 3d
Point	1	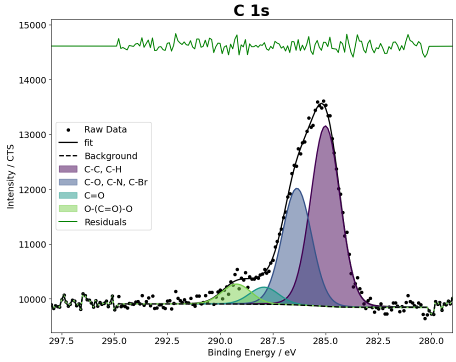	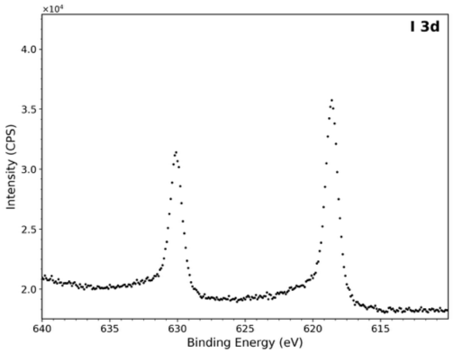
	2	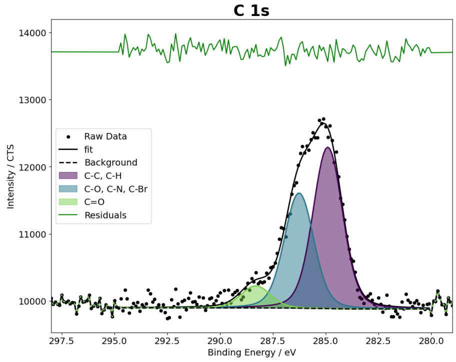	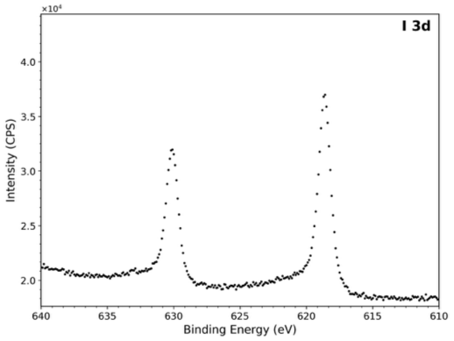
	3	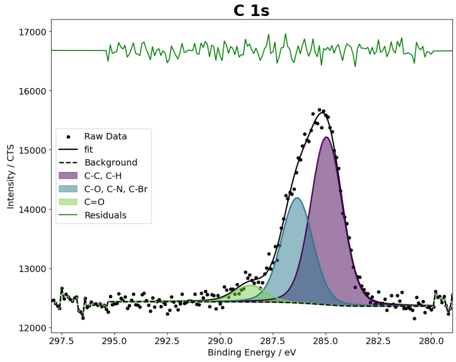	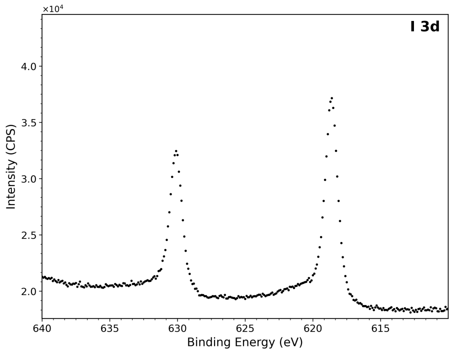

Table 30. *Au* 4f and *N* 1s XPS spectra of monolayers of **2a** formed under optimised conditions (1 mM, 5 mM TEAB, 5 Å MS, THF, 2 h), at room temperature.

<table>
<tr><td colspan="3"></td><td colspan="2">Atom</td></tr>
<tr><td colspan="3"></td><td>Au 4f</td><td>N 1s</td></tr>
<tr><td rowspan="6">Point</td><td rowspan="2">1</td><td>Fit</td><td>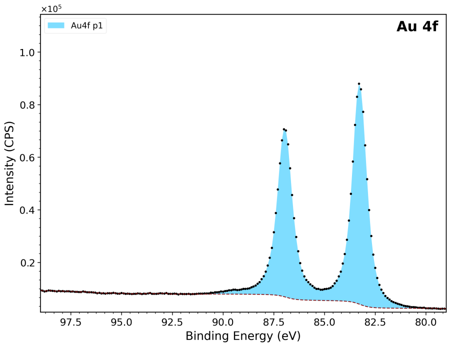
</td><td>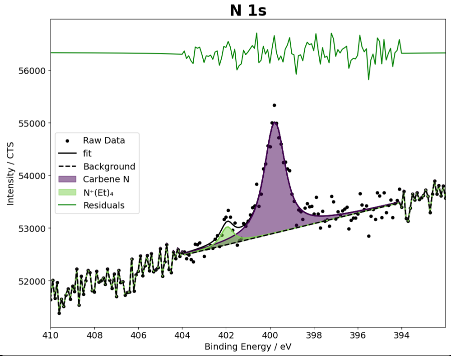
</td></tr>
<tr><td>Area</td><td>164352</td><td>3471</td></tr>
<tr><td rowspan="2">2</td><td>Fit</td><td>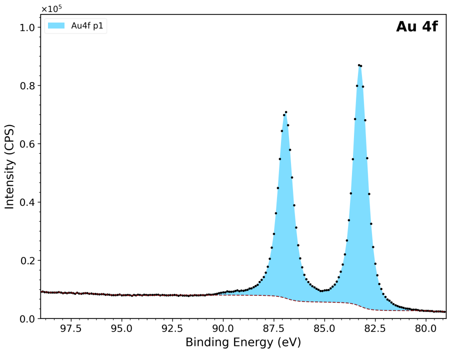
</td><td>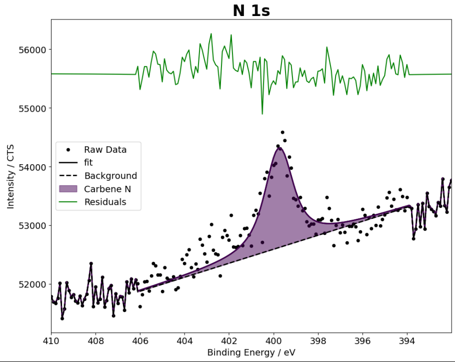
</td></tr>
<tr><td>Area</td><td>161474</td><td>3790</td></tr>
<tr><td rowspan="2">3</td><td>Fit</td><td>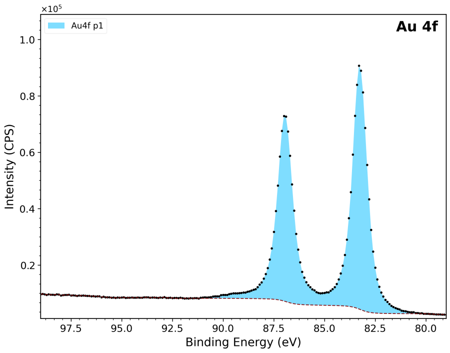
</td><td>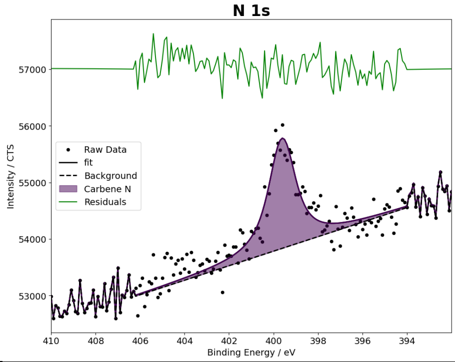
</td></tr>
<tr><td>Area</td><td>169666</td><td>4189</td></tr>
</table>

Table S31. *C* 1s and *I* 3d XPS spectra of monolayers of **2b** formed under optimised conditions (1 mM, 5 mM TEAB, 5 Å MS, THF, 2 h, 55 ºC).

<table>
<tr><td></td><td></td><td colspan="2">Atom</td></tr>
<tr><td></td><td></td><td>C 1s</td><td>I 3d</td></tr>
<tr><td rowspan="3">Point</td><td>1</td><td>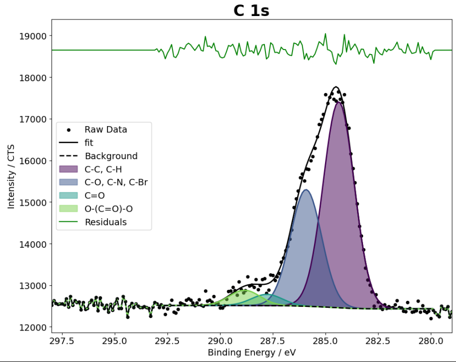
</td><td>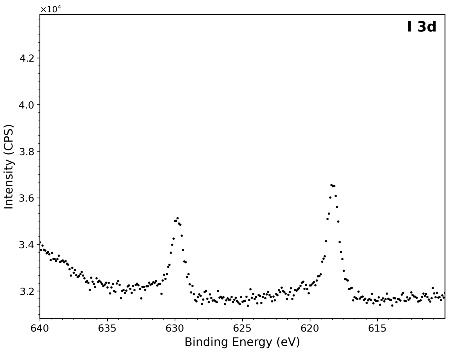
</td></tr>
<tr><td>2</td><td>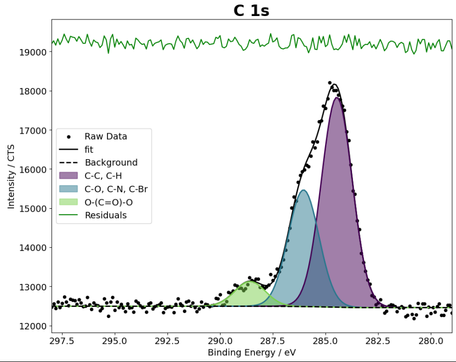
</td><td>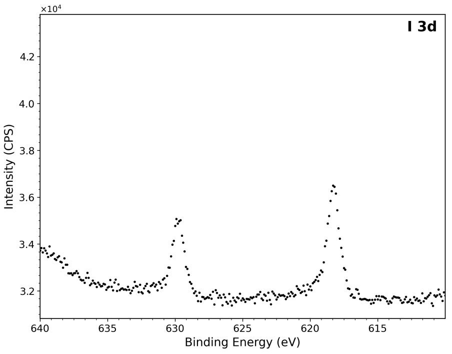
</td></tr>
<tr><td>3</td><td>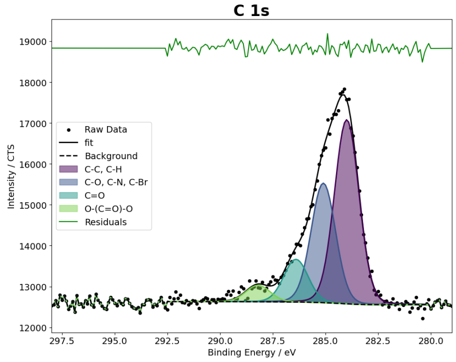
</td><td>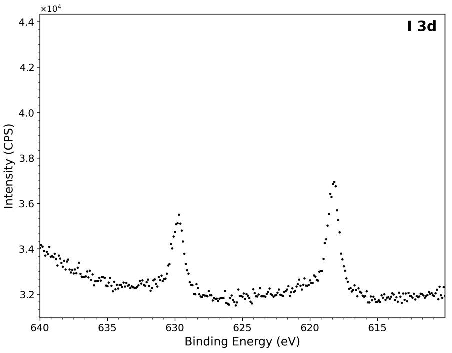
</td></tr>
</table>

Table 32. *Au* 4f and *N* 1s XPS spectra of monolayers of **2a** formed using electrochemical deposition.[10]

			Atom	
			Au 4f	*N* 1s
Point	1	Fit	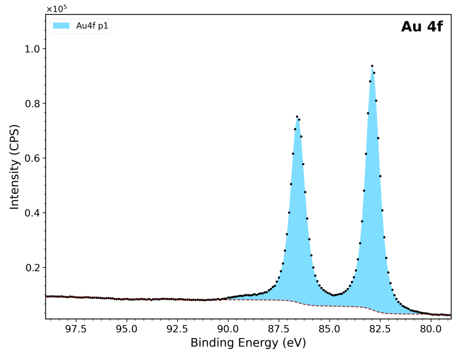	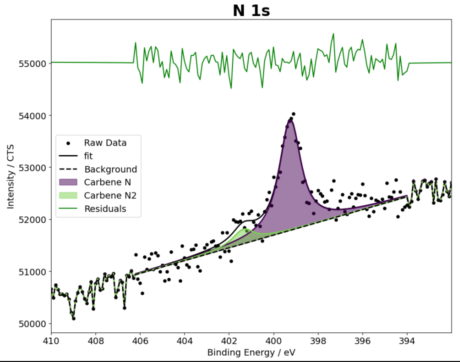
		Area	169357	3776
	2	Fit	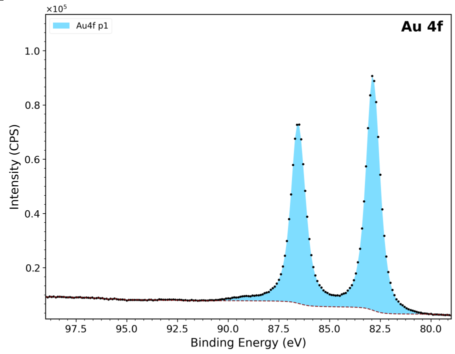	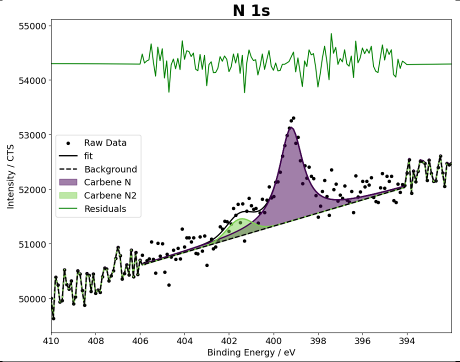
		Area	165766	3369
	3	Fit	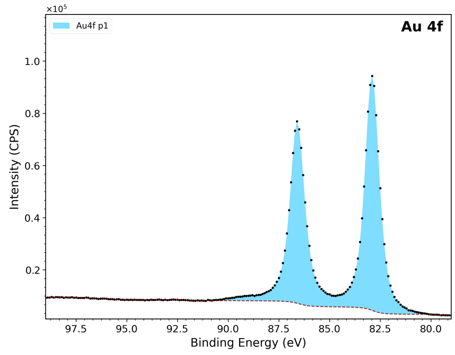	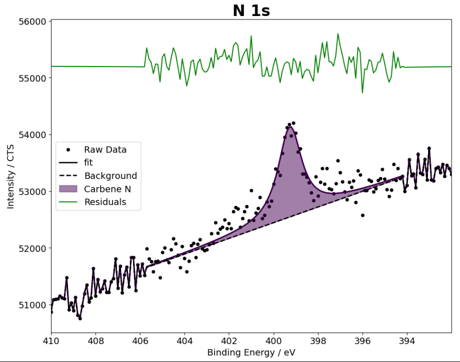
		Area	172859	3127

Table S33. *C* 1s and *I* 3d XPS spectra of monolayers of **2b** formed using electrochemical deposition.[10]

<table>
<tr><td></td><td></td><td colspan="2">Atom</td></tr>
<tr><td></td><td></td><td>C 1s</td><td>I 3d</td></tr>
<tr><td rowspan="3">Point</td><td>1</td><td>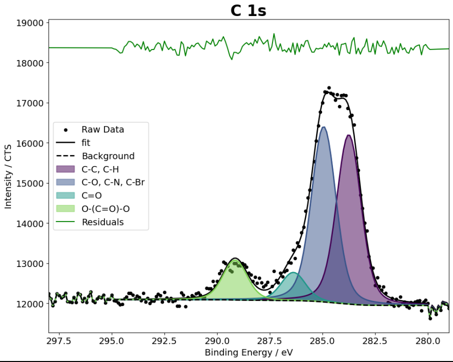
</td><td>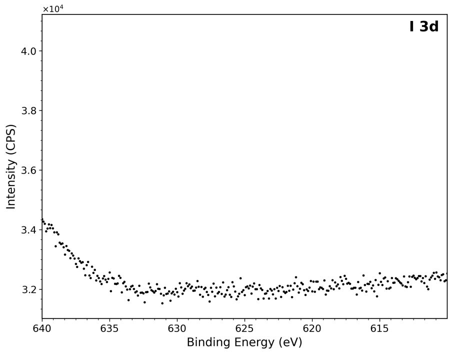
</td></tr>
<tr><td>2</td><td>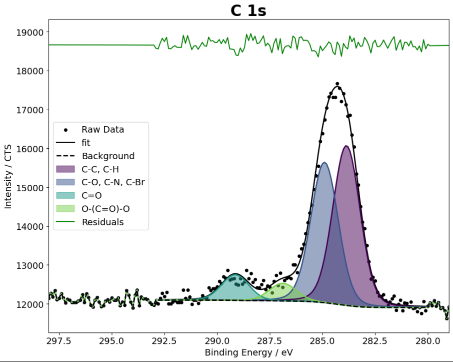
</td><td>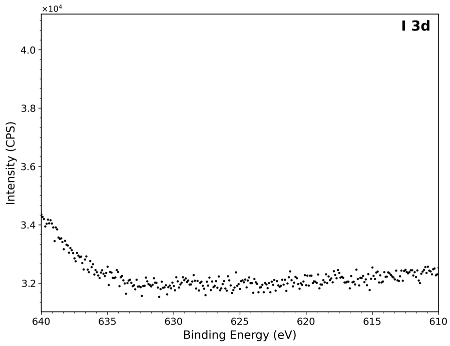
</td></tr>
<tr><td>3</td><td>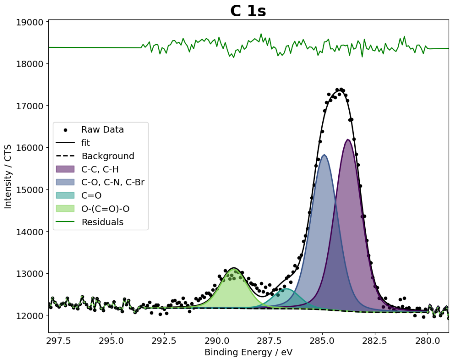
</td><td>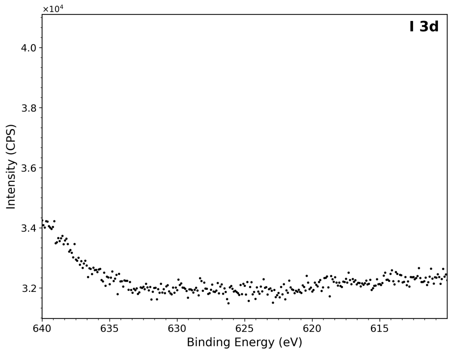
</td></tr>
</table>

Table S34. Summary of $N_{(\sim 400\ eV)}/Au$ ratio for each condition as tested by XPS.

Entry	Compound	[x] / mM	[TEAB] / mM	Temperature / °C	Additive	Solvent	*N/Au* ratio
1	**2c**	5	0	55	5 Å MS	MeCN	0.0174 ± 0.0017
2	**2c**	5	0	55	N/A	EtOH	0.0106 ± 0.0013
3	**2c**	5	0	55	N/A	MeCN	0.0226 ± 0.0012
4	**2c**	5	0	RT	N/A	MeOH	0.0150 ± 0.0008
5	**2a**	1	0	55	N/A	THF	0.0037± 0.0009
6	**2a**	1	0	55	5 Å MS	THF	0.0065 ± 0.0016
7	**2a**	1	5	55	N/A	THF	0.0187 ± 0.0018
8	**2a**	1	5	55	5 Å MS	THF	0.0183 ± 0.0010
9	**2b**	1	5	55	5 Å MS	THF	0.0117 ± 0.0026
10	**2a**	1	5	RT	5 Å MS	THF	0.0231± 0.0010
11*	**2a**	5	0	RT	$TBABF_4$ 0.1 M	MeCN + H_2O 50 mM	0.0202 ± 0.0012

Note: entry 11 corresponds to the electrochemical deposition, see above for experimental details and set up

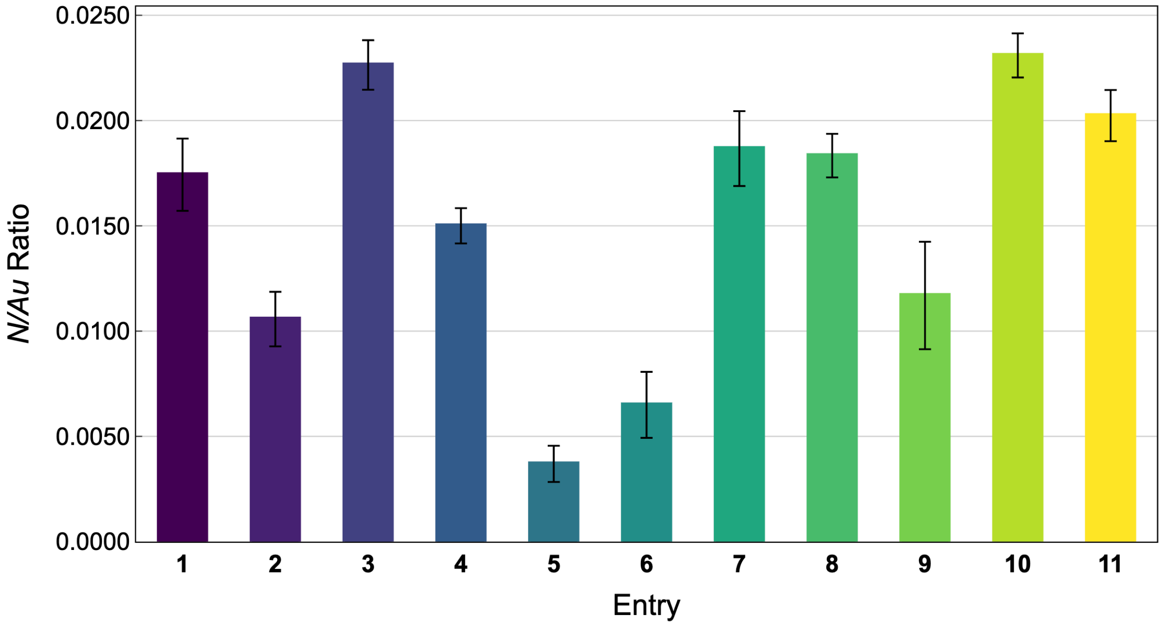

Figure S46. Bar chart showing the calculated $N_{(\sim 400\ eV)}/Au$ ratio as determined by XPS for each entry in **Table S34**, N = 1, n = 3.

Table 35. *Au* 4f and *N* 1s XPS spectra of monolayers of **5a** formed under optimised conditions (1 mM, 5 mM TEAB, 5 Å MS, THF, 2 h, 55 ºC).

			Atom	
			Au 4f	*N* 1s
Point	1	Fit	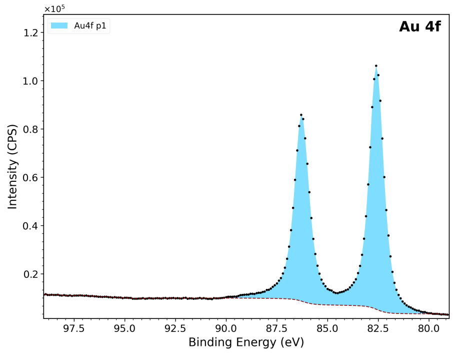	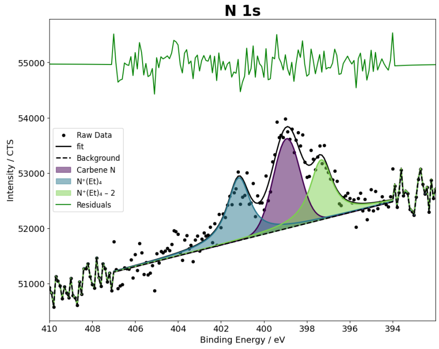
		Area	195212	2553
	2	Fit	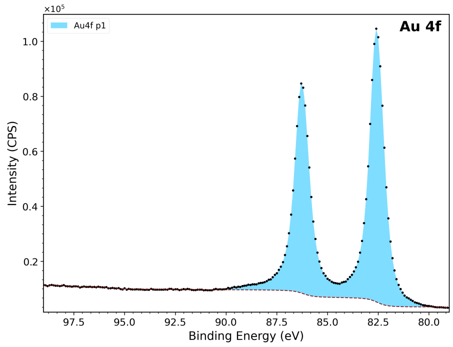	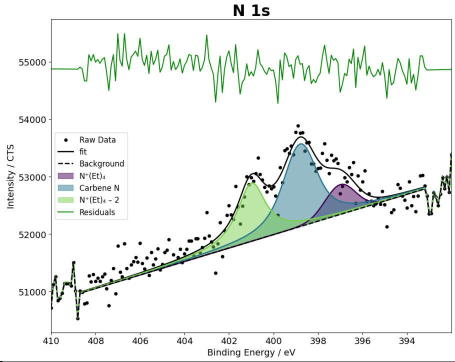
		Area	193816	3655
	3	Fit	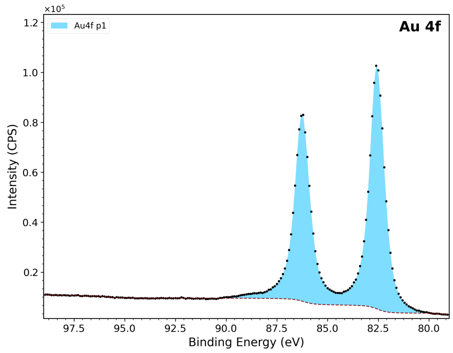	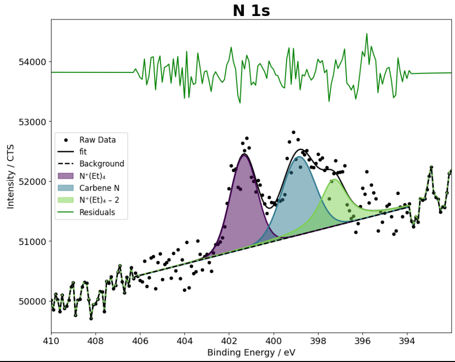
		Area	189021	2465

Table S36. *C* 1s and *I* 3d XPS spectra of monolayers of **5a** formed under optimised conditions (1 mM, 5 mM TEAB, 5 Å MS, THF, 2 h, 55 ºC).

<table>
<tr><td></td><td></td><td colspan="2">Atom</td></tr>
<tr><td></td><td></td><td>C 1s</td><td>I 3d</td></tr>
<tr><td rowspan="3">Point</td><td>1</td><td>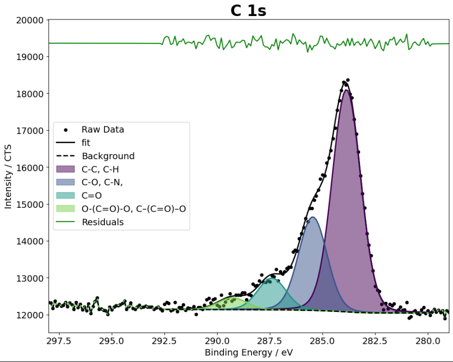
</td><td>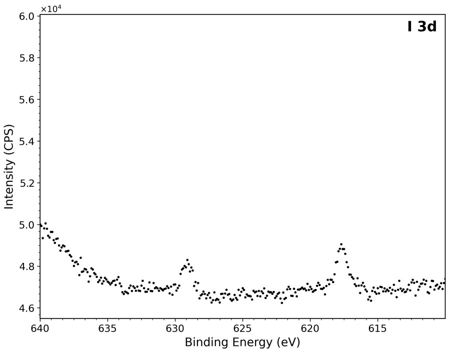
</td></tr>
<tr><td>2</td><td>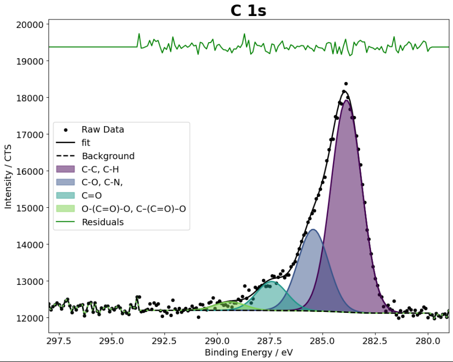
</td><td>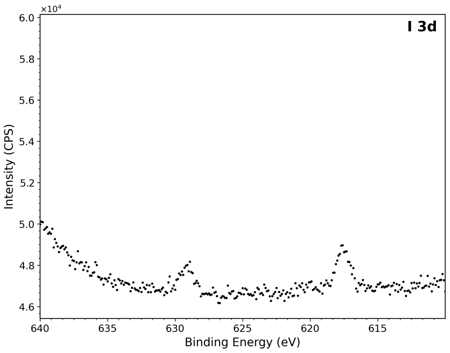
</td></tr>
<tr><td>3</td><td>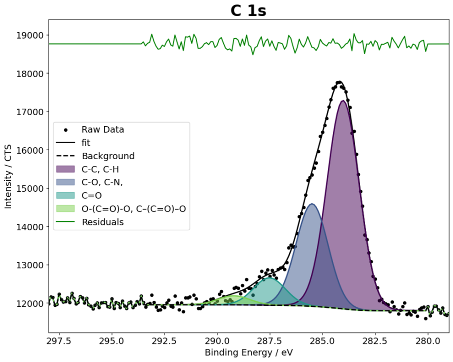
</td><td>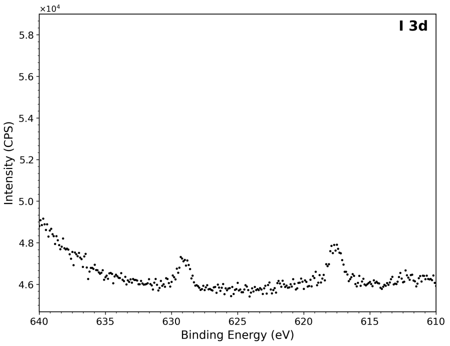
</td></tr>
</table>

Table 37. *Au* 4f and *N* 1s XPS spectra of monolayers of **6a** formed under optimised conditions (1 mM, 5 mM TEAB, 5 Å MS, THF, 2 h, 55 ºC).

			Atom	
			Au 4f	*N* 1s
Point	1	Fit	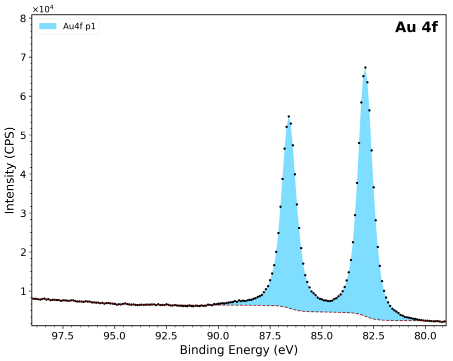	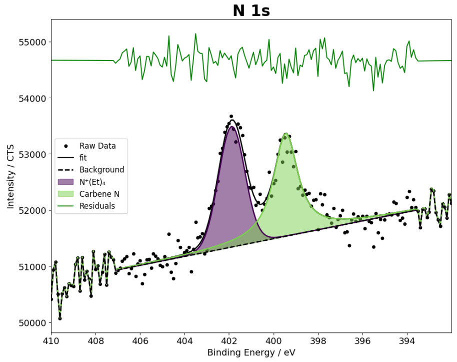
		Area	121174	3721
	2	Fit	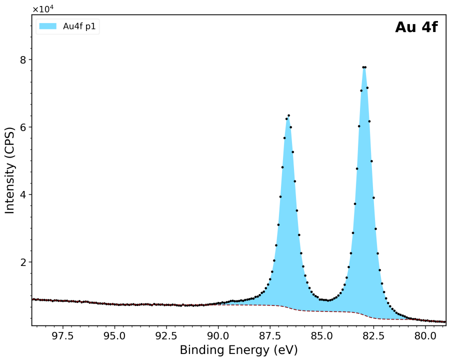	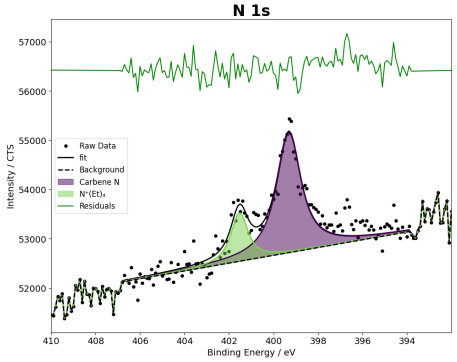
		Area	140511	4874
	3	Fit	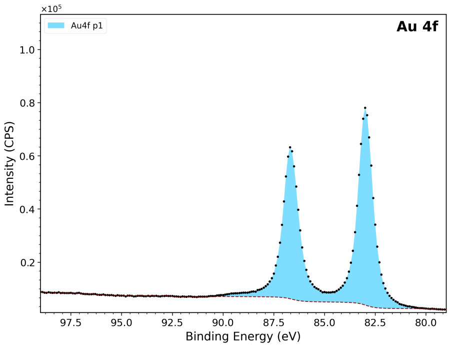	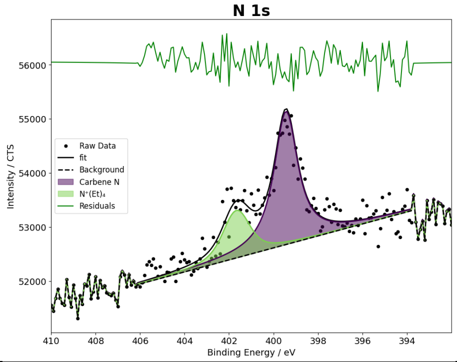
		Area	142215	4841

Table S38. *C* 1s and *I* 3d XPS spectra of monolayers of **6a** formed under optimised conditions (1 mM, 5 mM TEAB, 5 Å MS, THF, 2 h, 55 ºC).

		Atom	
		C 1s	*I* 3d
Point	1	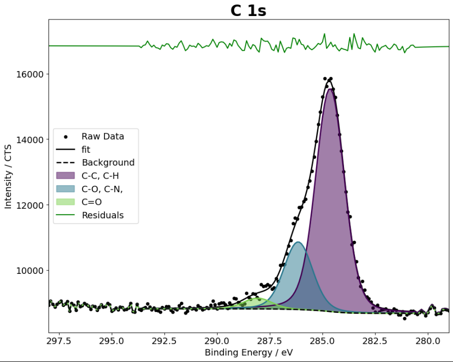	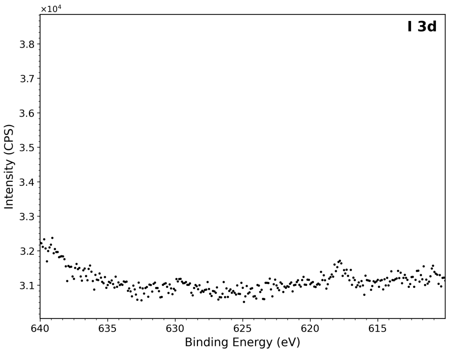
	2	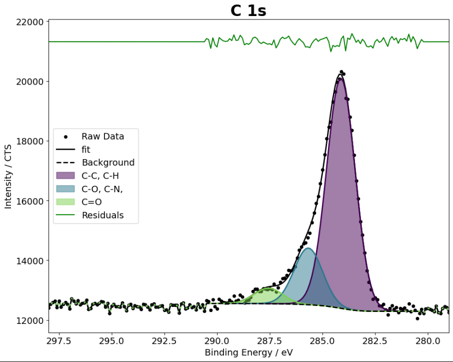	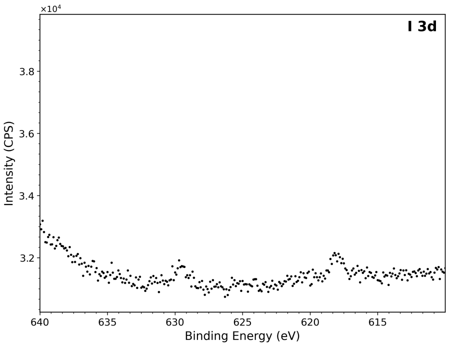
	3	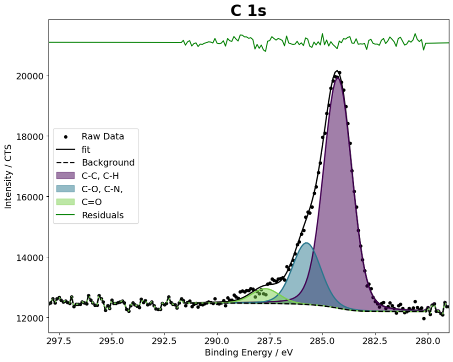	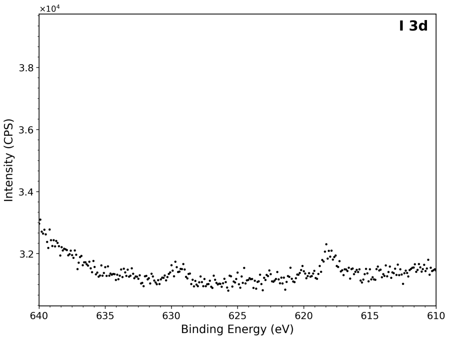

Table 39. *Au* 4f and *N* 1s XPS spectra of monolayers of **7a** formed under optimised conditions (1 mM, 5 mM TEAB, 5 Å MS, THF, 2 h, 55 ºC).

<table>
<tr><td></td><td></td><td></td><td colspan="2">Atom</td></tr>
<tr><td></td><td></td><td></td><td>Au 4f</td><td>N 1s</td></tr>
<tr><td rowspan="6">Point</td><td rowspan="2">1</td><td>Fit</td><td>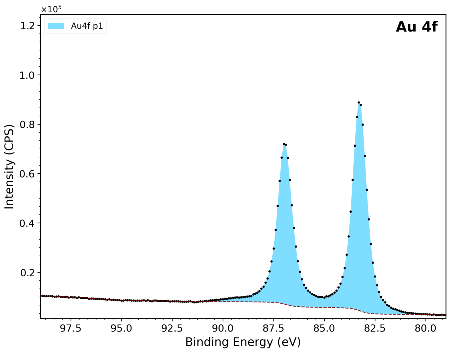
</td><td>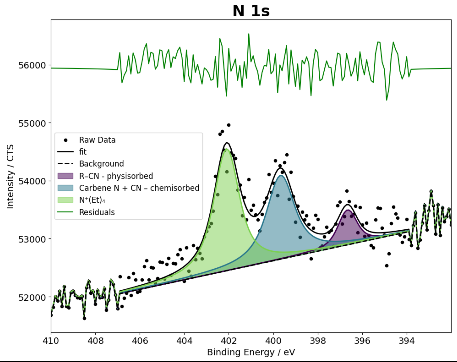
</td></tr>
<tr><td>Area</td><td>163274</td><td>3239.00</td></tr>
<tr><td rowspan="2">2</td><td>Fit</td><td>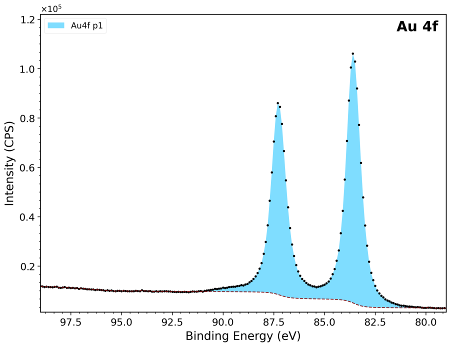
</td><td>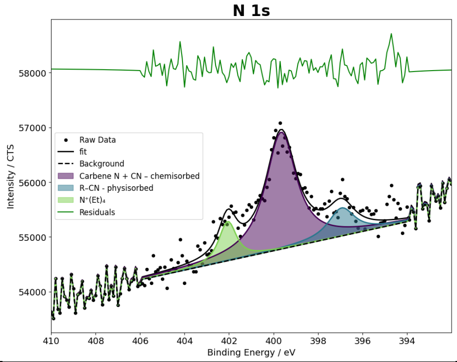
</td></tr>
<tr><td>Area</td><td>195364</td><td>5456</td></tr>
<tr><td rowspan="2">3</td><td>Fit</td><td>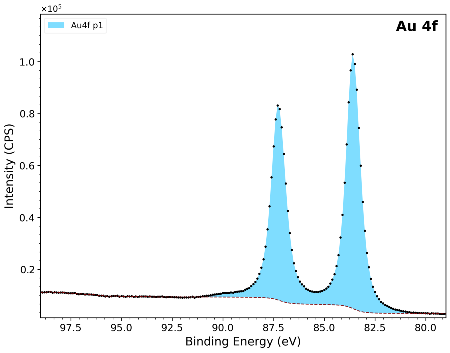
</td><td>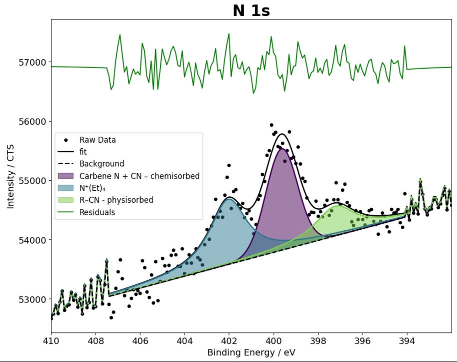
</td></tr>
<tr><td>Area</td><td>187684</td><td>3071</td></tr>
</table>

Table S40. *C* 1s and *I* 3d XPS spectra of monolayers of **7a** formed under optimised conditions (1 mM, 5 mM TEAB, 5 Å MS, THF, 2 h, 55 ºC).

<table>
<tr><th></th><th></th><th colspan="2">Atom</th></tr>
<tr><th></th><th></th><th>C 1s</th><th>I 3d</th></tr>
<tr><td rowspan="3">Point</td><td>1</td><td>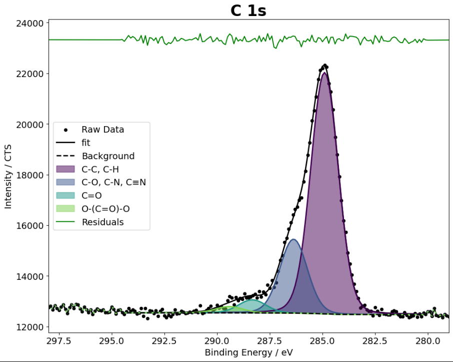
</td><td>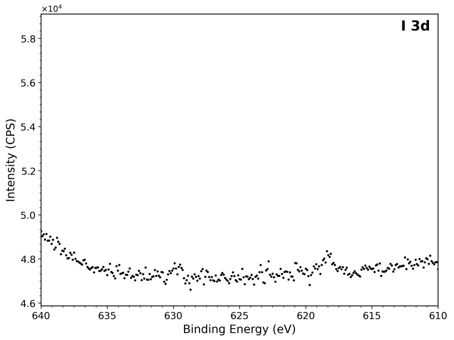
</td></tr>
<tr><td>2</td><td>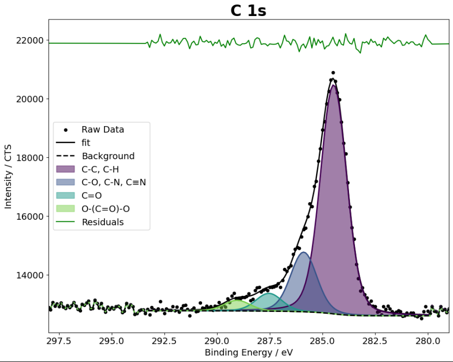
</td><td>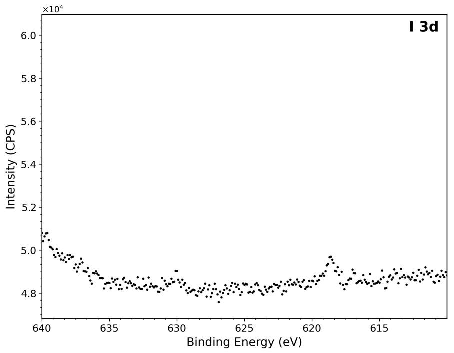
</td></tr>
<tr><td>3</td><td>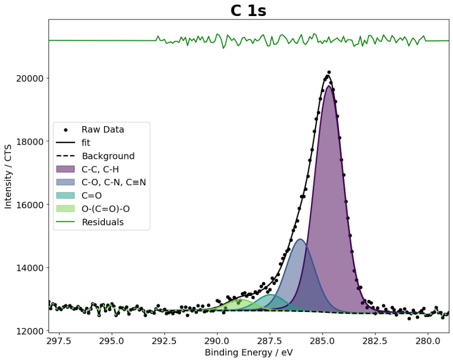
</td><td>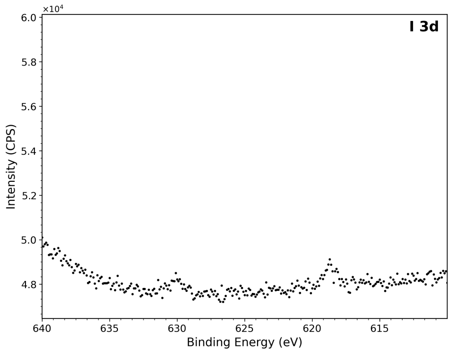
</td></tr>
</table>

Table 41. *Au* 4f and *N* 1s XPS spectra of monolayers of **8a** formed under optimised conditions (1 mM, 5 mM TEAB, 5 Å MS, THF, 2 h, 55 ºC).

<table>
<tr><td colspan="3"></td><td colspan="2">Atom</td></tr>
<tr><td colspan="3"></td><td>Au 4f</td><td>N 1s</td></tr>
<tr><td rowspan="6">Point</td><td rowspan="2">1</td><td>Fit</td><td>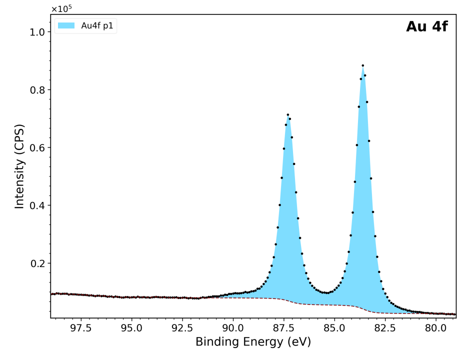
</td><td>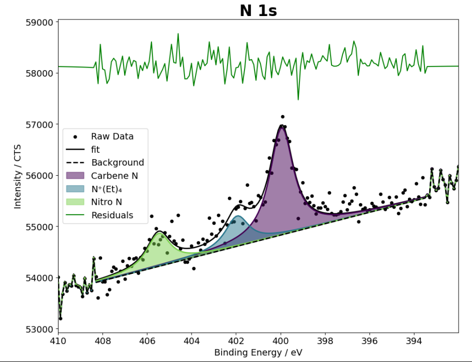
</td></tr>
<tr><td>Area</td><td>164618</td><td>4115</td></tr>
<tr><td rowspan="2">2</td><td>Fit</td><td>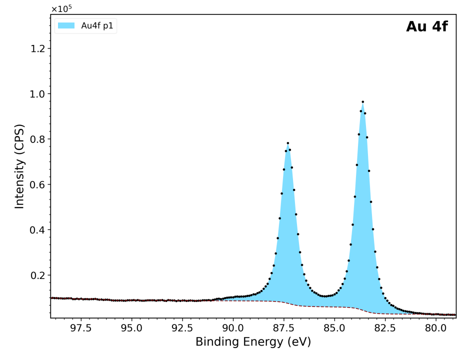
</td><td>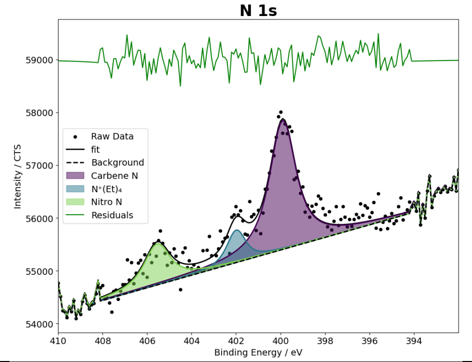
</td></tr>
<tr><td>Area</td><td>179236</td><td>5232</td></tr>
<tr><td rowspan="2">3</td><td>Fit</td><td>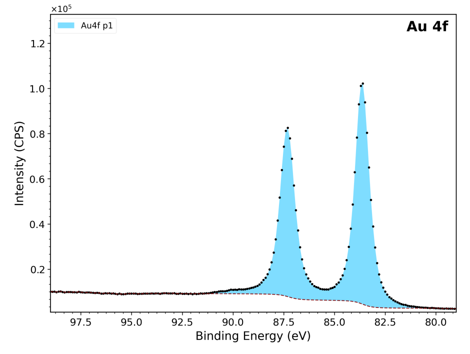
</td><td>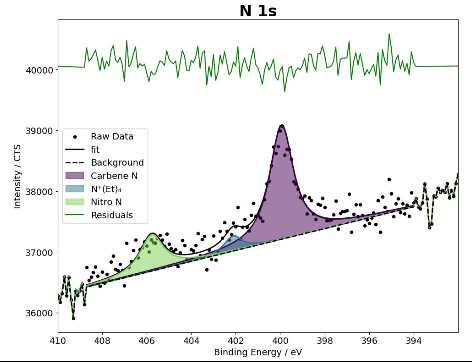
</td></tr>
<tr><td>Area</td><td>190300</td><td>3672</td></tr>
</table>

Table S42. *C* 1s and *I* 3d XPS spectra of monolayers of **8a** formed under optimised conditions (1 mM, 5 mM TEAB, 5 Å MS, THF, 2 h, 55 ºC).

		Atom	
		C 1s	*I* 3d
Point	1	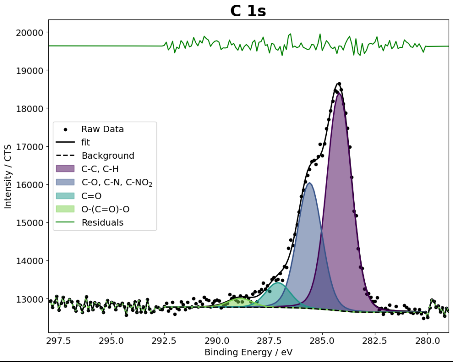	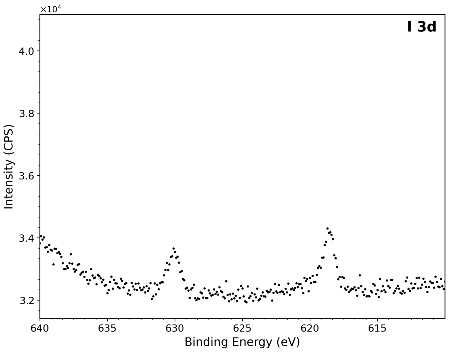
	2	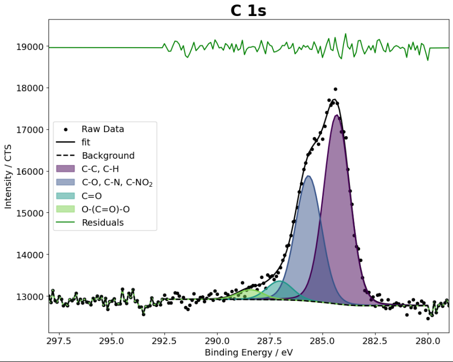	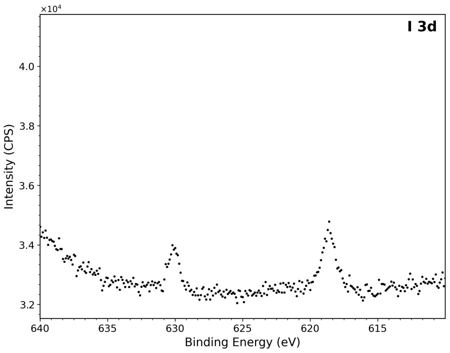
	3	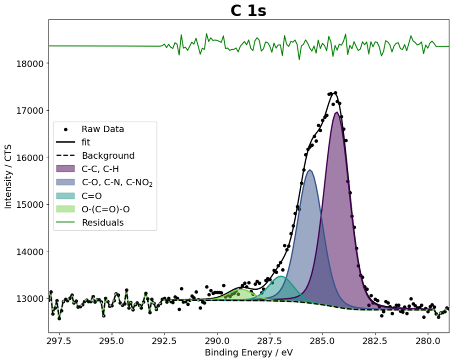	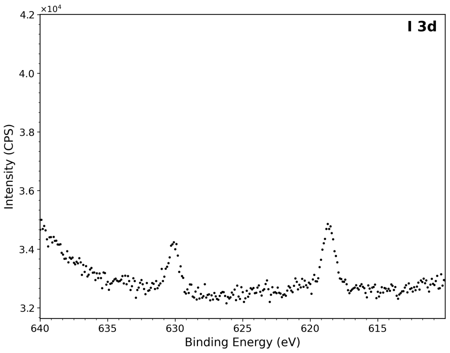

S9 DFT Studies

The free energies, G, at 328.15 K including ZPE correction were extracted from the log files and utilised to generate the free energy plots and comparative energy charts.

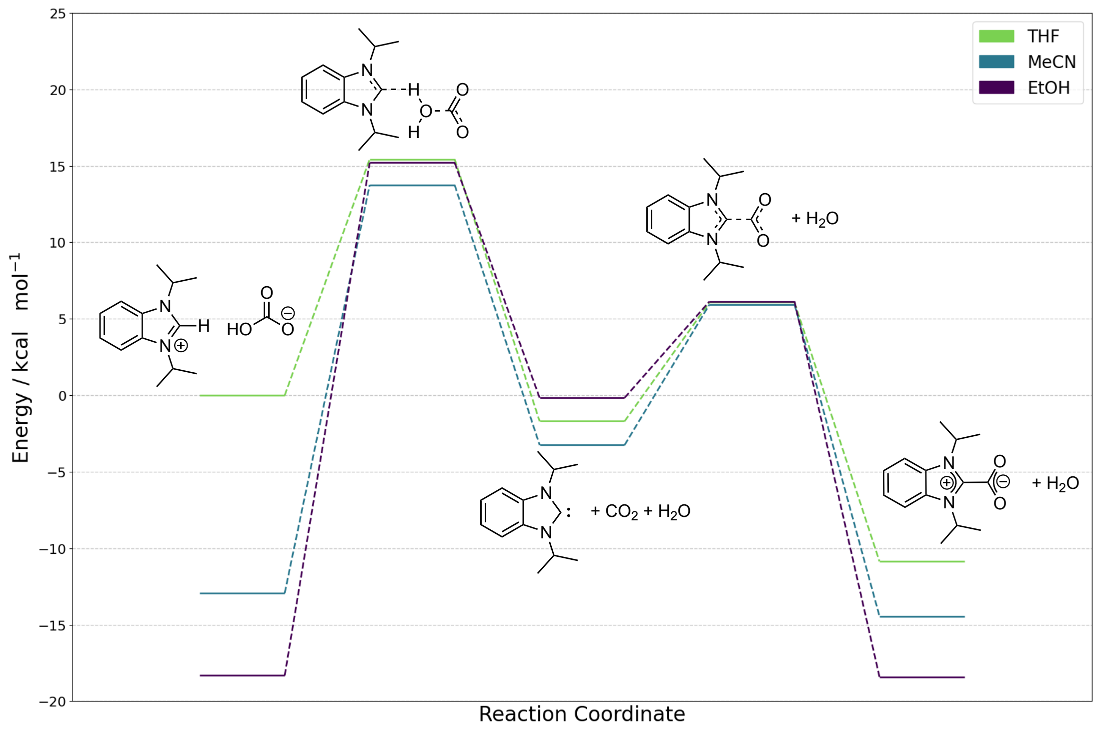

Figure S47. Equilibrium for the energy profile of **1** and the bicarbonate anion, and the 2-carboxy species *via* the free carbene in different solvents. The energy of **1** and the bicarbonate anion in THF are set as reference to 0.

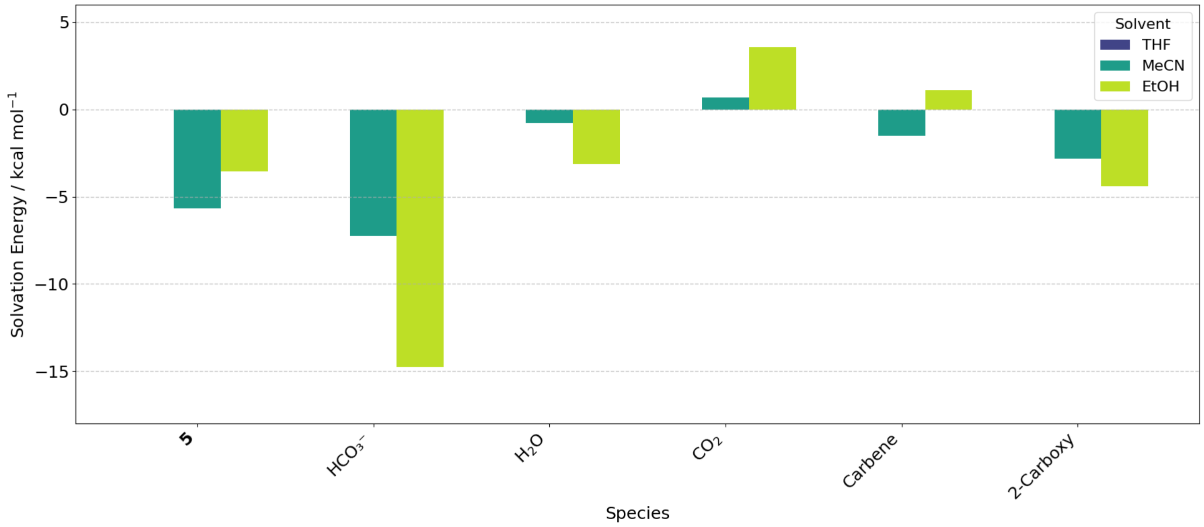

Figure S48. Energy of the species involved in the equilibrium in different solvents relative to their energy in THF for **1**.

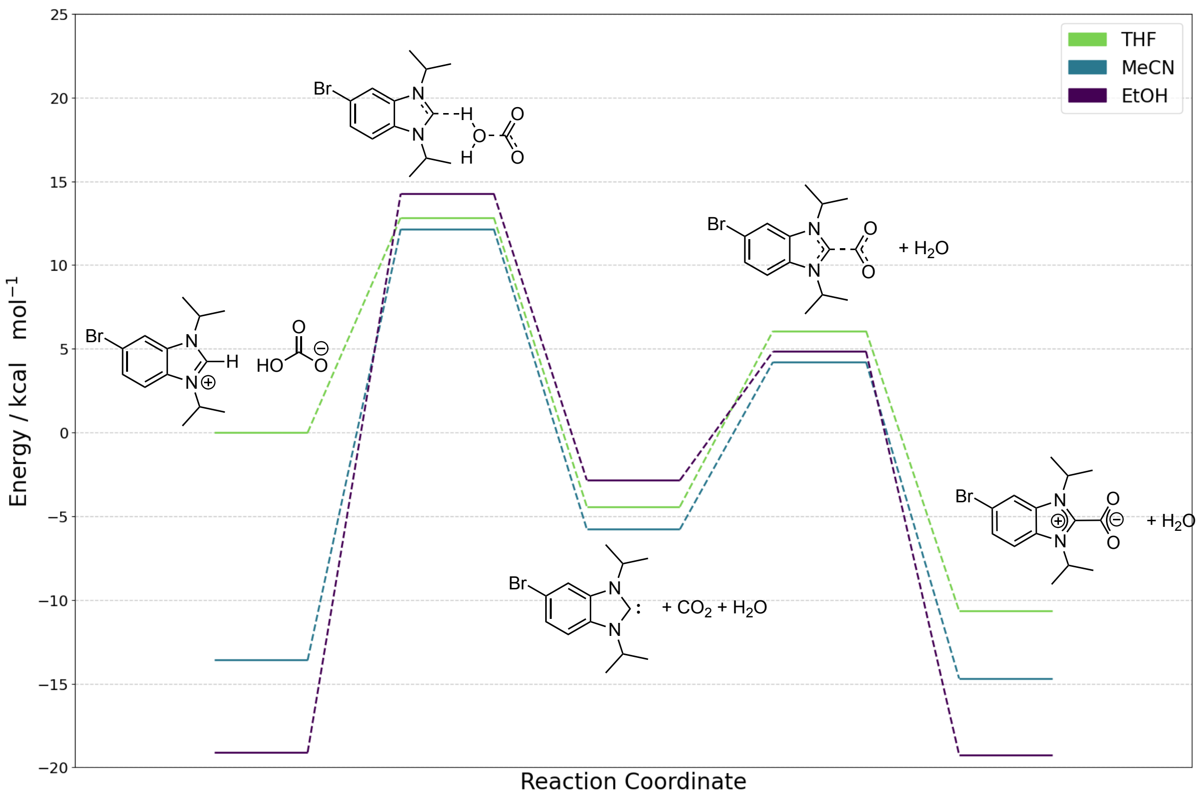

Figure S49. Equilibrium for the energy profile of **2** and the bicarbonate anion, and the 2-carboxy specie *via* the free carbene in different solvents. The energy of **2** and the bicarbonate anion in THF are set as reference to 0.

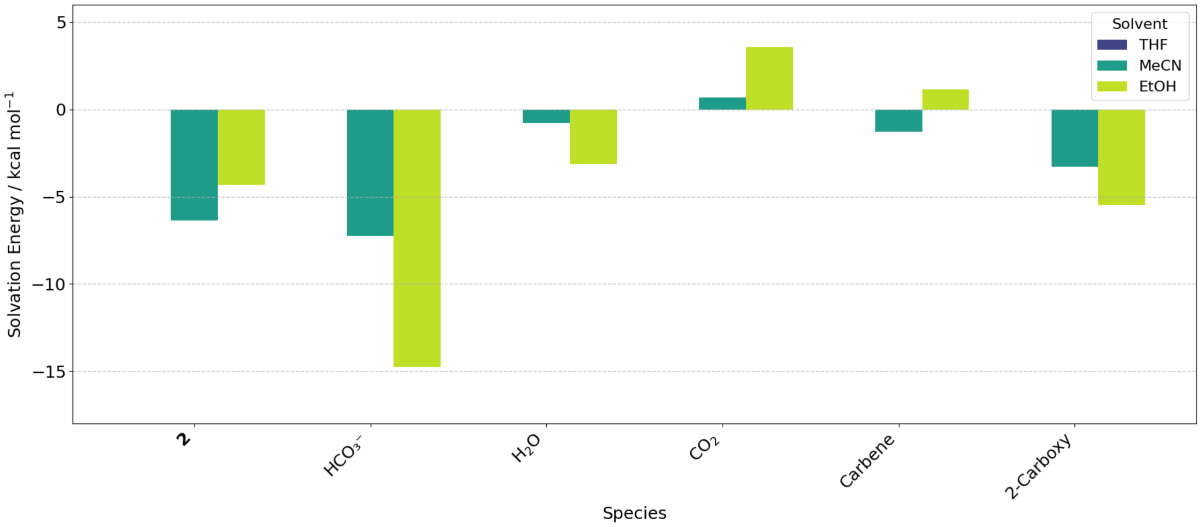

Figure S50. Energy of the species involved in the equilibrium in different solvents relative to their energy in THF for **2**.

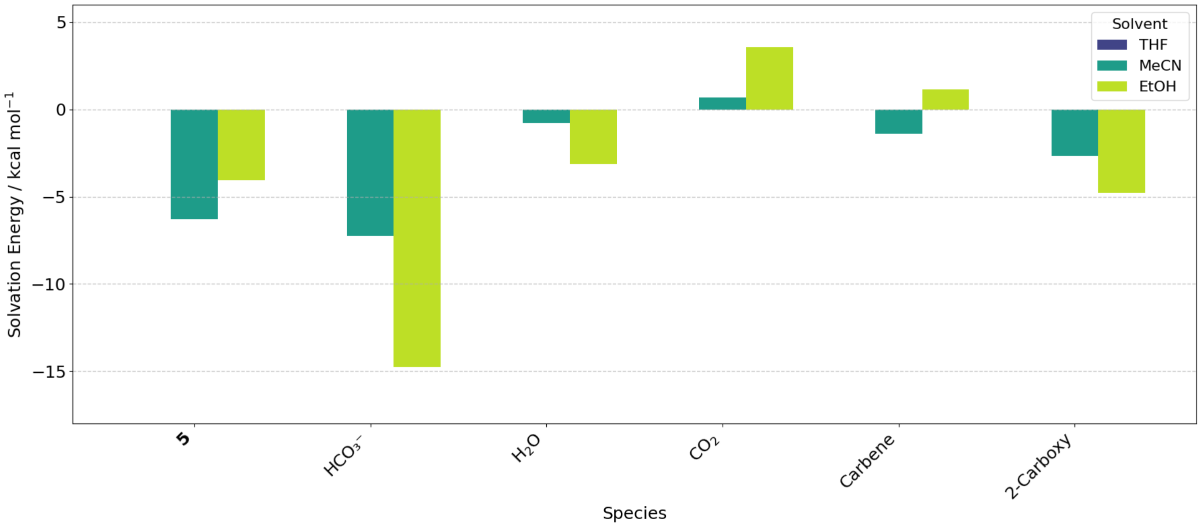

Figure S51. Energy of the species involved in the equilibrium in different solvents relative to their energy in THF for **5**.

S10 NMR Spectra

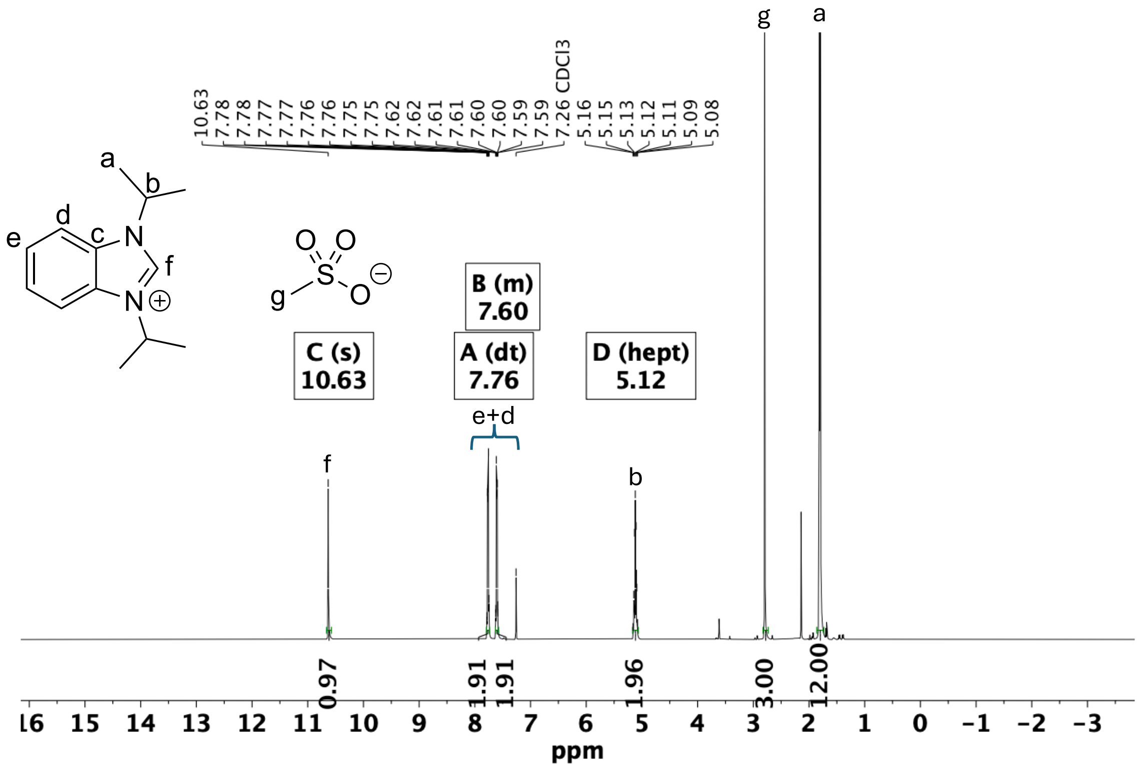

Figure S52. 500 MHz ^{1}H NMR spectrum of **1a**.

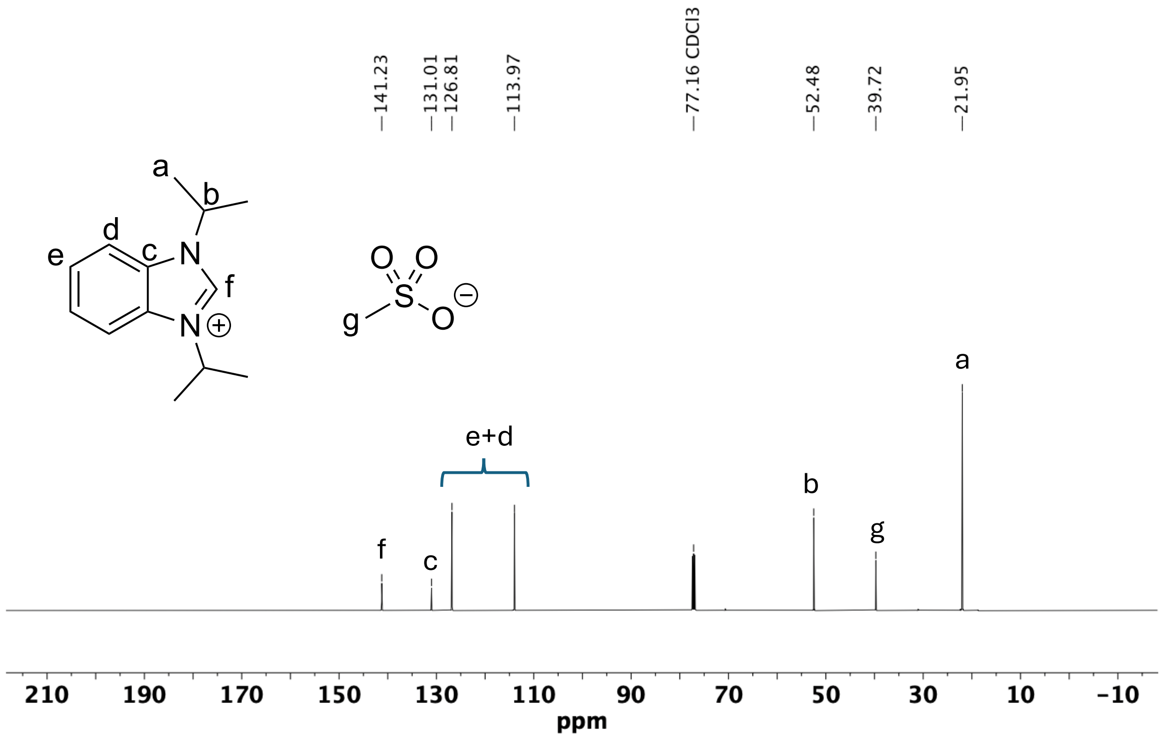

Figure S53. 126 MHz ^{13}C NMR spectrum of **1a**.

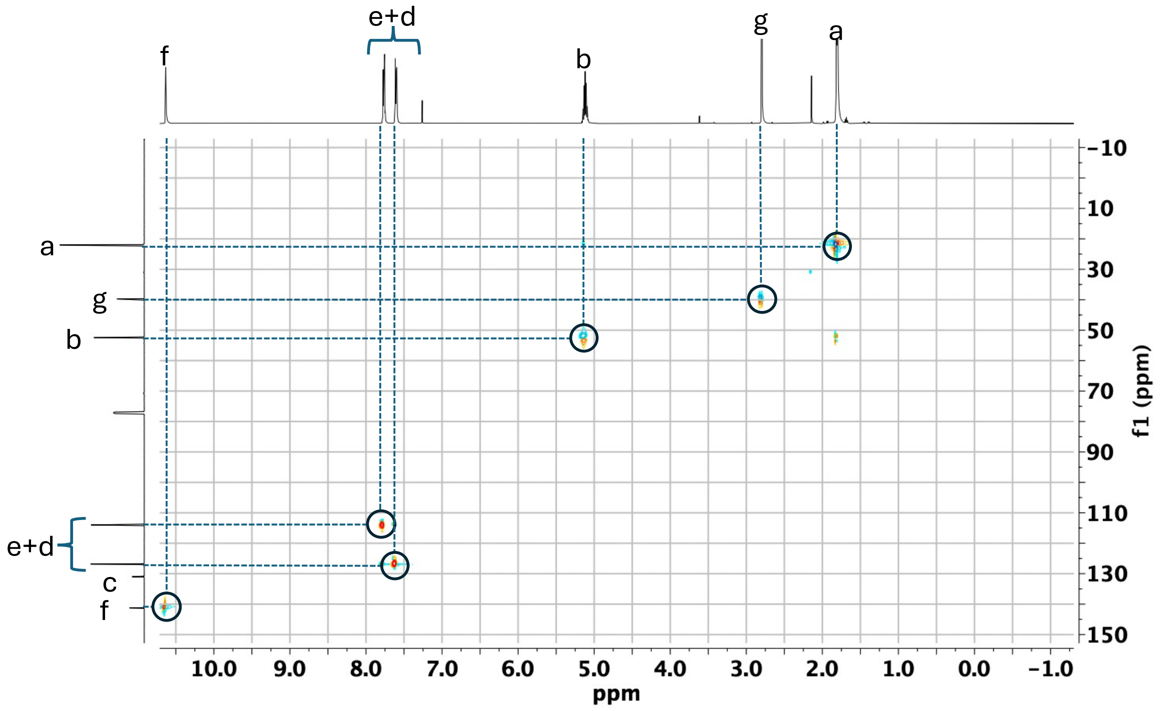

Figure S54. ^{1}H-^{13}C HSQC of compound **1a**.

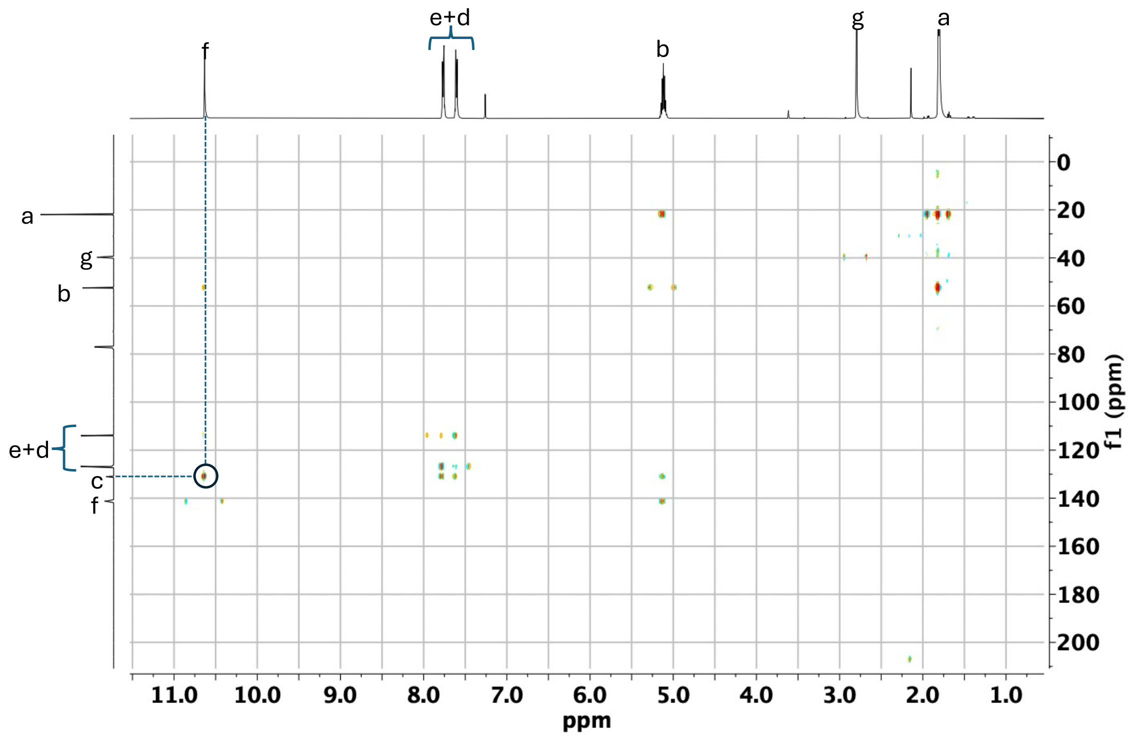

Figure S55. ^{1}H-^{13}C HMBC of compound **1a**.

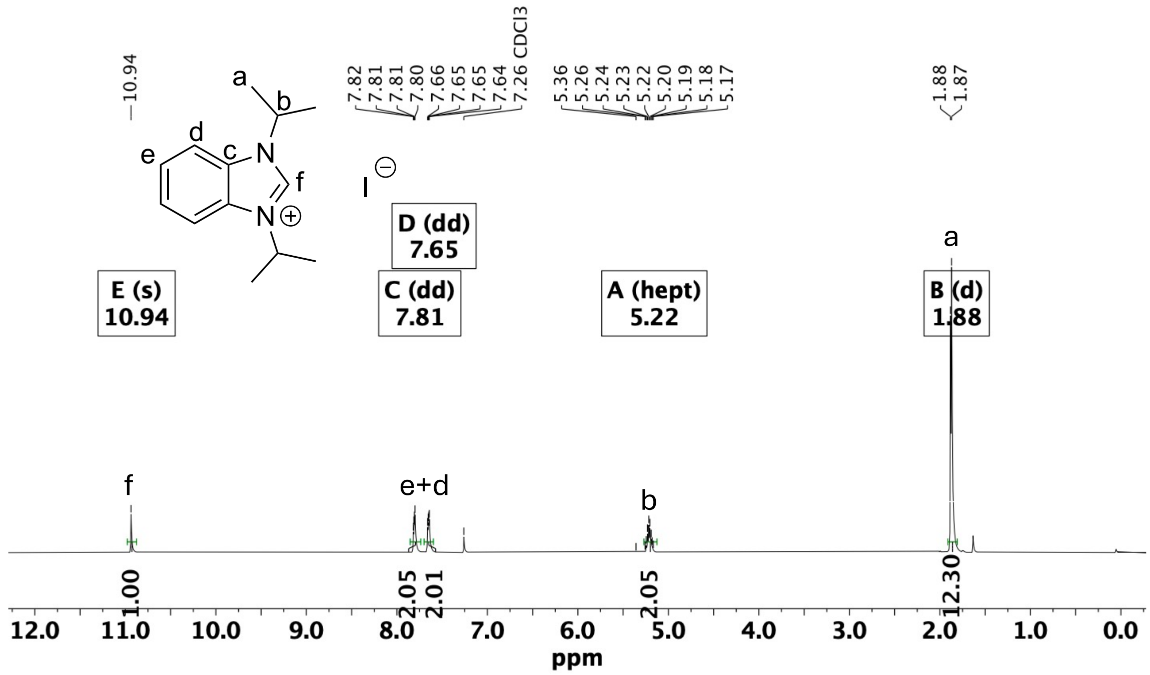

Figure S56. 500 MHz ^{1}H NMR spectrum of **1b**.

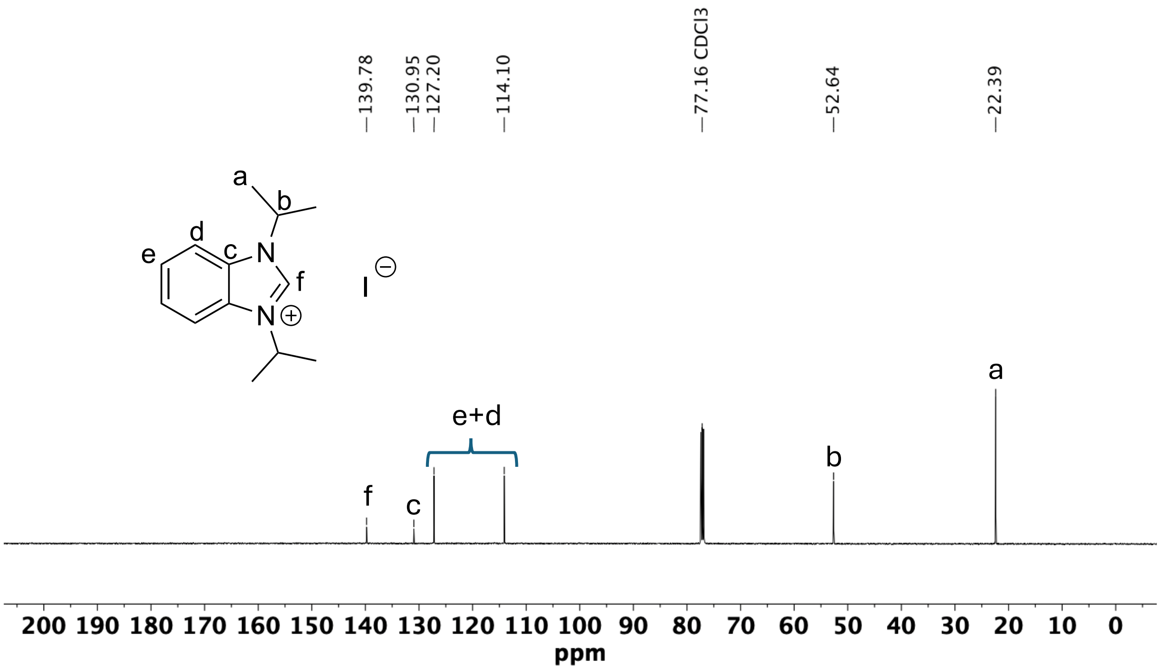

Figure S57. 126 MHz ^{13}C NMR spectrum of **1b**.

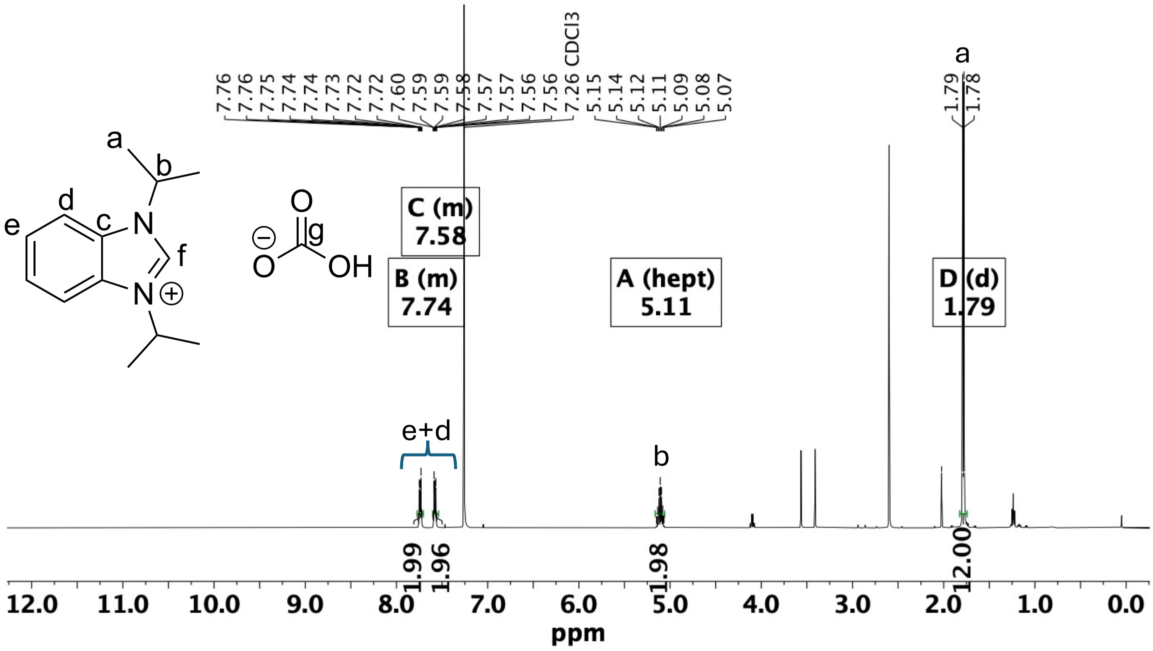

Figure S58. 500 MHz ^{1}H NMR spectrum of **1c**.

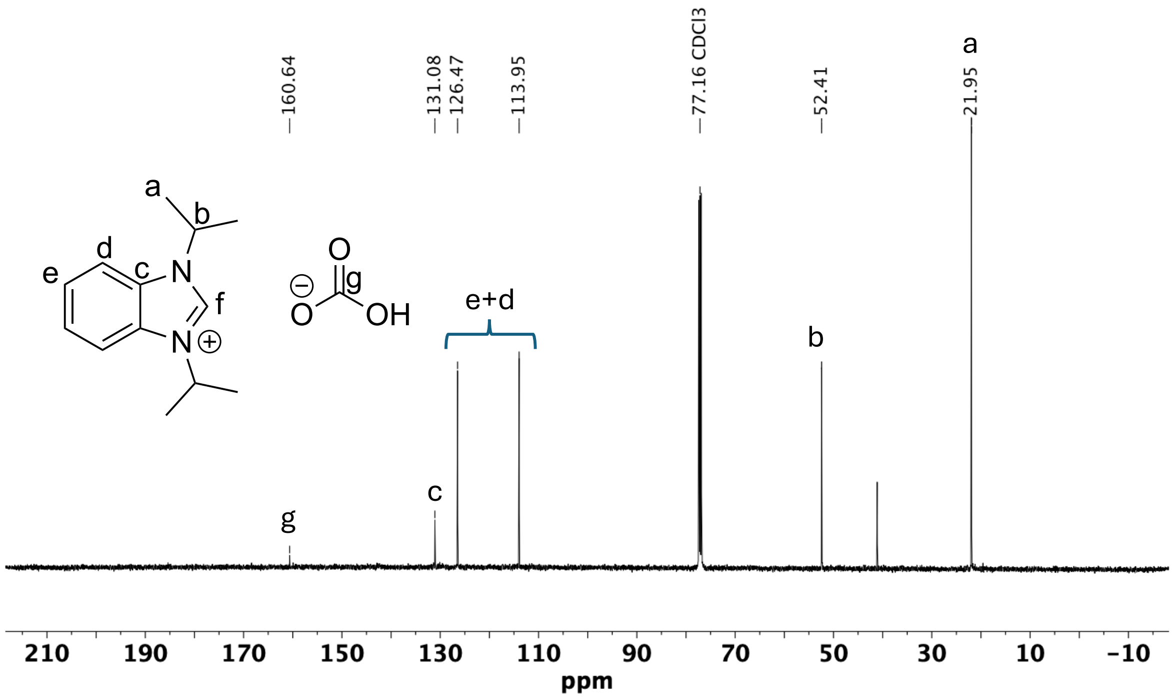

Figure S59. 126 MHz ^{13}C NMR spectrum of **1c**.

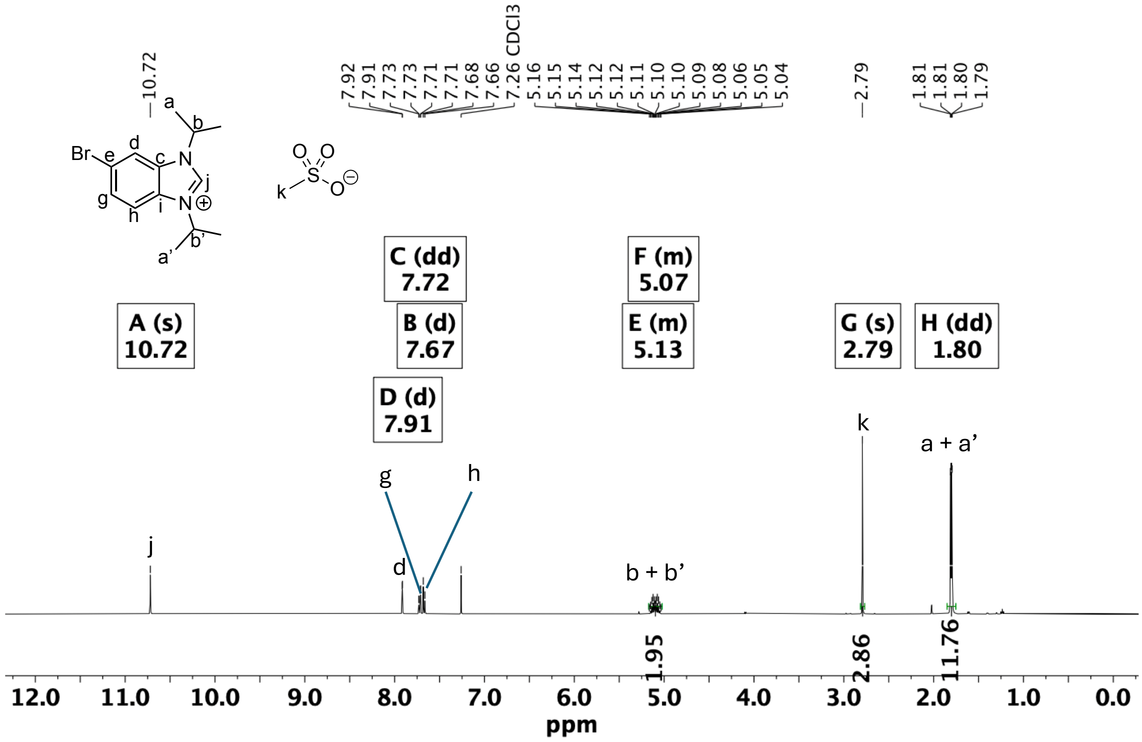

Figure S60. 500 MHz ^{1}H NMR spectrum of **2a**.

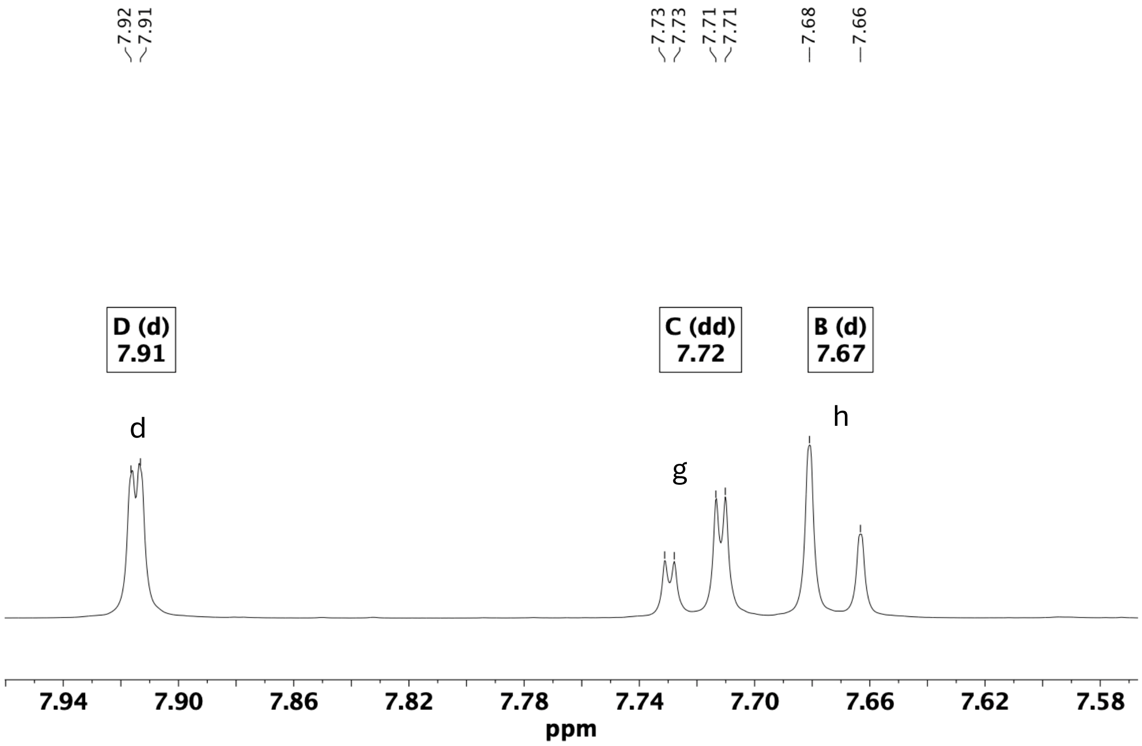

Figure S61. Zoomed–in 500 MHz ^1H NMR spectrum of **2a**.

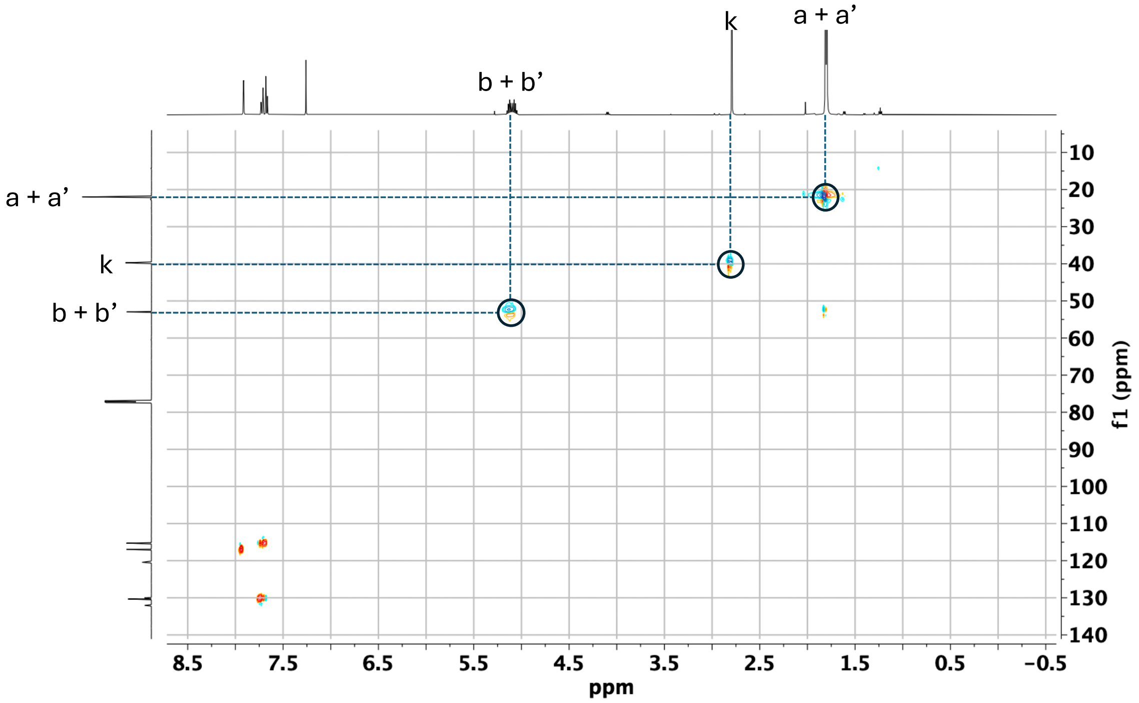

Figure S62. ^1H-^{13}C HSQC of compound **2a**.

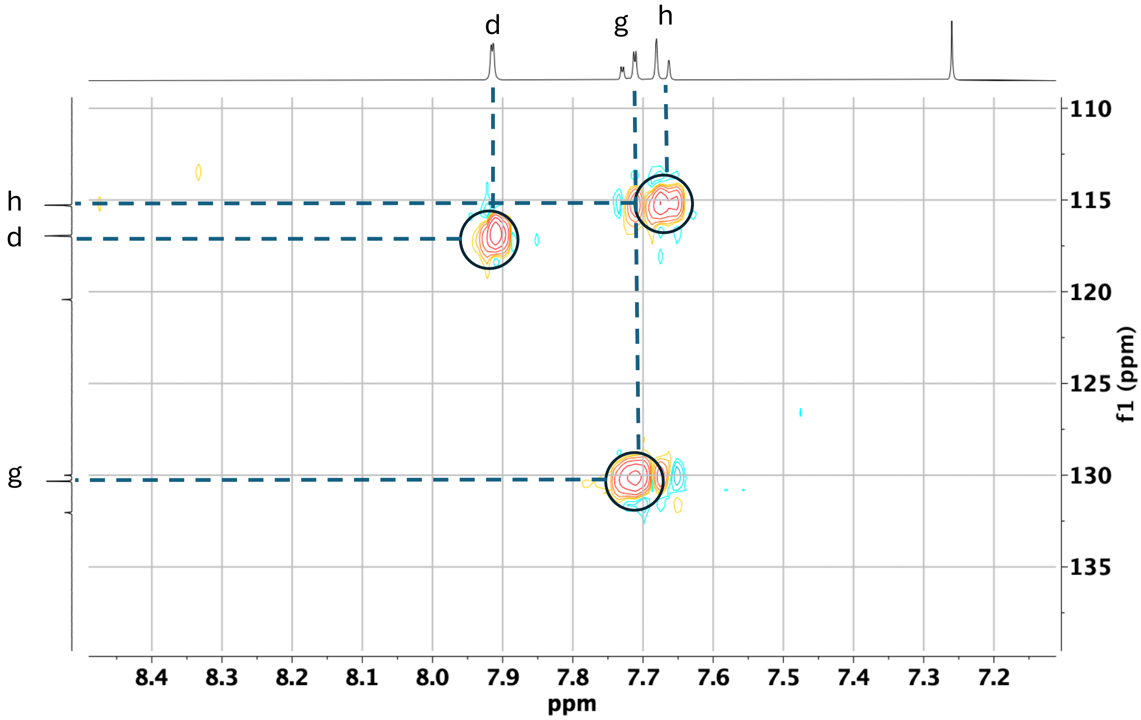

Figure S63. Zoomed–in ^{1}H-^{13}C HSQC of compound **2a**.

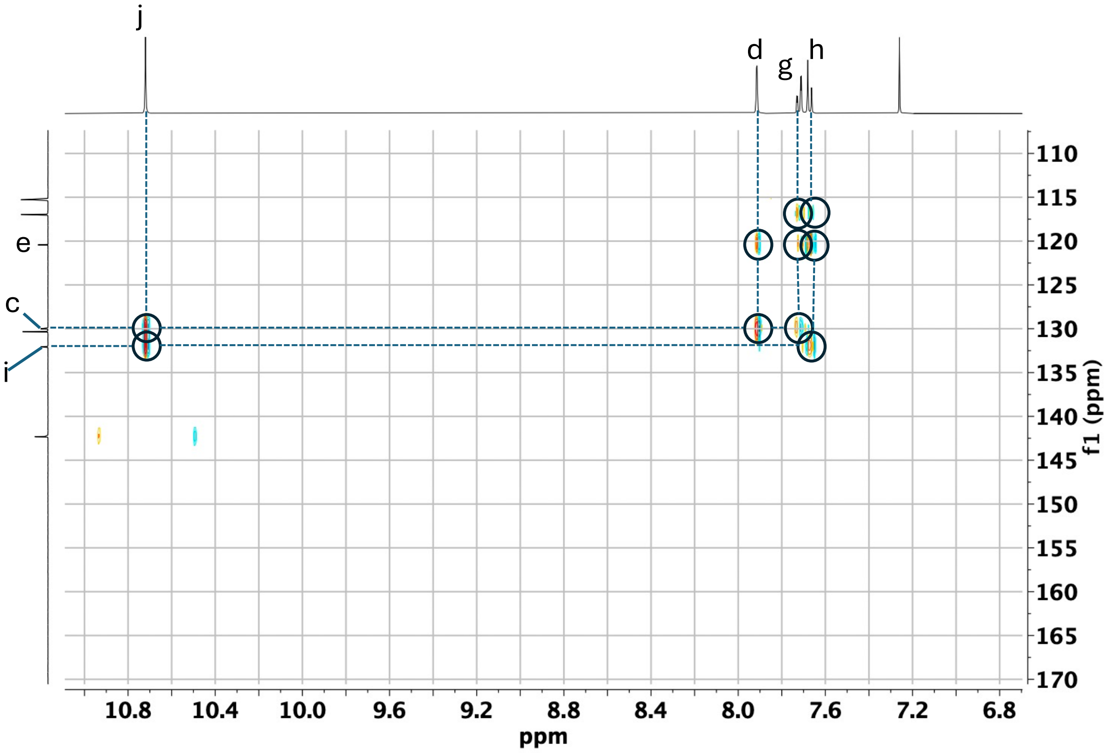

Figure S64. Zoomed–in ^{1}H-^{13}C HMBC of compound **2a**.

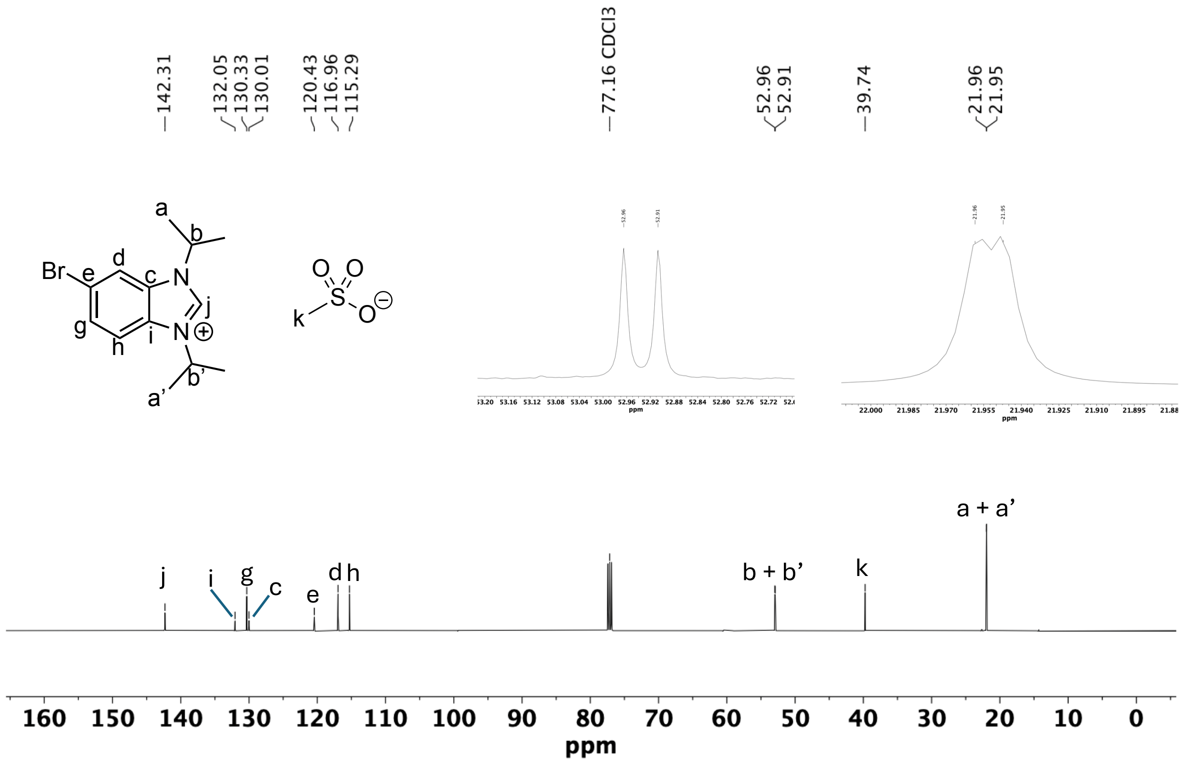

Figure S67. 126 MHz ^{13}C NMR spectrum of **2a**.

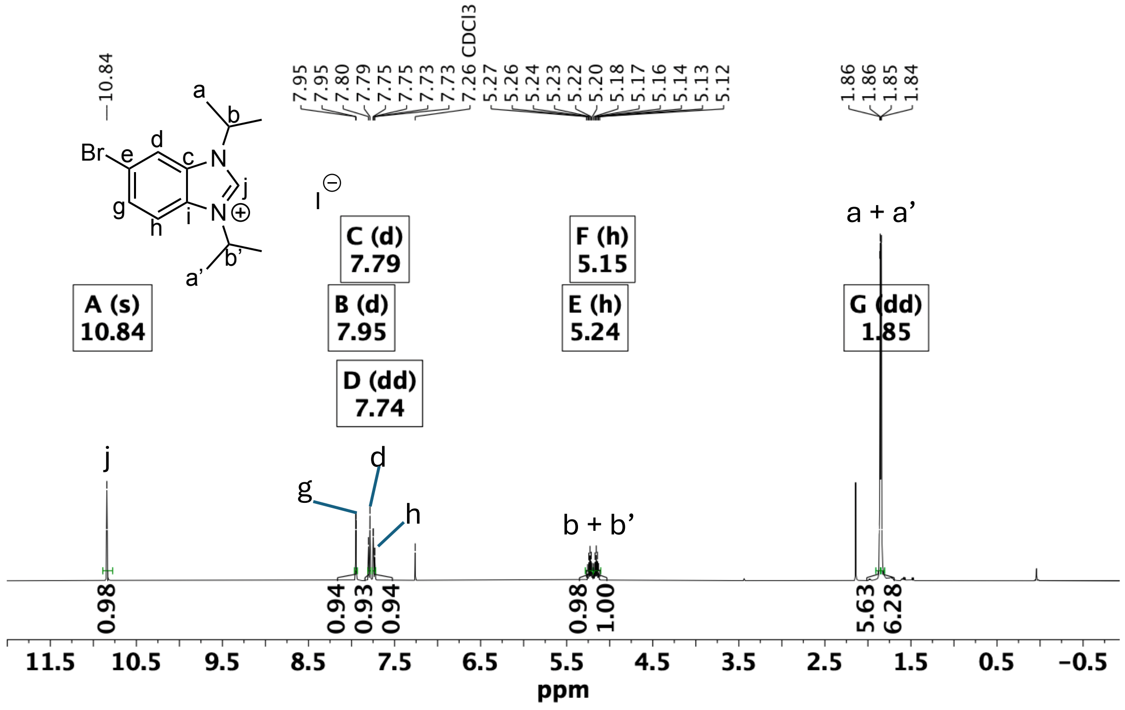

Figure S68. 500 MHz ^{1}H NMR spectrum of **2b**.

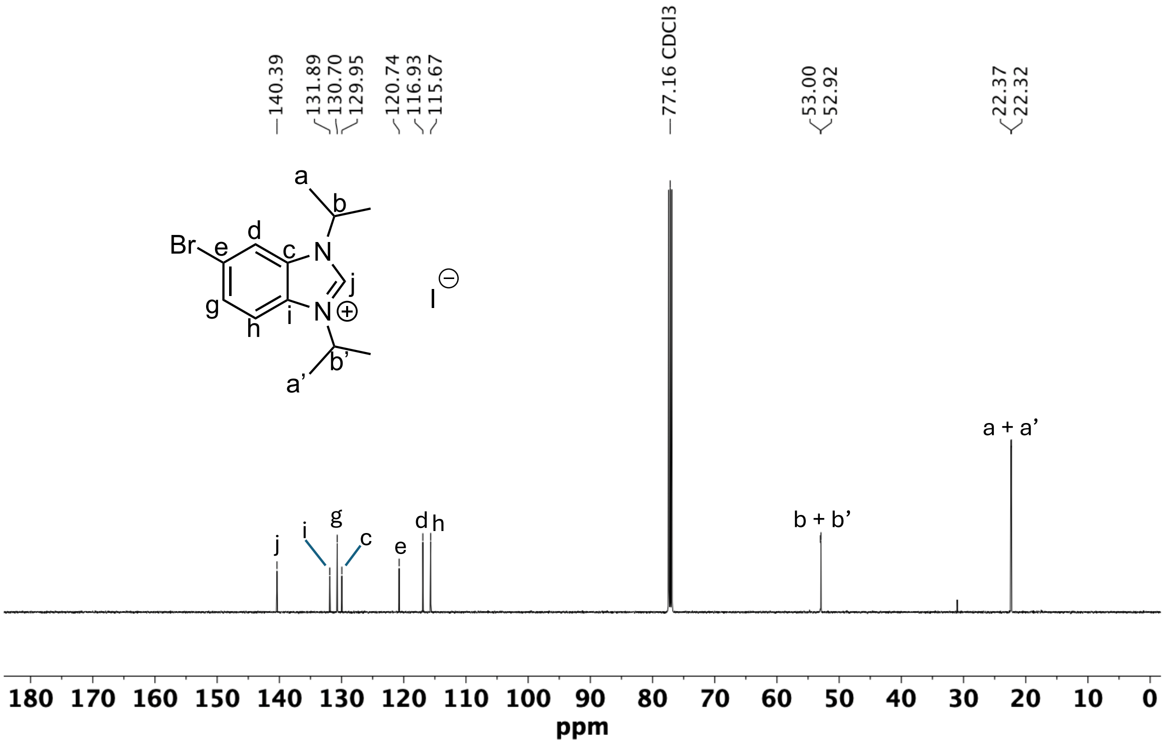

Figure S69. 126 MHz ^{13}C NMR spectrum of **2b**.

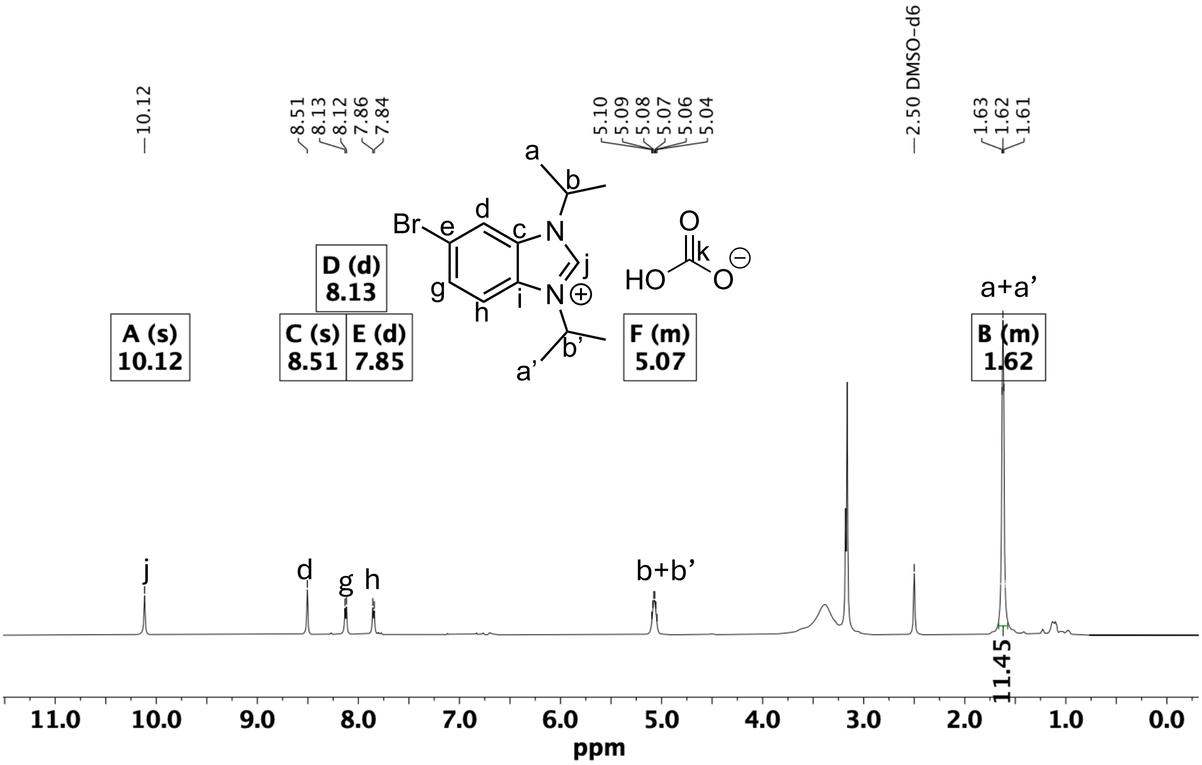

Figure S70. 600 MHz ^{1}H NMR spectrum of **2c**.

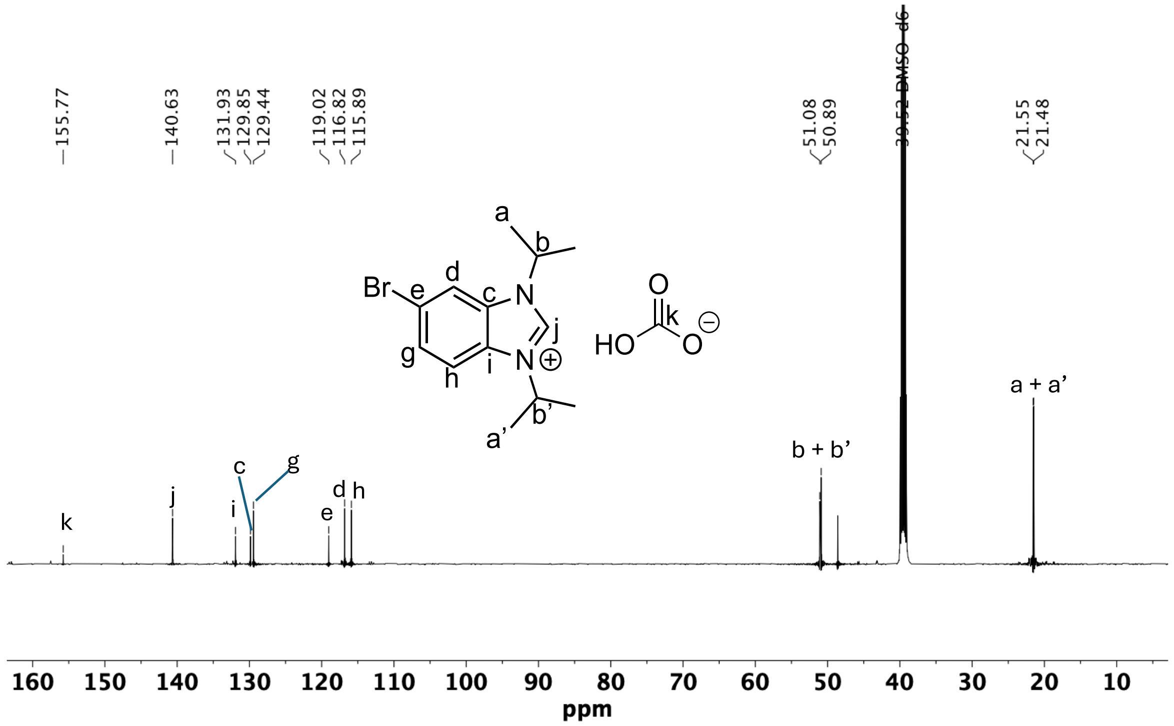

Figure S71. 151 MHz ^{13}C NMR spectrum of **2c**.

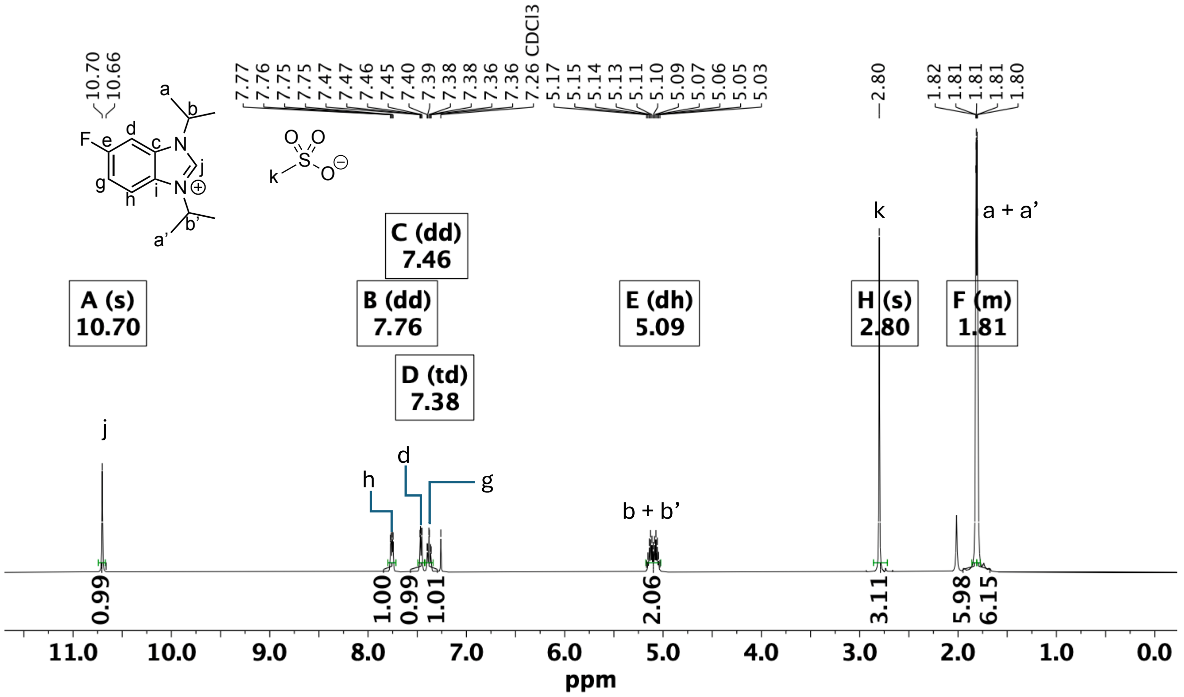

Figure S72. 500 MHz ^{1}H NMR spectrum of **3a**.

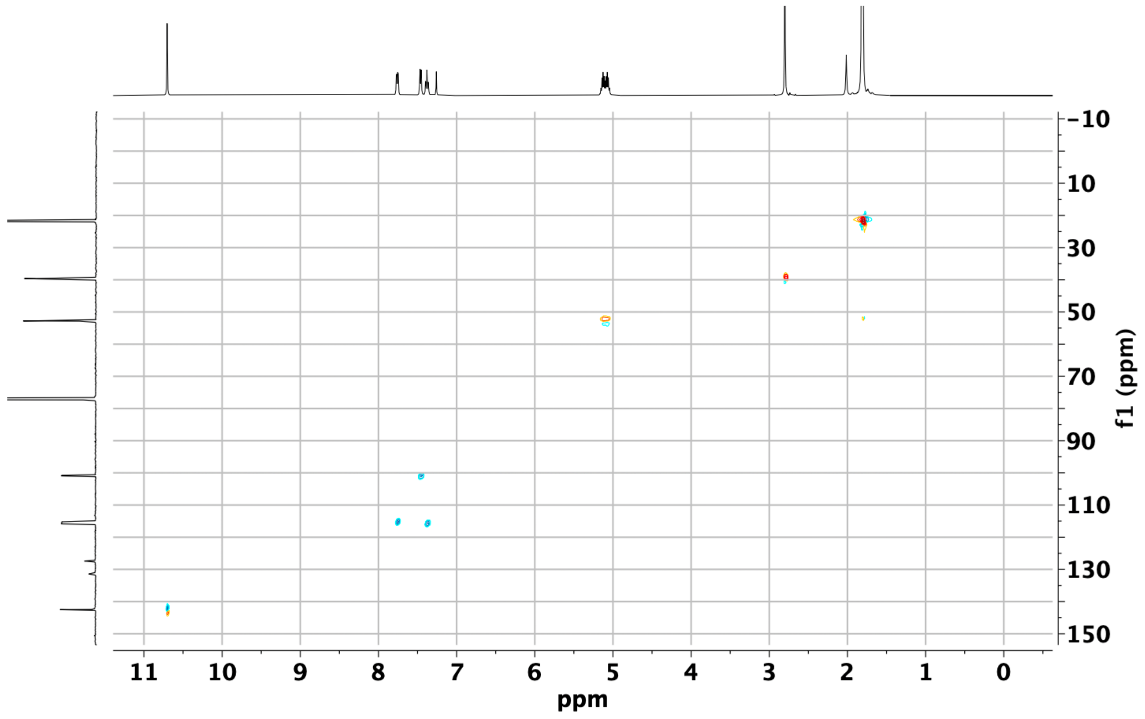

Figure S73. ^{1}H-^{13}C HSQC spectrum of **3a**.

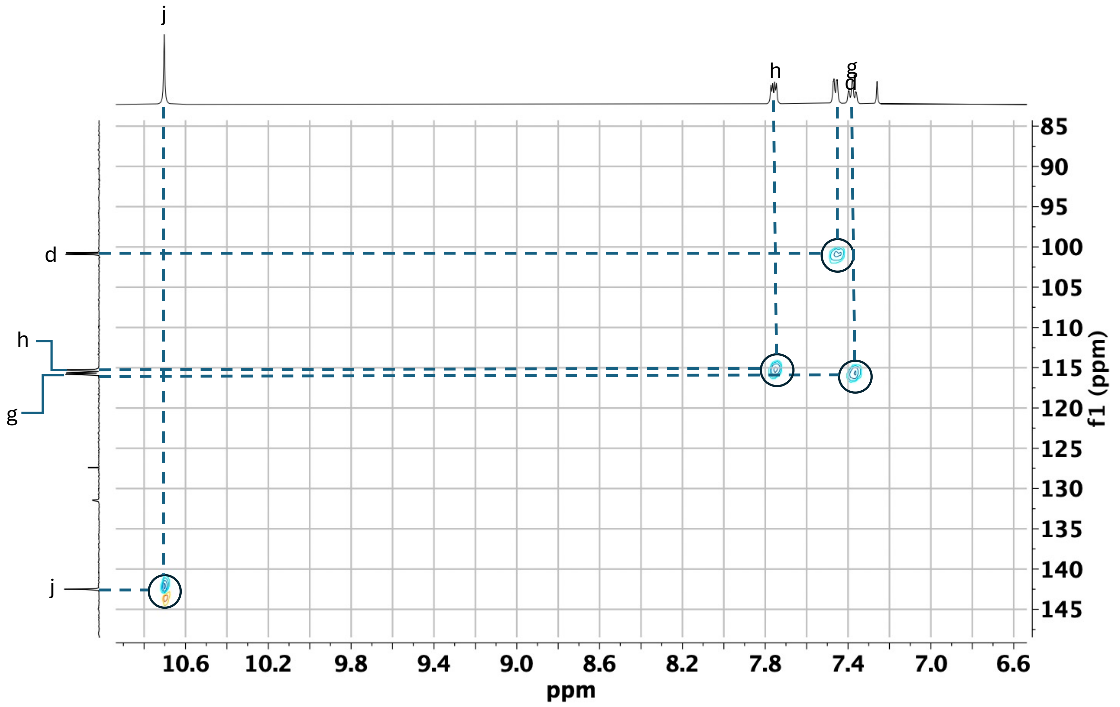

Figure S74. Zoomed–in ^{1}H-^{13}C HSQC spectrum of **3a**.

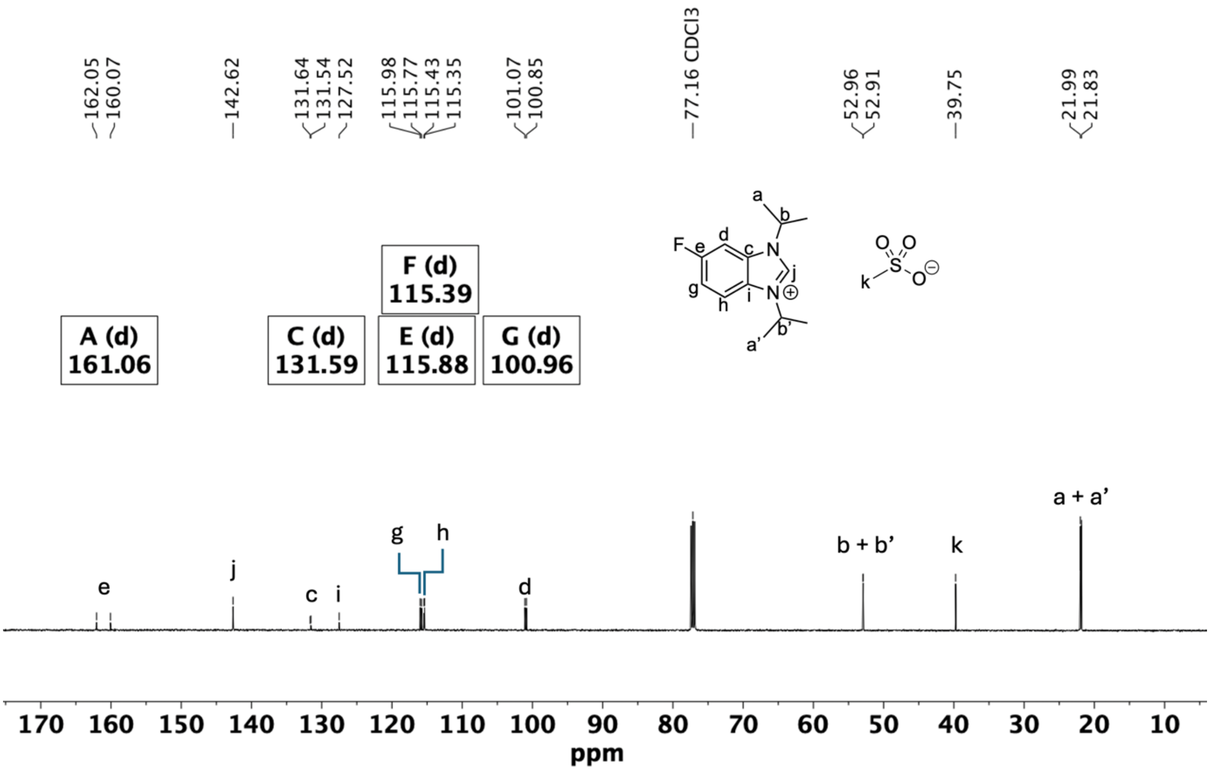

Figure S75. 126 MHz^{13}C spectrum of **3a**.

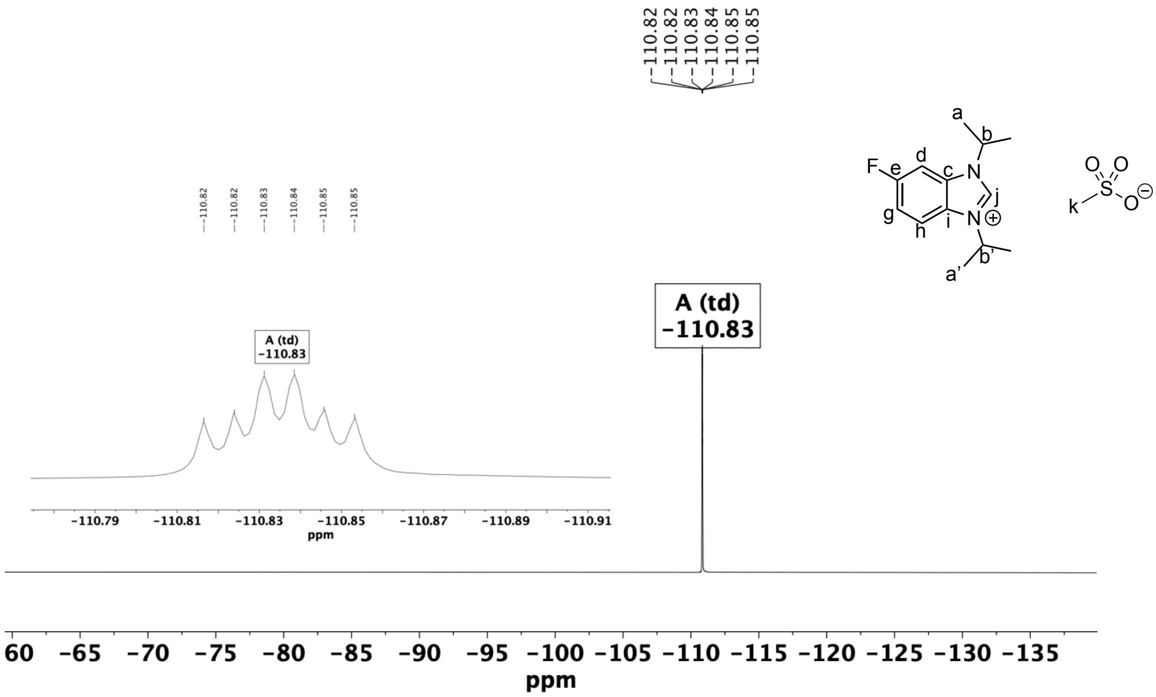

Figure S76. 565 MHz ^{19}F NMR spectrum of **3a**.

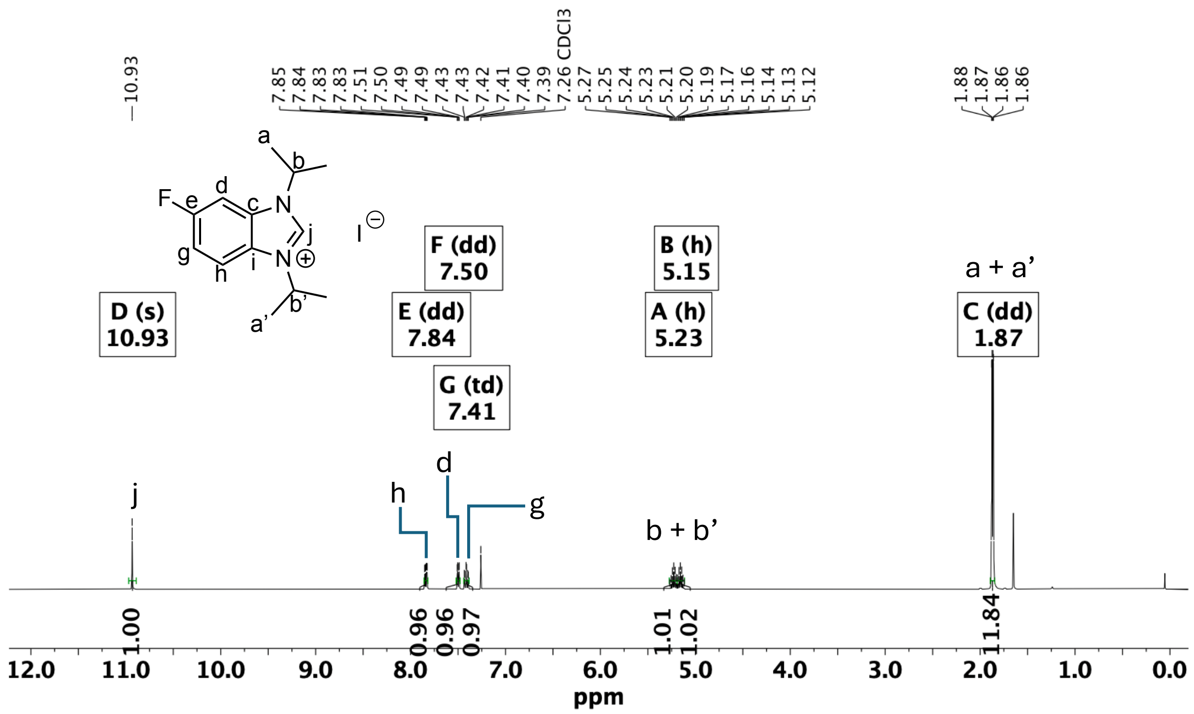

Figure S77. 500 MHz ^{1}H spectrum of **3b**.

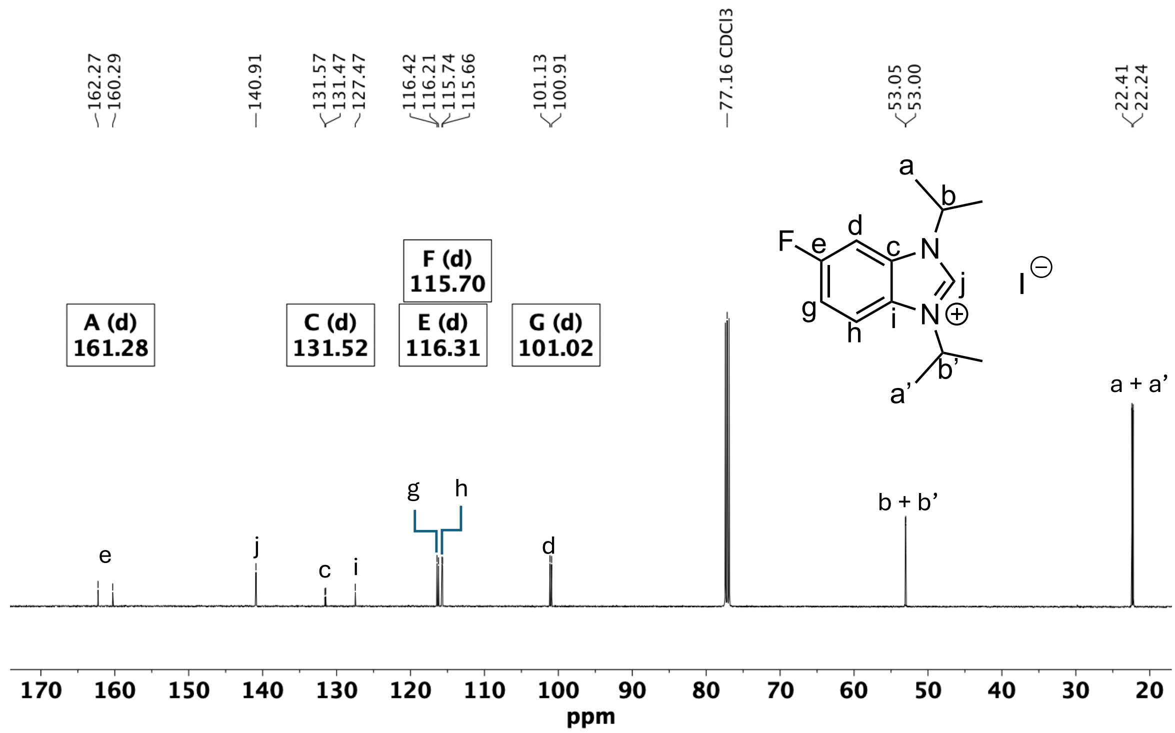

Figure S78. 126 MHz^{13}C spectrum of **3b**.

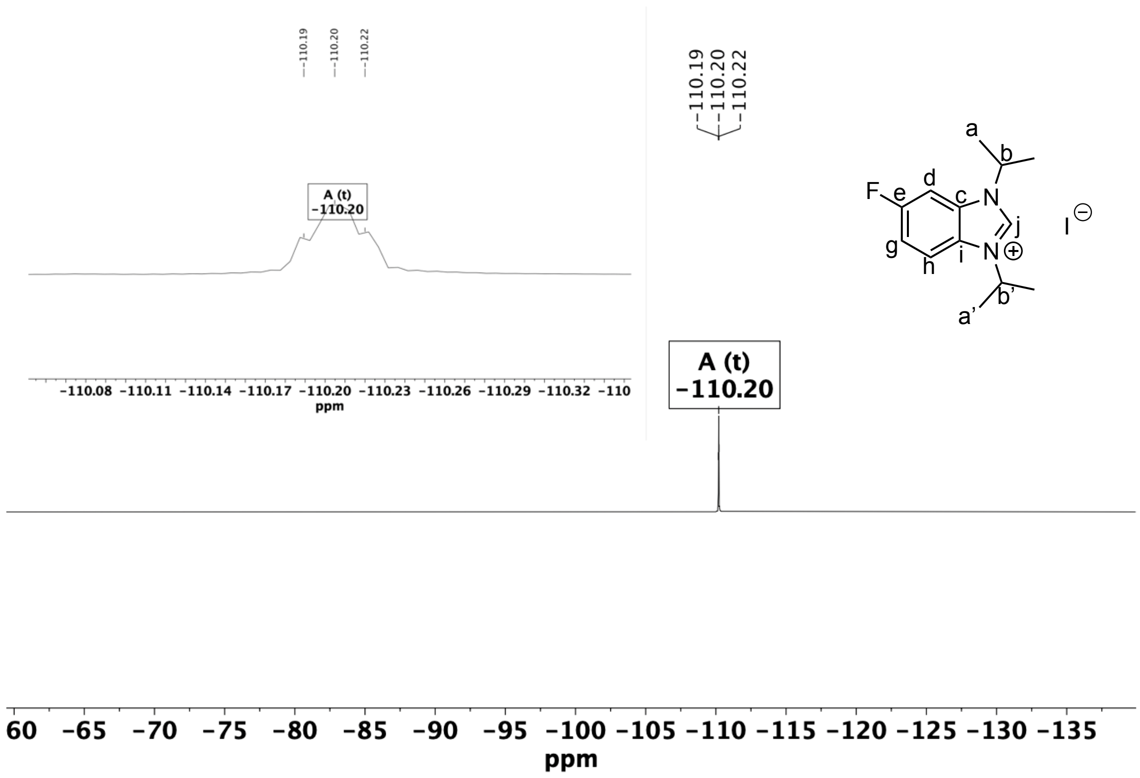

Figure S79. 565 MHz ^{19}F NMR spectrum of **3b**.

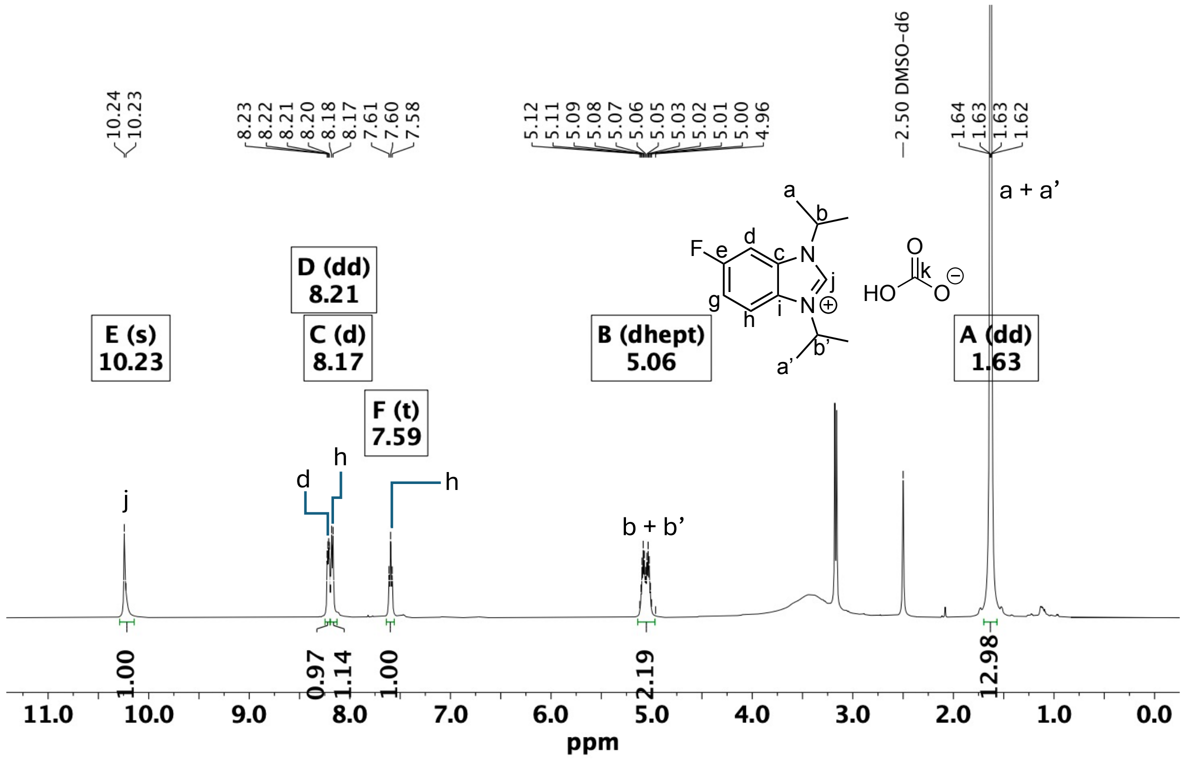

Figure S80. 500 MHz ^{1}H spectrum of **3c**.

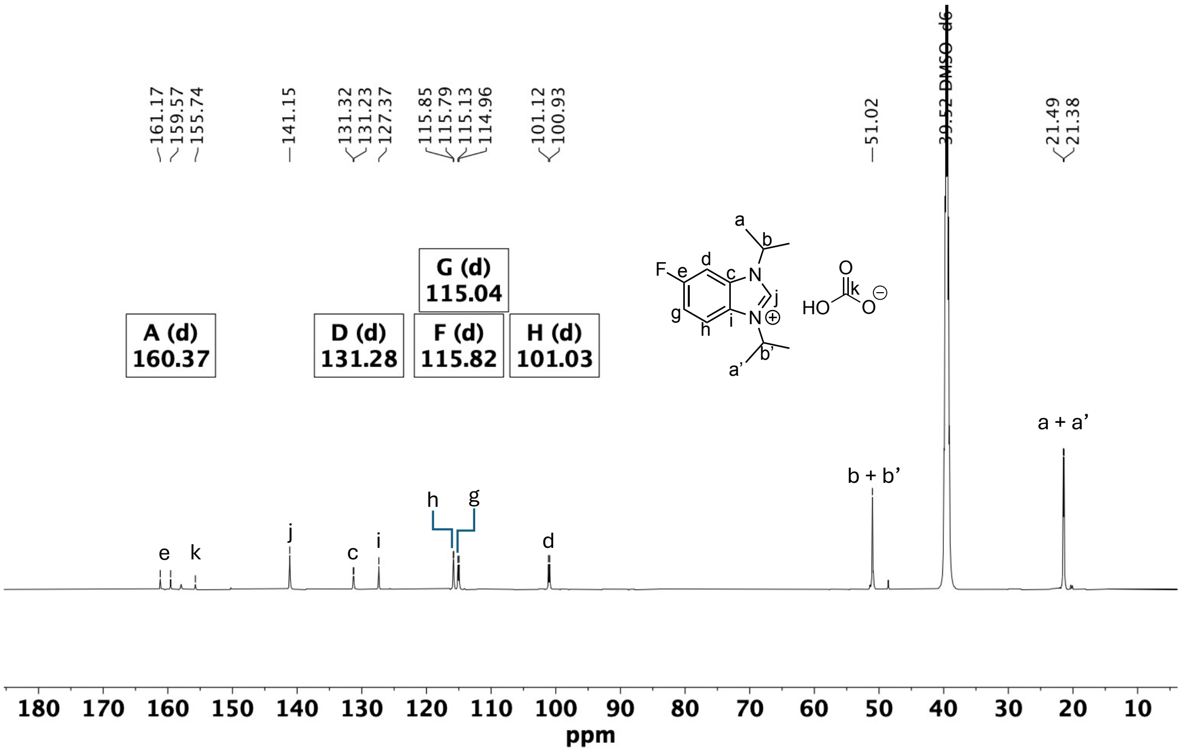

Figure S81. 126 MHz ^{13}C spectrum of **3c**.

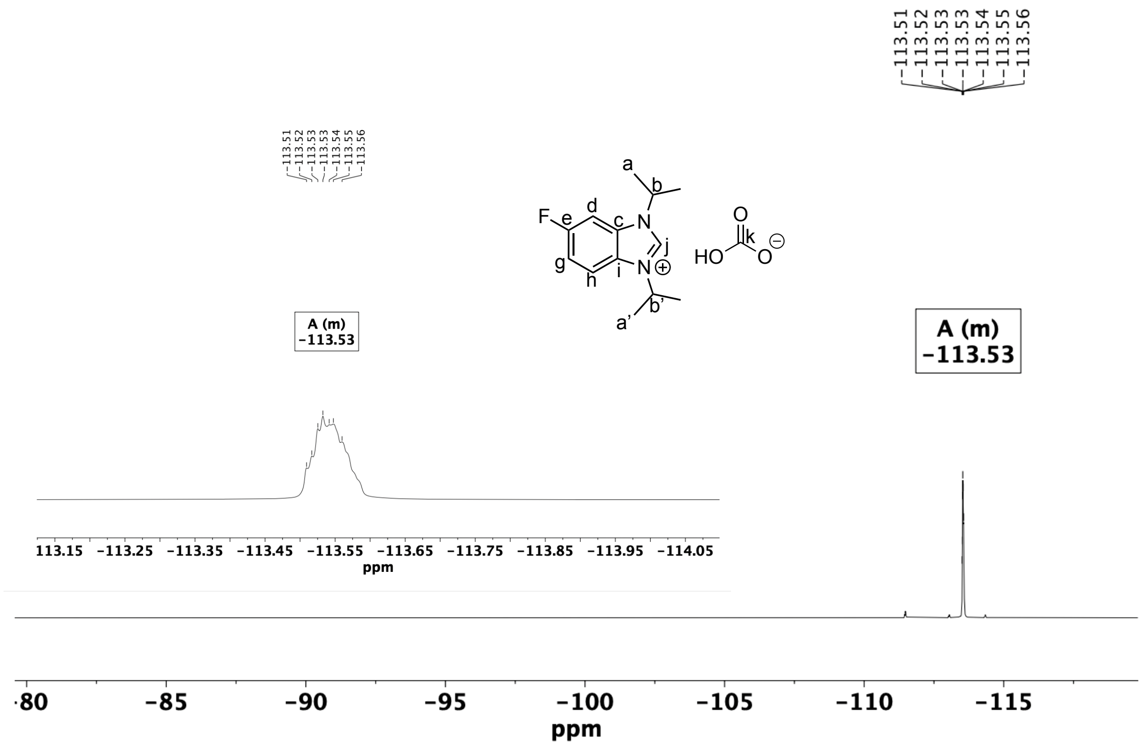

Figure S82. 565 MHz ^{19}F NMR spectrum of **3c**.

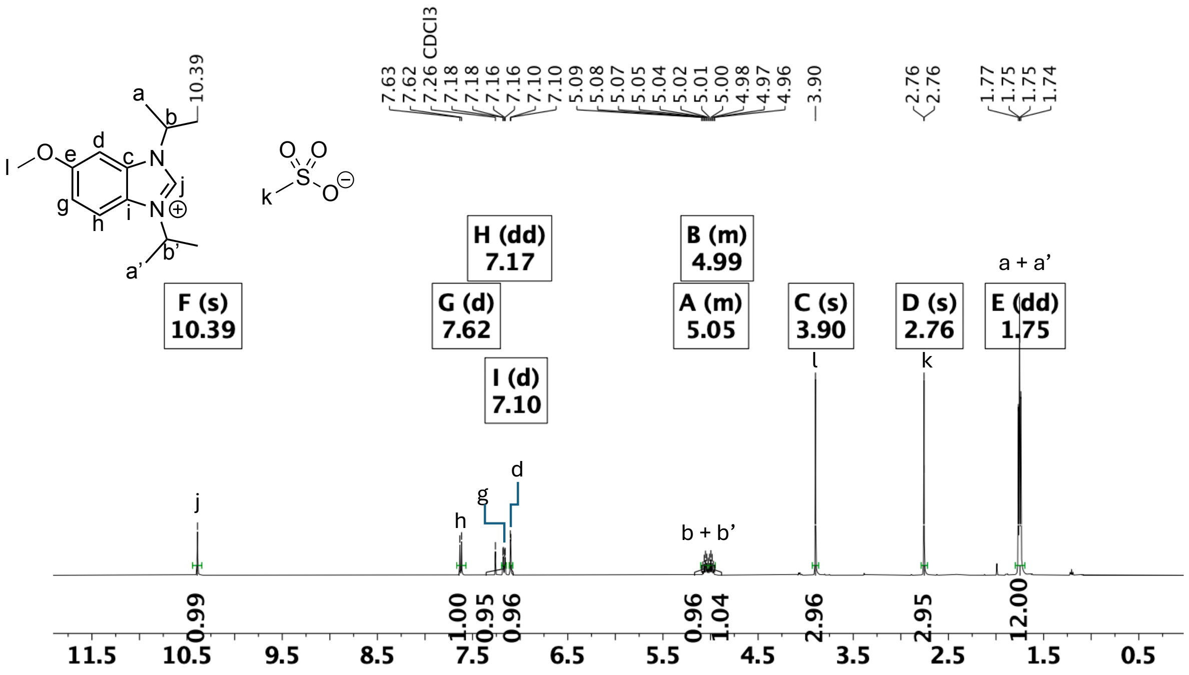

Figure S83. 500 MHz ^{1}H spectrum of 4a.

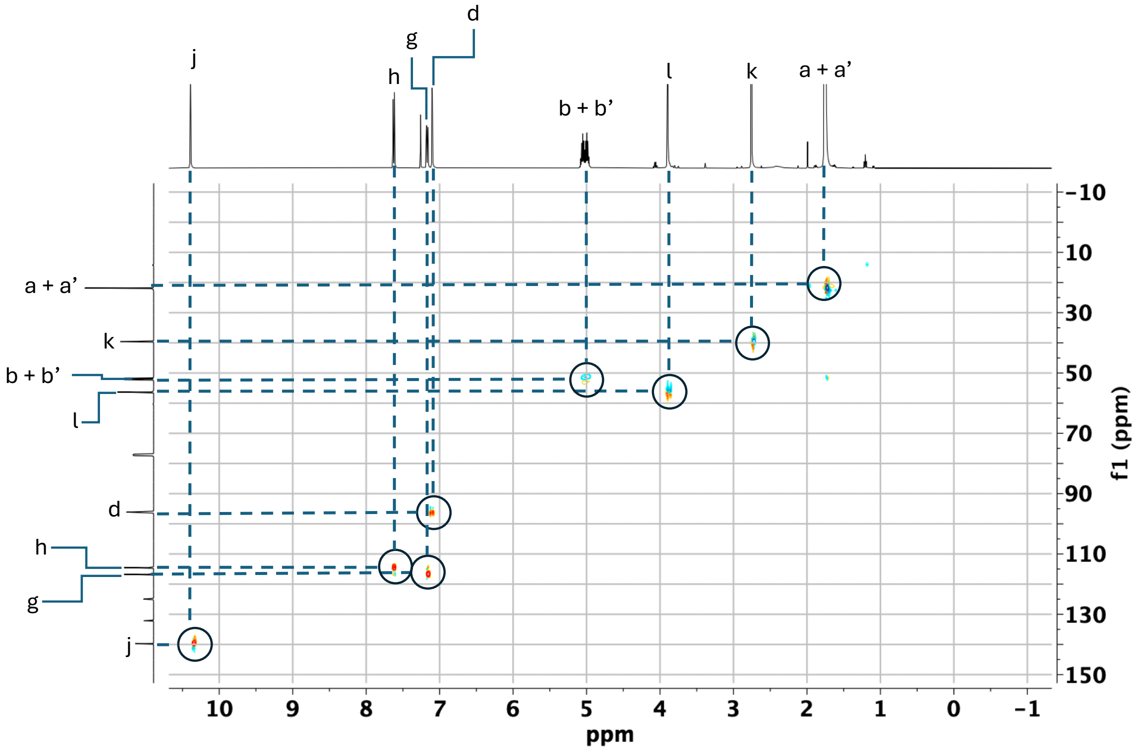

Figure S84. ^{1}H-^{13}C HSQC spectrum of 4a.

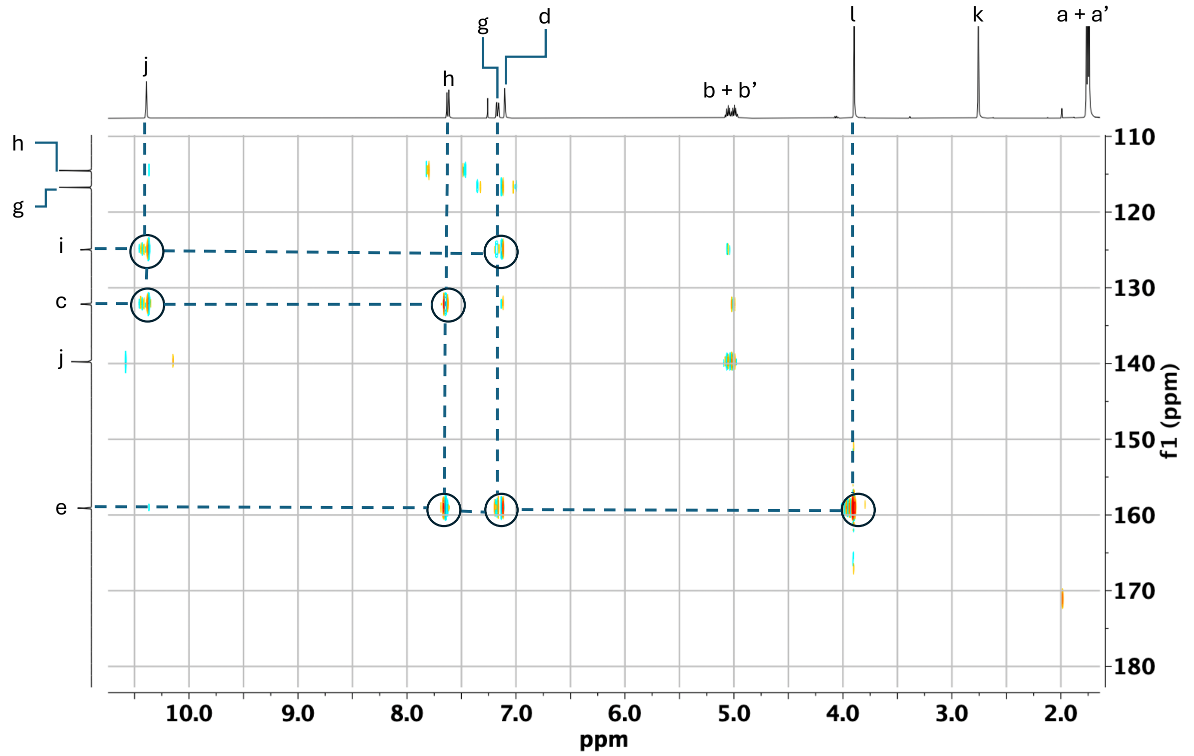

Figure S85. ^1H-^{13}C HMBC spectrum of **4a**.

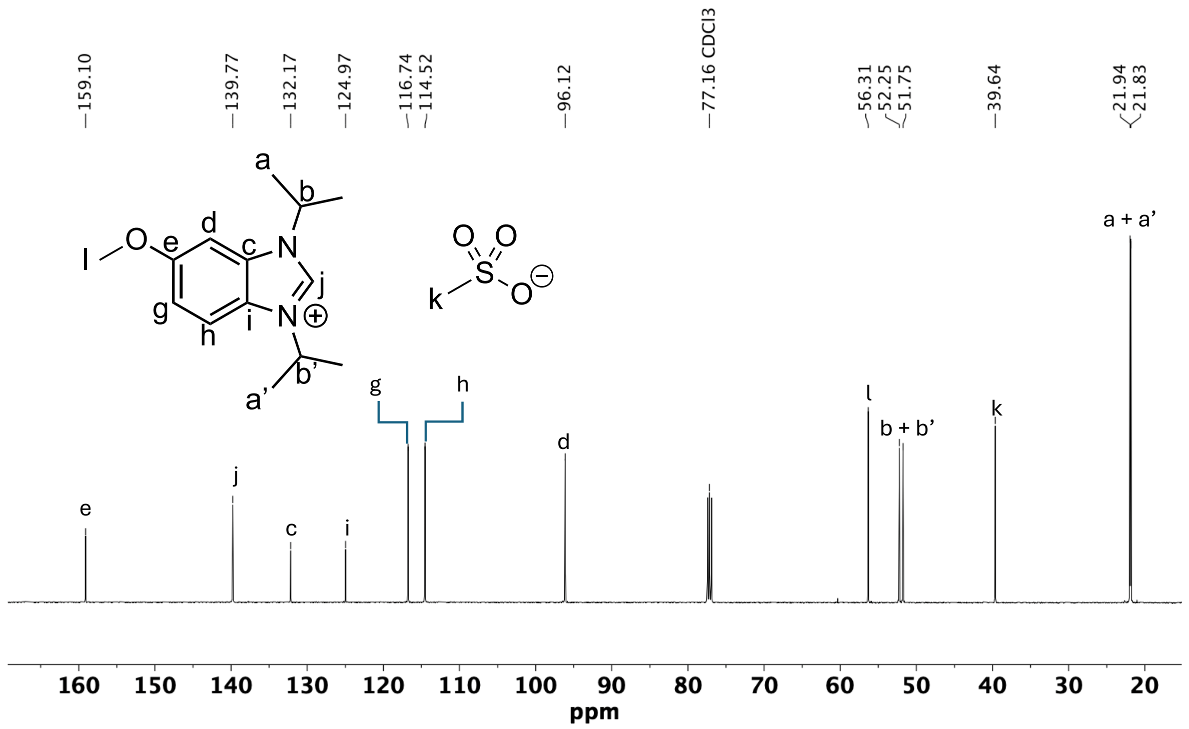

Figure S86. 126 MHz ^{13}C spectrum of **4a**.

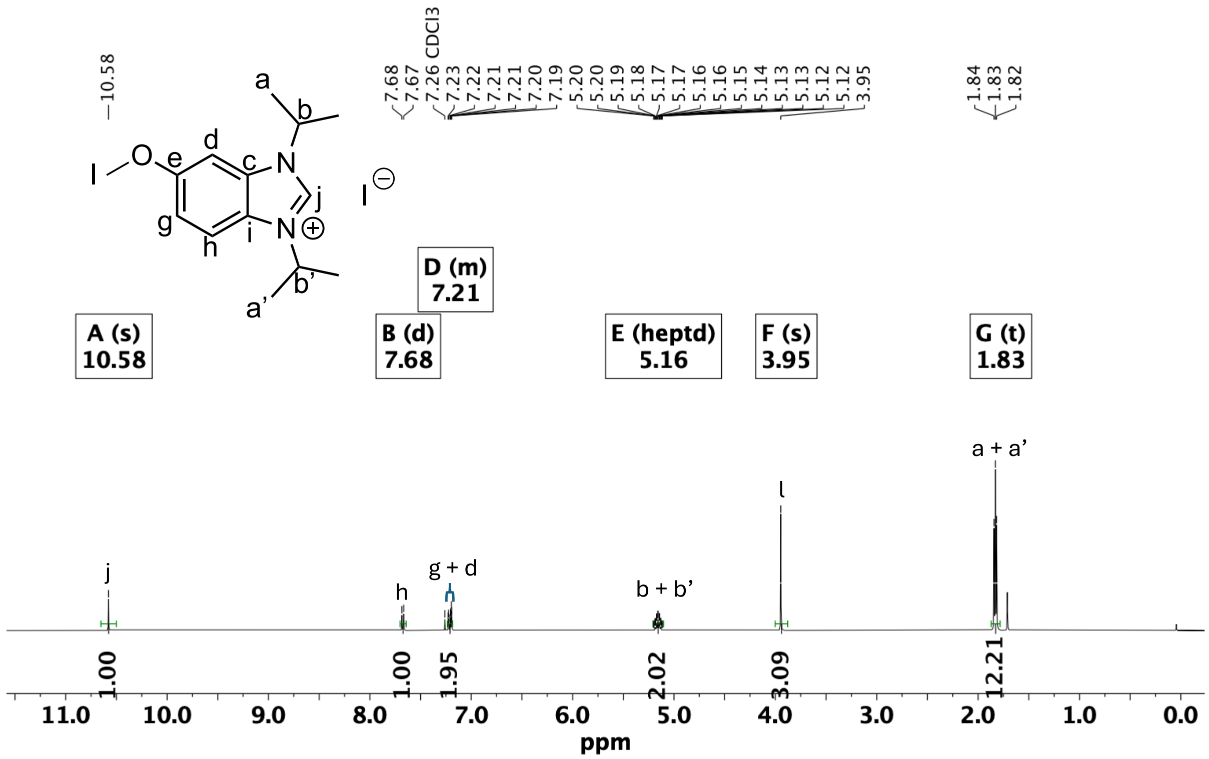

Figure S87. 500 MHz ^{1}H spectrum of **4b**.

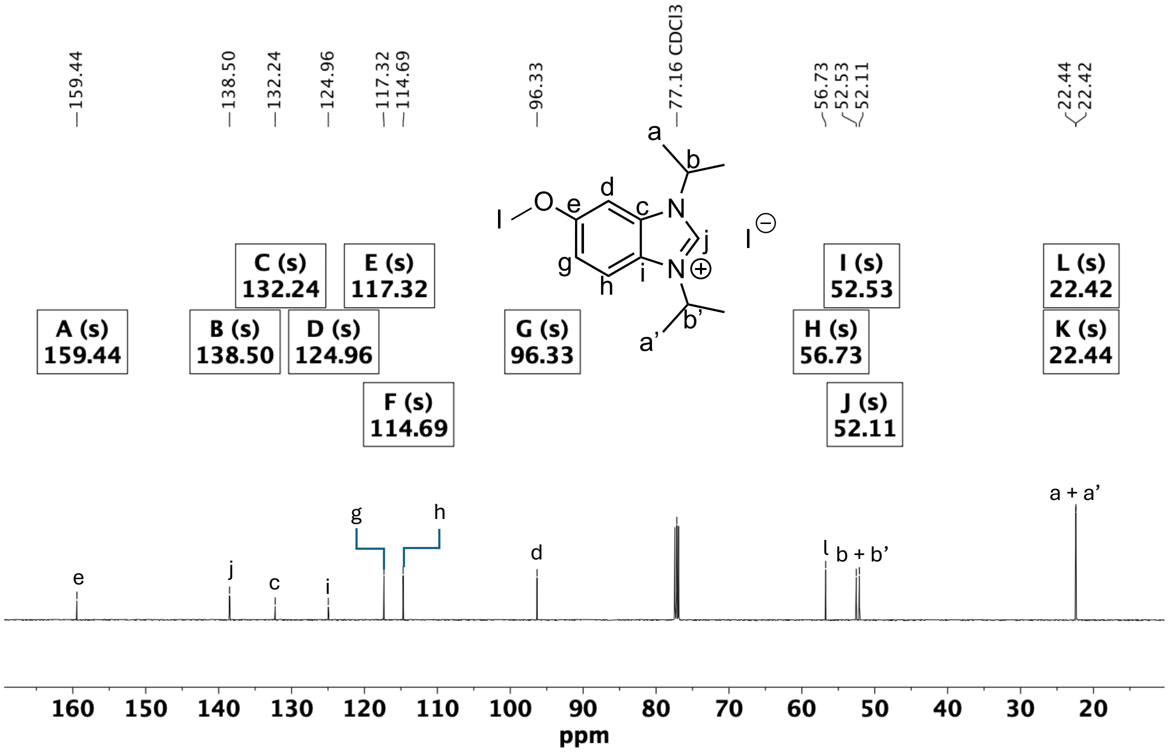

Figure S88. 126 MHz ^{13}C spectrum of **4b**.

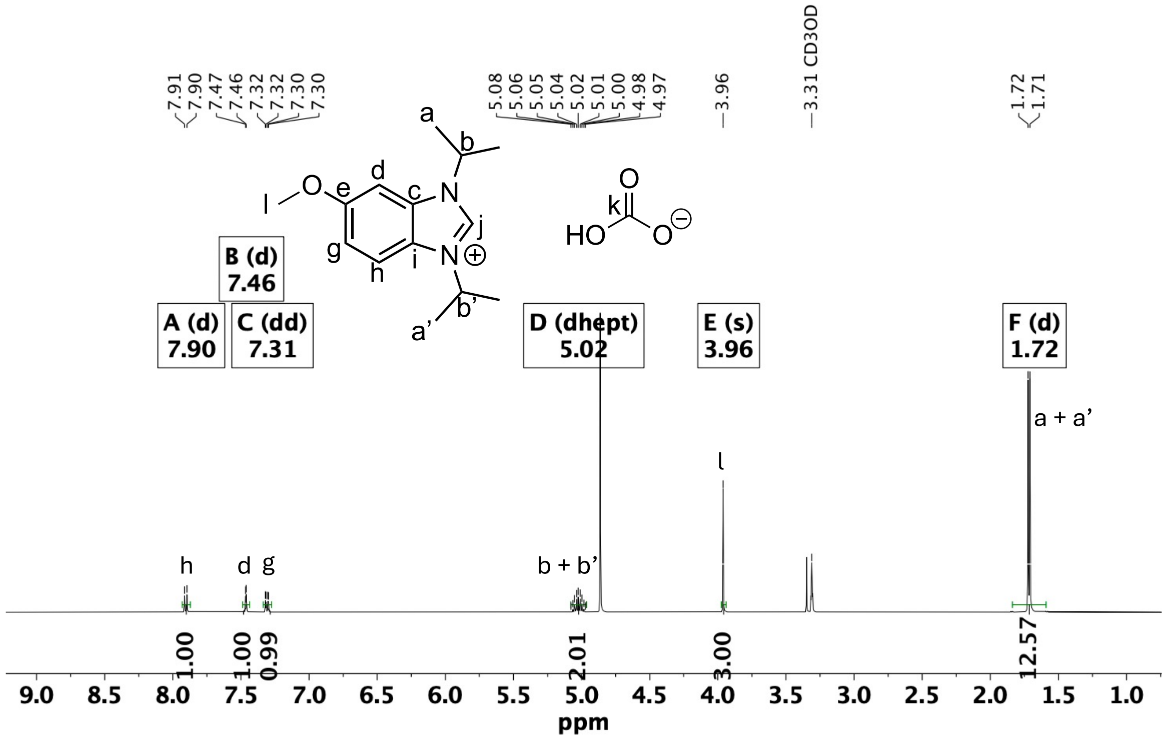

Figure S89. 500 MHz ^{1}H spectrum of **4c**.

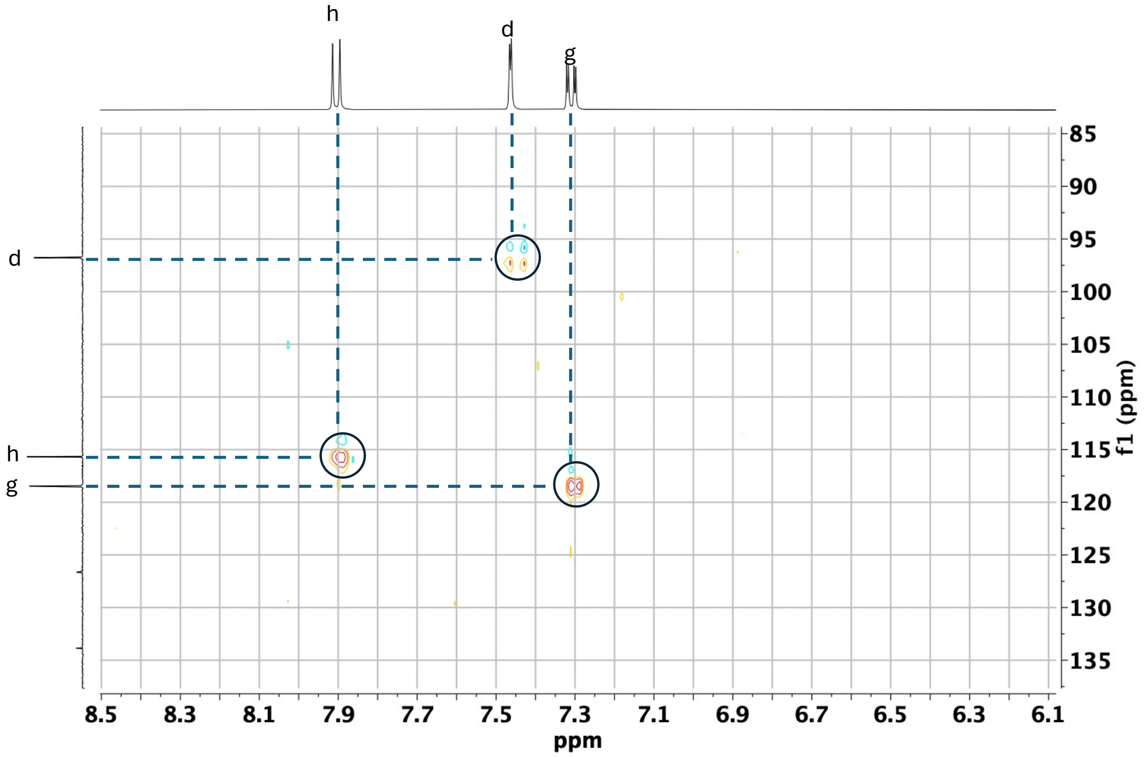

Figure S90. Zoomed in ^{1}H-^{13}C HSQC spectra of **4c**.

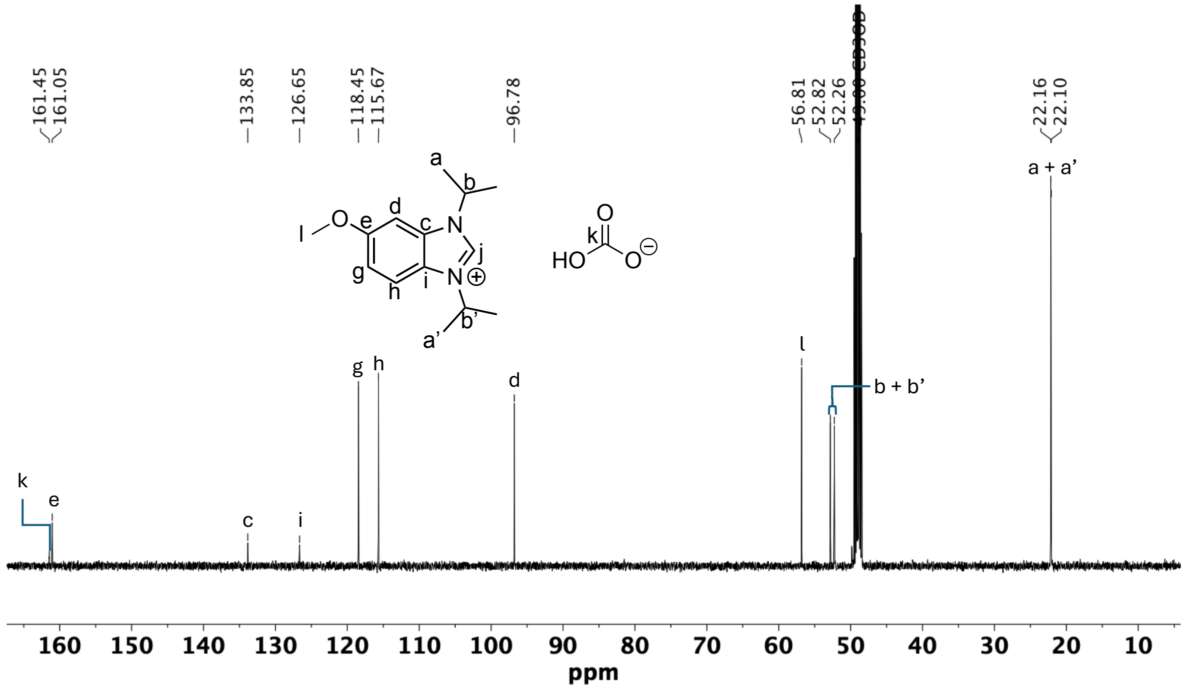

Figure S91. 126 MHz ^{13}C spectrum of **4c**.

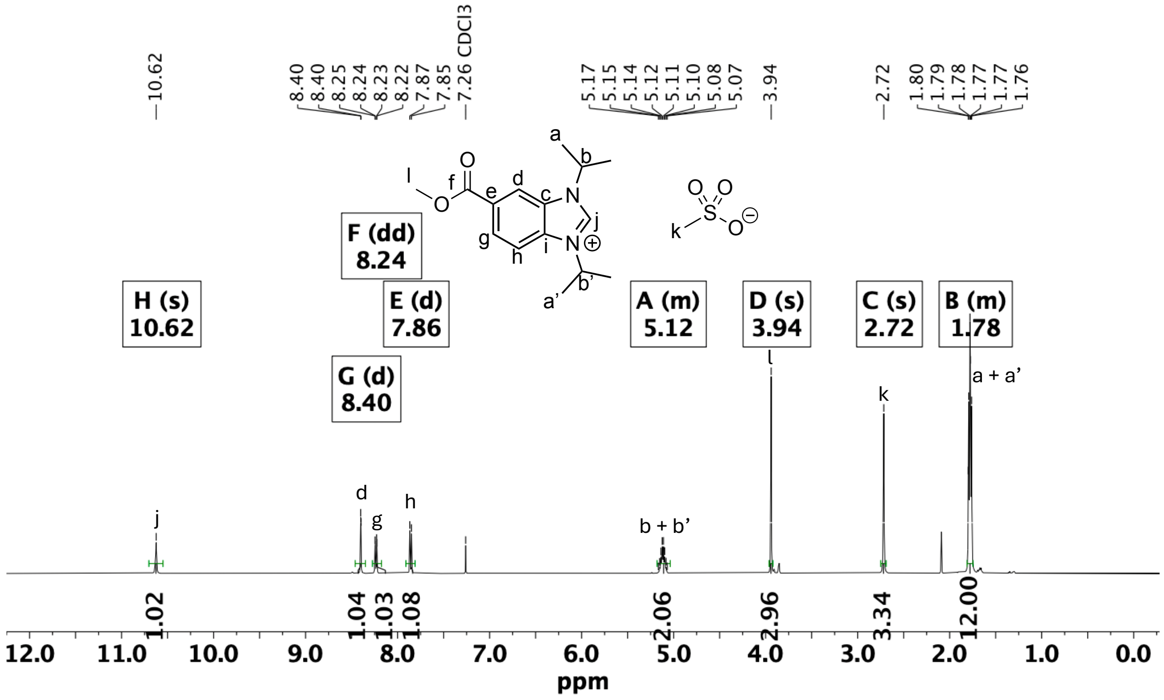

Figure S92. 500 MHz ^{1}H spectrum of **5a**.

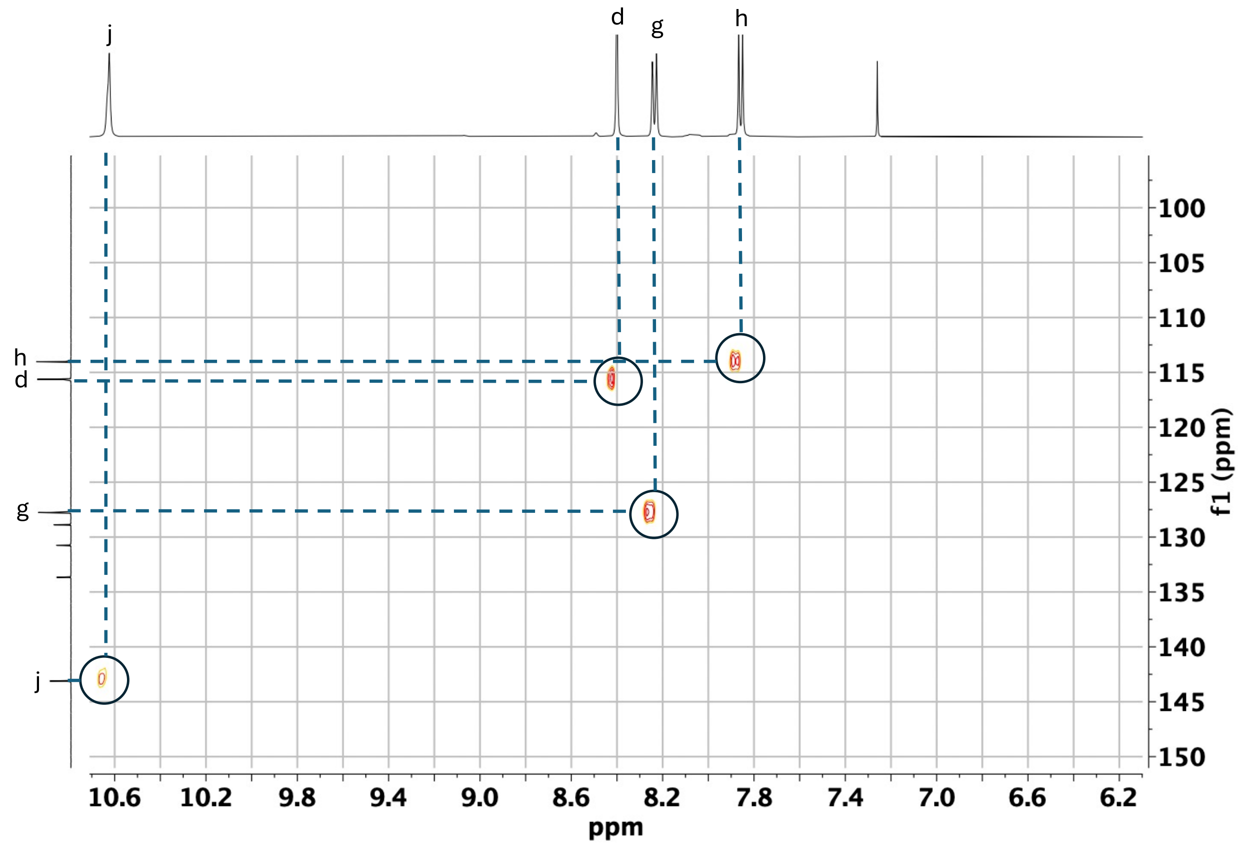

Figure S93. Zoomed in ^{1}H-^{13}C HSQC spectrum of **5a**.

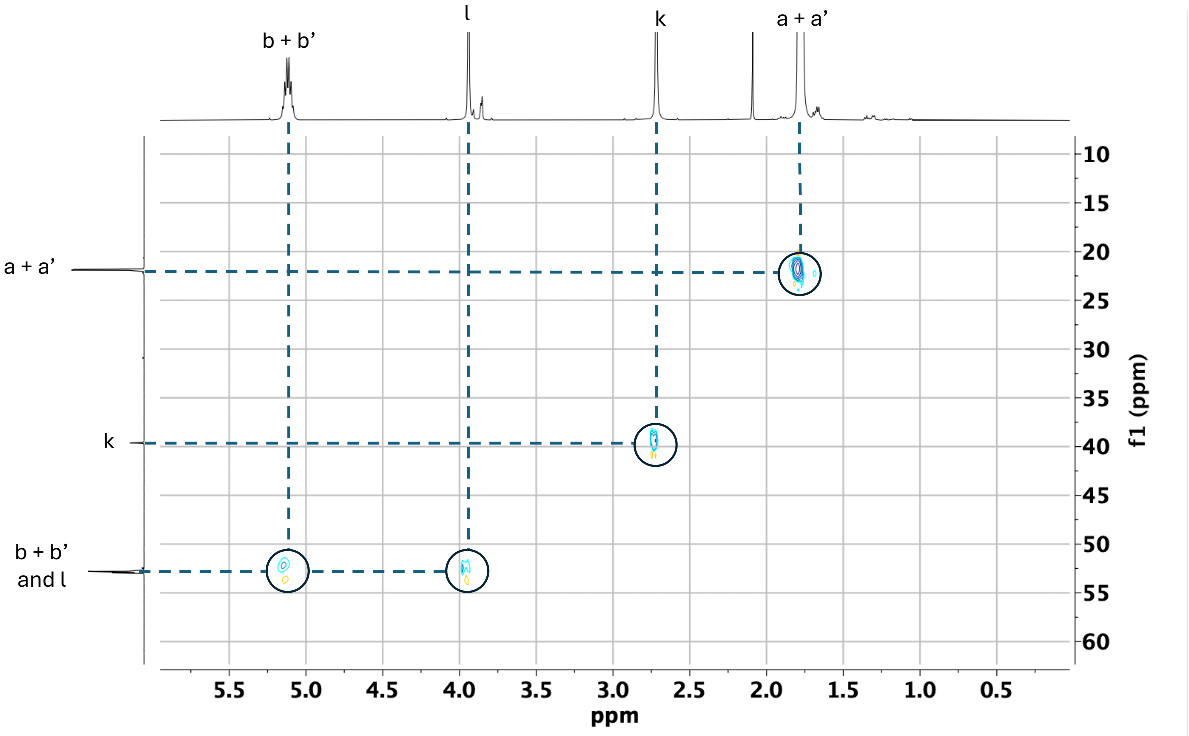

Figure S94. Zoomed in ^{1}H-^{13}C HSQC spectrum of **5a**.

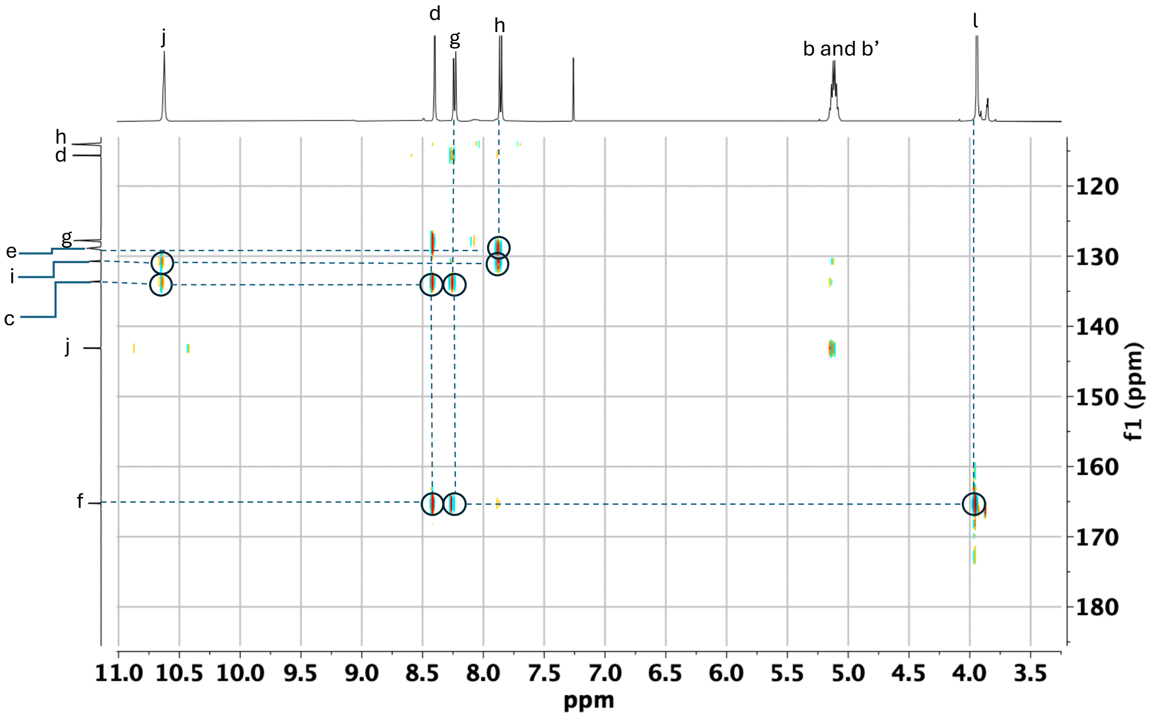

Figure S95. Zoomed in ^{1}H-^{13}C HMBC spectrum of **5a**.

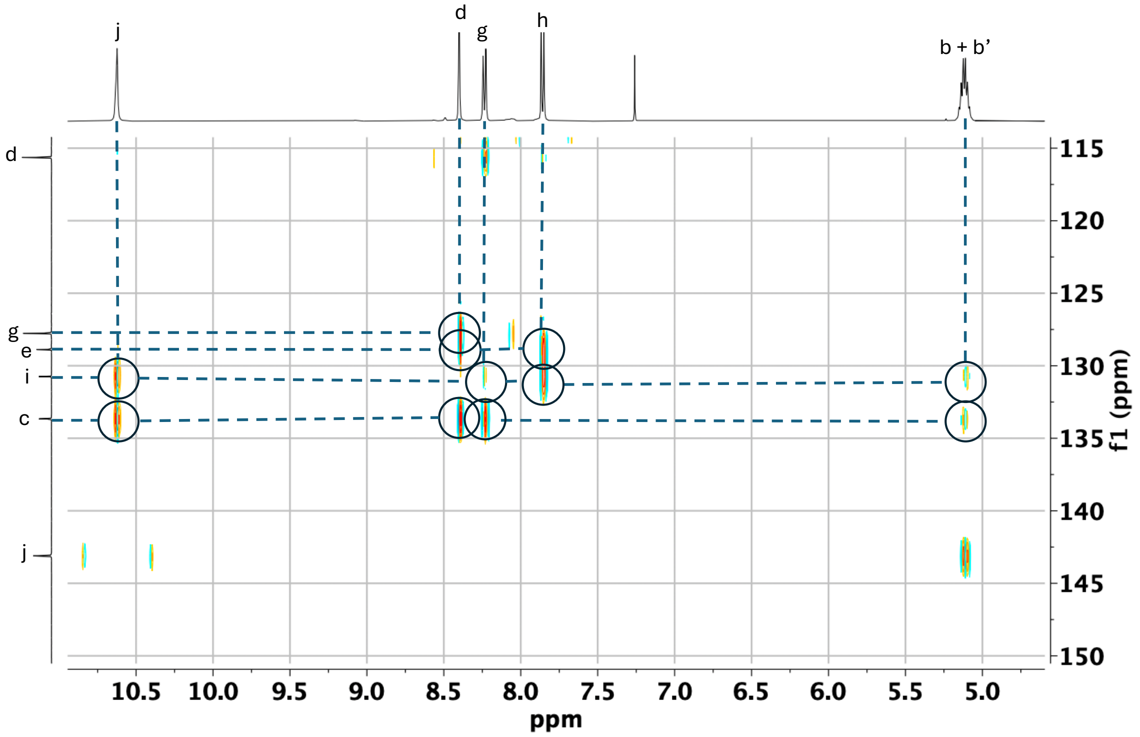

Figure S96. Zoomed in ^{1}H-^{13}C HMBC spectrum of **5a**.

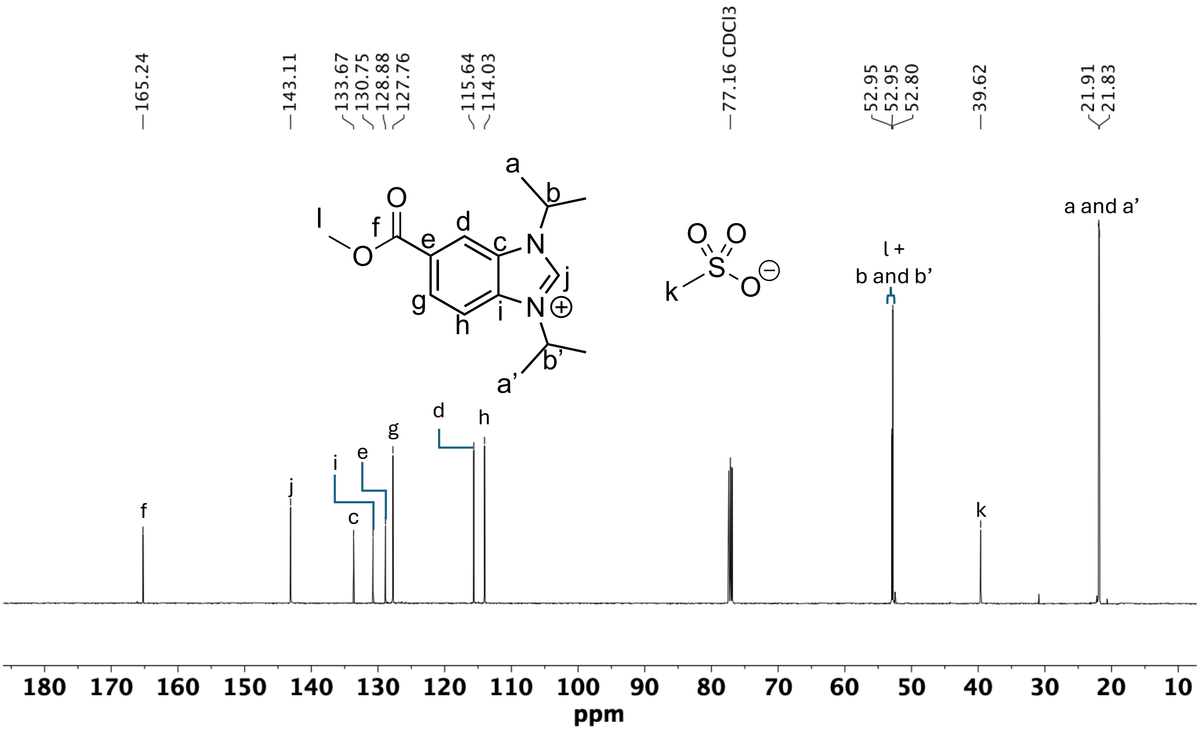

Figure S97. 126 MHz ^{13}C spectrum of **5a**.

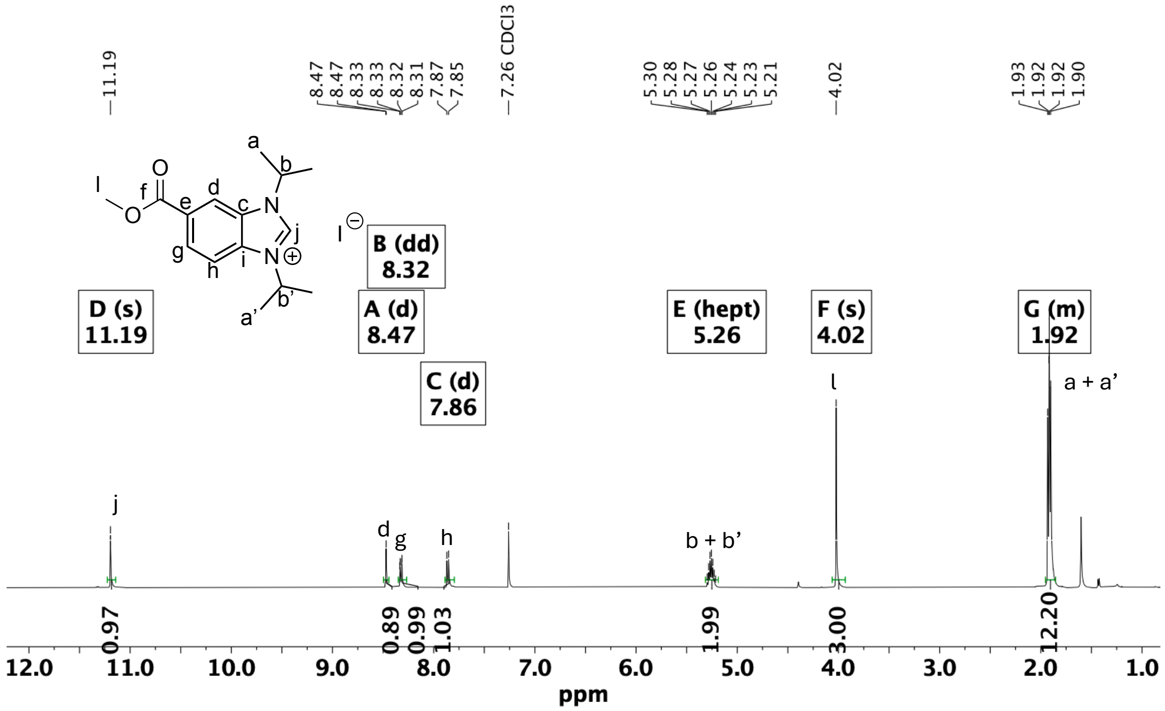

Figure S98. 500 MHz ^{1}H spectrum of **5b**.

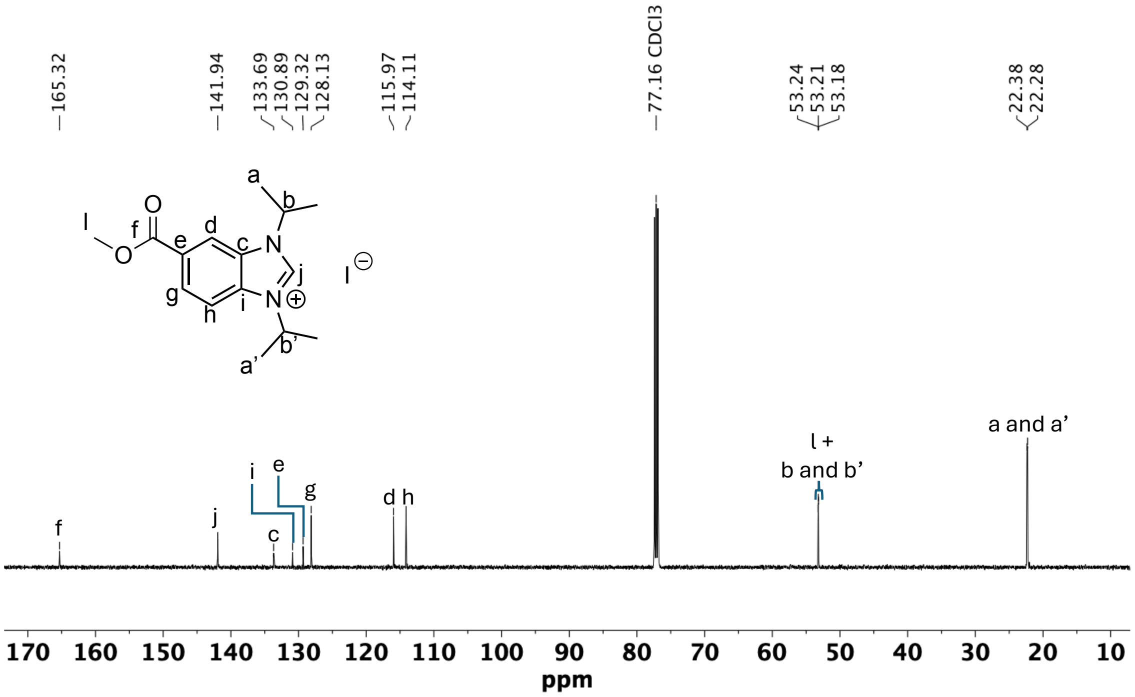

Figure S99. 126 MHz ^{13}C spectrum of **5b**.

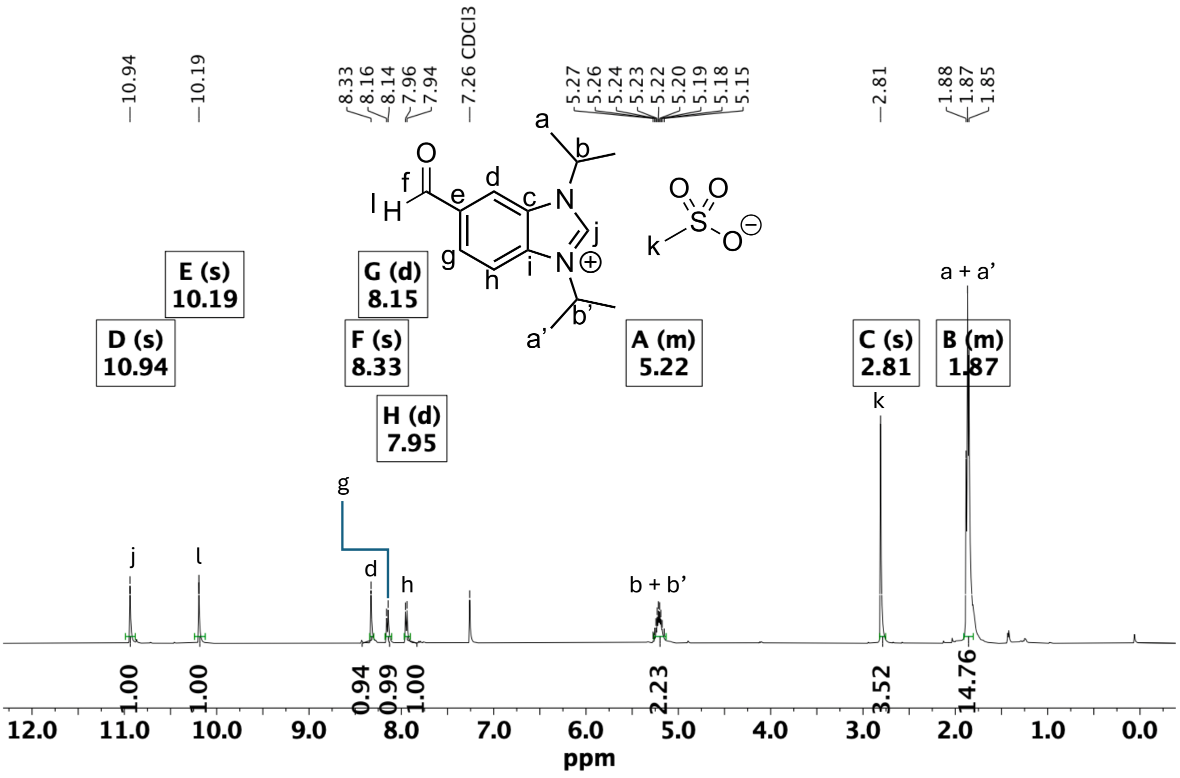

Figure S100. 500 MHz ^{1}H spectrum of **6a**.

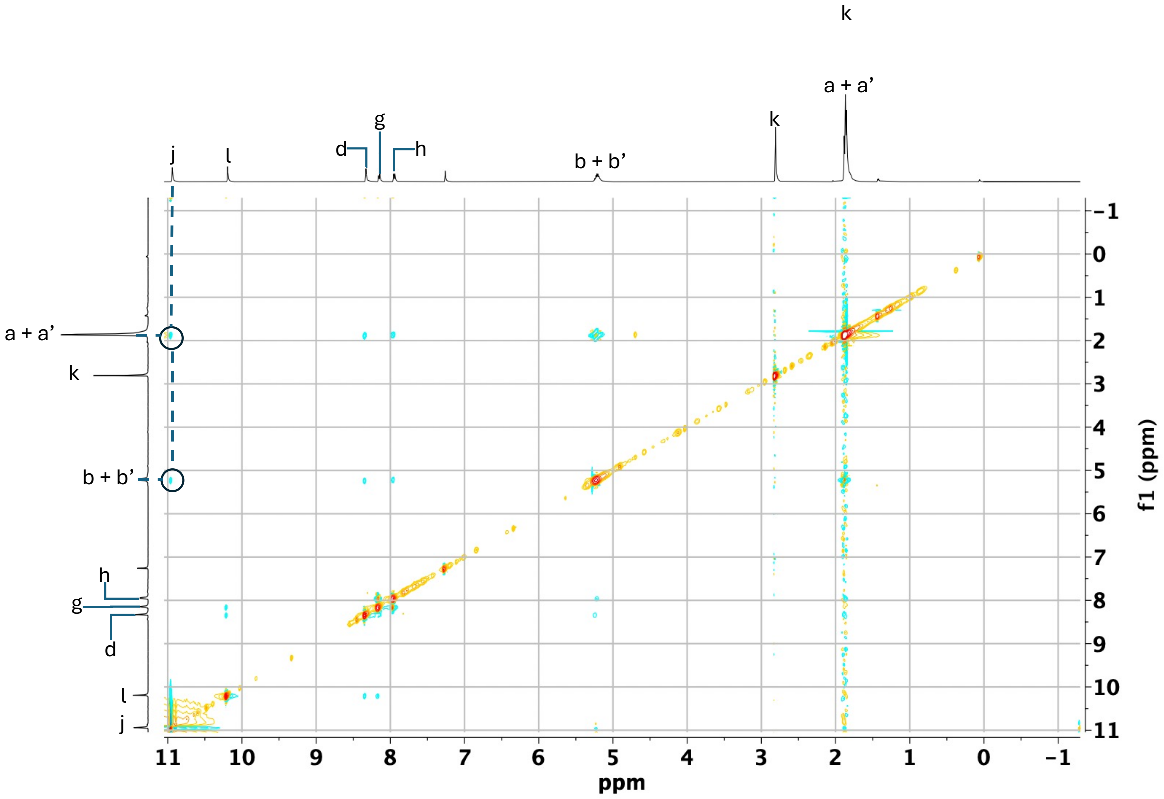

Figure S101. ^{1}H-^{1}H NOSEY spectrum of **6a**.

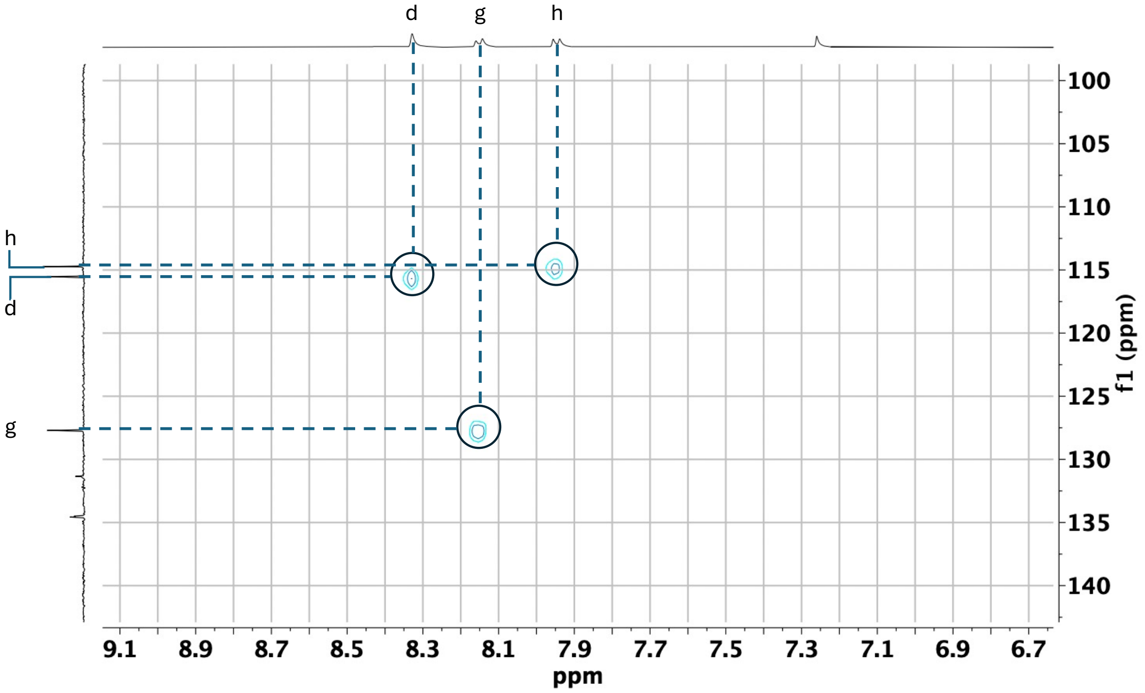

Figure S102. Zoomed in ^{1}H-^{13}C HSQC spectrum of **6a**.

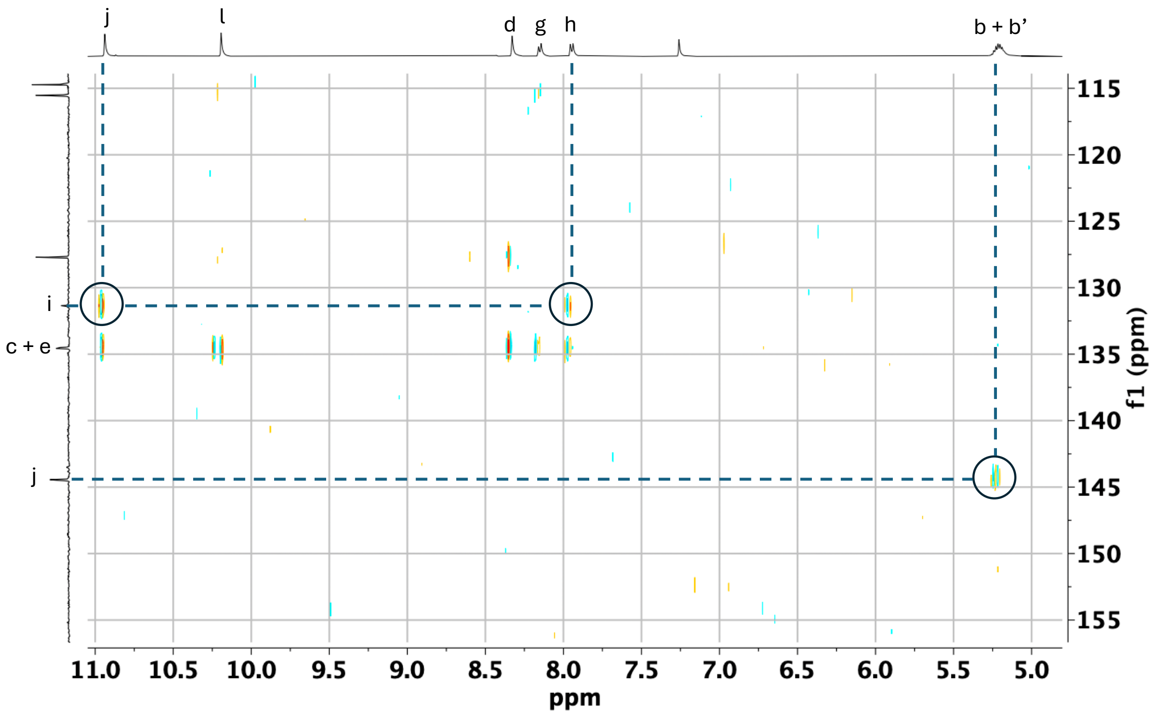

Figure S103. Zoomed in HMBC spectrum of **6a**.

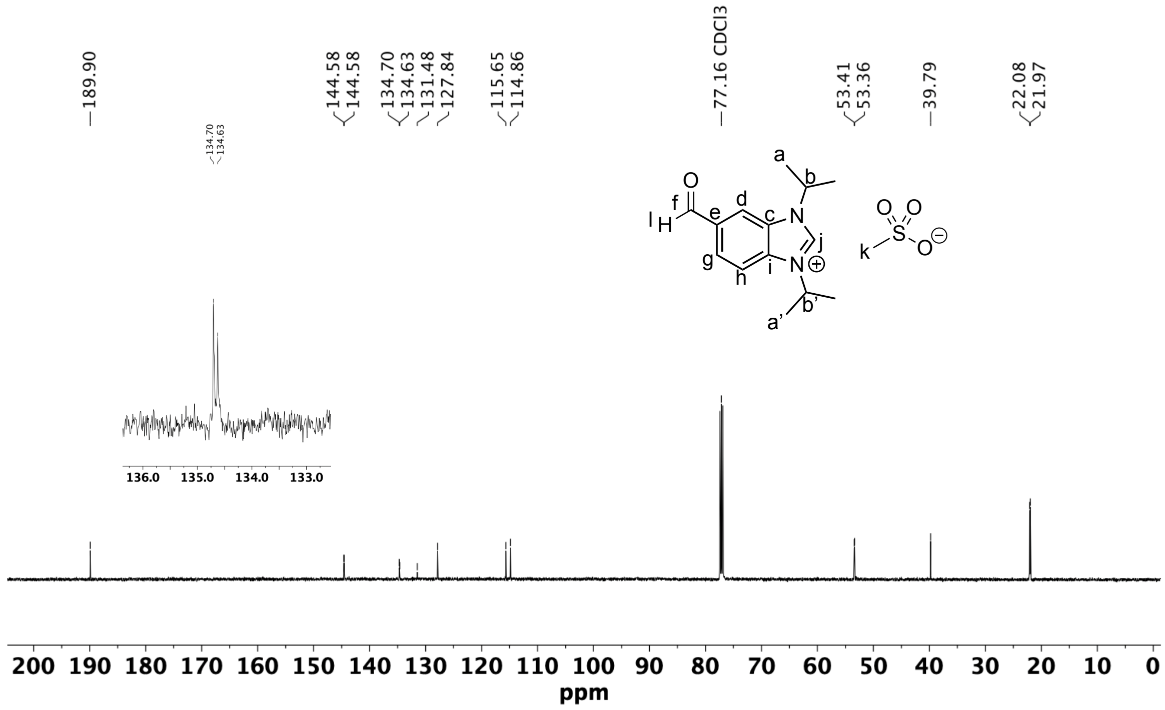

Figure S104. 126 MHz ^{13}C spectrum of **6a**.

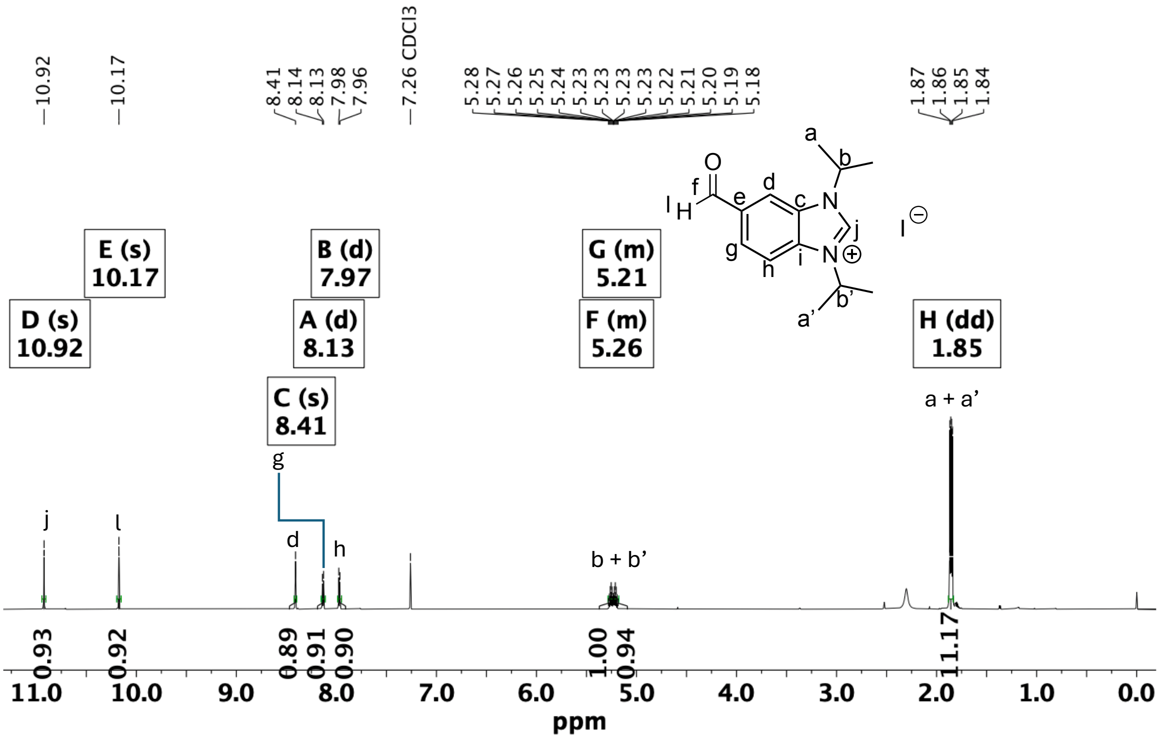

Figure S105. 750 MHz ^{1}H spectrum of **6b**.

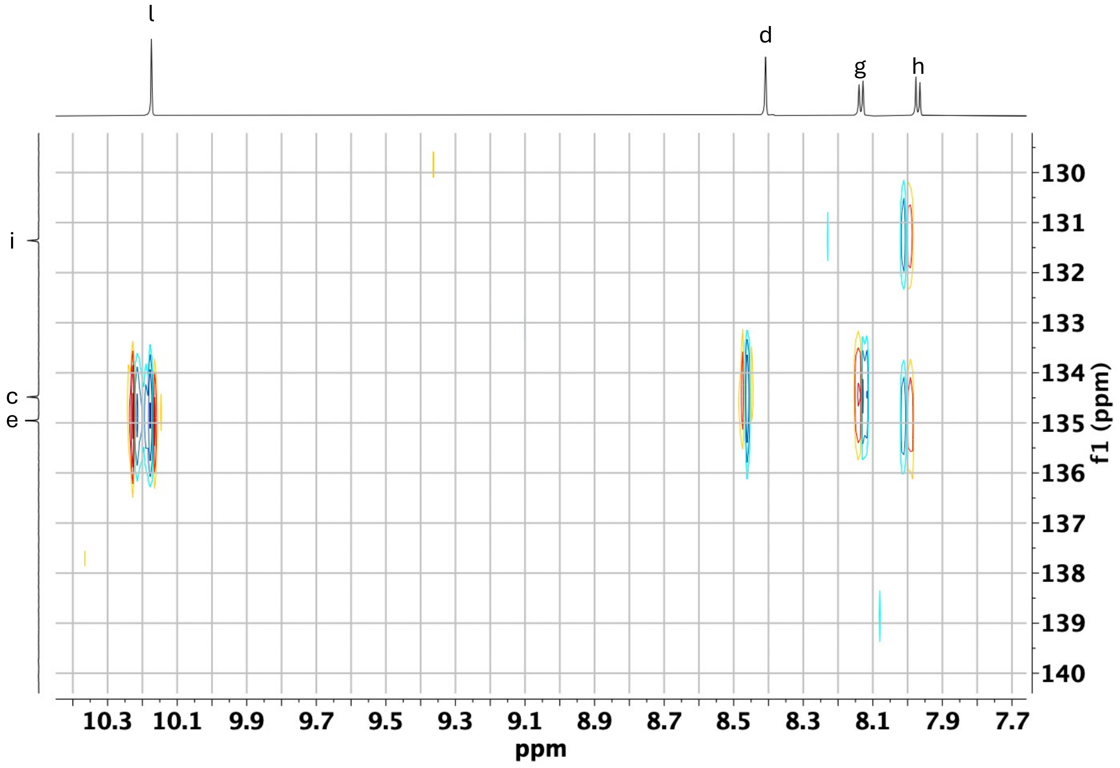

Figure S106. HMBC spectrum of **6b**.

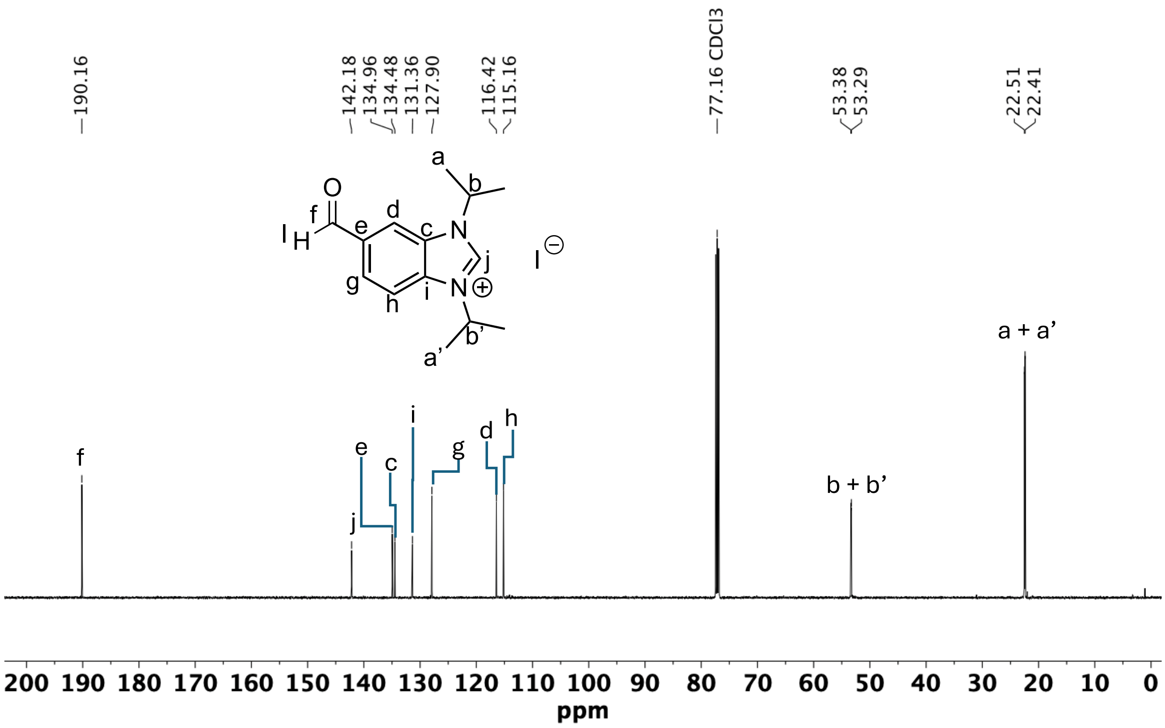

Figure S107. 126 MHz ^{13}C spectrum of **6b**.

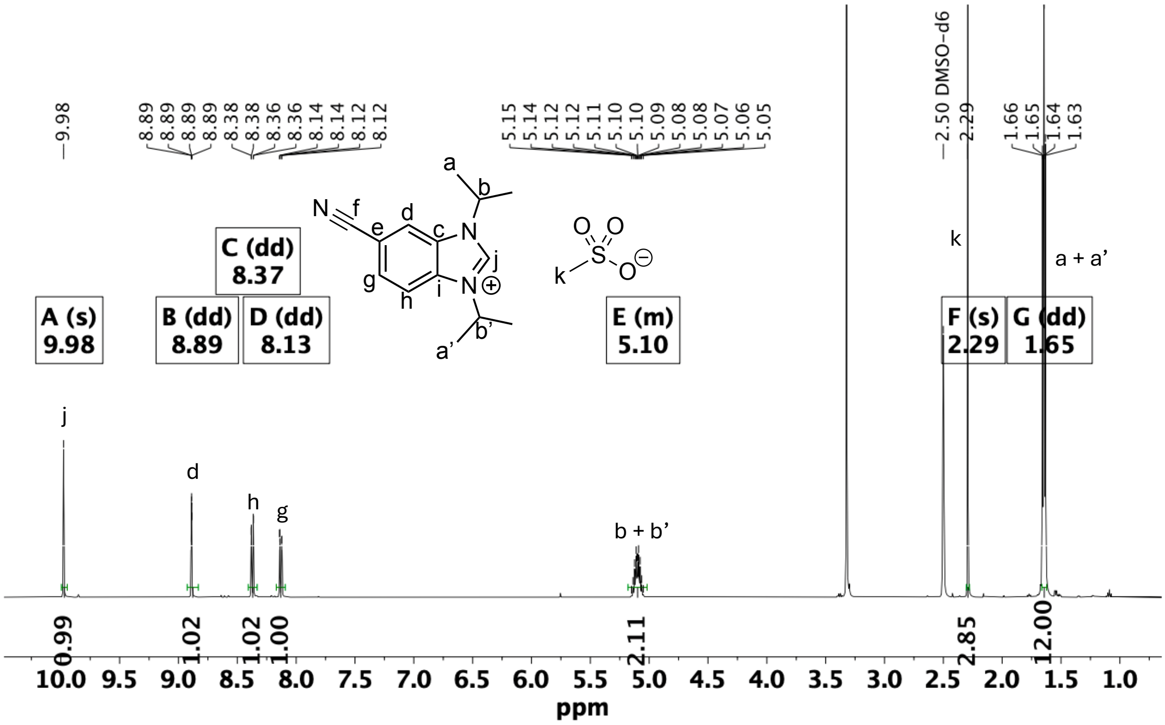

Figure S108. 500 MHz ^{1}H spectrum of **7a**.

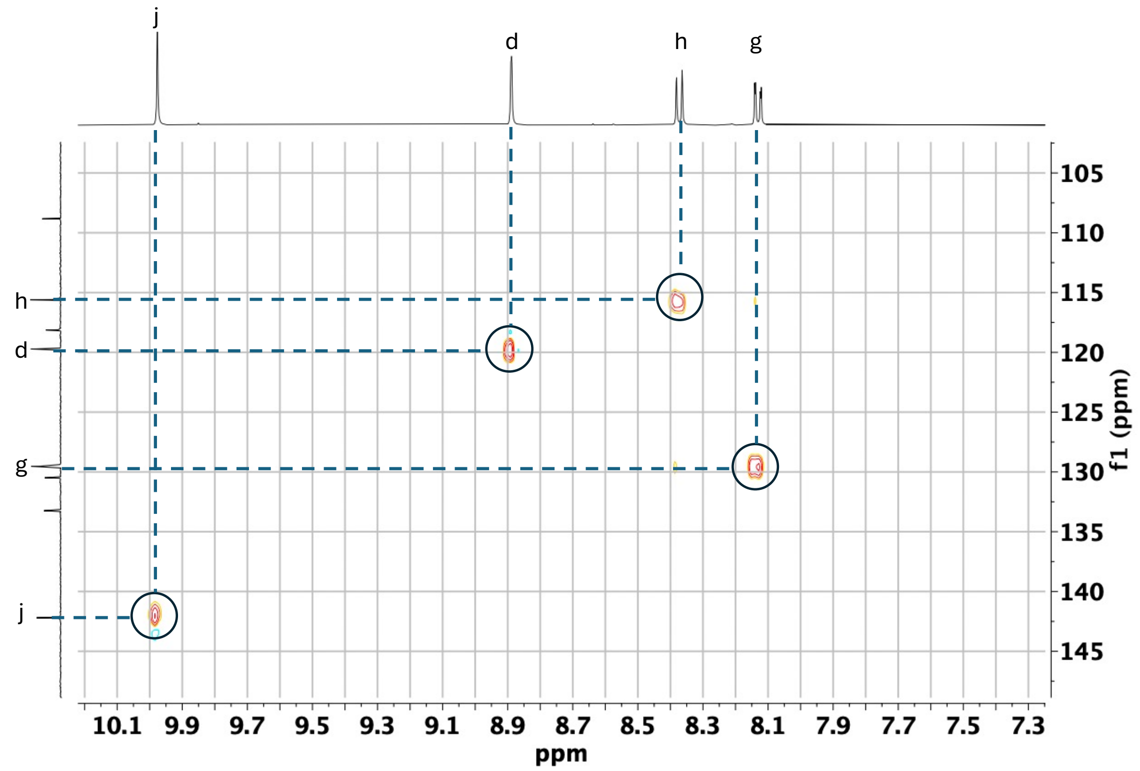

Figure S109. Zoomed in ^1H-^{13}C HSQC spectrum of **7a**.

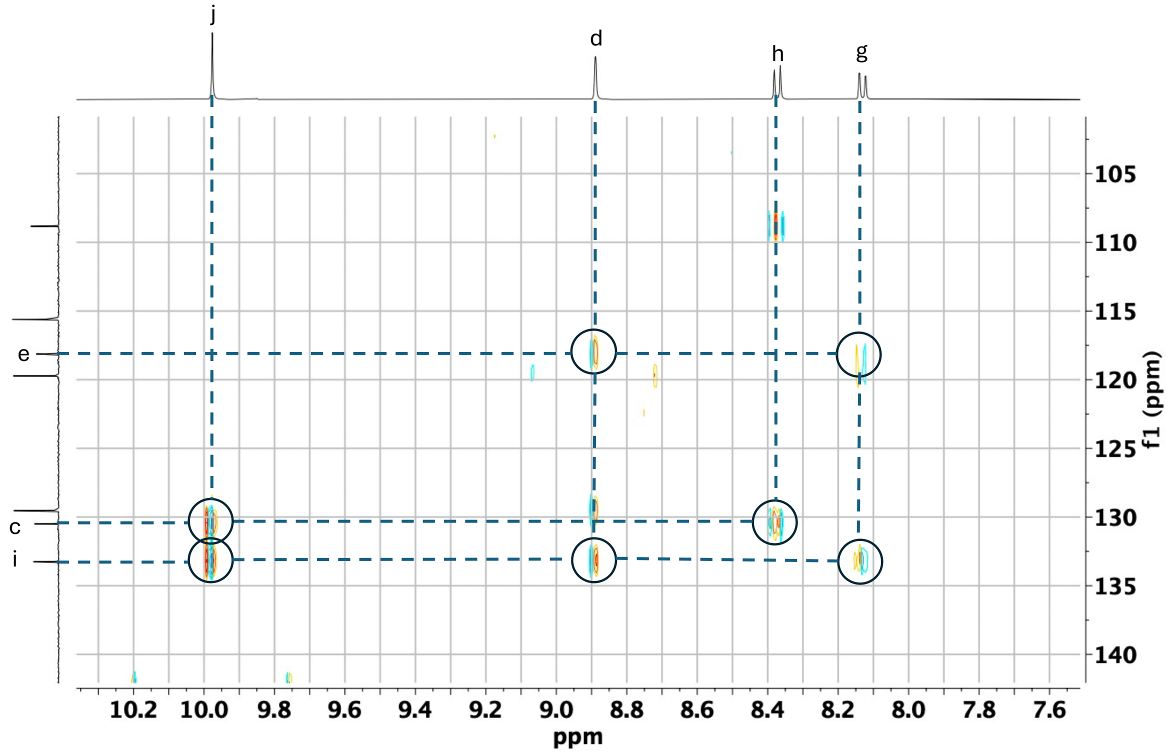

Figure S110. Zoomed in ^1H-^{13}C HMBC spectrum of **7a**.

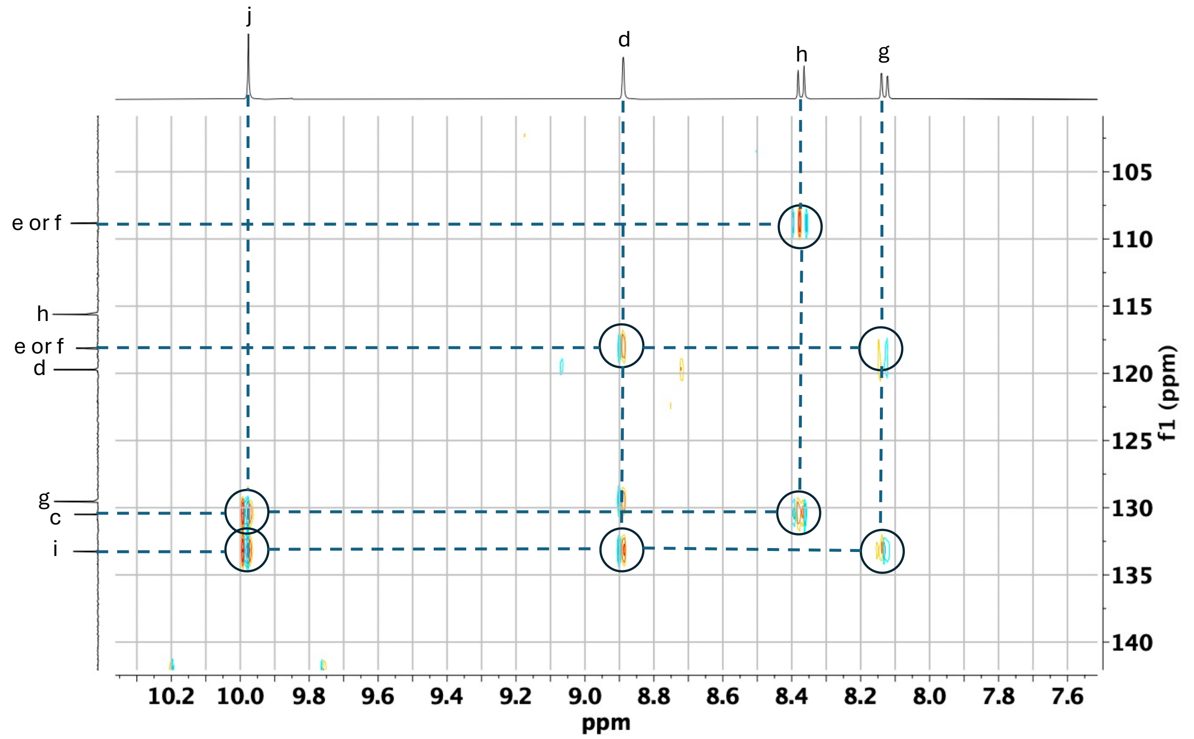

Figure S111. Zoomed in ^{1}H-^{13}C HSQC spectrum of **7a**.

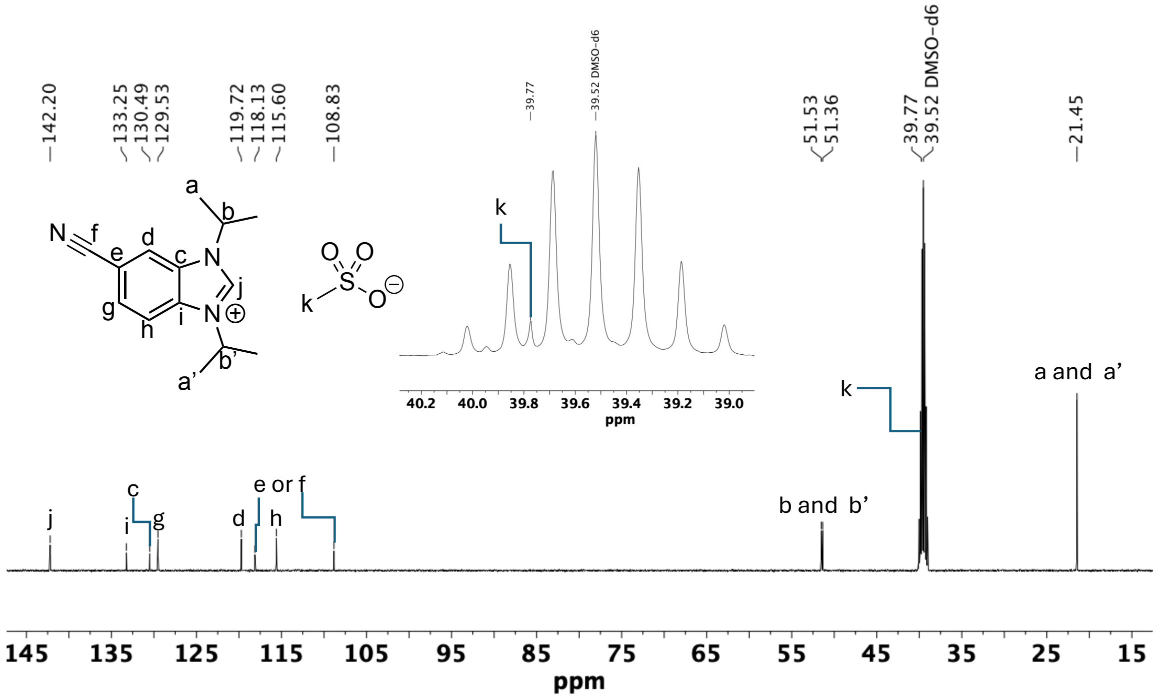

Figure S112. 126 MHz ^{13}C spectrum of **7a**.

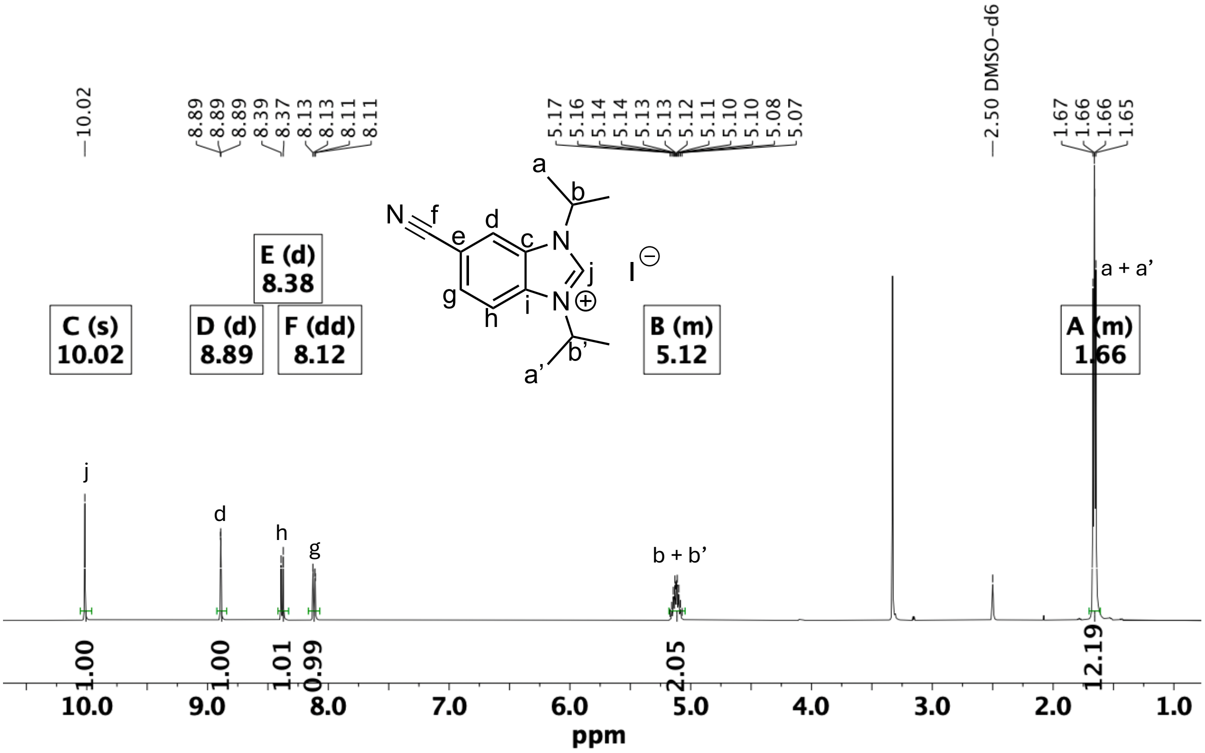

Figure S113. 500 MHz ^{1}H spectrum of **7b**.

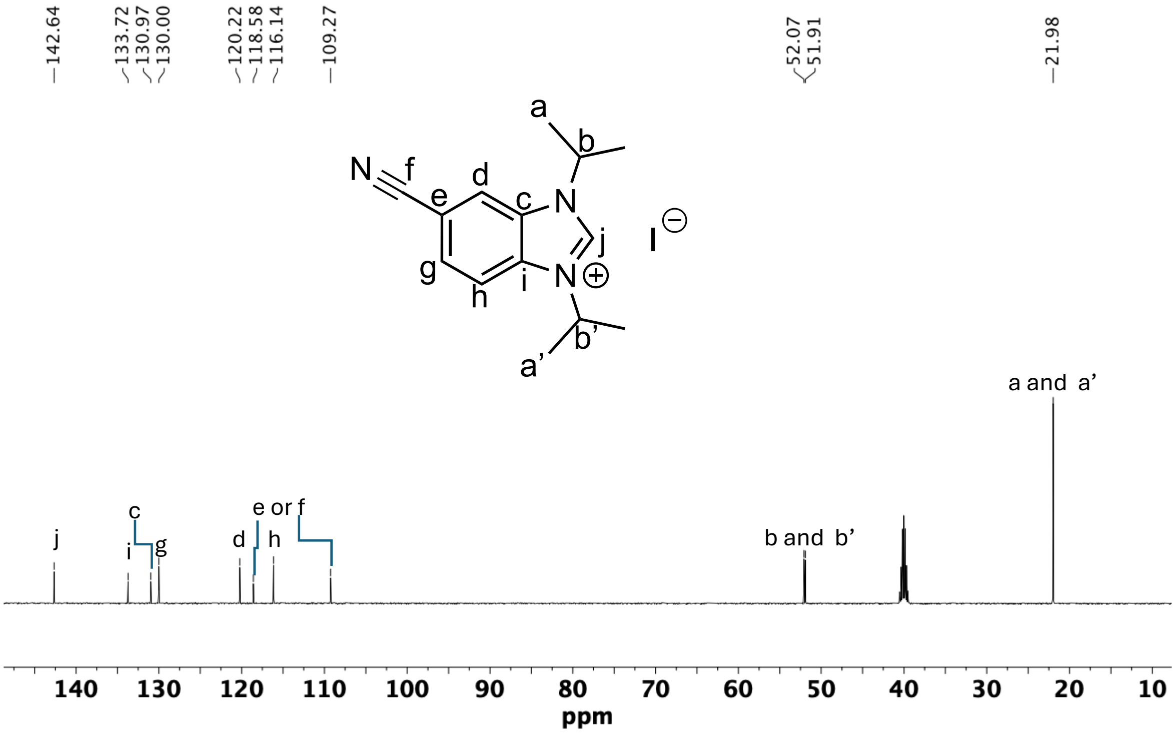

Figure S114. 500 MHz ^{13}C spectrum of **7b**.

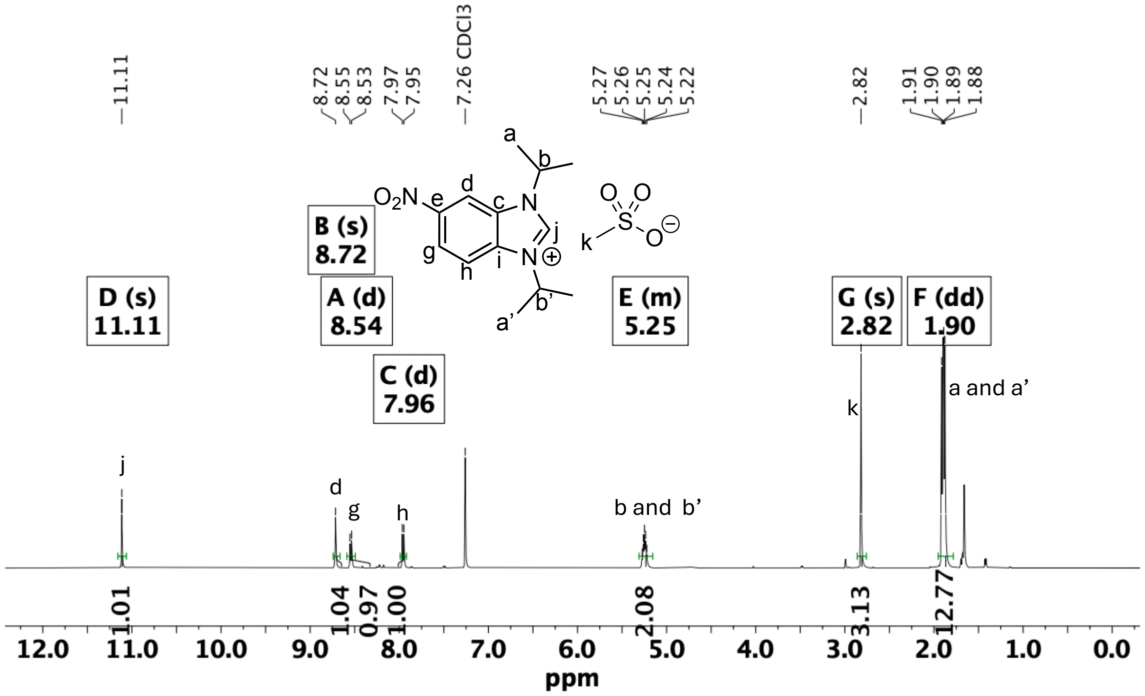

Figure S115. 500 MHz ^{1}H spectrum of **8a**.

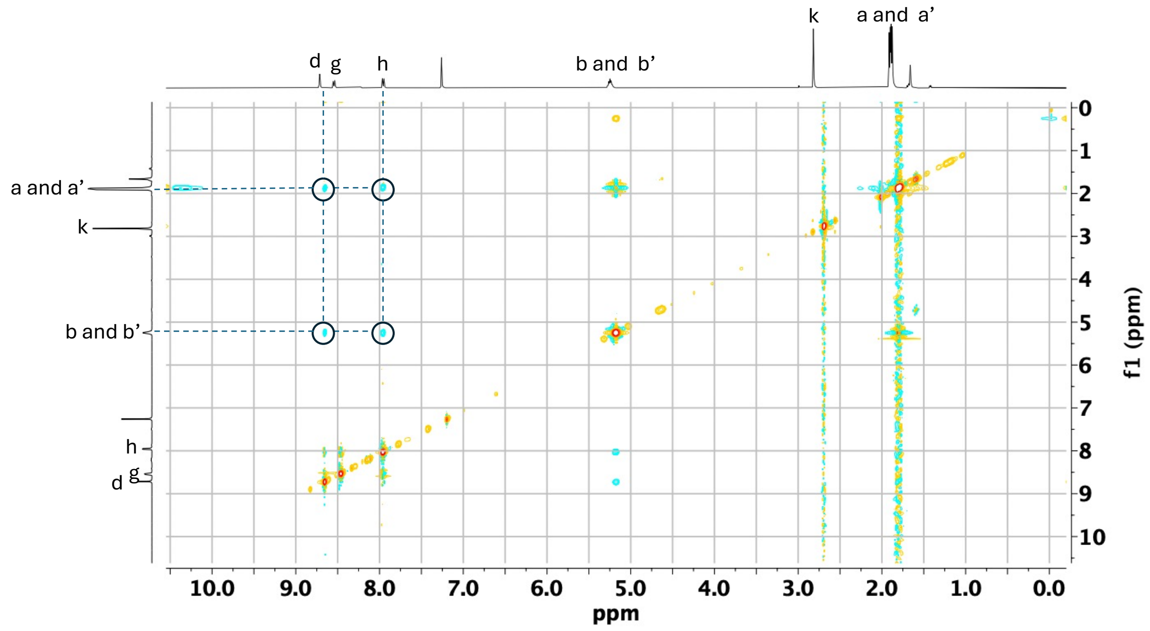

Figure S116. ^{1}H-^{1}H NOSEY spectrum of **8a**.

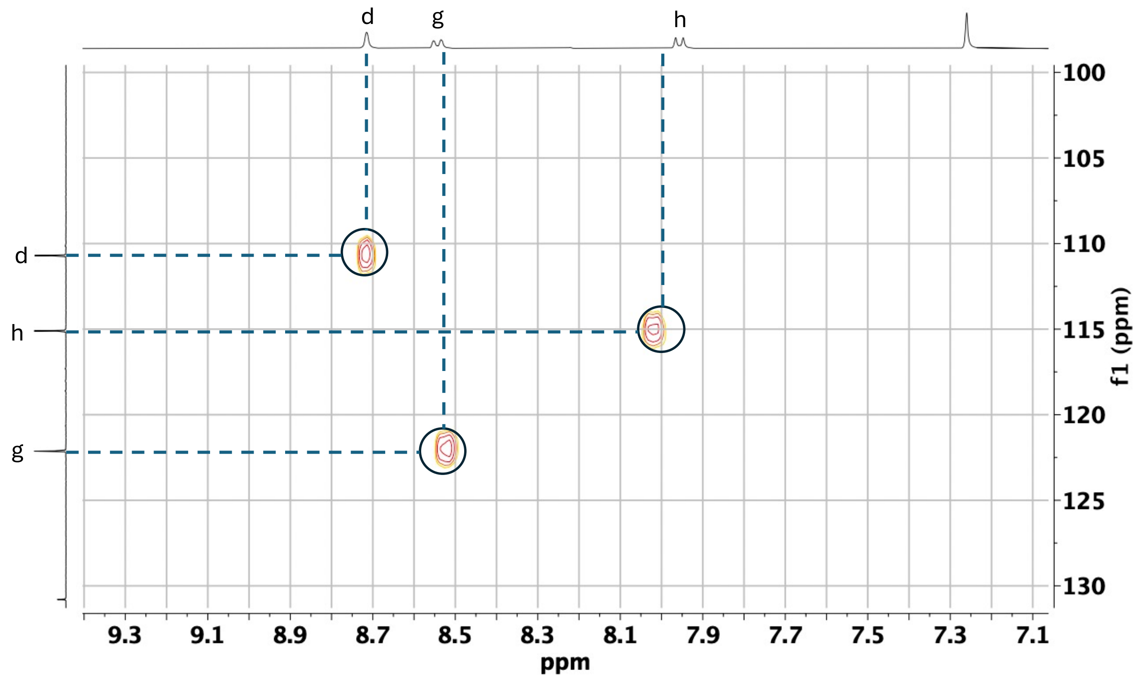

Figure S117. Zoomed in ^{1}H-^{13}C HSQC spectrum of **8a**.

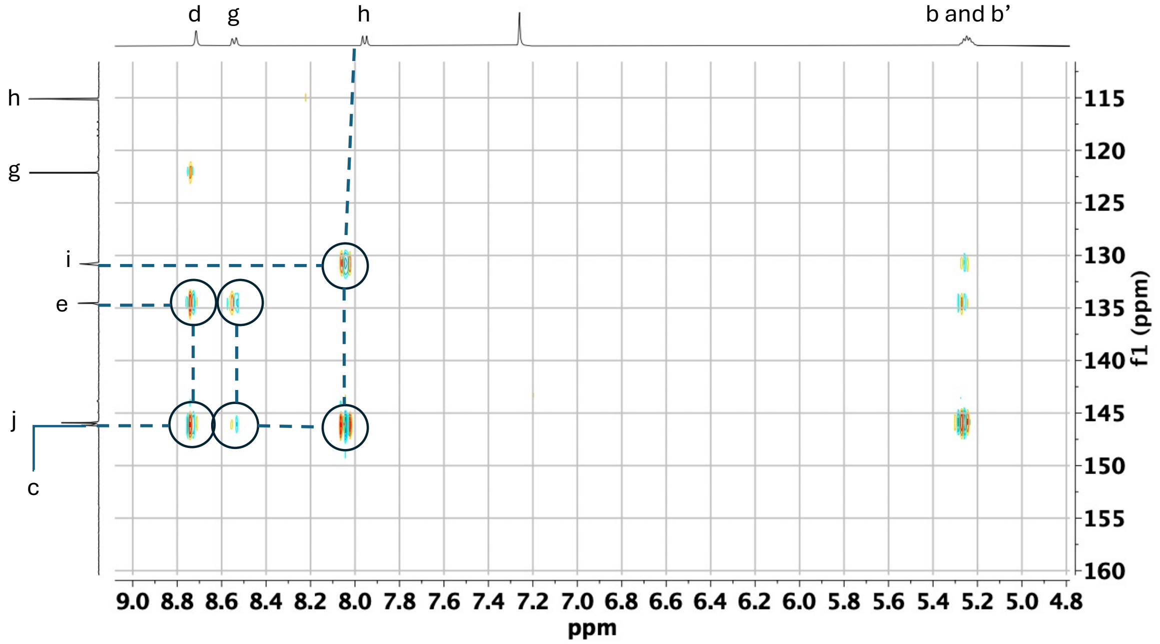

Figure S118. Zoomed in ^{1}H-^{13}C HMBC spectrum of **8a**.

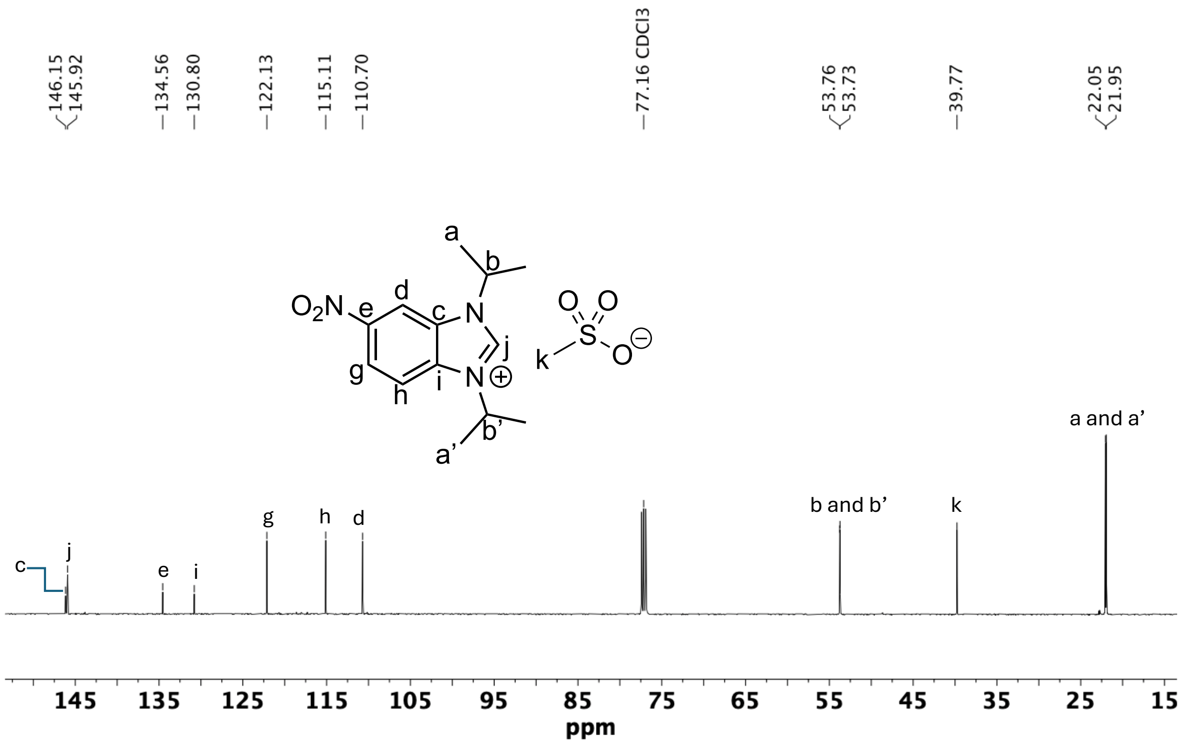

Figure S119. 126 MHz ^{13}C spectrum of **8a**.

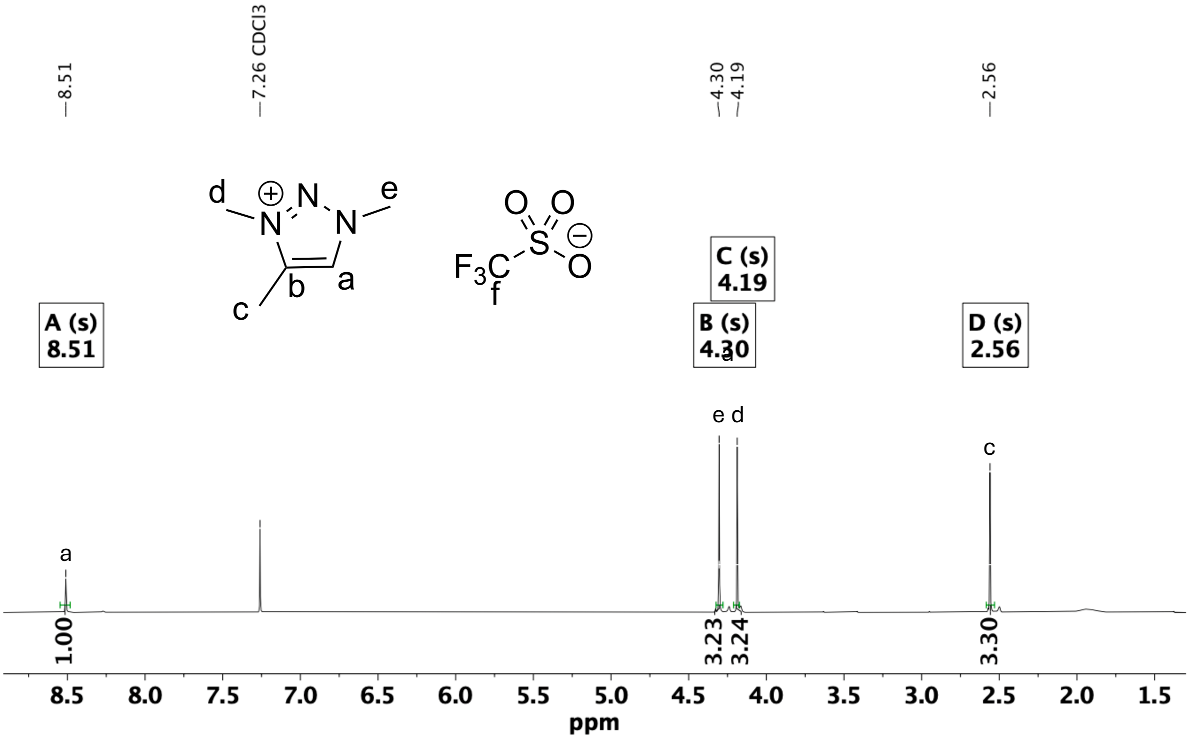

Figure S120. 500 MHz ^{1}H spectrum of **12i**.

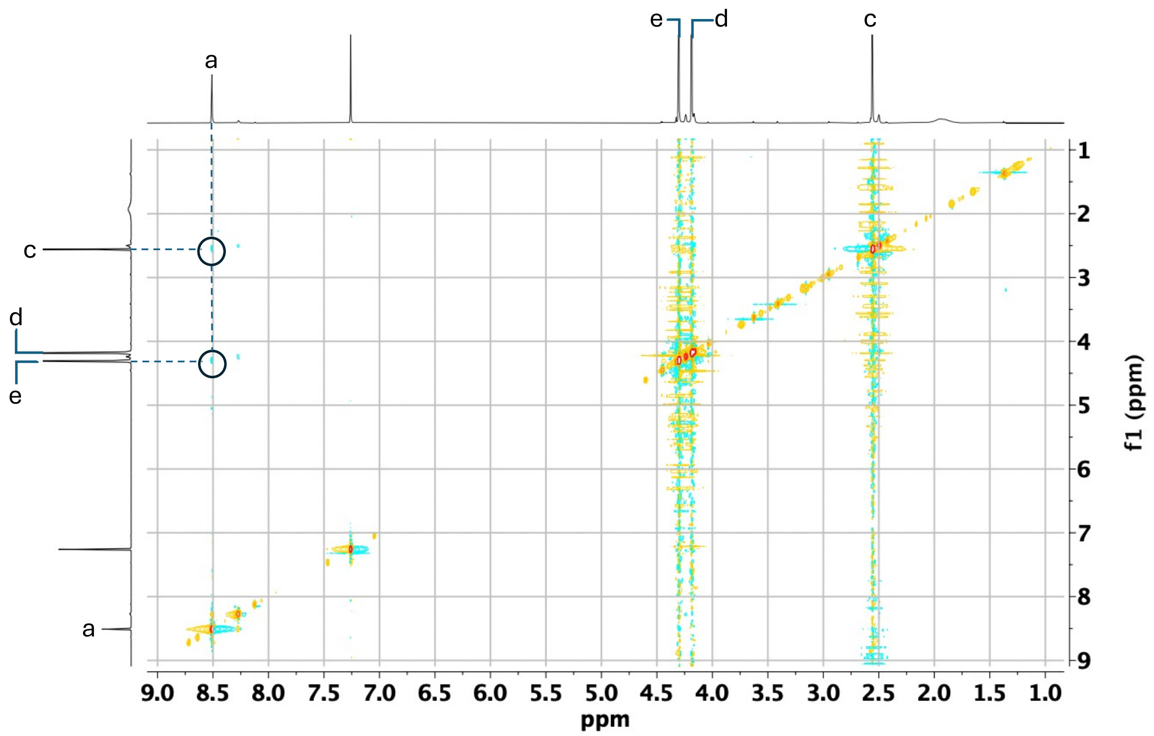

Figure S121. ^{1}H-^{1}H ROSEY spectrum of **12i**.

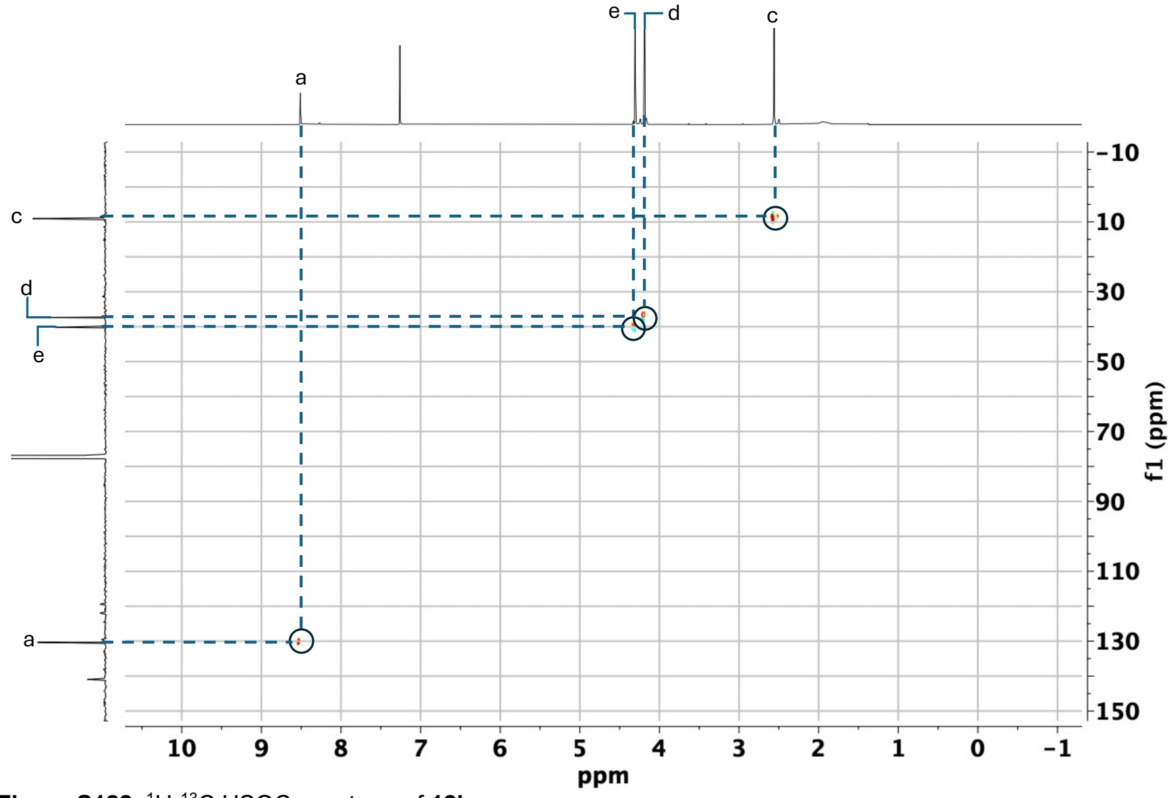

Figure S122. ^{1}H-^{13}C HSQC spectrum of **12i**.

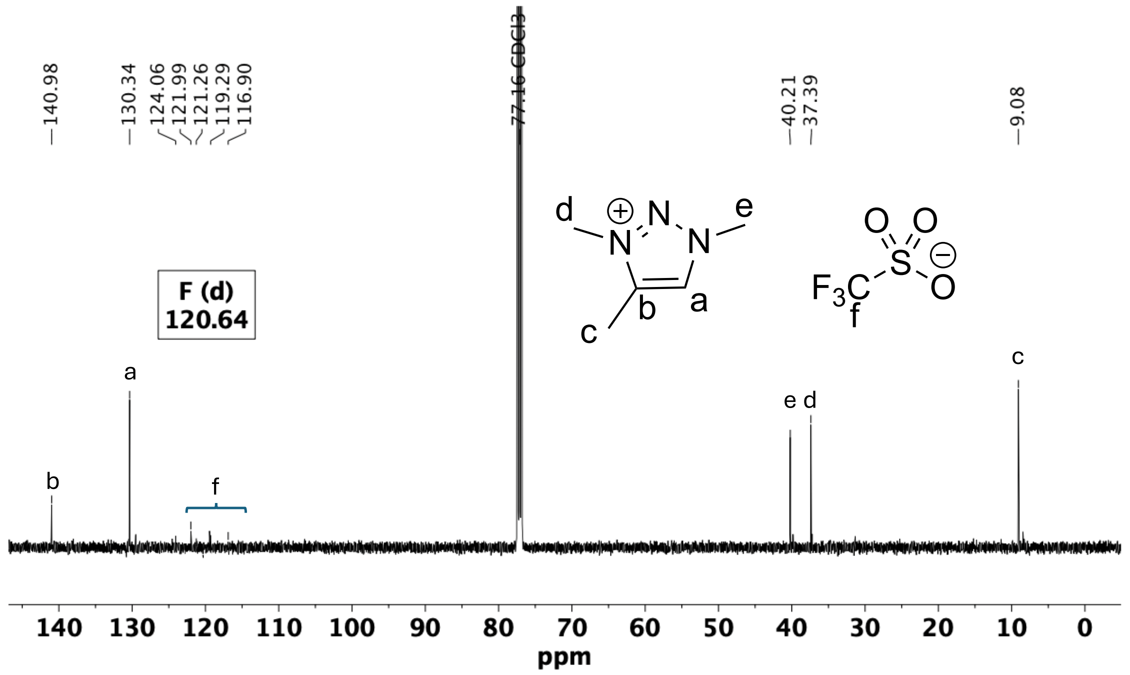

Figure S123. 126 MHz ^{13}C spectrum of **12i**.

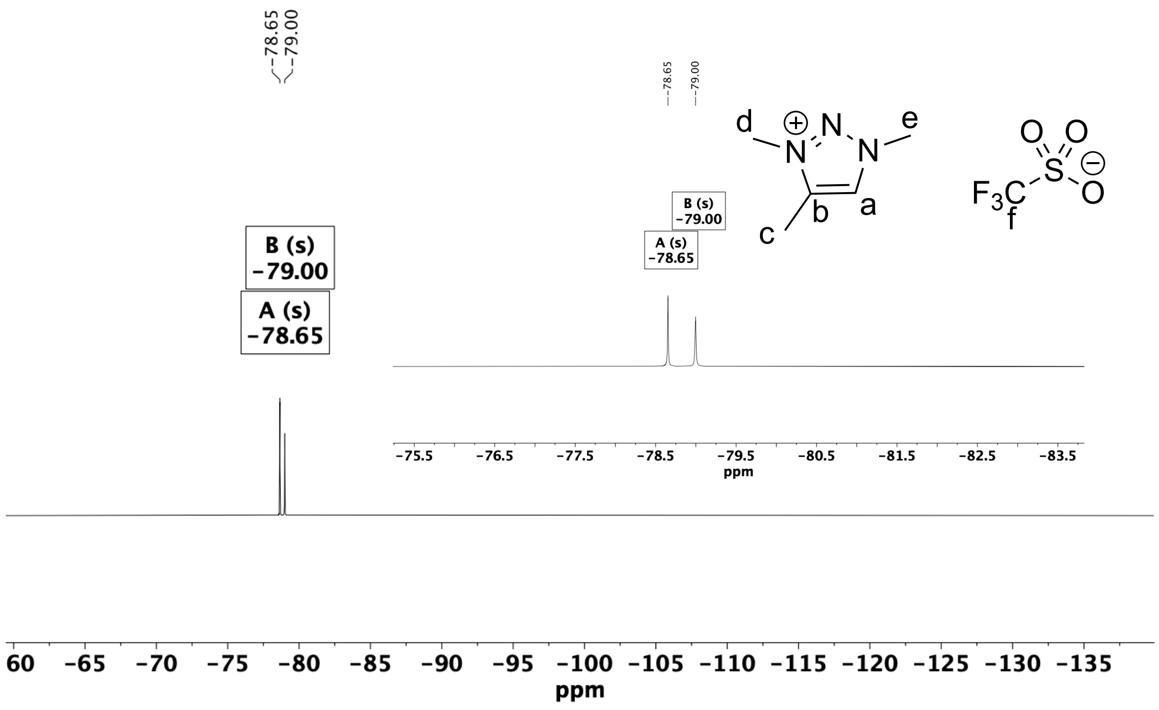

Figure S124. 565 MHz ^{19}F NMR spectrum of **12i**.

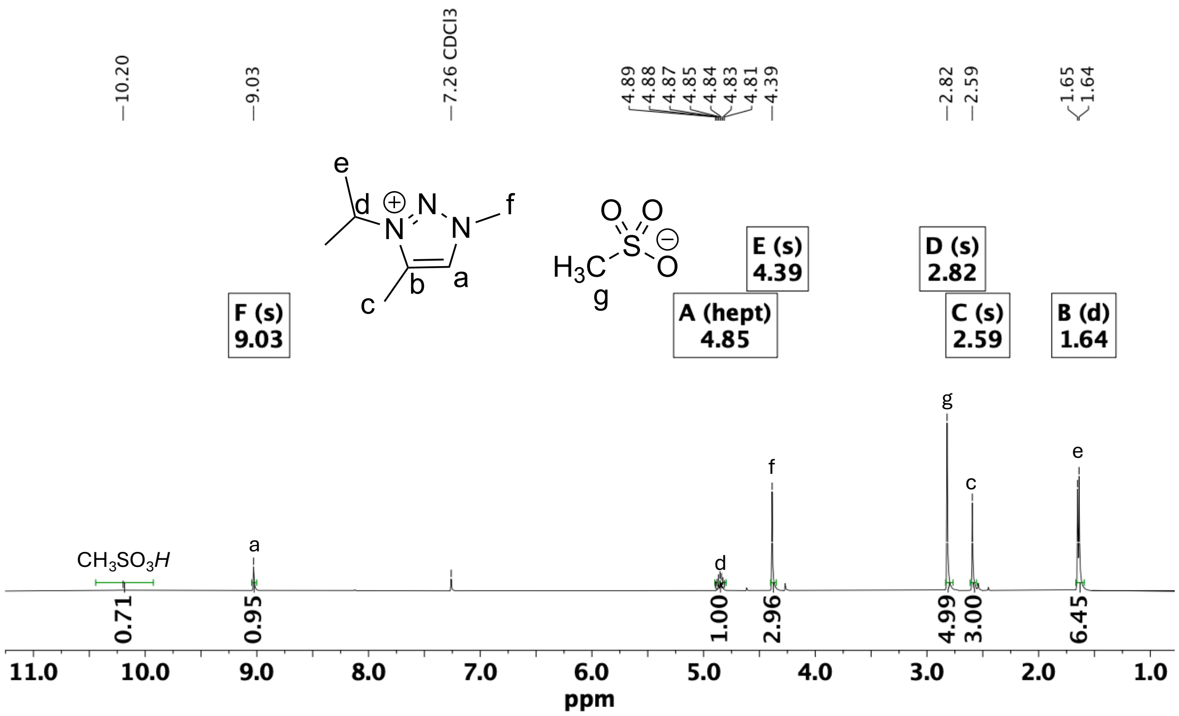

Figure S125. 500 MHz ^{1}H spectrum of **13a**.

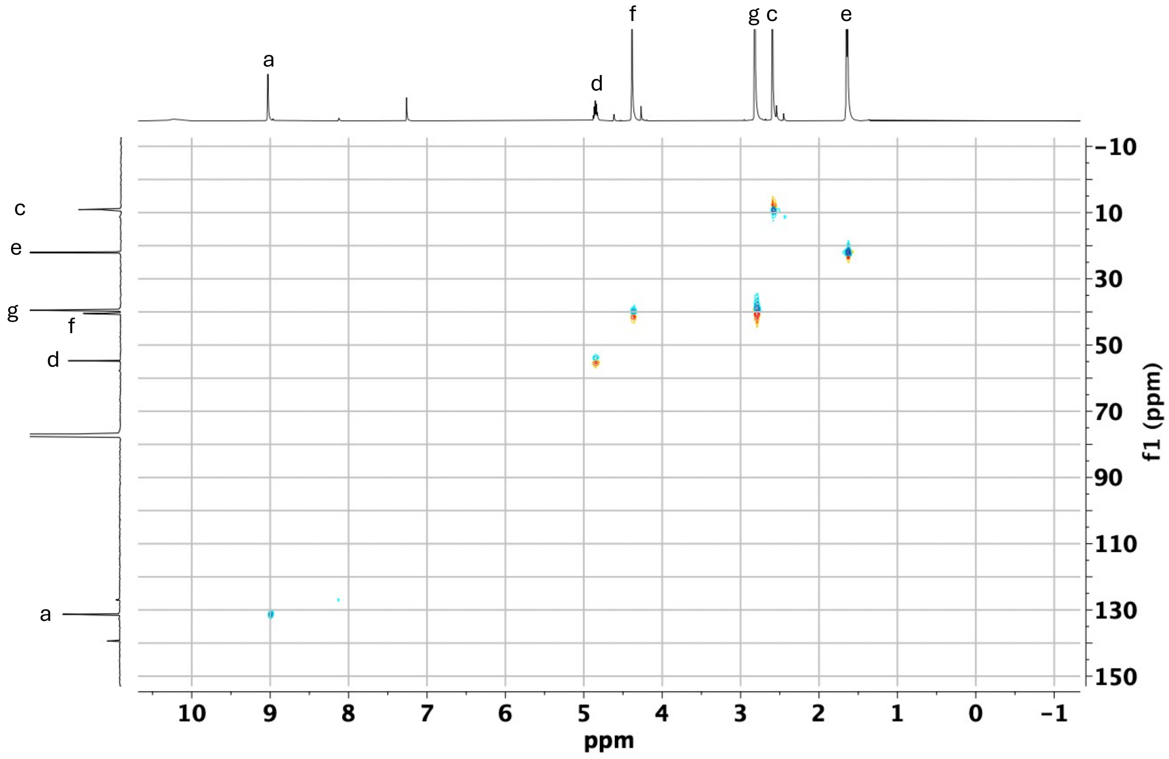

Figure S126. ^{1}H-^{13}C HSQC spectrum of **13a**.

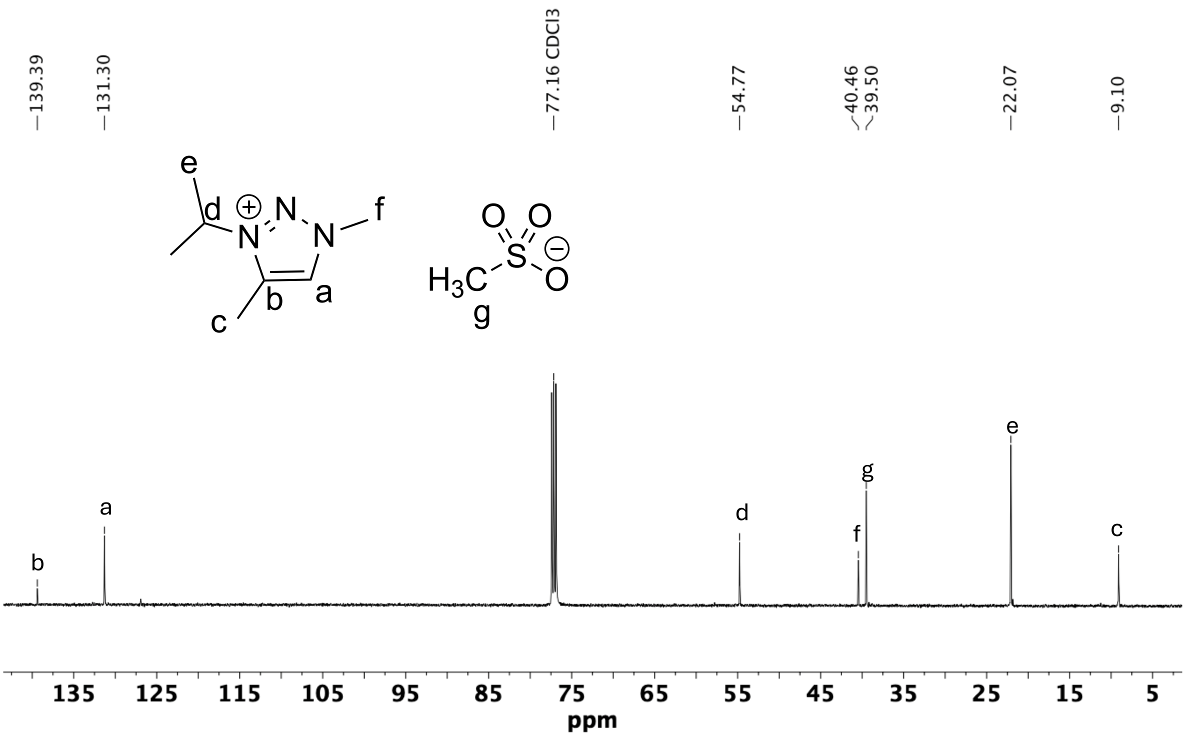

Figure S127. 126 MHz ^{13}C spectrum of **13a**.

S11 3D Printing and Stirrers

To allow for efficient mass transfer, the chips were immobilized onto 3D printed stirrers with stirrer bars inside. Once placed inside a vial and onto a traditional magnetically stirred hot–plate, the motion of the stirrer bar allowed for the whole structure to spin, therefore causing a mixing motion.

For all solvents, except THF and 2–MeTHF, polypropylene was used as the 3D printing material. For the ethereal solvents, PA6-CF (a carbon fibre and nylon-6 blend) was utilised instead due to significant swelling of the polypropylene.

The stirrers could be re–utilised for multiple functionalizations, but they were previously sonicated in DI–H_2O for 30 minutes, 3 times, then in methanol for 30 minutes, 3 times, and then dried in the oven for at least 2 hours. The stirrers were also cleaned with oxygen plasma with the SERS–substrates.

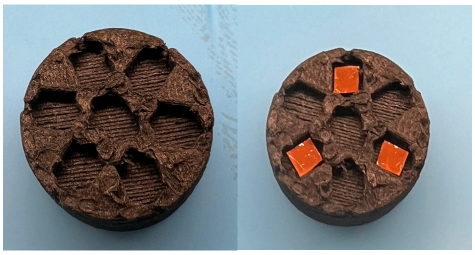

Figure S128. Photo of the 3D-printed spinners without (left) and with (right) SERS–active chips inside. Chips are 3 mm x 3 mm in size for reference.

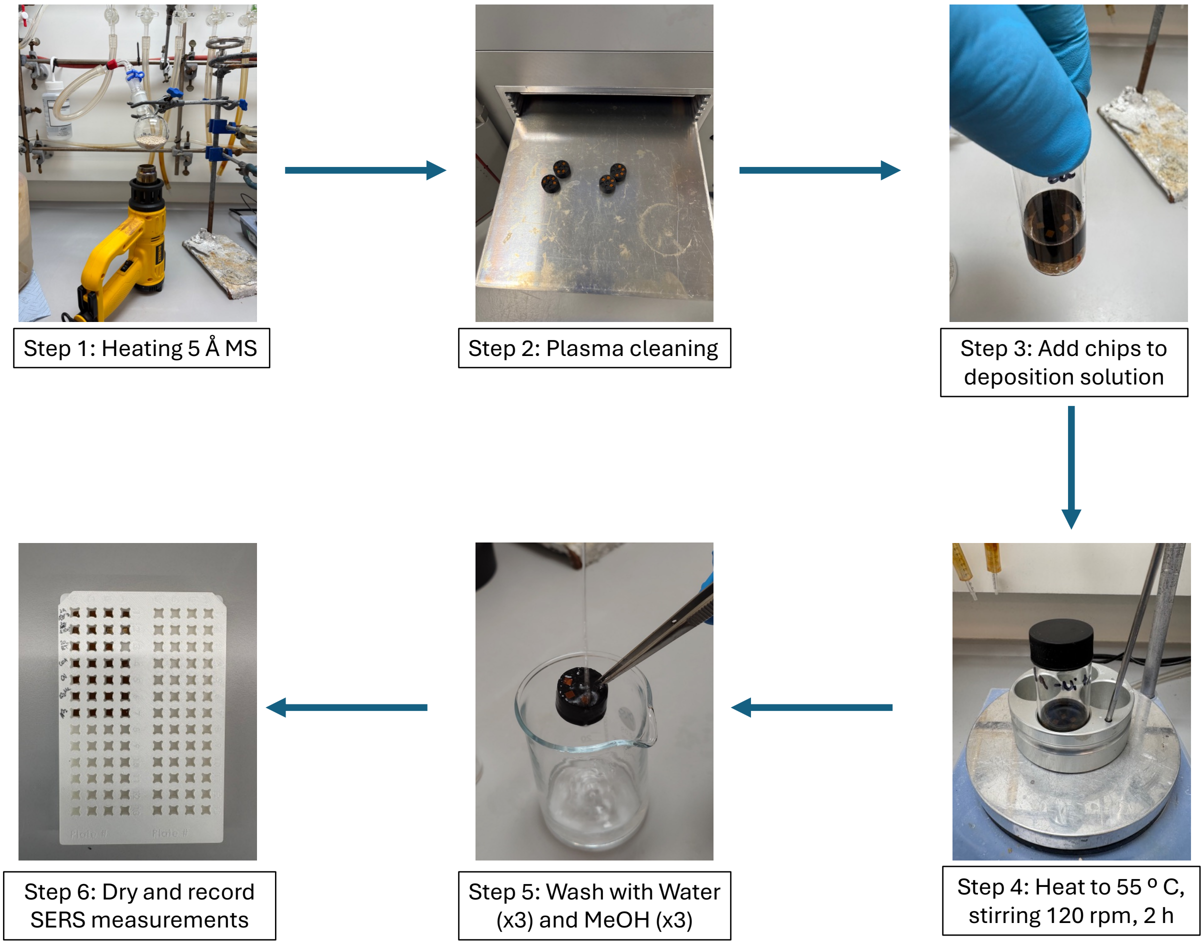

Figure S129. General procedure for the optimised deposition.

S12 DFT Coordinates and Free energies

1. Optimised structures in ethanol and energies

HCO_3^-
-264.397604 Hartree

O	1.19965	0.48075	0.00000
C	0.00000	0.13761	0.00000
O	-0.25532	-1.22495	0.00000
O	-1.01925	0.84985	0.00000
H	0.59930	-1.67086	0.00000

H_2O
-76.390001 Hartree

O	0.00000	0.00000	0.11811
H	0.00000	0.75923	-0.47246
H	0.00000	-0.75923	-0.47246

CO_2
-188.467372 Hartree

O	0.00000	0.00000	1.15704
C	0.00000	0.00000	0.00000
O	0.00000	0.00000	-1.15704

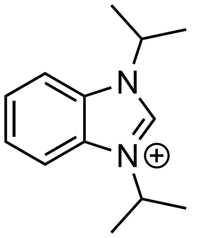

-615.485422 Hartree

C	-2.60490	-2.32423	-0.68045
C	-2.48688	-0.92683	-0.11453
C	-3.02321	-0.82434	1.30100
N	-1.09253	-0.45708	-0.15204
C	0.00003	-1.21077	-0.14911
N	1.09256	-0.45703	-0.15202
C	2.48693	-0.92672	-0.11451
C	3.02322	-0.82429	1.30103
C	2.60502	-2.32408	-0.68052
C	0.69834	0.87063	-0.14752
C	1.42847	2.05123	-0.15898
C	0.70094	3.22612	-0.16873
C	-0.70108	3.22609	-0.16875
C	-1.42856	2.05116	-0.15901
C	-0.69838	0.87060	-0.14753
H	-3.66252	-2.58138	-0.74372
H	-2.12326	-3.06386	-0.03691
H	-2.17716	-2.38948	-1.68234
H	-3.02992	-0.23619	-0.76204
H	-2.48753	-1.50913	1.96221
H	-4.08037	-1.09350	1.30643
H	-2.92661	0.18894	1.69342
H	0.00005	-2.28689	-0.15021
H	3.02995	-0.23602	-0.76197
H	2.48754	-1.50913	1.96220
H	2.92659	0.18896	1.69351
H	4.08039	-1.09342	1.30648
H	2.12339	-3.06377	-0.03704
H	3.66265	-2.58118	-0.74377
H	2.17731	-2.38929	-1.68243
H	2.51054	2.05109	-0.15862
H	1.22740	4.17239	-0.17621
H	-1.22759	4.17233	-0.17625
H	-2.51063	2.05098	-0.15868

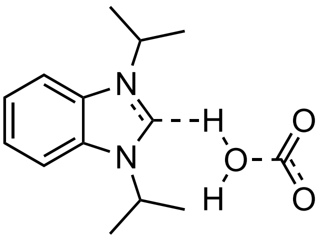

-879.829610 Hartree

C	0.31279	0.46683	0.38731
H	1.72132	0.85545	0.63970
C	0.87921	-1.91816	0.63046
C	-0.85434	2.65125	0.01214
C	1.17238	-2.72601	-0.62020
H	1.94930	-3.46108	-0.40297
H	0.28915	-3.26278	-0.97125
H	1.52790	-2.07952	-1.42490

C	0.40828	-2.76913	1.79549
H	-0.48047	-3.35161	1.55016
H	1.20017	-3.46904	2.06777
C	3.61770	0.08918	-0.77488
O	3.08645	0.61108	-1.68669
O	4.35208	-0.67922	-0.26578
O	2.83647	1.06964	0.75199
H	3.12265	0.60158	1.54317
H	0.18743	-2.14826	2.66581
H	-0.71315	2.96749	2.14081
H	-2.36989	2.71315	1.56240
C	-1.40967	3.17483	1.32483
H	-1.55605	4.25469	1.26237
H	-1.89235	-3.05259	-0.11522
N	-0.05791	-0.82741	0.34581
C	-2.23130	-2.03327	-0.23239
C	-1.38889	-0.94696	-0.02436
H	-4.21795	-2.58582	-0.77752
C	-3.53604	-1.76126	-0.60824
C	-1.84820	0.36102	-0.20314
N	-0.76928	1.18891	0.05082
C	-3.99375	-0.45014	-0.77538
C	-3.15684	0.63428	-0.57629
H	1.20514	3.14413	0.46471
H	-3.51043	1.64895	-0.70608
C	0.47801	3.27691	-0.33648
H	0.33260	4.34770	-0.48836
H	0.89027	2.85237	-1.25342
H	-1.56204	2.87634	-0.78899
H	1.79361	-1.40970	0.93375
H	-5.02290	-0.28021	-1.06780

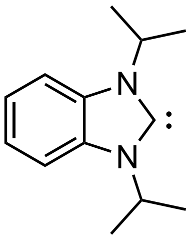

-614.996732 Hartree

C	3.13608	-1.79986	-0.83506
C	2.68699	-0.51633	-0.17113
C	3.24391	-0.37751	1.23551
N	1.22857	-0.39490	-0.15066
C	0.35767	-1.42651	-0.06349
N	-0.86553	-0.84452	-0.00058
C	-2.09265	-1.63539	0.08557
C	-2.96407	-1.44684	-1.14352
C	-2.83708	-1.37169	1.38250
C	0.58231	0.82813	-0.12790
C	-0.78304	0.53967	-0.03785
C	-1.72506	1.56178	-0.00597
C	-1.25601	2.86524	-0.06060
C	0.11009	3.14830	-0.14511
C	1.05265	2.13301	-0.17934
H	4.22377	-1.78630	-0.92802
H	2.70678	-1.90228	-1.83329
H	2.85447	-2.67411	-0.24732
H	3.04240	0.32212	-0.77532
H	4.33542	-0.37854	1.20961
H	2.91267	-1.21256	1.85781
H	2.91467	0.55288	1.70228
H	-1.73366	-2.66514	0.09832
H	-3.81068	-2.13455	-1.09819
H	-2.39971	-1.65911	-2.05378
H	-3.36095	-0.43272	-1.21252
H	-3.67556	-2.06558	1.46710
H	-3.23759	-0.35761	1.42663
H	-2.18120	-1.52162	2.24240
H	-2.78537	1.36241	0.05969
H	-1.96670	3.68275	-0.03567
H	0.43839	4.18010	-0.18279
H	2.11079	2.35369	-0.23993

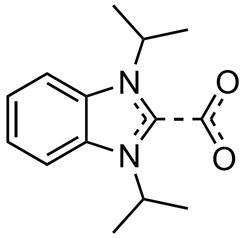

-803.454055 Hartree

C	3.40883	-0.00010	0.00015
O	3.56701	-0.22136	1.13411
O	3.56743	0.22114	-1.13374
H	0.51366	-2.60255	-2.17614
H	0.53067	2.64353	-2.12806
H	-0.84954	-3.37038	-1.34551
H	0.73815	-4.13824	-1.32698
H	-0.84135	3.39153	-1.29368
C	0.22295	-3.17646	-1.29408
H	0.74405	4.16346	-1.24815
C	0.23139	3.19983	-1.23764

C	0.60956	-2.44235	-0.02274
H	-2.45814	-2.49962	-0.01745
C	-2.44260	-1.41896	-0.01004
N	0.08273	1.07702	0.00801
C	-3.62546	-0.69837	-0.00471
C	-1.25252	-0.70047	-0.00507
C	0.89829	-0.00002	-0.00018
C	-1.25249	0.70054	0.00518
C	-3.62543	0.69853	0.00534
N	0.08267	-1.07701	-0.00819
H	-4.56871	1.23140	0.00909
C	-2.44254	1.41908	0.01041
H	1.69104	-2.30042	-0.02533
H	-2.45804	2.49973	0.01784
C	0.60970	2.44233	0.02239
H	1.69117	2.30032	0.02488
C	0.23133	-3.19994	1.23726
H	0.74396	-4.16359	1.24764
C	0.22327	3.17659	1.29370
H	-0.84140	-3.39160	1.29339
H	0.73860	4.13830	1.32649
H	-0.84919	3.37066	1.34518
H	0.53072	-2.64373	2.12770
H	0.51395	2.60271	2.17579
H	-4.56876	-1.23120	-0.00826

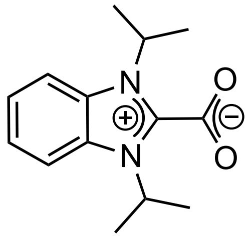

-803.493173 Hartree

C	-3.17977	-0.38923	1.28176
C	-2.46368	-0.75121	-0.00468
C	-3.21373	-0.33965	-1.25623
N	-1.09438	-0.20880	-0.01036
C	0.00028	-0.96740	-0.01240
C	0.00067	-2.49088	-0.01218
O	0.00075	-3.01274	-1.13777
O	0.00084	-3.01051	1.11447
N	1.09452	-0.20820	-0.01030
C	2.46411	-0.74996	-0.00468
C	3.18019	-0.38751	1.28163
C	3.21385	-0.33822	-1.25634
C	0.69963	1.12418	-0.00461

C	1.42207	2.31068	0.00146
C	0.69806	3.48829	0.00777
C	-0.70010	3.48788	0.00770
C	-1.42339	2.30984	0.00134
C	-0.70022	1.12378	-0.00465
H	-4.14365	-0.89983	1.30365
H	-3.36475	0.68298	1.35788
H	-2.60230	-0.70872	2.15064
H	-2.32871	-1.83295	-0.02879
H	-3.41443	0.73194	-1.27899
H	-4.17144	-0.86162	-1.27862
H	-2.65380	-0.61174	-2.15233
H	2.32964	-1.83176	-0.02862
H	3.36488	0.68477	1.35755
H	4.14420	-0.89786	1.30353
H	2.60290	-0.70701	2.15062
H	3.41404	0.73347	-1.27929
H	2.65400	-0.61070	-2.15236
H	4.17181	-0.85973	-1.27871
H	2.50217	2.32402	0.00185
H	1.22889	4.43219	0.01313
H	-1.23148	4.43147	0.01300
H	-2.50350	2.32246	0.00162

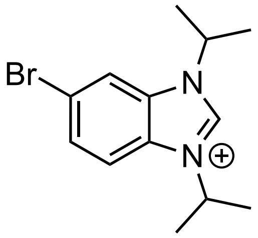

-3188.808003 Hartree

C	-2.44332	3.30743	-0.70234
C	-1.19292	2.70327	-0.10436
C	-0.94715	3.16449	1.31977
N	-1.24511	1.23139	-0.14615
C	-2.33591	0.47368	-0.17049
N	-2.01674	-0.81450	-0.16220
C	-2.94792	-1.95646	-0.14049
C	-3.05431	-2.49922	1.27222
C	-4.29207	-1.57312	-0.71669
C	-0.63717	-0.91259	-0.12292
C	0.21375	-2.00805	-0.11144
C	1.56956	-1.74933	-0.08733
C	2.04557	-0.43252	-0.07750
C	1.21468	0.66912	-0.09090
C	-0.14519	0.39389	-0.11276

H	-2.30522	4.38692	-0.76711
H	-3.31973	3.12386	-0.07710
H	-2.63317	2.92648	-1.70726
H	-0.33714	2.96792	-0.72813
H	-1.79662	2.90665	1.95595
H	-0.82266	4.24817	1.33017
H	-0.04625	2.71349	1.73812
H	-3.34169	0.85471	-0.20076
H	-2.48532	-2.70483	-0.78656
H	-3.50887	-1.75760	1.93263
H	-2.07681	-2.77243	1.67187
H	-3.68261	-3.39098	1.26802
H	-4.82257	-0.86940	-0.07171
H	-4.90147	-2.47388	-0.79288
H	-4.19432	-1.14181	-1.71443
H	-0.15950	-3.02314	-0.12053
H	2.26979	-2.57386	-0.07609
Br	3.91769	-0.15725	-0.04400
H	1.59254	1.68185	-0.08441

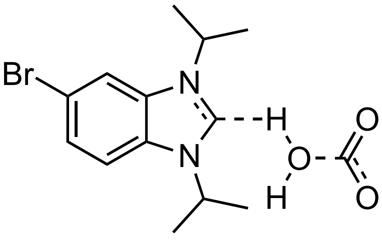

-3453.152443 Hartree

C	1.51150	0.46870	0.44356
H	2.97674	0.83235	0.49867
C	2.05363	-1.92838	0.62963
C	0.34891	2.67704	0.21003
C	2.16233	-2.75330	-0.63892
H	2.94761	-3.50128	-0.51684
H	1.23140	-3.27679	-0.86418
H	2.42056	-2.12168	-1.49101
C	1.71995	-2.75574	1.85696
H	0.78788	-3.31015	1.74212
H	2.51908	-3.47958	2.02576
C	4.63634	0.02223	-1.11656
O	3.99248	0.52155	-1.96926
O	5.43426	-0.74658	-0.70876
O	4.08366	1.01667	0.45067
H	4.46930	0.56836	1.21094
H	1.64054	-2.12210	2.74231
H	0.75373	2.98926	2.30492
H	-0.96671	2.77516	1.93226

C	-0.03139	3.21304	1.57875
H	-0.15848	4.29602	1.53063
H	-0.81835	-3.00437	0.25192
N	1.11167	-0.81747	0.45797
C	-1.14220	-1.97678	0.17180
C	-0.25653	-0.91056	0.26300
H	-3.19471	-2.49600	-0.11127
C	-2.47949	-1.68776	-0.03610
C	-0.70823	0.40581	0.13576
N	0.40797	1.21233	0.24395
C	-2.90834	-0.36416	-0.15033
C	-2.04611	0.71211	-0.06934
Br	-4.75447	-0.03263	-0.43085
H	2.45700	3.13001	0.40997
H	-2.39279	1.73218	-0.15970
C	1.64205	3.27326	-0.29967
H	1.49964	4.34593	-0.44036
H	1.93322	2.83638	-1.25634
H	-0.44514	2.91678	-0.50065
H	3.01064	-1.43845	0.80417

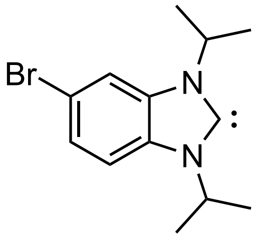

-3188.322332 Hartree

C	2.31630	3.48540	-0.87413
C	1.16634	2.80326	-0.16572
C	0.98025	3.32074	1.25021
N	1.32436	1.34722	-0.15222
C	2.50700	0.68935	-0.10230
N	2.17374	-0.62222	-0.02506
C	3.18948	-1.67367	0.03986
C	3.12387	-2.59038	-1.16857
C	3.12481	-2.42702	1.35632
C	0.25276	0.47801	-0.09177
C	0.80037	-0.80651	-0.01553
C	-0.02273	-1.92364	0.04770
C	-1.39334	-1.72184	0.03794
C	-1.91514	-0.43070	-0.03483
C	-1.11771	0.69680	-0.10001
H	2.09441	4.55096	-0.95829
H	2.45887	3.08309	-1.87862

H	3.24963	3.37339	-0.32153
H	0.25362	2.99511	-0.73528
H	0.77755	4.39336	1.23410
H	1.88561	3.14901	1.83757
H	0.14570	2.82332	1.74831
H	4.12944	-1.12210	0.00343
H	3.97557	-3.27294	-1.15125
H	3.16626	-2.01430	-2.09500
H	2.21462	-3.19354	-1.17740
H	3.96507	-3.12106	1.41871
H	2.20539	-3.00700	1.45129
H	3.18717	-1.73748	2.20047
H	0.37094	-2.92842	0.10445
H	-2.05978	-2.57251	0.08859
Br	-3.80118	-0.21611	-0.04145
H	-1.53877	1.69134	-0.15278

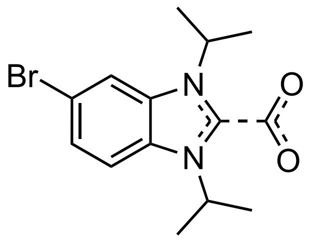

-3376.777471 Hartree

C	4.25957	-0.95325	-0.40447
O	4.83836	-0.87302	0.60554
O	3.98571	-1.13529	-1.52519
H	1.08394	-2.71800	-1.85960
H	2.50322	2.27081	-2.00764
H	-0.50680	-3.02150	-1.14112
H	0.78213	-4.21190	-0.96059
H	1.36652	3.43192	-1.30156
C	0.56805	-3.14220	-0.99607
H	3.10224	3.72205	-1.19242
C	2.33439	2.94666	-1.16707
C	1.04603	-2.49070	0.28871
H	-1.90264	-1.69510	0.08055
C	-1.58415	-0.66389	0.05887
Br	-4.34481	-0.06664	-0.13782
N	1.53147	1.03547	0.16713
C	-2.49966	0.36652	-0.04137
C	-0.24509	-0.29826	0.12406
C	2.00783	-0.22474	0.24940
C	0.14806	1.04577	0.08782
C	-2.12836	1.70967	-0.07651
N	0.92429	-1.03256	0.22530

H	-2.88337	2.48040	-0.15599
C	-0.79164	2.06317	-0.01191
H	2.12074	-2.65433	0.37881
H	-0.50909	3.10552	-0.04170
C	2.42159	2.19845	0.15073
H	3.41700	1.75979	0.23126
C	0.37565	-3.05401	1.52855
H	0.60079	-4.11918	1.60631
C	2.19332	3.09096	1.35679
H	-0.70936	-2.94489	1.49714
H	2.95400	3.87349	1.37576
H	1.21629	3.57605	1.32864
H	0.74744	-2.55945	2.42797
H	2.26897	2.51770	2.28276

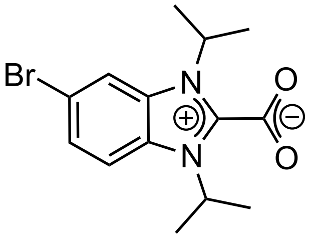

-3376.815836 Hartree

C	0.41168	3.16617	-1.26869
C	1.01433	2.59802	0.00125
C	0.40960	3.16111	1.27251
N	0.94794	1.12596	-0.00159
C	2.02076	0.33461	-0.00291
C	3.46184	0.83089	-0.00248
O	3.95479	0.99053	1.12432
O	3.94965	1.01001	-1.12857
N	1.65653	-0.94657	-0.00321
C	2.61257	-2.06895	-0.00527
C	2.47019	-2.89920	-1.26581
C	2.49729	-2.87939	1.27076
C	0.26939	-1.00209	-0.00109
C	-0.62420	-2.06385	0.00107
C	-1.97292	-1.76627	0.00189
C	-2.40470	-0.43874	0.00074
C	-1.53510	0.63325	-0.00037
C	-0.18195	0.32219	-0.00089
H	0.58389	4.24326	-1.28675
H	-0.66441	2.99722	-1.32395
H	0.88180	2.72839	-2.15058
H	2.08152	2.82140	0.00263
H	-0.66678	2.99328	1.32496
H	0.58302	4.23790	1.29570

H	0.87743	2.71893	2.15344
H	3.59232	-1.59095	-0.01990
H	1.52799	-3.44778	-1.29174
H	3.28221	-3.62716	-1.29721
H	2.53795	-2.27177	-2.15581
H	1.55282	-3.42201	1.32684
H	2.58923	-2.23876	2.14908
H	3.30608	-3.61125	1.29401
H	-0.29595	-3.09208	0.00282
H	-2.69531	-2.57136	0.00345
Br	-4.26690	-0.09558	0.00107
H	-1.89181	1.65168	-0.00107

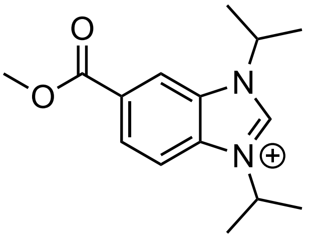

-843.178155 Hartree

C	-4.42397	-1.15563	-0.78937
C	-3.14140	-1.72141	-0.22290
C	-3.33322	-2.35896	1.14018
N	-2.09435	-0.68689	-0.14021
C	-2.27503	0.62030	0.02569
N	-1.11231	1.24747	0.13608
C	-0.90081	2.70053	0.25975
C	-2.11616	3.37484	0.85471
C	-0.51873	3.27630	-1.09090
C	-0.10613	0.30142	0.03621
C	-0.73552	-0.93434	-0.13463
C	-0.00855	-2.11065	-0.26339
C	1.36348	-1.99530	-0.21606
C	1.99864	-0.75338	-0.04387
C	3.48084	-0.73897	-0.00309
O	3.97935	0.48210	0.14636
C	5.40536	0.57929	0.19790
O	4.16850	-1.73292	-0.09620
C	1.27105	0.42062	0.08433
H	-5.12162	-1.97820	-0.94820
H	-4.25519	-0.66024	-1.74705
H	-4.89518	-0.45150	-0.10060
H	-2.74279	-2.46090	-0.91923
H	-3.72959	-1.63006	1.85046
H	-2.39418	-2.75436	1.53018
H	-4.04356	-3.18256	1.05607

H	-0.06253	2.80021	0.95190
H	-2.96779	3.34709	0.17140
H	-1.87307	4.42192	1.03645
H	-2.40342	2.92096	1.80470
H	-1.34851	3.17975	-1.79450
H	0.35545	2.77506	-1.50881
H	-0.28307	4.33516	-0.97584
H	-0.49129	-3.06963	-0.39390
H	1.97966	-2.87939	-0.31193
H	5.84477	0.20846	-0.72816
H	5.79372	0.01308	1.04459
H	5.62200	1.63739	0.32081
H	1.75587	1.37696	0.21627
H	-3.23497	1.10525	0.06569

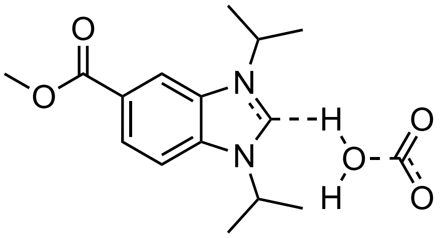

-1107.523950 Hartree

C	1.44336	0.40087	0.57346
H	2.86833	0.88949	0.59052
C	2.11561	-1.96852	0.49995
C	0.22078	2.56557	0.50446
C	2.25069	-2.66437	-0.84148
H	3.08679	-3.36454	-0.80036
H	1.35260	-3.22872	-1.09919
H	2.44765	-1.94226	-1.63593
C	1.84640	-2.92431	1.64701
H	0.94188	-3.51311	1.48979
H	2.68422	-3.61788	1.73654
C	4.50631	0.30583	-1.13520
O	3.77740	0.76309	-1.94172
O	5.39076	-0.39444	-0.78704
O	3.94003	1.20889	0.48270
H	4.42120	0.80551	1.21318
H	1.74911	-2.38238	2.58958
H	2.07774	2.95166	1.54623
H	0.68362	2.69482	2.61255
C	1.00889	3.13305	1.66715
H	0.85033	4.21167	1.71545
H	-0.69468	-3.15113	0.02593
N	1.11086	-0.90065	0.44526
C	-1.07817	-2.14170	0.05349

C	-0.24907	-1.04199	0.25108
H	-3.10344	-2.72492	-0.27618
C	-2.42502	-1.89711	-0.11726
C	-0.77208	0.25716	0.27254
N	0.30302	1.10130	0.48177
C	-2.94764	-0.59520	-0.09454
C	-2.12103	0.50553	0.10057
H	1.71744	2.91454	-1.01036
H	-2.52109	1.50917	0.11356
C	0.66187	3.13189	-0.83237
H	0.52651	4.21491	-0.83950
H	0.07690	2.70698	-1.65037
H	-0.83468	2.79372	0.65740
H	3.04542	-1.44070	0.71016
H	-6.76188	0.59500	0.35446
C	-4.40426	-0.43164	-0.28767
O	-5.17744	-1.35244	-0.45490
O	-4.79145	0.84079	-0.25929
C	-6.18921	1.07836	-0.43736
H	-6.31205	2.15731	-0.38482
H	-6.51809	0.70865	-1.40882

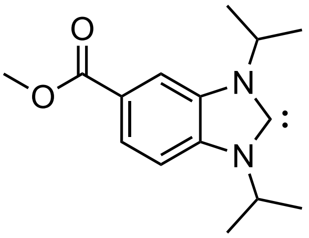

-842.693576 Hartree

C	-4.38465	-1.21358	-0.91063
C	-3.14208	-1.69476	-0.19302
C	-3.44928	-2.21475	1.20038
N	-2.11736	-0.64987	-0.12581
C	-2.36801	0.67716	0.02786
N	-1.14575	1.24587	0.13031
C	-0.88632	2.68468	0.22179
C	-1.99016	3.40826	0.96163
C	-0.65242	3.26169	-1.16363
C	-0.13029	0.30964	0.03879
C	-0.76780	-0.92543	-0.11397
C	-0.04134	-2.10526	-0.21907
C	1.33557	-2.00448	-0.17836
C	1.97811	-0.76557	-0.03463
C	3.45402	-0.75360	-0.00272
O	3.96163	0.47163	0.11880
C	5.38652	0.56237	0.16031

O	4.14879	-1.74719	-0.07831
C	1.24661	0.41539	0.07540
H	-5.06737	-2.05587	-1.03777
H	-4.14268	-0.81486	-1.89748
H	-4.89950	-0.43878	-0.34183
H	-2.69883	-2.50147	-0.78132
H	-3.89974	-1.42641	1.80840
H	-2.54343	-2.56284	1.70088
H	-4.15049	-3.04958	1.14340
H	0.03405	2.77950	0.80313
H	-2.92483	3.39275	0.40008
H	-1.69408	4.44921	1.10454
H	-2.16859	2.96339	1.94219
H	-1.56191	3.18461	-1.76446
H	0.15088	2.73471	-1.68237
H	-0.37634	4.31530	-1.08916
H	-0.52590	-3.06661	-0.32794
H	1.94307	-2.89650	-0.25821
H	5.82148	0.17011	-0.75928
H	5.77941	0.01387	1.01680
H	5.61001	1.62189	0.25882
H	1.74217	1.36953	0.18541

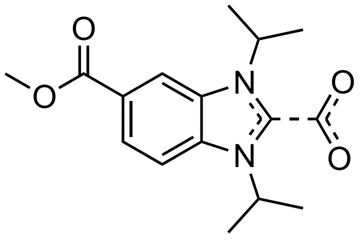

-1031.146978 Hartree

C	-4.17667	1.18782	0.36053
O	-4.09603	0.93991	1.49927
O	-4.56745	1.55034	-0.67784
H	-0.51949	2.60005	-2.35287
H	-2.43446	-2.33928	-2.30704
H	0.98979	2.80706	-1.44861
H	-0.19246	4.11443	-1.49891
H	-1.51677	-3.51501	-1.35126
C	-0.07817	3.02999	-1.45170
H	-3.27455	-3.62606	-1.43097
C	-2.43610	-2.92860	-1.38810
C	-0.77486	2.50697	-0.20902
H	2.07645	1.42049	-0.05625
C	1.66196	0.42443	-0.04175
N	-1.59580	-0.96023	-0.16155
C	2.49614	-0.68623	0.03918

C	0.29859	0.19606	-0.10129
C	-1.95010	0.34585	-0.21827
C	-0.22500	-1.10502	-0.08497
C	1.96995	-1.98530	0.06055
N	-0.79703	1.04261	-0.18212
H	2.65295	-2.82195	0.12737
C	0.61089	-2.21421	-0.00095
H	-1.82969	2.77801	-0.26723
H	0.22329	-3.22231	0.01957
C	-2.59501	-2.03144	-0.17412
H	-3.54187	-1.49817	-0.26715
C	-0.20454	3.07590	1.07746
H	-0.32105	4.16111	1.07419
C	-2.60216	-2.79485	1.13746
H	0.85815	2.85463	1.18867
H	-3.43003	-3.50615	1.13733
H	-1.67874	-3.35596	1.28917
H	-0.73418	2.67752	1.94497
H	-2.73622	-2.11339	1.97973
H	6.26387	0.50238	-0.71011
C	3.96368	-0.53268	0.10674
O	4.74494	-1.45947	0.18107
O	4.35532	0.73929	0.07804
C	5.76430	0.96632	0.14067
H	5.88915	2.04582	0.10631
H	6.17402	0.56947	1.06980

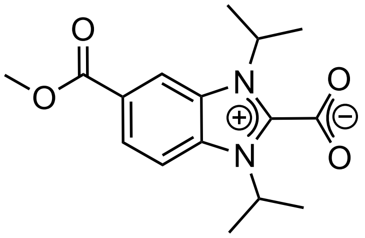

-1031.180200 Hartree

C	-3.56550	-2.00286	-1.09625
C	-2.36647	-2.22517	-0.19833
C	-2.73863	-2.69322	1.19543
N	-1.47981	-1.04552	-0.11875
C	-1.81270	0.24740	0.00227
N	-0.70753	0.99179	0.09421
C	-0.58237	2.46151	0.18278
C	-1.59600	3.08516	1.11906
C	-0.59526	3.06891	-1.20687
C	0.39264	0.15135	0.02738
C	-0.10183	-1.14684	-0.09881
C	0.74776	-2.24297	-0.17544
C	2.10128	-1.98437	-0.13425
C	2.60092	-0.67779	-0.02124
C	4.07137	-0.50115	0.00602
O	4.43628	0.77326	0.09096
C	5.84326	1.02564	0.12140
O	4.86464	-1.41707	-0.04450
C	1.74931	0.41580	0.06195
H	-4.02639	-2.97397	-1.28272
H	-3.27171	-1.57651	-2.05615
H	-4.31997	-1.36628	-0.63546
H	-1.74137	-2.98232	-0.67098
H	-3.36536	-1.95487	1.69710
H	-1.84799	-2.87033	1.80088
H	-3.29466	-3.62924	1.12198
H	0.40339	2.60698	0.62436
H	-2.59794	3.11106	0.69214
H	-1.29080	4.11666	1.30091
H	-1.62604	2.56587	2.07767
H	-1.56970	2.93429	-1.67817
H	0.16925	2.61627	-1.84065
H	-0.38947	4.13791	-1.13229
H	0.37678	-3.25500	-0.25899
H	2.80503	-2.80417	-0.19058
H	6.31461	0.66065	-0.79124
H	6.29698	0.54633	0.98899
H	5.94522	2.10564	0.19187
H	2.13696	1.42038	0.14662
C	-3.24241	0.78671	0.03566
O	-3.83957	0.59944	1.10523
O	-3.61478	1.33912	-1.00895

2. Optimised structures in MeCN and energies

HCO_3^-
-264.385609 Hartree

O	1.21006	0.44886	0.00000
C	0.00000	0.14823	0.00000
O	-0.27749	-1.22460	0.00000
O	-1.00556	0.87140	0.00000
H	0.58391	-1.65470	0.00000

H_2O
-76.386208 Hartree

O	0.00000	0.00000	0.11799
H	0.00000	0.75947	-0.47196
H	0.00000	-0.75947	-0.47196

CO_2
-188.471952 Hartree

O	0.00000	0.00000	1.15734
C	0.00000	0.00000	0.00000
O	0.00000	0.00000	-1.15734

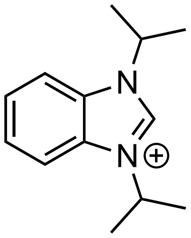

-615.488834 Hartree

C	-2.60770	-2.32358	-0.68087
C	-2.48829	-0.92579	-0.11572
C	-3.03251	-0.82059	1.29699
N	-1.09260	-0.45873	-0.14649
C	-0.00000	-1.21238	-0.14386
N	1.09260	-0.45874	-0.14649
C	2.48828	-0.92580	-0.11572
C	3.03250	-0.82059	1.29699
C	2.60769	-2.32359	-0.68086
C	0.69842	0.86882	-0.14481
C	1.42861	2.04955	-0.15873
C	0.70110	3.22463	-0.17141
C	-0.70109	3.22463	-0.17141
C	-1.42861	2.04956	-0.15872
C	-0.69842	0.86882	-0.14481
H	-3.66566	-2.57932	-0.74244
H	-2.12563	-3.06237	-0.03695
H	-2.18086	-2.38960	-1.68286
H	-3.02573	-0.23349	-0.76597
H	-2.50440	-1.50796	1.96129
H	-4.09082	-1.08483	1.29510
H	-2.93349	0.19240	1.68912
H	-0.00001	-2.28836	-0.14759
H	3.02572	-0.23350	-0.76597
H	2.50440	-1.50796	1.96129
H	2.93349	0.19240	1.68911
H	4.09082	-1.08483	1.29509
H	2.12562	-3.06238	-0.03694
H	3.66565	-2.57933	-0.74243
H	2.18085	-2.38961	-1.68286
H	2.51056	2.04870	-0.15798
H	1.22757	4.17075	-0.18120
H	-1.22755	4.17075	-0.18120
H	-2.51055	2.04871	-0.15797

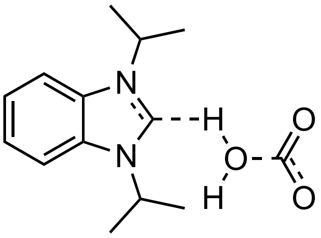

-879.831931 Hartree

C	0.29491	0.40338	0.48697
H	1.70271	0.71920	0.66508
C	0.74428	-2.01631	0.45859
C	-0.73119	2.67660	0.33019
C	0.89723	-2.74532	-0.86338
H	1.64961	-3.52857	-0.75671
H	-0.03542	-3.21576	-1.17847
H	1.22388	-2.06084	-1.64809
C	0.30745	-2.92309	1.59449
H	-0.64096	-3.41820	1.38228
H	1.06246	-3.69659	1.74448
C	3.69115	-0.04380	-0.74749
O	3.18834	0.44623	-1.69388
O	4.43234	-0.78881	-0.20877
O	2.84385	0.91691	0.72391
H	3.16489	0.50233	1.53078
H	0.20527	-2.35886	2.52323
H	1.25725	2.93424	1.14746
H	-0.01889	2.78504	2.36878
C	0.22459	3.19142	1.38535
H	0.14482	4.27857	1.43368
H	-2.12400	-2.92870	-0.22653
N	-0.14468	-0.85776	0.32675
C	-2.41097	-1.88693	-0.23223
C	-1.49855	-0.87032	0.02726
H	-4.45080	-2.28096	-0.71423
C	-3.71576	-1.51308	-0.50612
C	-1.89361	0.47122	0.01206
N	-0.76297	1.21101	0.30916
C	-4.10596	-0.16971	-0.52316
C	-3.20128	0.84592	-0.26593
H	0.60391	2.92801	-1.34644

H	-3.50287	1.88504	-0.28588
C	-0.41004	3.21083	-1.05438
H	-0.47823	4.30012	-1.05705
H	-1.10777	2.82166	-1.79815
H	-1.74328	2.98095	0.60263
H	1.70709	-1.57999	0.72364
H	-5.13603	0.08097	-0.74521

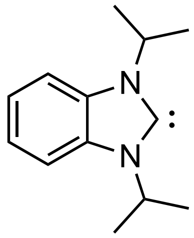

-615.000866 Hartree

C	3.14259	-1.79377	-0.84113
C	2.68681	-0.51628	-0.16978
C	3.24859	-0.37966	1.23552
N	1.22748	-0.39921	-0.14510
C	0.35501	-1.42954	-0.06011
N	-0.86760	-0.84636	0.00032
C	-2.09771	-1.63355	0.08521
C	-2.96778	-1.44126	-1.14462
C	-2.84235	-1.36707	1.38177
C	0.58230	0.82454	-0.12544
C	-0.78345	0.53776	-0.03755
C	-1.72452	1.56117	-0.00782
C	-1.25386	2.86411	-0.06274
C	0.11294	3.14555	-0.14521
C	1.05440	2.12907	-0.17688
H	4.22985	-1.77138	-0.93576
H	2.71256	-1.89396	-1.83908
H	2.86984	-2.67291	-0.25671
H	3.03600	0.32676	-0.77082
H	4.33979	-0.37235	1.20359
H	2.92747	-1.21991	1.85581
H	2.91445	0.54641	1.70697
H	-1.74341	-2.66458	0.09807
H	-3.81580	-2.12706	-1.10009
H	-2.40304	-1.65336	-2.05452
H	-3.36200	-0.42612	-1.21150
H	-3.68284	-2.05842	1.46538
H	-3.23960	-0.35178	1.42409
H	-2.18725	-1.51832	2.24188
H	-2.78496	1.36254	0.05647
H	-1.96358	3.68235	-0.03956

H	0.44249	4.17682	-0.18306
H	2.11294	2.34788	-0.23510

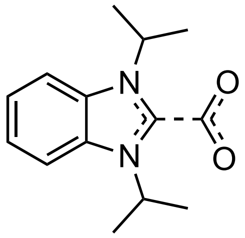

-803.458164 Hartree

C	3.33218	0.06307	-0.13381
O	3.36492	0.13117	-1.30065
O	3.66787	0.00140	0.98375
H	0.36995	2.62380	2.25112
H	0.50232	-2.61241	2.23744
H	-0.96954	3.36120	1.35645
H	0.60153	4.16090	1.40576
H	-0.81954	-3.38872	1.34988
C	0.10772	3.18890	1.35471
H	0.77814	-4.13600	1.38067
C	0.25142	-3.18094	1.33995
C	0.56595	2.45828	0.10508
H	-2.49721	2.44800	-0.05891
C	-2.45847	1.36803	-0.05946
H	-4.57700	1.13393	-0.17430
N	0.11736	-1.07312	0.06822
C	-3.62374	0.62179	-0.12444
C	-1.25463	0.67544	0.00255
C	0.90659	0.02162	0.11413
C	-1.22408	-0.72530	-0.00121
C	-3.59320	-0.77493	-0.12811
N	0.07040	1.08117	0.07435
H	-4.52319	-1.32799	-0.18037
C	-2.39637	-1.46975	-0.06700
H	1.64908	2.34113	0.15602
H	-2.38739	-2.55041	-0.07177
C	0.67259	-2.42734	0.09097
H	1.74975	-2.26311	0.13750
C	0.22579	3.20158	-1.17426
H	0.71477	4.17729	-1.16589
C	0.36015	-3.18083	-1.18958
H	-0.84771	3.36622	-1.27761
H	0.89902	-4.12993	-1.19012
H	-0.70418	-3.40068	-1.28512
H	0.57748	2.64911	-2.04764
H	0.67563	-2.60692	-2.06297

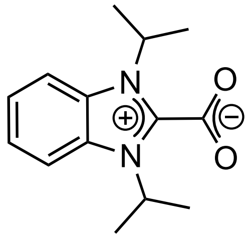

-1031.176806 Hartree

C	-3.51861	-2.02833	-1.13628
C	-2.36201	-2.22743	-0.17850
C	-2.79890	-2.64034	1.21432
N	-1.47394	-1.04969	-0.10659
C	-1.81198	0.24419	0.00198
N	-0.70485	0.98938	0.08204
C	-0.58713	2.45895	0.16386
C	-1.54867	3.06642	1.16431
C	-0.69305	3.07041	-1.22039
C	0.39707	0.15125	0.02203
C	-0.09603	-1.14934	-0.09200
C	0.75590	-2.24414	-0.16360
C	2.10969	-1.98340	-0.12820
C	2.60810	-0.67583	-0.02491
C	4.08074	-0.50011	-0.00131
O	4.44135	0.77754	0.10142
C	5.84710	1.03037	0.13068
O	4.87277	-1.41198	-0.06731
C	1.75404	0.41680	0.05223
H	-3.99717	-2.99641	-1.29111
H	-3.17304	-1.66049	-2.10322
H	-4.27015	-1.34619	-0.74122
H	-1.72293	-3.00570	-0.59416
H	-3.42998	-1.86977	1.65811
H	-1.93535	-2.80876	1.86022
H	-3.36639	-3.57019	1.15107
H	0.42245	2.61372	0.54328
H	-2.57831	3.05164	0.80943
H	-1.26363	4.10867	1.31462
H	-1.49343	2.55564	2.12648
H	-1.69233	2.91499	-1.62826
H	0.04044	2.63055	-1.89843
H	-0.49952	4.14228	-1.15452
H	0.38570	-3.25706	-0.23902
H	2.81645	-2.80084	-0.18056
H	6.31876	0.67643	-0.78616
H	6.30605	0.54086	0.98984
H	5.95063	2.10941	0.21360
H	2.14112	1.42236	0.12753
C	-3.24790	0.78430	0.03280
O	-3.86169	0.52900	1.07625
O	-3.58221	1.39649	-0.98883

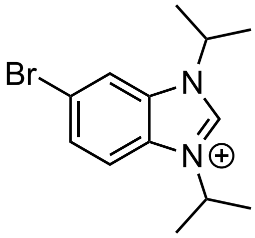

-3188.811224 Hartree

C	-2.43974	3.31094	-0.70221
C	-1.18913	2.70526	-0.10581
C	-0.93696	3.17300	1.31547
N	-1.24508	1.23309	-0.14044
C	-2.33647	0.47628	-0.16532
N	-2.01857	-0.81216	-0.15610
C	-2.94901	-1.95507	-0.14288
C	-3.05816	-2.50673	1.26656
C	-4.29273	-1.57045	-0.71974
C	-0.63917	-0.91153	-0.11868
C	0.21078	-2.00799	-0.10800
C	1.56704	-1.75066	-0.08595
C	2.04408	-0.43416	-0.07661
C	1.21442	0.66848	-0.08833
C	-0.14595	0.39465	-0.10887
H	-2.30044	4.39030	-0.76474
H	-3.31531	3.12613	-0.07641
H	-2.63030	2.93163	-1.70739
H	-0.33434	2.96394	-0.73322
H	-1.78563	2.92232	1.95529
H	-0.80798	4.25610	1.31877
H	-0.03658	2.72026	1.73268
H	-3.34175	0.85815	-0.19836
H	-2.48325	-2.69890	-0.79172
H	-3.51689	-1.77046	1.92978
H	-2.08100	-2.77976	1.66673
H	-3.68415	-3.39991	1.25412
H	-4.82176	-0.86577	-0.07481
H	-4.90249	-2.47090	-0.79513
H	-4.19414	-1.13958	-1.71735
H	-0.16412	-3.02242	-0.11587
H	2.26671	-2.57556	-0.07573
Br	3.91691	-0.16071	-0.04592
H	1.59302	1.68086	-0.08132

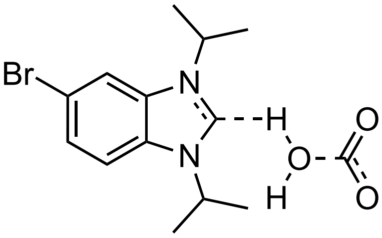

-3453.155808 Hartree

C	1.46978	0.43484	0.33074
H	2.87984	0.80094	0.44251
C	1.99353	-1.96707	0.48711
C	0.34340	2.66248	0.15758
C	2.04756	-2.80574	-0.77600
H	2.82867	-3.56065	-0.67210
H	1.10423	-3.32161	-0.96198
H	2.28276	-2.18586	-1.64283
C	1.68993	-2.77828	1.73304
H	0.74433	-3.31570	1.65295
H	2.48156	-3.51487	1.87950
C	4.91531	0.02683	-0.87639
O	4.38220	0.39152	-1.86352
O	5.71316	-0.61660	-0.28712
O	4.01015	1.02841	0.49886
H	4.33239	0.67803	1.33532
H	1.65206	-2.13551	2.61434
H	1.07823	2.94906	2.16567
H	-0.68308	2.76524	2.06272
C	0.19374	3.19210	1.57231
H	0.08057	4.27734	1.55129
H	-0.89928	-3.00682	0.17846
N	1.05920	-0.84566	0.34005
C	-1.21503	-1.97557	0.11530
C	-0.31524	-0.91964	0.18598
H	-3.28165	-2.46915	-0.10282
C	-2.55500	-1.66991	-0.04476
C	-0.75502	0.40384	0.08683
N	0.37335	1.19583	0.17034
C	-2.97211	-0.34013	-0.13111
C	-2.09627	0.72626	-0.06828
Br	-4.82303	0.01384	-0.34506
H	2.47297	3.02558	-0.00110
H	-2.43628	1.75046	-0.13328
C	1.55418	3.23147	-0.55046
H	1.43784	4.31337	-0.63055
H	1.65123	2.82060	-1.55698
H	-0.54654	2.92498	-0.41677

H	2.96184	-1.48631	0.62278

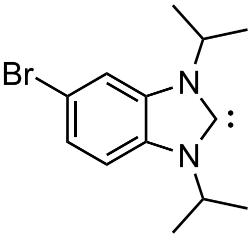

-3188.326212 Hartree

C	2.30083	3.49406	-0.87984
C	1.15937	2.80459	-0.16446
C	0.97224	3.32833	1.24938
N	1.32540	1.34890	-0.14542
C	2.50853	0.69197	-0.09749
N	2.17690	-0.61995	-0.02211
C	3.19175	-1.67307	0.03979
C	3.12478	-2.58532	-1.17228
C	3.12474	-2.43155	1.35349
C	0.25479	0.47817	-0.08793
C	0.80371	-0.80580	-0.01353
C	-0.01816	-1.92430	0.04729
C	-1.38905	-1.72411	0.03670
C	-1.91219	-0.43320	-0.03420
C	-1.11617	0.69548	-0.09648
H	2.06793	4.55691	-0.96669
H	2.44314	3.08982	-1.88340
H	3.23688	3.39438	-0.32974
H	0.24348	2.98682	-0.73168
H	0.75868	4.39858	1.22623
H	1.88067	3.17031	1.83550
H	0.14383	2.82550	1.75191
H	4.13284	-1.12386	0.00604
H	3.97461	-3.27000	-1.15618
H	3.16901	-2.00567	-2.09626
H	2.21378	-3.18553	-1.18302
H	3.96515	-3.12533	1.41410
H	2.20536	-3.01213	1.44329
H	3.18481	-1.74511	2.20017
H	0.37735	-2.92835	0.10279
H	-2.05476	-2.57534	0.08525
Br	-3.79862	-0.22099	-0.04231
H	-1.53808	1.68971	-0.14715

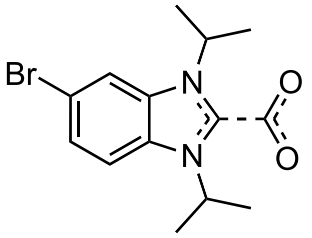

-3376.782255 Hartree

C	-4.30550	0.92091	-0.15553
O	-4.64851	0.95520	0.96186
O	-4.32903	0.99632	-1.32292
H	-0.95766	2.68129	-2.04448
H	-2.42526	-2.34439	-2.09345
H	0.59175	3.00209	-1.24762
H	-0.70444	4.19487	-1.16294
H	-1.31073	-3.47557	-1.30725
C	-0.48912	3.12501	-1.16406
H	-3.04885	-3.76624	-1.24466
C	-2.28339	-2.98856	-1.22369
C	-1.03670	2.50217	0.10759
H	1.91035	1.70009	0.02678
C	1.59302	0.66843	0.02054
Br	4.35897	0.06560	-0.05407
N	-1.52688	-1.02703	0.06520
C	2.51066	-0.36449	-0.01576
C	0.25197	0.30469	0.04598
C	-2.00367	0.23529	0.09409
C	-0.14119	-1.03950	0.03477
C	2.13935	-1.70814	-0.02657
N	-0.91961	1.04222	0.08288
H	2.89587	-2.48081	-0.05530
C	0.80056	-2.05941	-0.00134
H	-2.11309	2.67390	0.13772
H	0.51701	-3.10172	-0.01103
C	-2.41426	-2.19262	0.06237
H	-3.41286	-1.75591	0.09512
C	-0.42607	3.08712	1.36856
H	-0.63962	4.15658	1.40926
C	-2.22352	-3.04046	1.30707
H	0.65734	2.96184	1.39588
H	-2.98602	-3.82092	1.33101
H	-1.24714	-3.52696	1.32537
H	-0.85180	2.61797	2.25748
H	-2.32587	-2.43320	2.20834

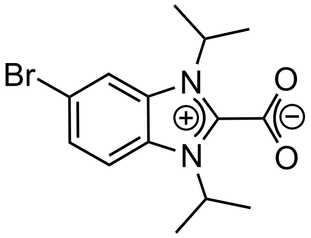

-3376.812366 Hartree

C	0.41290	3.17001	-1.25936
C	1.01556	2.59693	0.00901
C	0.40978	3.15912	1.28076
N	0.94851	1.12593	0.00263
C	2.02423	0.33609	0.00121
C	3.47179	0.83200	-0.00181
O	3.97598	0.93071	1.12439
O	3.92374	1.06116	-1.13128
N	1.65609	-0.94531	-0.00036
C	2.61190	-2.06625	-0.00644
C	2.48032	-2.88464	-1.27637
C	2.48786	-2.89146	1.25983
C	0.26914	-1.00175	-0.00056
C	-0.62425	-2.06346	-0.00272
C	-1.97396	-1.76675	-0.00212
C	-2.40534	-0.43962	0.00020
C	-1.53548	0.63296	0.00245
C	-0.18214	0.32296	0.00191
H	0.58796	4.24659	-1.27583
H	-0.66369	3.00392	-1.31210
H	0.87946	2.73168	-2.14280
H	2.08297	2.81799	0.01072
H	-0.66696	2.99308	1.32969
H	0.58522	4.23545	1.30687
H	0.87420	2.71307	2.16151
H	3.58996	-1.58494	-0.00749
H	1.53754	-3.43152	-1.31394
H	3.29182	-3.61304	-1.30850
H	2.55450	-2.24825	-2.15943
H	1.54430	-3.43678	1.30083
H	2.56904	-2.26003	2.14581
H	3.29826	-3.62143	1.28251
H	-0.29476	-3.09137	-0.00443
H	-2.69623	-2.57183	-0.00346
Br	-4.26813	-0.09571	-0.00030
H	-1.89272	1.65120	0.00366

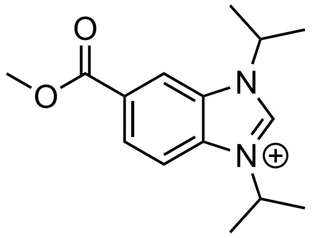

-843.181695

C	-4.42664	-1.15329	-0.78622
C	-3.14245	-1.72044	-0.22427
C	-3.33366	-2.36874	1.13426
N	-2.09649	-0.68516	-0.13522
C	-2.27643	0.62244	0.02703
N	-1.11322	1.24971	0.13263
C	-0.89865	2.70203	0.26078
C	-2.11371	3.37949	0.85322
C	-0.50975	3.28107	-1.08699
C	-0.10747	0.30314	0.03428
C	-0.73757	-0.93278	-0.13297
C	-0.01085	-2.10964	-0.26077
C	1.36141	-1.99419	-0.21546
C	1.99765	-0.75243	-0.04607
C	3.48204	-0.74319	-0.00547
O	3.98050	0.48077	0.14630
C	5.40580	0.57291	0.20194
O	4.16528	-1.73631	-0.09867
C	1.27039	0.42180	0.08038
H	-5.12496	-1.97568	-0.94230
H	-4.26064	-0.65772	-1.74406
H	-4.89394	-0.44904	-0.09506
H	-2.74242	-2.45457	-0.92535
H	-3.73289	-1.64646	1.84942
H	-2.39404	-2.76489	1.52160
H	-4.04190	-3.19317	1.04224
H	-0.06199	2.79693	0.95536
H	-2.96437	3.35047	0.16896
H	-1.86864	4.42652	1.03187
H	-2.40232	2.92838	1.80389
H	-1.33756	3.19067	-1.79344
H	0.36390	2.77782	-1.50314
H	-0.27048	4.33840	-0.96640
H	-0.49489	-3.06833	-0.38799
H	1.97983	-2.87676	-0.30994
H	5.84896	0.20194	-0.72224
H	5.79219	0.00312	1.04712
H	5.62796	1.62962	0.32783
H	1.75537	1.37847	0.20883

H	-3.23606	1.10765	0.06768

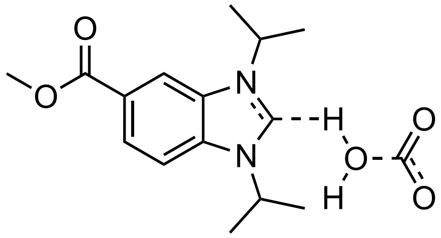

-1107.526493 Hartree

C	1.43053	0.39678	0.55875
H	2.81914	0.85158	0.59870
C	2.11393	-1.96928	0.44031
C	0.16337	2.55503	0.54983
C	2.22757	-2.66165	-0.90501
H	3.06493	-3.36082	-0.87806
H	1.32616	-3.22620	-1.14892
H	2.41109	-1.93714	-1.70010
C	1.86352	-2.92859	1.58900
H	0.95442	-3.51334	1.44357
H	2.70094	-3.62444	1.66098
C	4.67327	0.35274	-1.04251
O	3.99603	0.78455	-1.90725
O	5.57110	-0.30328	-0.63975
O	3.92890	1.15954	0.53319
H	4.38600	0.75939	1.27978
H	1.78344	-2.38894	2.53430
H	2.21784	3.03451	1.04006
H	1.18696	2.72146	2.44755
C	1.21602	3.16395	1.45019
H	1.01993	4.23315	1.54351
H	-0.69623	-3.15567	-0.02801
N	1.10561	-0.90359	0.40688
C	-1.08334	-2.14830	0.01882
C	-0.25605	-1.04848	0.22541
H	-3.11288	-2.72968	-0.29976
C	-2.43286	-1.90461	-0.13387
C	-0.78427	0.24641	0.27710
N	0.28727	1.09433	0.49268
C	-2.96043	-0.60540	-0.08433
C	-2.13590	0.49432	0.12181
H	1.17268	2.96602	-1.31210
H	-2.53778	1.49682	0.15298
C	0.19538	3.13361	-0.85369
H	0.01198	4.20866	-0.81451
H	-0.56893	2.67861	-1.48617
H	-0.81790	2.74130	0.99082

H	3.04710	-1.44176	0.63569
H	-6.77969	0.54651	0.41430
C	-4.42232	-0.44707	-0.26334
O	-5.18838	-1.36542	-0.44928
O	-4.81220	0.82586	-0.19693
C	-6.21117	1.06019	-0.36125
H	-6.34233	2.13587	-0.27218
H	-6.54402	0.72089	-1.34250

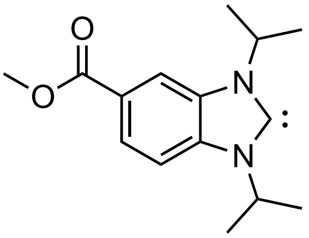

-842.697582 Hartree

C	-4.38988	-1.21835	-0.90877
C	-3.14404	-1.69234	-0.19178
C	-3.44893	-2.21969	1.19980
N	-2.12223	-0.64412	-0.12231
C	-2.36983	0.68349	0.02716
N	-1.14636	1.25069	0.12536
C	-0.87827	2.68822	0.21978
C	-1.97291	3.42030	0.96530
C	-0.64208	3.26872	-1.16420
C	-0.13267	0.31206	0.03607
C	-0.77243	-0.92185	-0.11275
C	-0.04807	-2.10342	-0.21576
C	1.32931	-2.00443	-0.17684
C	1.97460	-0.76668	-0.03669
C	3.45362	-0.76169	-0.00544
O	3.96174	0.46573	0.12144
C	5.38588	0.55020	0.16487
O	4.14218	-1.75479	-0.08458
C	1.24529	0.41535	0.07084
H	-5.06551	-2.06603	-1.03690
H	-4.15101	-0.81665	-1.89498
H	-4.91173	-0.44964	-0.33831
H	-2.69637	-2.49527	-0.78165
H	-3.90802	-1.43837	1.81012
H	-2.54074	-2.56171	1.69987
H	-4.14252	-3.06033	1.13753
H	0.04445	2.77412	0.79845
H	-2.90954	3.41620	0.40712
H	-1.66562	4.45785	1.10837
H	-2.15130	2.97590	1.94589

H	-1.55373	3.20564	-1.76308
H	0.15401	2.73498	-1.68678
H	-0.35392	4.31860	-1.08463
H	-0.53493	-3.06396	-0.32101
H	1.93830	-2.89551	-0.25454
H	5.82257	0.16002	-0.75478
H	5.77775	-0.00510	1.01745
H	5.61611	1.60783	0.26926
H	1.74310	1.36864	0.17701

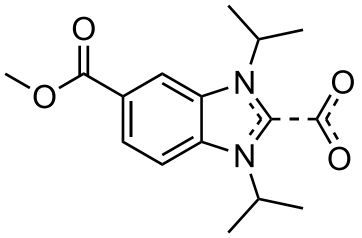

-1031.152379 Hartree

C	4.17642	-1.20638	-0.19852
O	4.52200	-1.36254	0.90748
O	4.18383	-1.18966	-1.36852
H	0.70011	-2.65748	-2.02965
H	2.63904	2.20064	-2.06463
H	-0.87297	-2.84682	-1.23655
H	0.31586	-4.14722	-1.15534
H	1.61814	3.42653	-1.29399
C	0.19314	-3.06283	-1.15203
H	3.37432	3.56345	-1.20813
C	2.54304	2.85642	-1.19730
C	0.78975	-2.49557	0.12352
H	-2.06868	-1.42409	0.03565
C	-1.66046	-0.42552	0.02298
N	1.59274	0.97641	0.08305
C	-2.50303	0.68034	-0.02575
C	-0.29686	-0.18966	0.05404
C	1.95444	-0.32767	0.12030
C	0.21885	1.11431	0.03966
C	-1.98465	1.98242	-0.04407
N	0.80501	-1.03099	0.10222
H	-2.67621	2.81368	-0.08435
C	-0.62548	2.21932	-0.01192
H	1.84648	-2.76294	0.15707
H	-0.24399	3.22989	-0.02882
C	2.58499	2.05424	0.09053
H	3.53785	1.52583	0.13491
C	0.12328	-3.02652	1.37981
H	0.24412	-4.11040	1.42018

C	2.45971	2.91608	1.33404
H	-0.94546	-2.80855	1.39993
H	3.28974	3.62393	1.36592
H	1.53135	3.48913	1.34407
H	0.58213	-2.59675	2.27214
H	2.49787	2.30185	2.23558
H	-6.19823	-0.58955	-0.97808
C	-3.97412	0.52113	-0.06030
O	-4.75912	1.44174	-0.10749
O	-4.35371	-0.75746	-0.03319
C	-5.76132	-0.99243	-0.06399
H	-5.88065	-2.07285	-0.03645
H	-6.24531	-0.53895	0.80134

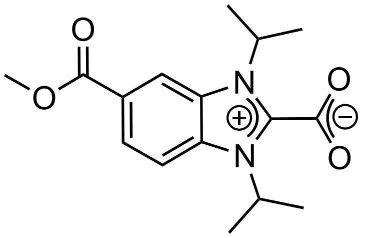

-1031.176806 Hartree

C	-3.51861	-2.02833	-1.13628
C	-2.36201	-2.22743	-0.17850
C	-2.79890	-2.64034	1.21432
N	-1.47394	-1.04969	-0.10659
C	-1.81198	0.24419	0.00198
N	-0.70485	0.98938	0.08204
C	-0.58713	2.45895	0.16386
C	-1.54867	3.06642	1.16431
C	-0.69305	3.07041	-1.22039
C	0.39707	0.15125	0.02203
C	-0.09603	-1.14934	-0.09200
C	0.75590	-2.24414	-0.16360
C	2.10969	-1.98340	-0.12820
C	2.60810	-0.67583	-0.02491
C	4.08074	-0.50011	-0.00131
O	4.44135	0.77754	0.10142
C	5.84710	1.03037	0.13068
O	4.87277	-1.41198	-0.06731
C	1.75404	0.41680	0.05223
H	-3.99717	-2.99641	-1.29111
H	-3.17304	-1.66049	-2.10322
H	-4.27015	-1.34619	-0.74122
H	-1.72293	-3.00570	-0.59416
H	-3.42998	-1.86977	1.65811
H	-1.93535	-2.80876	1.86022
H	-3.36639	-3.57019	1.15107
H	0.42245	2.61372	0.54328
H	-2.57831	3.05164	0.80943
H	-1.26363	4.10867	1.31462
H	-1.49343	2.55564	2.12648
H	-1.69233	2.91499	-1.62826
H	0.04044	2.63055	-1.89843
H	-0.49952	4.14228	-1.15452
H	0.38570	-3.25706	-0.23902
H	2.81645	-2.80084	-0.18056
H	6.31876	0.67643	-0.78616
H	6.30605	0.54086	0.98984
H	5.95063	2.10941	0.21360
H	2.14112	1.42236	0.12753
C	-3.24790	0.78430	0.03280
O	-3.86169	0.52900	1.07625
O	-3.58221	1.39649	-0.98883

3. Optimised structures in THF and energies

HCO_3^-
-264.374074 Hartree

O	1.21421	0.43603	0.00000
C	0.00000	0.15303	0.00000
O	-0.28679	-1.22550	0.00000
O	-0.99975	0.88041	0.00000
H	0.57861	-1.64569	0.00000

H_2O
-76.384997 Hartree

O	0.00000	0.00000	0.11784
H	0.00000	0.75949	-0.47136
H	0.00000	-0.75949	-0.47136

CO_2
-188.473067 Hartree

O	0.00000	0.00000	1.15737
C	0.00000	0.00000	0.00000
O	0.00000	0.00000	-1.15737

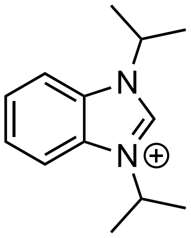

-615.479772

C	-2.59926	-2.34043	-0.65482
C	-2.48837	-0.92955	-0.12184
C	-3.04990	-0.79345	1.28159
N	-1.09274	-0.46105	-0.14786
C	0.00000	-1.21428	-0.14754
N	1.09274	-0.46105	-0.14786
C	2.48837	-0.92954	-0.12184
C	3.04990	-0.79344	1.28159
C	2.59928	-2.34042	-0.65482
C	0.69836	0.86710	-0.14536
C	1.42840	2.04818	-0.16020
C	0.70100	3.22265	-0.17321
C	-0.70101	3.22265	-0.17321
C	-1.42841	2.04818	-0.16021
C	-0.69837	0.86710	-0.14536
H	-3.65537	-2.60341	-0.71501
H	-2.11908	-3.06279	0.00903
H	-2.16980	-2.43051	-1.65381
H	-3.01984	-0.25470	-0.79533
H	-2.53581	-1.47010	1.96744
H	-4.10933	-1.05250	1.27231
H	-2.95143	0.22545	1.65791
H	0.00001	-2.29038	-0.15697
H	3.01985	-0.25468	-0.79532
H	2.53581	-1.47009	1.96744
H	2.95143	0.22545	1.65792
H	4.10933	-1.05250	1.27231
H	2.11909	-3.06278	0.00901
H	3.65538	-2.60340	-0.71501
H	2.16981	-2.43049	-1.65382
H	2.51056	2.04807	-0.15996
H	1.22715	4.16874	-0.18340
H	-1.22717	4.16874	-0.18340
H	-2.51057	2.04806	-0.15997

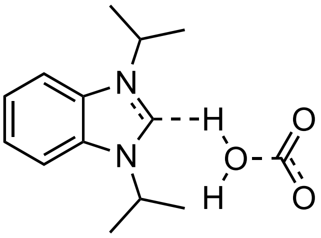

-879.829239 Hartree

C	0.29374	0.39266	0.47468
H	1.67792	0.69490	0.65455
C	0.71952	-2.03067	0.43215
C	-0.70652	2.67785	0.32471
C	0.84755	-2.75554	-0.89476
H	1.59376	-3.54638	-0.80257
H	-0.09325	-3.21525	-1.20145
H	1.17046	-2.07129	-1.68086
C	0.28791	-2.93687	1.57056
H	-0.66926	-3.42030	1.37051
H	1.03516	-3.72000	1.70854
C	3.72555	-0.05646	-0.71837
O	3.24852	0.42371	-1.68332
O	4.45610	-0.79746	-0.15775
O	2.83618	0.89775	0.72113
H	3.16146	0.49686	1.53239
H	0.20378	-2.37581	2.50296
H	1.29293	2.90940	1.12343
H	0.02619	2.78414	2.35640
C	0.26636	3.18280	1.36897
H	0.20352	4.27118	1.41440
H	-2.16674	-2.91097	-0.21935
N	-0.15941	-0.86299	0.31619
C	-2.44150	-1.86598	-0.22376
C	-1.51561	-0.86015	0.02771
H	-4.48928	-2.23744	-0.68866
C	-3.74402	-1.47798	-0.48673
C	-1.89588	0.48549	0.01678
N	-0.75511	1.21275	0.30569
C	-4.11923	-0.13083	-0.50053
C	-3.20141	0.87428	-0.25036
H	0.61401	2.91527	-1.36568
H	-3.49250	1.91642	-0.26792
C	-0.39441	3.20670	-1.06412
H	-0.45331	4.29654	-1.06905
H	-1.10146	2.82206	-1.80144
H	-1.71281	2.99250	0.60826
H	1.69057	-1.60506	0.68468
H	-5.14811	0.13121	-0.71432

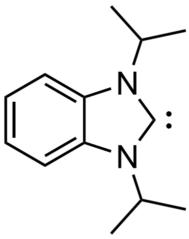

-614.998484 Hartree

C	3.13753	-1.80105	-0.83397
C	2.68508	-0.51728	-0.17253
C	3.25057	-0.36976	1.23043
N	1.22677	-0.39971	-0.14526
C	0.35546	-1.43150	-0.05969
N	-0.86662	-0.84659	0.00067
C	-2.09593	-1.63317	0.08706
C	-2.96552	-1.44381	-1.14367
C	-2.84023	-1.36405	1.38342
C	0.58229	0.82437	-0.12614
C	-0.78339	0.53740	-0.03794
C	-1.72377	1.56081	-0.00880
C	-1.25410	2.86404	-0.06424
C	0.11189	3.14563	-0.14659
C	1.05314	2.12896	-0.17777
H	4.22527	-1.78568	-0.92607
H	2.70869	-1.90662	-1.83167
H	2.85575	-2.67385	-0.24514
H	3.03363	0.32053	-0.78210
H	4.34183	-0.36078	1.19756
H	2.93170	-1.20607	1.85675
H	2.91559	0.55801	1.69802
H	-1.74078	-2.66376	0.10204
H	-3.81573	-2.12694	-1.09860
H	-2.40038	-1.66057	-2.05206
H	-3.35708	-0.42803	-1.21709
H	-3.68173	-2.05385	1.47019
H	-3.23600	-0.34811	1.42641
H	-2.18447	-1.51434	2.24303
H	-2.78426	1.36259	0.05560
H	-1.96413	3.68195	-0.04144
H	0.44139	4.17684	-0.18472
H	2.11144	2.34916	-0.23600

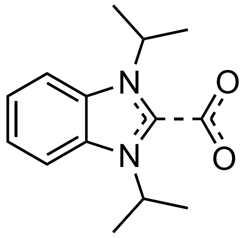

-803.459159 Hartree

C	3.32674	0.00037	0.00012
O	3.52062	-0.23547	-1.12909
O	3.52023	0.23624	1.12939
H	0.53587	2.64553	2.12621
H	0.52975	-2.59683	2.17698
H	-0.83311	3.39344	1.28853
H	0.75220	4.16513	1.24653
H	-0.83536	-3.36540	1.35224
C	0.23962	3.20163	1.23501
H	0.75097	-4.13550	1.33225
C	0.23717	-3.17318	1.29748
C	0.62057	2.44238	-0.02348
H	-2.44699	2.49852	-0.01632
C	-2.43119	1.41797	-0.00959
N	0.09483	-1.07703	0.00747
C	-3.61412	0.69767	-0.00455
C	-1.24092	0.70027	-0.00485
C	0.90847	0.00016	-0.00018
C	-1.24070	-0.70058	0.00479
C	-3.61390	-0.69875	0.00485
N	0.09451	1.07713	-0.00771
H	-4.55688	-1.23174	0.00827
C	-2.43074	-1.41867	0.00970
H	1.70170	2.30099	-0.02357
H	-2.44616	-2.49922	0.01645
C	0.62125	-2.44214	0.02327
H	1.70235	-2.30046	0.02316
C	0.23609	3.17346	-1.29755
H	0.74978	4.13584	-1.33238
C	0.24031	-3.20161	-1.23510
H	-0.83647	3.36558	-1.35203
H	0.75319	-4.16495	-1.24664
H	-0.83236	-3.39375	-1.28843
H	0.52851	2.59721	-2.17717
H	0.53624	-2.64548	-2.12640
H	-4.55727	1.23037	-0.00783

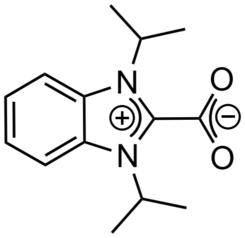

-803.486147

C	-3.19141	-0.36840	1.27297
C	-2.46135	-0.74795	-0.00124
C	-3.20175	-0.35532	-1.26540
N	-1.09367	-0.20601	-0.00315
C	-0.00161	-0.97169	-0.00421
C	-0.00392	-2.50342	-0.00908
O	0.02551	-2.99921	-1.14209
O	-0.03480	-3.00898	1.11944
N	1.09285	-0.20949	-0.00297
C	2.45891	-0.75526	0.00366
C	3.18330	-0.38239	1.28311
C	3.20676	-0.35975	-1.25518
C	0.70191	1.12399	-0.00143
C	1.42511	2.30953	-0.00041
C	0.70442	3.49014	0.00005
C	-0.69264	3.49251	-0.00052
C	-1.41744	2.31441	-0.00167
C	-0.69844	1.12629	-0.00193
H	-4.15237	-0.88419	1.29728
H	-3.38405	0.70365	1.32984
H	-2.61957	-0.66847	2.15222
H	-2.32285	-1.82923	-0.00713
H	-3.39860	0.71657	-1.30795
H	-4.16110	-0.87412	-1.28880
H	-2.63541	-0.64286	-2.15239
H	2.31758	-1.83612	-0.00805
H	3.37616	0.68929	1.34612
H	4.14385	-0.89886	1.30948
H	2.60708	-0.68636	2.15817
H	3.40733	0.71164	-1.29213
H	2.64375	-0.64163	-2.14609
H	4.16447	-0.88164	-1.27616
H	2.50523	2.32100	-0.00069
H	1.23769	4.43252	0.00058
H	-1.22269	4.43670	-0.00023
H	-2.49749	2.32993	-0.00244

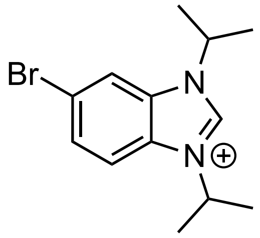

-3188.801099 Hartree

C	-2.43571	3.31911	-0.69310
C	-1.18318	2.70806	-0.10647
C	-0.91651	3.17738	1.31190
N	-1.24506	1.23579	-0.13963
C	-2.33762	0.48070	-0.16830
N	-2.02218	-0.80842	-0.15736
C	-2.95337	-1.95139	-0.14836
C	-3.04240	-2.52709	1.25303
C	-4.30614	-1.55973	-0.69890
C	-0.64245	-0.91002	-0.11844
C	0.20653	-2.00754	-0.11001
C	1.56258	-1.75153	-0.08776
C	2.04304	-0.43579	-0.07703
C	1.21347	0.66701	-0.08780
C	-0.14717	0.39505	-0.10735
H	-2.29337	4.39774	-0.75839
H	-3.30719	3.14195	-0.05923
H	-2.63779	2.94184	-1.69677
H	-0.33238	2.96174	-0.74158
H	-1.76180	2.93745	1.96018
H	-0.77791	4.25913	1.31246
H	-0.01785	2.71881	1.72625
H	-3.34206	0.86378	-0.20857
H	-2.49755	-2.68394	-0.81706
H	-3.48987	-1.80370	1.93755
H	-2.06144	-2.80997	1.63643
H	-3.67039	-3.41858	1.23543
H	-4.82914	-0.86771	-0.03521
H	-4.91617	-2.45945	-0.77877
H	-4.22591	-1.11528	-1.69219
H	-0.16880	-3.02181	-0.11941
H	2.26195	-2.57664	-0.07915
Br	3.91364	-0.16459	-0.04656
H	1.59450	1.67837	-0.08185

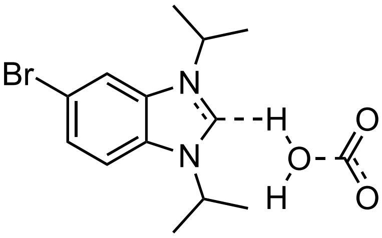

-3453.154718 Hartree

C	1.47672	0.38728	0.53417
H	2.85918	0.74673	0.58930
C	2.00352	-2.01539	0.43369
C	0.36388	2.62880	0.49878
C	2.06032	-2.72291	-0.90720
H	2.85229	-3.47313	-0.88406
H	1.12326	-3.23147	-1.13983
H	2.28136	-2.01789	-1.70992
C	1.69714	-2.94832	1.59047
H	0.75077	-3.47302	1.45270
H	2.48643	-3.69797	1.66490
C	4.77478	0.10272	-0.97483
O	4.18108	0.56299	-1.88486
O	5.59596	-0.61108	-0.50968
O	4.00813	0.99339	0.54303
H	4.43096	0.60031	1.31226
H	1.65574	-2.39938	2.53283
H	2.43483	2.95349	1.04494
H	1.34110	2.74969	2.42429
C	1.43479	3.16987	1.42123
H	1.32298	4.25284	1.49214
H	-0.89258	-3.01491	0.00143
N	1.06817	-0.88577	0.39899
C	-1.20527	-1.98145	0.03649
C	-0.30465	-0.94081	0.22354
H	-3.26897	-2.44394	-0.26332
C	-2.54172	-1.65749	-0.11386
C	-0.74277	0.38634	0.25984
N	0.38198	1.16227	0.46216
C	-2.95613	-0.32482	-0.07787
C	-2.07933	0.72676	0.10530
Br	-4.80029	0.05443	-0.29462
H	1.45072	2.94203	-1.33830
H	-2.41718	1.75355	0.12094
C	0.47547	3.18362	-0.91020
H	0.36696	4.26933	-0.89134
H	-0.29961	2.77324	-1.56007
H	-0.61222	2.89132	0.91211
H	2.97121	-1.54897	0.61755

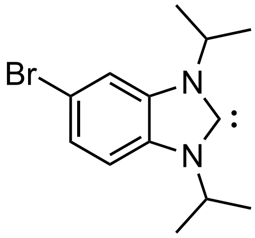

-3188.324158 Hartree

C	2.30551	3.49202	-0.87579
C	1.16049	2.80273	-0.16600
C	0.96857	3.32428	1.24830
N	1.32591	1.34778	-0.14862
C	2.51016	0.69206	-0.10083
N	2.17666	-0.61969	-0.02553
C	3.19121	-1.67168	0.03942
C	3.12271	-2.59069	-1.16758
C	3.12734	-2.42242	1.35787
C	0.25474	0.47783	-0.09120
C	0.80366	-0.80621	-0.01647
C	-0.01895	-1.92363	0.04545
C	-1.38997	-1.72373	0.03609
C	-1.91329	-0.43354	-0.03537
C	-1.11599	0.69434	-0.09947
H	2.07958	4.55705	-0.95553
H	2.44699	3.09337	-1.88153
H	3.24050	3.38015	-0.32691
H	0.24656	2.98666	-0.73665
H	0.75286	4.39424	1.22817
H	1.87582	3.16652	1.83588
H	0.14102	2.81880	1.74972
H	4.13134	-1.12147	0.00057
H	3.97509	-3.27230	-1.15318
H	3.16023	-2.01552	-2.09447
H	2.21444	-3.19522	-1.17312
H	3.96437	-3.12015	1.42062
H	2.20559	-2.99781	1.45759
H	3.19409	-1.73090	2.19972
H	0.37576	-2.92799	0.10103
H	-2.05634	-2.57424	0.08608
Br	-3.79895	-0.21982	-0.04096
H	-1.53976	1.68760	-0.15165

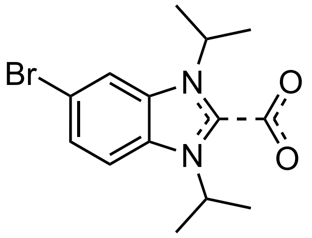

-3376.780512 Hartree

C	4.29061	-0.91877	-0.09326
O	4.56273	-1.01997	1.04050
O	4.40176	-0.93138	-1.25876
H	0.92903	-2.66432	-2.09389
H	2.42383	2.35513	-2.10905
H	-0.61307	-2.98329	-1.28461
H	0.67626	-4.18442	-1.22437
H	1.31663	3.48393	-1.31125
C	0.46785	-3.11327	-1.21246
H	3.05548	3.76834	-1.25305
C	2.28782	2.99279	-1.23380
C	1.03228	-2.50425	0.05862
H	-1.91091	-1.69741	0.01208
C	-1.59220	-0.66627	0.01029
Br	-4.35799	-0.06040	-0.03301
N	1.53034	1.02321	0.04158
C	-2.50976	0.36717	-0.00991
C	-0.25087	-0.30485	0.02649
C	2.00473	-0.23989	0.05675
C	0.14441	1.03850	0.02216
C	-2.13604	1.70987	-0.01476
N	0.91962	-1.04449	0.04780
H	-2.89247	2.48286	-0.03134
C	-0.79666	2.05899	0.00105
H	2.10833	-2.68000	0.07496
H	-0.51191	3.10086	-0.00428
C	2.42033	2.18605	0.04532
H	3.41748	1.74564	0.07109
C	0.43324	-3.09706	1.32153
H	0.63939	-4.16829	1.35173
C	2.23718	3.02348	1.29820
H	-0.64895	-2.96454	1.36366
H	3.00361	3.79983	1.32904
H	1.26373	3.51552	1.32519
H	0.87196	-2.63826	2.20932
H	2.33815	2.40731	2.19337

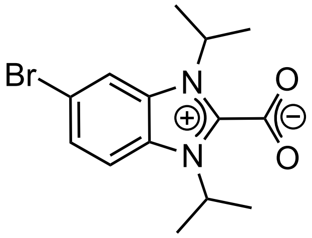

-3376.807140 Hartree

C	-0.40528	3.15869	1.26801
C	-1.01120	2.59560	-0.00339
C	-0.40348	3.16204	-1.27237
N	-0.94817	1.12517	-0.00468
C	-2.02633	0.33777	-0.00642
C	-3.47506	0.83714	-0.00705
O	-3.96281	0.95220	-1.13747
O	-3.93018	1.04806	1.12316
N	-1.65781	-0.94435	-0.00616
C	-2.61529	-2.06316	0.00190
C	-2.50479	-2.85974	1.28782
C	-2.47403	-2.90924	-1.24884
C	-0.27088	-1.00284	-0.00350
C	0.62303	-2.06382	-0.00136
C	1.97320	-1.76796	-0.00010
C	2.40575	-0.44169	-0.00080
C	1.53474	0.63034	-0.00263
C	0.18158	0.32160	-0.00360
H	-0.57228	4.23638	1.29157
H	0.67020	2.98474	1.32197
H	-0.87544	2.71973	2.14909
H	-2.07788	2.81993	-0.00315
H	0.67208	2.98830	-1.32532
H	-0.57045	4.23980	-1.29312
H	-0.87238	2.72554	-2.15537
H	-3.59205	-1.57990	-0.02064
H	-1.55724	-3.39576	1.35793
H	-3.30891	-3.59642	1.31625
H	-2.60562	-2.20980	2.15812
H	-1.53485	-3.46337	-1.26695
H	-2.53494	-2.29143	-2.14588
H	-3.28942	-3.63338	-1.27681
H	0.29383	-3.09184	-0.00110
H	2.69597	-2.57250	0.00127
Br	4.26745	-0.09727	0.00112
H	1.89280	1.64822	-0.00282

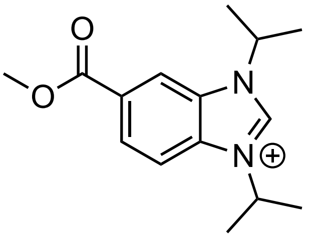

-843.171705 Hartree

C	-4.44220	-1.14198	-0.75079
C	-3.14508	-1.71796	-0.22878
C	-3.30934	-2.40407	1.11493
N	-2.09945	-0.68177	-0.13335
C	-2.27796	0.62613	0.02774
N	-1.11435	1.25295	0.13268
C	-0.89767	2.70539	0.25877
C	-2.11437	3.38570	0.84462
C	-0.50130	3.28078	-1.08850
C	-0.10916	0.30552	0.03468
C	-0.73966	-0.92987	-0.13242
C	-0.01257	-2.10610	-0.26304
C	1.35950	-1.99005	-0.21991
C	1.99583	-0.74896	-0.04879
C	3.48202	-0.74294	-0.01074
O	3.98093	0.47793	0.16652
C	5.40691	0.56574	0.21735
O	4.16070	-1.73468	-0.12616
C	1.26883	0.42448	0.08001
H	-5.14010	-1.96248	-0.91706
H	-4.29916	-0.62075	-1.69866
H	-4.90321	-0.46089	-0.03245
H	-2.75697	-2.43224	-0.95710
H	-3.69684	-1.70420	1.85830
H	-2.36334	-2.80989	1.47556
H	-4.01805	-3.22693	1.01439
H	-0.06462	2.80047	0.95840
H	-2.96405	3.35438	0.15901
H	-1.87234	4.43430	1.01737
H	-2.40494	2.94272	1.79843
H	-1.32594	3.19349	-1.79910
H	0.37145	2.77370	-1.50204
H	-0.25820	4.33741	-0.97063
H	-0.49463	-3.06580	-0.39042
H	1.97981	-2.87088	-0.31699
H	5.84366	0.23319	-0.72438
H	5.79823	-0.04016	1.03445
H	5.63161	1.61593	0.38600
H	1.75485	1.38016	0.21060

H	-3.23634	1.11325	0.06730

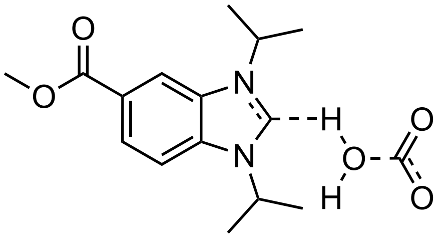

-1107.524644

C	1.42395	0.39668	0.53221
H	2.78385	0.83919	0.59523
C	2.10716	-1.96839	0.40286
C	0.15907	2.55699	0.52595
C	2.19659	-2.66795	-0.94054
H	3.03524	-3.36577	-0.92649
H	1.29186	-3.23487	-1.16622
H	2.36467	-1.94822	-1.74304
C	1.87438	-2.92028	1.56143
H	0.95805	-3.49910	1.43836
H	2.70754	-3.62224	1.62154
C	4.73218	0.34999	-0.97961
O	4.09771	0.77802	-1.87815
O	5.61009	-0.30689	-0.53411
O	3.91512	1.15262	0.55535
H	4.36207	0.77032	1.31633
H	1.81614	-2.37604	2.50567
H	2.22643	3.03427	0.96334
H	1.22899	2.73870	2.39702
C	1.23479	3.17095	1.39478
H	1.04456	4.24162	1.48307
H	-0.70629	-3.15364	-0.03645
N	1.09891	-0.90259	0.38120
C	-1.09319	-2.14618	0.01121
C	-0.26407	-1.04654	0.20876
H	-3.12746	-2.72601	-0.28817
C	-2.44374	-1.90256	-0.13023
C	-0.79090	0.24852	0.26279
N	0.28242	1.09618	0.47171
C	-2.97004	-0.60337	-0.07918
C	-2.14360	0.49622	0.11722
H	1.11882	2.95928	-1.36389
H	-2.54718	1.49790	0.14917
C	0.15375	3.12801	-0.88119
H	-0.02987	4.20309	-0.84422
H	-0.62522	2.66907	-1.49267
H	-0.81071	2.74360	0.99266

H	3.04428	-1.44010	0.57731
H	-6.78018	0.55856	0.46060
C	-4.43430	-0.44667	-0.24374
O	-5.20175	-1.36538	-0.41195
O	-4.82240	0.82874	-0.18479
C	-6.22330	1.05846	-0.33232
H	-6.35564	2.13556	-0.26182
H	-6.57185	0.69962	-1.30099

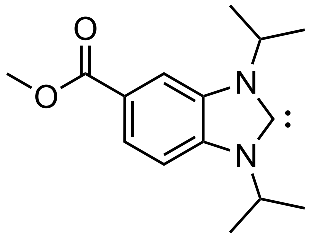

-842.695391 Hartree

C	-4.38335	-1.21788	-0.92095
C	-3.14427	-1.69150	-0.19190
C	-3.46138	-2.21151	1.19987
N	-2.12223	-0.64473	-0.12006
C	-2.37221	0.68294	0.02871
N	-1.14800	1.24906	0.12782
C	-0.87999	2.68569	0.22296
C	-1.98439	3.41961	0.95193
C	-0.62499	3.26349	-1.15912
C	-0.13327	0.31129	0.04028
C	-0.77246	-0.92289	-0.10923
C	-0.04655	-2.10317	-0.21207
C	1.33087	-2.00384	-0.17321
C	1.97524	-0.76646	-0.03245
C	3.45443	-0.76185	-0.00444
O	3.96258	0.46726	0.12609
C	5.38603	0.54871	0.16100
O	4.14325	-1.75273	-0.08947
C	1.24437	0.41462	0.07591
H	-5.06475	-2.06158	-1.04573
H	-4.13588	-0.82612	-1.90890
H	-4.90087	-0.43866	-0.36161
H	-2.69330	-2.49821	-0.77479
H	-3.92544	-1.42653	1.80120
H	-2.55812	-2.54970	1.71147
H	-4.15430	-3.05286	1.13815
H	0.03550	2.77226	0.81391
H	-2.91466	3.40658	0.38395
H	-1.68267	4.45924	1.09275
H	-2.17358	2.97978	1.93235

H	-1.52926	3.20085	-1.76873
H	0.17539	2.72701	-1.67239
H	-0.33524	4.31302	-1.07962
H	-0.53162	-3.06451	-0.31843
H	1.94169	-2.89348	-0.25151
H	5.81689	0.16835	-0.76565
H	5.78435	-0.01749	1.00337
H	5.61942	1.60468	0.27609
H	1.74285	1.36737	0.18314

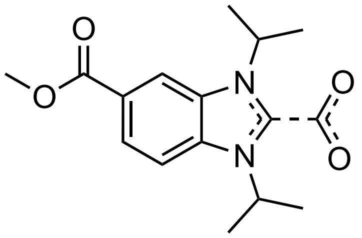

-1031.151247 Hartree

C	4.15948	1.19810	-0.16127
O	4.25256	1.08909	-1.32380
O	4.44042	1.45396	0.94578
H	0.60389	2.61533	2.23620
H	2.50253	-2.26600	2.23933
H	-0.93427	2.81146	1.38108
H	0.24728	4.12056	1.37798
H	1.52528	-3.45770	1.36853
C	0.13289	3.03572	1.34592
H	3.28225	-3.60733	1.38938
C	2.45844	-2.89280	1.34692
C	0.78843	2.49803	0.08673
H	-2.06806	1.42379	0.02317
C	-1.65843	0.42585	0.01227
N	1.59626	-0.97226	0.06708
C	-2.50063	-0.68024	-0.02608
C	-0.29465	0.19140	0.03720
C	1.95643	0.33198	0.09481
C	0.22205	-1.11191	0.02797
C	-1.98148	-1.98145	-0.04250
N	0.80675	1.03399	0.07589
H	-2.67455	-2.81158	-0.07623
C	-0.62210	-2.21713	-0.01638
H	1.84448	2.76823	0.10877
H	-0.24109	-3.22790	-0.03336
C	2.58815	-2.04938	0.09118
H	3.54103	-1.52083	0.13170
C	0.17993	3.05470	-1.18789
H	0.29903	4.13941	-1.20117

C	2.55075	-2.86873	-1.18592
H	-0.88609	2.83514	-1.26417
H	3.38343	-3.57417	-1.18742
H	1.62771	-3.44256	-1.27815
H	0.68085	2.64442	-2.06652
H	2.64639	-2.22430	-2.06151
H	-6.23375	0.53514	0.83017
C	-3.97252	-0.52449	-0.04936
O	-4.75560	-1.44559	-0.08038
O	-4.35419	0.75538	-0.03059
C	-5.76248	0.98267	-0.04538
H	-5.88834	2.06279	-0.02738
H	-6.21028	0.56743	-0.94863

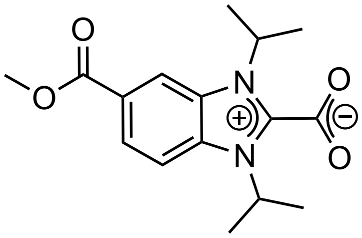

-1031.172561 Hartree

C	-3.51073	-2.02601	-1.15070
C	-2.36292	-2.22614	-0.18235
C	-2.81427	-2.63183	1.20819
N	-1.47257	-1.05064	-0.10657
C	-1.81269	0.24328	0.00281
N	-0.70469	0.98747	0.08828
C	-0.58921	2.45669	0.17340
C	-1.55410	3.05926	1.17388
C	-0.69626	3.07106	-1.20964
C	0.39779	0.15015	0.02926
C	-0.09464	-1.15080	-0.08747
C	0.75933	-2.24406	-0.15737
C	2.11312	-1.98273	-0.11974
C	2.61028	-0.67545	-0.01698
C	4.08325	-0.50013	0.00462
O	4.44368	0.78098	0.08161
C	5.84943	1.03116	0.10459
O	4.87427	-1.41226	-0.04300
C	1.75443	0.41584	0.06013
H	-3.99493	-2.99146	-1.30488
H	-3.15646	-1.66473	-2.11693
H	-4.26037	-1.33656	-0.76562
H	-1.72208	-3.00745	-0.59058
H	-3.44345	-1.85440	1.64229
H	-1.95834	-2.80333	1.86364
H	-3.38776	-3.55805	1.14519
H	0.41998	2.61183	0.55524
H	-2.58299	3.03961	0.81798
H	-1.27489	4.10274	1.32717
H	-1.49959	2.54727	2.13539
H	-1.69480	2.91072	-1.61705
H	0.03874	2.63692	-1.88993
H	-0.50861	4.14398	-1.14294
H	0.39154	-3.25779	-0.23411
H	2.82226	-2.79806	-0.17119
H	6.32000	0.65931	-0.80579
H	6.30967	0.55594	0.97106
H	5.95637	2.11142	0.16797
H	2.14186	1.42121	0.13540
C	-3.25099	0.78448	0.02618
O	-3.86975	0.51626	1.06184
O	-3.56899	1.40557	-0.99384

S13 References

(1) Major, G. H.; Fairley, N.; Sherwood, P. M. A.; Linford, M. R.; Terry, J.; Fernandez, V.; Artyushkova, K. Practical Guide for Curve Fitting in X-Ray Photoelectron Spectroscopy. *J. Vac. Sci. Technol. A* **2020**, *38* (6), 061203. https://doi.org/10.1116/6.0000377.

(2) Kolářová, L.; Prokeš, L.; Kučera, L.; Hampl, A.; Peňa-Méndez, E.; Vaňhara, P.; Havel, J. Clusters of Monoisotopic Elements for Calibration in (TOF) Mass Spectrometry. *J. Am. Soc. Mass Spectrom.* **2017**, *28* (3), 419–427. https://doi.org/10.1007/s13361-016-1567-x.

(3) Alvaro Fernandez Galiana; Simon Vilms Pedersen; Molly M. Stevens. B-Raman: An Open-Source, Versatile Platform for Automated Brightfield and Raman Microspectroscopy; 2025; Vol. PC13327, p PC133270C. https://doi.org/10.1117/12.3043951.

(4) Georgiev, D.; Fernández-Galiana, Á.; Vilms Pedersen, S.; Papadopoulos, G.; Xie, R.; Stevens, M. M.; Barahona, M. Hyperspectral Unmixing for Raman Spectroscopy via Physics-Constrained Autoencoders. *Proc. Natl. Acad. Sci.* **2024**, *121* (45), e2407439121. https://doi.org/10.1073/pnas.2407439121.

(5) Whitaker, D. A.; Hayes, K. A Simple Algorithm for Despiking Raman Spectra. *Chemom. Intell. Lab. Syst.* **2018**, *179*, 82–84. https://doi.org/10.1016/j.chemolab.2018.06.009.

(6) Eilers, P. H. C. A Perfect Smoother. *Anal. Chem.* **2003**, *75* (14), 3631–3636. https://doi.org/10.1021/ac034173t.

(7) Ye, J.; Tian, Z.; Wei, H.; Li, Y. Baseline Correction Method Based on Improved Asymmetrically Reweighted Penalized Least Squares for the Raman Spectrum. *Appl. Opt.* **2020**, *59* (34), 10933–10943. https://doi.org/10.1364/AO.404863.

(8) Georgiev, D.; Pedersen, S. V.; Xie, R.; Fernández-Galiana, Á.; Stevens, M. M.; Barahona, M. RamanSPy: An Open-Source Python Package for Integrative Raman Spectroscopy Data Analysis. *Anal. Chem.* **2024**, *96* (21), 8492–8500. https://doi.org/10.1021/acs.analchem.4c00383.

(9) *Erb, D. (2022). pybaselines: A Python library of algorithms for the baseline correction of experimental data (1.0.0). Zenodo. https://doi.org/10.5281/zenodo.7255880*.

(10) Amit, E.; Dery, L.; Dery, S.; Kim, S.; Roy, A.; Hu, Q.; Gutkin, V.; Eisenberg, H.; Stein, T.; Mandler, D.; Dean Toste, F.; Gross, E. Electrochemical Deposition of N-Heterocyclic Carbene Monolayers on Metal Surfaces. *Nat. Commun.* **2020**, *11* (1), 5714. https://doi.org/10.1038/s41467-020-19500-7.

(11) Gaussian 16, Revision C.01, M. J. Frisch, G. W. Trucks, H. B. Schlegel, G. E. Scuseria, M. A. Robb, J. R. Cheeseman, G. Scalmani, V. Barone, G. A. Petersson, H. Nakatsuji, X. Li, M. Caricato, A. V. Marenich, J. Bloino, B. G. Janesko, R. Gomperts, B. Mennucci, H. P. Hratchian, J. V. Ortiz, A. F. Izmaylov, J. L. Sonnenberg, D. Williams-Young, F. Ding, F. Lipparini, F. Egidi, J. Goings, B. Peng, A. Petrone, T. Henderson, D. Ranasinghe, V. G. Zakrzewski, J. Gao, N. Rega, G. Zheng, W. Liang, M. Hada, M. Ehara, K. Toyota, R. Fukuda, J. Hasegawa, M. Ishida, T. Nakajima, Y. Honda, O. Kitao, H. Nakai, T. Vreven, K. Throssell, J. A. Montgomery, Jr., J. E. Peralta, F. Ogliaro, M. J. Bearpark, J. J. Heyd, E. N. Brothers, K. N. Kudin, V. N. Staroverov, T. A. Keith, R. Kobayashi, J. Normand, K. Raghavachari, A. P. Rendell, J. C. Burant, S. S. Iyengar, J. Tomasi, M. Cossi, J. M. Millam, M. Klene, C. Adamo, R. Cammi, J. W.

Ochterski, R. L. Martin, K. Morokuma, O. Farkas, J. B. Foresman, and D. J. Fox, Gaussian, Inc., Wallingford CT, 2016.
(12) C. Adamo and V. Barone, “Toward Reliable Density Functional Methods without Adjustable Parameters: The PBE0 Model,” J. Chem. Phys., 110 (1999) 6158-69. DOI: 10.1063/1.478522.
(13) Grimme, S.; Ehrlich, S.; Goerigk, L. Effect of the Damping Function in Dispersion Corrected Density Functional Theory. *J. Comput. Chem.* **2011**, *32* (7), 1456–1465. https://doi.org/10.1002/jcc.21759.
(14) Marenich, A. V.; Cramer, C. J.; Truhlar, D. G. Universal Solvation Model Based on Solute Electron Density and on a Continuum Model of the Solvent Defined by the Bulk Dielectric Constant and Atomic Surface Tensions. *J. Phys. Chem. B* **2009**, *113* (18), 6378–6396. https://doi.org/10.1021/jp810292n.
(15) Li, X.; Frisch, M. J. Energy-Represented Direct Inversion in the Iterative Subspace within a Hybrid Geometry Optimization Method. *J. Chem. Theory Comput.* **2006**, *2* (3), 835–839. https://doi.org/10.1021/ct050275a.
(16) Gutheil, C.; Roß, G.; Amirjalayer, S.; Mo, B.; Schäfer, A. H.; Doltsinis, N. L.; Braunschweig, B.; Glorius, F. Tailored Monolayers of N-Heterocyclic Carbenes by Kinetic Control. *ACS Nano* **2024**, *18* (4), 3043–3052. https://doi.org/10.1021/acsnano.3c08045.
(17) Chandran, A.; Dominique, N. L.; Kaur, G.; Clark, V.; Nalaoh, P.; Ekowo, L. C.; Jensen, I. M.; Aloisio, M. D.; Crudden, C. M.; Arroyo-Currás, N.; Jenkins, D. M.; Camden, J. P. Forming N-Heterocyclic Carbene Monolayers: Not All Deposition Methods Are the Same. *Nanoscale* **2025**, *17,* 5413–5428, 10.1039.D4NR04428B. https://doi.org/10.1039/D4NR04428B.
(18) DeJesus, J. F.; Trujillo, M. J.; Camden, J. P.; Jenkins, D. M. *N* -Heterocyclic Carbenes as a Robust Platform for Surface-Enhanced Raman Spectroscopy. *J. Am. Chem. Soc.* **2018**, *140* (4), 1247–1250. https://doi.org/10.1021/jacs.7b12779.
(19) Huynh, H. V.; Lam, T. T.; Luong, H. T. T. Anion Influences on Reactivity and NMR Spectroscopic Features of NHC Precursors. *RSC Adv.* **2018**, *8* (61), 34960–34966. https://doi.org/10.1039/C8RA05839C.
(20) Guisado-Barrios, G.; Bouffard, J.; Donnadieu, B.; Bertrand, G. Crystalline 1 *H* - 1,2,3-Triazol-5-ylidenes: New Stable Mesoionic Carbenes (MICs). *Angew. Chem. Int. Ed.* **2010**, *49* (28), 4759–4762. https://doi.org/10.1002/anie.201001864.
